# A photogenerated triplet aura-nitrene for gold-mediated nitrene transfer

**DOI:** 10.1038/s41557-026-02152-3

**Published:** 2026-07-21

**Authors:** Jaime Martín, Marc Fernández-Sabaté, Bernhard Spingler, John H. Beale, Dominik Munz, Cristina Nevado

**Affiliations:** 1 Department of Chemistry, University of Zurich, Zurich, Switzerland; 2 Paul Scherrer Institute, Villigen, Switzerland; 3 Coordination Chemistry, Saarland University, Saarbrücken, Germany

**Keywords:** Chemical bonding, Reaction mechanisms

## Abstract

The isolation and characterization of metal-stabilized, low-valent nitrogen species remain central challenges in advancing nitrogen-atom transfer chemistry. Although gold has emerged as a powerful catalyst in C–N bond formation, proposed mechanisms have consistently bypassed gold nitrenes as key intermediates—leaving their chemistry largely uncharted. Here we report the characterization of a bona fide aura-nitrene, formed via photochemical N_2_ extrusion from a gold(III) azide complex. In crystallo and solid-state analyses reveal a diverse reactivity landscape, including a nitrene-to-nitro transformation via O_2_ activation, intra- and intermolecular C–H bond activation, CO fixation and alkyne addition/sigmatropic rearrangement. Computational investigations confirm a triplet ground state, with the nitrene best described as a nitrogen-centred diradical *σ*-bonded to a formal gold(III) centre. These findings highlight the dual electrophilic and diradical character of gold(III) metallonitrenes and establish a conceptual framework for gold-mediated nitrogen-atom transfer beyond conventional mechanistic paradigms.

**Figure:**
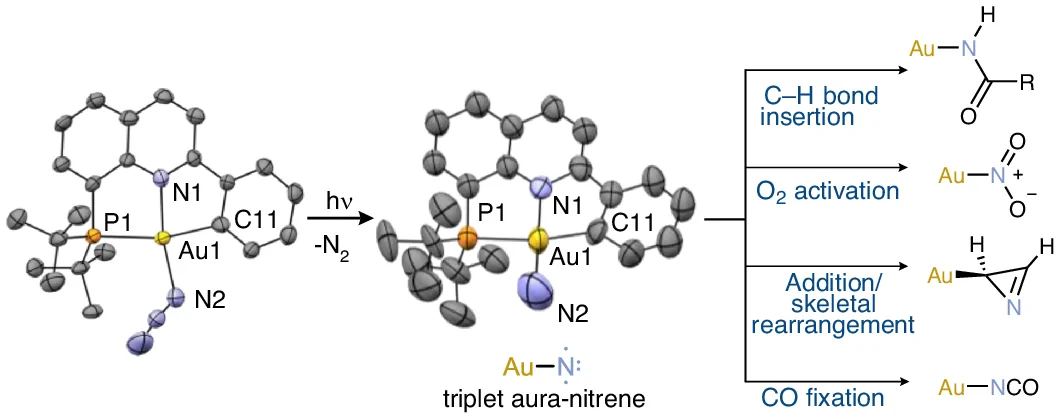

## Abstract (web summary)

Metal-stabilized nitrenes are important intermediates in nitrogen-atom transfer chemistry, but their isolation is challenging. Now a formal gold(III) aura-nitrene with triplet diradical character has been generated through photoirradiation of an azide precursor. The resulting nitrene enables a range of reactions, including O_2_ activation and C–H functionalization.

## Main

**Fig. 1:**
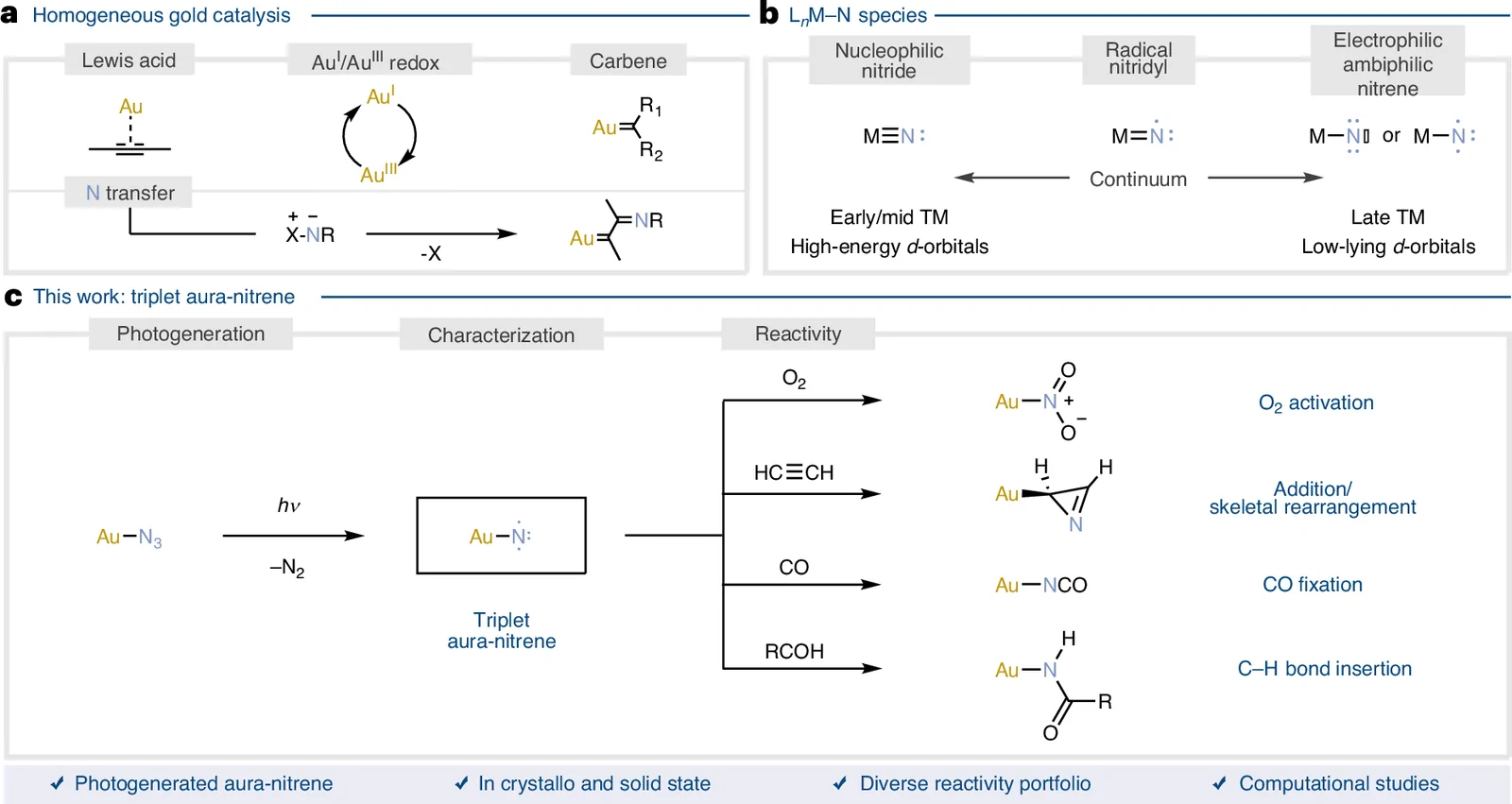
The chemistry of gold and L_*n*_M–N species. **a**, Classical reactivity modes in gold catalysis. **b**, Bonding spectrum for terminal metal nitrido species L_*n*_M–N. TM, transition metal. **c**, This work: characterization and reactivity of a triplet aura-nitrene.

Gold, the most electronegative metal in the periodic table, has profoundly impacted modern synthetic chemistry as a result of its carbophilic Lewis acid character and redox [Au(I)/Au(III)] reactivity^1,2^. Its ability to coordinate and concomitantly lower the energy of the LUMO of unsaturated systems (that is, alkynes, alkenes, allenes and so on) has unlocked molecular complexity-building transformations based on the incorporation of C-, N- and O-based nucleophiles onto these abundant feedstocks^3,4^. For the longest time, gold was considered redox inert due to its high oxidation potential (standard electrode potentials for Au^3+^/Au^+^ in aqueous solution *E*° = +1.41 V); however, recent advances in ligand design have enabled oxidative cross-coupling processes featuring the formation of C*sp*^2^–C*sp*^2^, –C*sp*, –C*sp*^3^, –O, –N and –F bonds. These processes not only complement but also expand the scope of classical protocols mediated by neighbouring group 10–11 metals^5,6^. Alongside Lewis acid and redox reactivity, gold carbenes have played a key role in the rise of gold catalysis^7^, with countless studies devoted to elucidating their structural and electronic properties^8–11^ (Fig. 1a). However, in stark contrast to this bountiful reactivity portfolio, gold’s ability to form terminal imido (L_*n*_Au–NR) or formal nitrido (L_*n*_Au–N) complexes has yet to be demonstrated^12–14^.

Nitrenes, neutral monovalent nitrogen species with only six valence electrons^15^, are versatile intermediates for introducing nitrogen atoms into organic molecules^16–18^, a process in high demand due to the ubiquitous presence of C–N bonds in pharmaceuticals and biologically active molecules^19^. The selection of bulky ligands has been crucial to achieve the recent characterization of few isolated organic nitrenes^20–22^. Besides the kinetic stability provided by steric protection, late transition metals of groups 9–11 have been pivotal to stabilizing these elusive species, thereby enabling controlled and (stereo)selective nitrogen-group transfer reactions such as C–H insertions, alkene aziridination and ring expansions, among others^23,24^. Despite their synthetic impact, the structural characterization and detailed bonding analysis of the L_*n*_M–NR intermediates underlying these transformations have only recently borne fruit^25^. Leveraging the advancements of in crystallo organometallic chemistry^26,27^, Powers and Chang pioneered the characterization of rhodium nitrenoids through the photochemical extrusion of N_2_ from azide derivatives^28,29^, and CO_2_ from dioxazolone precursors^30^, respectively.

Nitrogen-atom transfer (NAT) reactions represent a promising alternative for the formal addition of N atoms to organic molecules. However, the application of L_*n*_M–N complexes in NAT reactions remains largely untapped^31^. Depending on the formal oxidation state and position of the metal in the periodic table, terminal nitrido complexes can display nucleophilic nitride, radical nitridyl or electrophilic/ambiphilic nitrene character^32–34^ (Fig. 1b). Many structurally well-defined terminal nitrido complexes have been described for early/mid transition metals^35–38^, particularly in the context of N_2_ fixation^39^. However, later congeners—which feature enhanced electrophilicity at nitrogen, becoming powerful intermediates in NAT catalysis—have proven more difficult to attain^40–43^. Groundbreaking work by Schneider and coworkers unveiled the characterization of Pt(II) and Pd(II) metallonitrenes within crystalline matrices along with their catalytic application, including the C–H activation of aldehydes to produce amides^44–46^.

Despite gold’s proximity to, and complementary reactivity with, the group 9 and 10 metals, as well as its rich carbene chemistry, formal gold nitrido or nitrene intermediates remain conspicuously absent from the growing field of NAT. Interestingly, C–N bond-forming transformations involving gold-vinyl species that rearrange into α-imino carbenes—often referred to as gold-catalysed nitrene transfer reactions^47^—have been developed (Fig. 1a). Yet, the proposed mechanisms for these processes notably bypass gold nitrene intermediates, leaving the chemistry of these intriguing species unexplored^48–50^.

Driven by the uncharted reactivity landscape of gold nitrenes and our continued interest in the study of elusive gold species^51–53^, we report the photochemical generation and comprehensive characterization of a triplet aura-nitrene (Fig. 1c). Crystallographic, spectroscopic and computational analyses delineate its electronic structure, revealing a nitrogen-centred diradical *σ*-bonded to a gold(III) centre. Beyond single-crystal-to-single-crystal (sc-t-sc) transformations, this species exhibits versatile in solido reactivity, including a rare O_2_ activation and a distinctive alkyne addition/sigmatropic rearrangement. Furthermore, the metallonitrene engages in intra- and intermolecular C–H bond insertions and CO fixation. These findings underscore the critical importance of structurally well-defined, open-shell gold nitrene species that unlock reactivity patterns inaccessible through conventional mechanisms, establishing gold as a viable platform for umpolung-based NAT.

## Results and discussion

### In crystallo generation and characterization of a triplet aura-nitrene

**Fig. 2:**
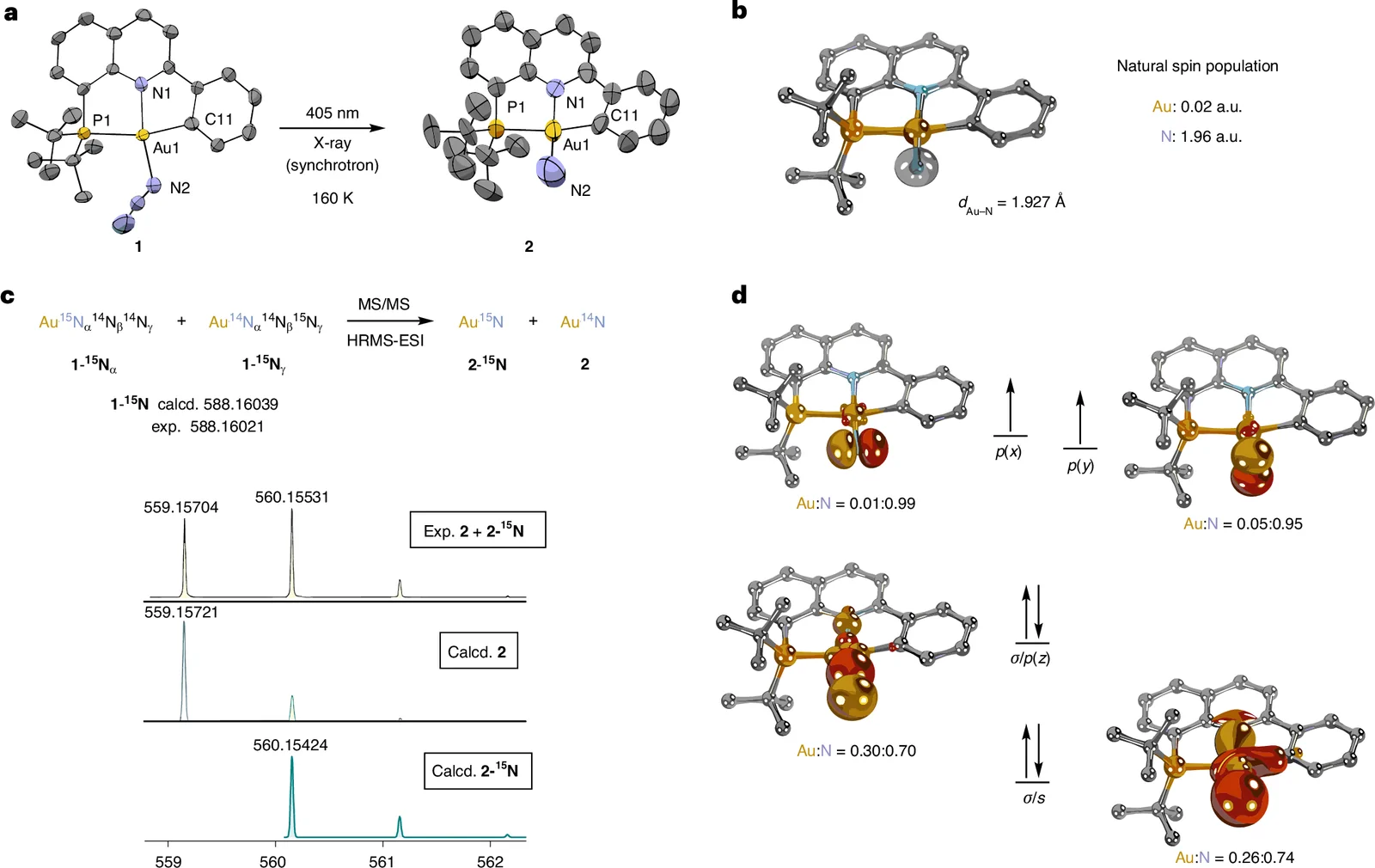
Synthesis and characterization of aura-nitrene 2. **a**, In crystallo formation of **2** (30% conversion). Ellipsoidal representation of **1** (dichloromethane/pentane) and **2** with 50% probability ellipsoids. The asymmetric units of the crystal structures of **1** and **2** contain two crystallographically independent molecules, and only one of them is depicted. Non-coordinating counter-anions, hydrogen atoms and the disordered azide anion of **2** are omitted for clarity. Selected distances (Å) in **1**: Au1–P1 2.400(2), Au1–N1 2.014(7), Au1–C11 2.046(9), Au1–N2 2.033(8); in **2**: Au1–P1 2.399(6), Au1–N1 1.95(2), Au1–C11 1.96(3), Au1–N2 1.978(18). Selected angles (°) in **1**: C11–Au1–P1 165.6(2), N1–Au1–N2 169.3(4); in **2**: C1–Au1–P1 168.9(8), N1–Au1–N2 175.4(14). **b**, Spin density as obtained at the ZORA-PBE0-D4/def2-TZVPP//ZORA-PBE0-D4/def2-SVP (*S* = 1) level of theory. **c**, Detection of **2** and **2-**^**15**^**N** by high resolution mass spectrometry-electrospray ionization (HRMS-ESI). calcd., calculated; exp., experimental. **d**, Natural orbitals pertinent to the triplet aura-nitrene **2** as well as their metal:nitrene contribution (Löwdin) as obtained at the ZORA-CASSCF(18,13)/def2-TZVPP level of theory.

The [(P^N^C)Au–N_3_]BF_4_ complex **1** was prepared in one step in 89% yield by reacting the corresponding hydroxo precursor^53^ with trimethylsilyl azide (TMSN_3_) for 1 h at room temperature. The complex displays the characteristic azide stretching vibration in the infrared (IR) spectrum at 2,048 cm^−1^. Ultraviolet–visible spectroscopic analysis of **1** in dichloromethane revealed absorption exclusively in the violet-blue region (up to 410 nm), whereas the absorption extends up to 550 nm in the solid state, hence suggesting intermolecular interactions (Supplementary Information). Unambiguous structural proof was obtained by the crystallographic analysis of single crystals obtained by slow diffusion of pentane into a dichloromethane solution of **1** (Fig. 2a). The asymmetric unit in **1** contains two crystallographically independent molecules, with Au–N_α_ distances of 2.033(8) and 2.043(8) Å in a slightly distorted square planar geometry (C–Au–P 165.6(2)° and 166.3(2)°; N–Au–N_α_ = 169.3(4)° and 177.0(3)°).

Once the optimal crystallization conditions for **1** were identified, we set out to explore the in crystallo formation of the corresponding formal gold nitrido complex via dinitrogen photoextrusion^54^. An extensive evaluation of the experimental parameters allowed us to follow the reaction progress by sequential collection of X-ray diffraction data over time after irradiation with a 405-nm laser at 160 K in the synchrotron (Fig. 2a; see Supplementary Information section 5 for details on the experimental set-up). Reaction monitoring showed the depletion of electron density at the N_β_ and N_γ_ positions of the azide group, while the occupancy of N_α_ remained unchanged. The crystallographic data were refined with a partial occupation model of both azide precursor **1** and the photoproduct, indicating the partial extrusion of N_2_ and a 30% conversion to the sought-after aura-nitrene **2** after 2 min.

The refined structure reveals comparable Au1–N2 bonds in complex **2** (**1**: 2.033(8) and 2.043(8) Å, **2**: 1.978(18) and 2.004(18) Å), although a shortening nearing statistical significance is observed in complex **2**. This is accompanied by a reduction in the *trans* influence of the N atom of the (P^N^C) ligand, reflected in a shortening of the N1–Au1 bond lengths (**1**: 2.014(7), **2**: 1.95(2) Å) observed in one of the pairs. While the quality of the X-ray data did not allow us to freely refine the molecule so as to unambiguously distinguish between the N_α_ atom of the azide and the nitrene, the Au1–N2 distance in the optimized structure of **2** (1.927 Å; Fig. 2b) agrees well with the expected value for a single bond according to Pyykko’s covalent radii for gold and nitrogen (1.95 Å)^55^. In addition, the square-planar geometry in aura-nitrene **2** is maintained (C–Au–P 168.9(8)° and 166.5(7)°, N–Au–N 175.4(14)° and 174.7(12)°). Prolonged irradiation of the crystals led to sample degradation and loss of crystallinity, preventing higher conversion rates and a better accuracy in the determination of the metrical parameters (Supplementary Information section 5).

The formation of **2** was further corroborated by high-resolution mass spectrometry (HRMS). First, a ^15^N-isotopologue of **1** was synthesized by anionic ligand exchange reaction between (P^N^C)Au–Cl and ^15^N-terminally labelled sodium azide (Na^15^N^14^N^14^N). This reaction yielded **1-**^**15**^**N**, which contains a 50% statistical distribution of ^14/15^N between the α and γ positions of the azide moiety (see **1-**^**15**^**N**_**α**_ and **1-**^**15**^**N**_**γ**_ in Fig. 2c). Upon electrospray ionization (ESI) of complex **1-**^**15**^**N** and the release of dinitrogen (^14^N≡^14^N and ^15^N≡^14^N), tandem mass spectrometry (MS/MS) analysis of the sample allowed the successful detection of the peak corresponding to **2** (mass/charge ratio (*m*/*z*) = 559.15704; calculated (calcd.) 559.15721) together with a 1-Da-shifted peak, confirming the formation of ^15^N-labelled **2-**^**15**^**N** (*m*/*z* = 560.15531, calcd. 560.15424). The isotope distribution of both peaks aligned with the simulated mass spectra for **2** and **2-**^**15**^**N** (Fig. 2c).

The electronic structure of complex **2** was investigated by density functional theory calculations^56^, which favour a triplet over an open-shell singlet ground state (Δ*E*^s/t^ = +47 kJ mol^**−**1^). The calculated structural parameters for the triplet spin state (*d*_Au–Nα_, 1.927 Å) show better agreement with the experimental values than those computed for the singlet excited state (*d*_Au–Nα_, 1.903 Å). The spin density is almost exclusively ligand-centred (Fig. 2b; natural spin population Au: 0.02 a.u., N: 1.96 a.u.), and population analysis, as well as localized and canonical orbital analyses, identify four metal-centred 5*d* orbitals and a covalent Au–N single bond (Supplementary Figs. 65–72 and Supplementary Table 18; Wiberg Bond Index Au–N: 0.75). Whereas aura-nitrene **2** is therefore well understood as a formal gold(III) complex, we note that the Au–N *σ*-bond is covalent (Au:N = 0.30:0.70) and that the physical oxidation state^57–59^ of the gold ion is lower (Supplementary Tables 18 and 23 and Supplementary Figs. 65–69, 71 and 72). This type of umpolung of nucleophilic imides/nitrides to electrophilic nitrenes is referred to as an inverted ligand field^13,25,37,60^. We conclude overall that *d*-metal admixing of aura-nitrene **2** is much weaker than in Schneider’s platina-nitrene (Pt: 0.13 a.u.; N: 1.81 a.u.) and even smaller than for the corresponding 4*d* pallada-nitrene (Pd: 0.07 a.u.; N: 1.91 a.u.)^44,45^. Ab initio CASSCF(18,13) calculations further corroborate a bona fide metalla triplet nitrene with two unpaired electrons in the *p*(*x*) and *p*(*y*) orbitals, a covalent Au–nitrene *σ*-bond, and another lone pair in the nitrene’s *s*-orbital (Fig. 2d and Supplementary Figs. 67–70). NEVPT2/CASSCF calculations under the inclusion of spin–orbit effects suggest only moderate axial zero-field splitting (*D* = 16 cm^**−**1^) and rhombicity (*E*/*D* = 0.28), further highlighting the ligand-diradical character^45^.

### Activation of O_2_

**Fig. 3:**
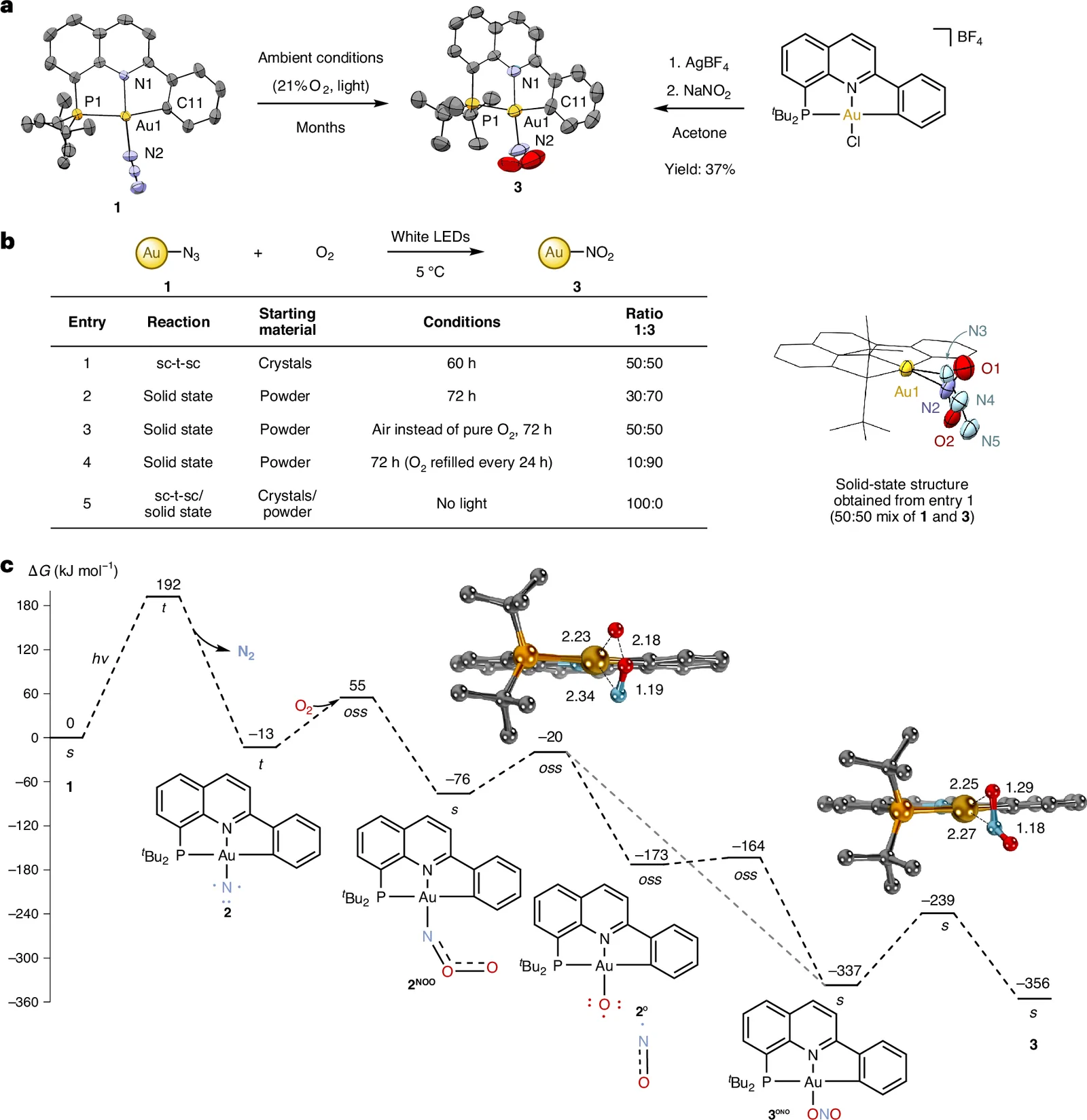
Nitrene-to-nitro oxidation by dioxygen. **a**, Left: Spontaneous sc-t-sc-transformation (left). Ellipsoidal representation of **1** (dichloromethane/hexane) and **3** with 50% probability ellipsoids. Counter-anions and hydrogen atoms are omitted for clarity. Selected distances (Å) in **1**: Au1–P1 2.3736(8), Au1–N1 2.010(3), Au1–C11 2.064(3), Au1–N2 2.017(3); in **3**: Au1–P1 2.388(2), Au1–N1 2.021(6), Au1–N2 2.010(9), Au1–C11 2.066(8). Selected angles (°) in **1**: C11–Au1–P1 166.48(11), N1–Au1–N2 174.84(13); in **3**: C11–Au1–P1 165.9(2), N2–Au1–N1 174.6(3). Right: Independent synthesis of the (P^N^C)Au–NO_2_ complex **3** (right). **b**, Dioxygen activation under controlled conditions in crystallo and solid-state ellipsoidal representation of a 50:50 mixture of **1** and **3** with 50% probability ellipsoids. The ellipsoidal representation is restricted to the Au atom and the N_3_ and NO_2_ moieties for clarity where the labels N_α_, N_β_ and N_γ_ correspond to N3, N4 and N5, respectively. Counter-anions and hydrogen atoms are omitted as well. Selected distances (Å): Au1–P1 2.373(2), Au1–N1 2.009(5), Au1–C11 2.087(7), Au1–N3 2.006(13), Au1–N2 2.080(18). Selected angles (°): C11–Au1–P1 166.5(2), N3–Au1–N1 170.5(6), N1–Au1–N2 169.5(7). **c**, Mechanism for the formation of nitro complex **3** as obtained at the ZORA-DLPNO-CCSD(T1)/def2-TZVPP//ZORA-PBE0-D4/def2-SVP level of theory. For comparison with ωB97X-V, the implicit consideration of the crystal’s polar lattice, as well as disfavoured pathways, see the Supplementary Information. *t*, triplet state; *s*, closed shell singlet state (in case spin-polarized); *oss*, open-shell (antiferromagnetic) singlet state.

Over the course of our studies, crystals of (P^N^C)Au–N_3_ complex **1** underwent a slight darkening when exposed to ambient conditions over extended periods of time (that is, months). X-ray diffraction analysis of such crystals (obtained by slow diffusion of hexane into a dichloromethane solution of **1**) revealed the unexpected formation of the (P^N^C)Au–NO_2_ complex **3** (Fig. 3a (left)). The conversion of **1** into **3** is accompanied by an expansion of the unit cell’s volume from 2,376.46(9) to 2,451.75(16) Å^3^ due to the extrusion of N_2_ and capture of O_2_. The structural parameters around the Au atom in complex **3** remain similar to those found for complex **1** (bond distances (Å) for **3** versus **1**: Au1–P1 2.388(2) versus 2.3736(8), Au1–N1 2.021(6) versus 2.010(3), Au1–C11 2.066(8) versus 2.064(3), Au1–N2 2.010(9) versus 2.017(3); angles (°) for **3** versus **1**: C11–Au1–P1 165.9(2) versus 166.48(11), N1–Au1–N2 174.6(3) versus 174.84(13)). The Au–N(O_2_) distance (2.010(9) Å) is in line with those reported for the only two examples featuring gold-nitro structures (2.142(11) Å and 2.03(3) Å) available in the literature^61,62^.

Additional characterization of complex **3** was carried out including HRMS (*m*/*z*: 591.14735; calcd. 591.14702) and by ^1^H, ^13^C and ^31^P nuclear magnetic resonance (NMR) spectroscopies. Furthermore, the IR spectrum showed a band at 1,327 cm^**−**1^ characteristic for the *ν*_s_(NO_2_) stretch. To unambiguously confirm the structural assignment, (P^N^C)Au–NO_2_ **3** was independently prepared by reaction of (P^N^C)Au–Cl with 1 equiv. of AgBF_4_, followed by treatment with NaNO_2_ in acetone (Fig. 3a (right)). The spectroscopic data (NMR, HRMS and IR) of this material perfectly matched with those obtained by spontaneous in crystallo oxidation of the gold azide complex **1**.

We hypothesized that the visible light in our laboratory might have activated the azide complex **1**, leading to the intermediate formation of aura-nitrene **2** in crystallo, which reacted with ambient O_2_ to produce the nitro complex **3** in a rare example of a spontaneous sc-t-sc transformation^63^. We thus sought to replicate this spontaneous sc-t-sc reaction under controlled conditions. Monocrystals of gold-azide **1** (obtained from a dichloromethane/cyclopentane mixture) were illuminated under neat O_2_ atmosphere (99.999%) at 1 atm. Photoirradiation with white light-emitting diodes (60 W, 4,000 K) at 5 °C for 60 h resulted in 50% conversion to **3**. The crystallographic data were refined with a partial occupation model of both azide **1** and nitro complex **3** in a 50:50 ratio (Fig. 3b, entry 1). As in the case of the spontaneous sc-t-sc transformation, both complexes display a very similar (P^N^C)Au framework, with a large degree of overlap both in terms of bond lengths and angles around the metal centre (selected distances (Å): Au1–P1 2.373(2), Au1–N1 2.009(5), Au1–C11 2.087(7), Au1–N3 2.006(13), Au1–N2 2.080(18); selected angles (°): C11–Au1–P1 166.5(2), N3–Au1–N1 170.5(6), N1–Au1–N2 169.5(7). It is important to note that the activation of O_2_ is not affected by different crystal polymorphs used as starting material (Supplementary Information section 8). As it is typical for sc-t-sc reactions, longer exposures to light and/or oxygen compromised the crystallinity of the samples and did not result in an improved outcome. Besides limitations associated with maintaining crystal integrity, in crystallo reactivity requires exquisite control over reaction conditions and advanced experimental set-ups beyond single-crystal X-ray diffraction, thus limiting its scalability and broad applicability^54^. To overcome these challenges, we set out to investigate reactions in the solid state using bulk powder^64,65^. Complex **1** was pulverized and placed in a 4-mL vial under an O_2_ atmosphere (99.999%) at 1 atm, followed by irradiation with a white light-emitting diode at 5 °C. The formation of the nitro complex **3** was monitored by solid IR spectroscopy revealing a 70% conversion of the azido precursor to **3** after 72 h (Fig. 3b, entry 2). This ratio was confirmed upon X-ray diffraction analysis of monocrystals obtained after recrystallization of the powder sample. Interestingly, 50% conversion was achieved by illuminating **1** under regular air (21% O_2_), reflecting a transformation akin to that occurring originally in crystallo (Fig. 3b, entry 3). Finally, sequential refill of the vial with fresh O_2_ over a period of 3 days resulted in 90% conversion of complex **3** (Fig. 3b, entry 4). Control experiments in the absence of light confirmed that the reaction, both in crystallo and in solido, requires irradiation for the key aura-nitrene to be formed (Fig. 3b, entry 5). Attempts to replicate this transformation in solution phase did not result in the formation of **3** but rather in decomposition of the starting material (Supplementary Information section 6). A careful analysis of the literature shows that, while arylnitrenes can easily undergo photooxidation with molecular oxygen using matrix isolation techniques^66–68^, the oxidation of metal nitrides to yield the corresponding metal–nitro complexes is not well documented. This prompted us to in silico study the mechanism of this intriguing transformation (Fig. 3c). Whereas a transition state for the substitution of N_2_ by O_2_ could not be located, the end-on addition of dioxygen to the nitrene **2** (Δ*G* = −13 kJ mol^**−**1^) affords the nitroso oxide **2**^**NOO**^ with a singlet ground state (Δ*G*^‡^ = +55 kJ mol^**−**1^; Δ*G* = −76 kJ mol^**−**1^)^69,70^. Rearrangement via an unusual four-membered open-shell singlet (*oss*) transition state (Δ*G*^‡^ = −20 kJ mol^**−**1^) affords the closed-shell *κ*-O coordinate nitrito complex **3**^**ONO**^ (Δ*G* = − 337 kJ mol^**−**1^). This concerted NO flip proceeds on a flat potential energy surface. Hence, the corresponding stepwise (Δ*G*^‡^ = −164 kJ mol^**−**1^) and formally bifurcating pathway^71^ involving an exciting gold terminal oxo-intermediate (Δ*G* = −173 kJ mol^**−**1^) was found a viable alternative mechanism. Interestingly, terminal gold oxo complexes have been intensively sought after given their potential relevance in heterogeneous oxidation processes, but their existence, and even more so their characterization, remains to be experimentally validated^14,72–74^. Due to the soft nature of gold, the *κ*-O nitrito complex **3**^**ONO**^ isomerizes eventually to the *κ*-N nitro congener **3** (Δ*G* = −356 kJ mol^**−**1^; Δ*G*^‡^ = −239 kJ mol^**−**1^). Alternative mechanisms involving other spin states as well as the side-on addition of dioxygen to generate a transient dioxaziridine are deemed unlikely (Supplementary Figs. 79–83).

### Solid-state reactivity

**Fig. 4:**
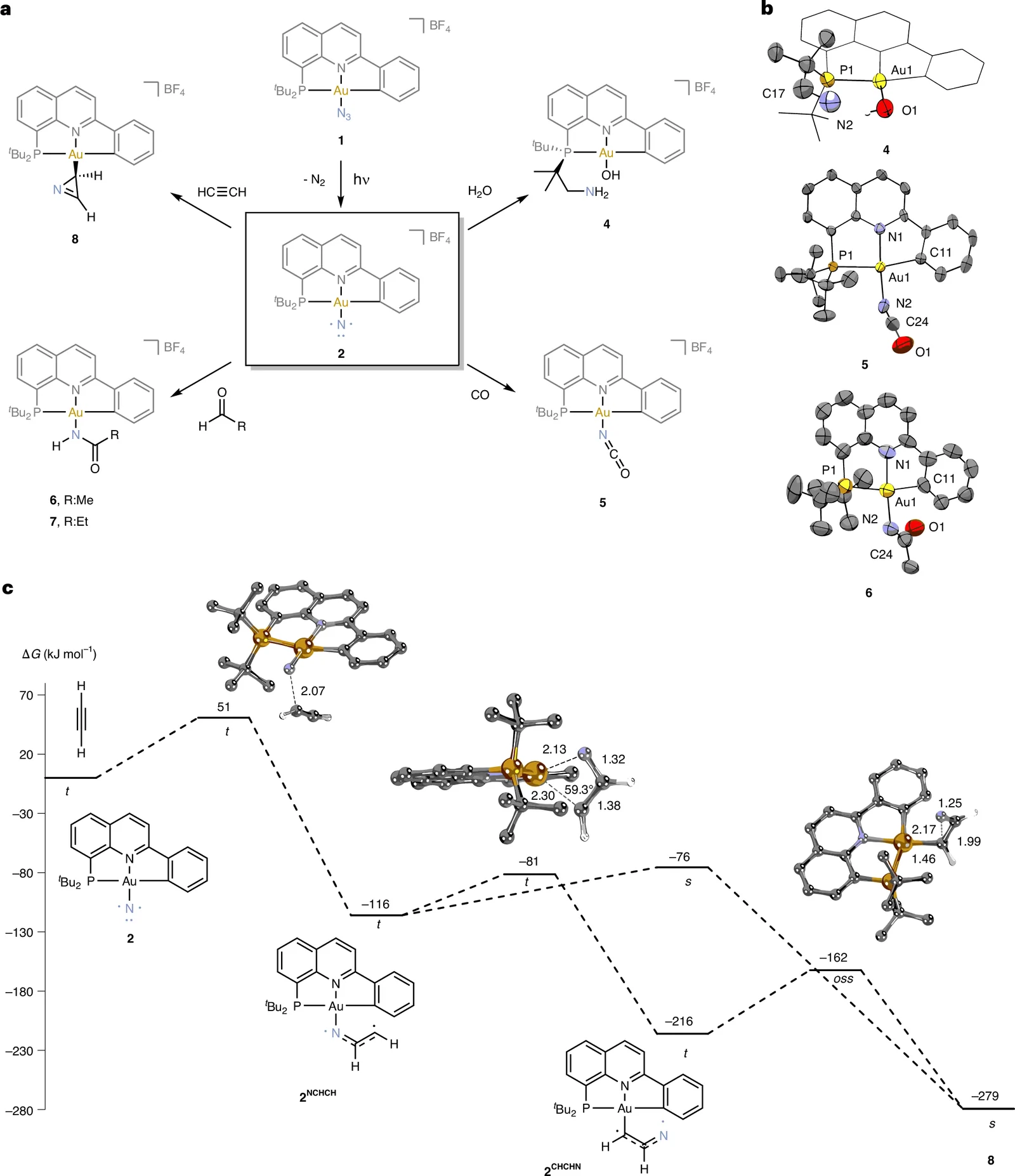
Solid-state reactivity of aura-nitrene 2. **a**, Solid-state reactivity of the photogenerated aura-nitrene **2**. **b**, Ellipsoidal representation of **4**, and one of the two molecules in the asymmetric unit of **5** and **6**, with 50% probability ellipsoids. Counter-anions, solvent molecules and hydrogen atoms (except H1 in **4**) are omitted for clarity. Only one molecule of **5** and **6** is described here for clarity (see Supplementary Figs. 67 and 68 and Supplementary Tables 16 and 17 for more details). Selected distances (Å) in **4**: Au1–P1 2.3705(16), Au1–N1 2.014(5), Au1–C11 2.054(6), Au1–O1 1.979(5), C17–N2 1.427(15); in **5**: Au1–P1 2.3909(19), Au1–N1 1.988(7), Au1–C11 2.064(8), Au1–N2 2.028(9), N2–C24 1.053(17), C24–O1 1.221(19); in **6**: Au1–P1 2.376(4), Au1–N1 2.031(14), Au1–C11 2.080(16), Au1–N2 2.031(14). Selected angles (°) in **4**: C11–Au1–P1 167.1(2), N1–Au1–O1 172.9(2); in **5**: C11–Au1–P1 166.7(2), N1–Au1–N2 175.9(3), Au1–N2–C24 134.3(12); in **6**: C11–Au1–P1 167.2(5), N1–Au1–N2 174.1(6), C24–N2–Au1 118.8(14). **c**, Mechanism for the formation of metallated azirine **8** as obtained at the ZORA-DLPNO-CCSD(T1)/def2-TZVPP//ZORA-PBE0-D4/def2-SVP level of theory. For comparison with ωB97X-V, the implicit consideration of the crystal’s polar lattice, as well as disfavoured pathways, see the Supplementary Information.

Inspired by the successful in solido generation of aura-nitrene **2**, its reactivity with various gases was explored next. Irradiation of **1** in powder form without rigorous exclusion of moisture, that is, non-dry N_2_, yielded compound **4** (Fig. 4a, top right). X-ray diffraction analysis of suitable crystals obtained by vapour diffusion of cyclopentane into a dichloromethane solution^75^ of the complex revealed the formation of a gold-hydroxo species with concomitant amination of the ^*t*^Bu_2_P moiety (Fig. 4b, top). This fragment displays a C17–N2 bond length of 1.427(15) Å and an NH_2_–OH distance of 2.79(1) Å, which suggests the presence of a molecular interaction between these two groups. In addition, complex **4** exhibits a short Au1–O1 distance of 1.979(5) Å, consistent with the weak *trans* influence of the quinoline nitrogen atom, and closely resembling previously reported (P^N^C)Au–OH, where the ^*t*^Bu groups remained unactivated^53^. The formation of **4** can be rationalized on the basis of an intramolecular C*sp*^3^–H bond insertion of a ^*t*^Bu group into transient **2**, followed by hydrolysis of the resulting amido complex (see additional discussion in Supplementary Information section 7).

The photoirradiation of **1** under CO atmosphere afforded the corresponding isocyanate complex **5** in quantitative yield (Fig. 4a, bottom right). Recrystallization by vapour diffusion of cyclopentane into a dichloromethane solution yielded crystals suitable for X-ray diffraction analysis, confirming the structure (Fig. 4b, middle). Complex **5** was further characterized by HRMS (*m*/*z*: 587.15168; calcd. 587.15210) and ^1^H, ^13^C and ^31^P NMR spectroscopies. Furthermore, the IR spectrum of **5** showed a band at 2,206 cm^**−**1^ characteristic of the *ν*(NCO) stretch. The reaction was also carried out in the presence of ^13^C-labelled carbon monoxide, resulting in the formation of isotopologue **5-**^**13**^**C** (*m*/*z*: 588.15536; calcd. 588.15546), which displays the IR *ν*(NCO) stretch shifted to 2,148 cm^**−**1^.

Insertion of gold metallonitrene **2** into the C*sp*^2^–H bond of aldehydes was also investigated. To carry out the reaction under similar solid-gas phase conditions, the experimental set-up was modified so that an inner vial containing a powder sample of gold-azide **1** was placed inside an outer vial filled with the reagent of interest. Irradiation of **1** in the presence of acetaldehyde and propionaldehyde delivered the corresponding gold–amidate complexes **6** and **7**, respectively (Fig. 4a, bottom left). Their independent synthesis and X-ray diffraction analysis of single crystals of **6** (obtained by diffusion of pentane into an acetonitrile solution) confirmed the intermolecular C–H bond insertion into the nitrene moiety (Fig. 4b, bottom). Complex **6** exhibits a Au1–N2 amido ligand bond length of 2.031(14) Å, closely matching the only previously reported gold–amidate complex (Au–N 2.015(10) Å)^76^, and a C24–N2–Au1 angle of 118.8(14)°, consistent with the *sp*^2^ hybridization of the N atom.

The reactivity of **2** was further investigated in the presence of C–C unsaturated moieties. While no reaction with ethylene could be detected, the photogenerated aura-nitrene **2** efficiently reacted with acetylene, leading to the clean formation of a new gold species **8** (Fig. 4a, top left). The ^1^H NMR spectra exhibited two new distinctive signals at 10.40 ppm (dd, ^3^*J*_HH_ = 1.8, ^3^*J*_HP_ = 1.8) and 3.38 ppm (dd, ^3^*J*_HH_ = 1.8, ^3^*J*_HP_ = 1.8), which correlate to two signals in the ^13^C NMR spectrum at 172.3 ppm and 33.1 ppm, respectively. In addition, the ^*t*^Bu groups of the ligand became inequivalent, suggesting that the final product does not contain a symmetrical *κ-N-*gold coordinated azirine stemming from a typical [1 + 2] cycloaddition. Although single crystals of this complex could not be obtained due to its low stability in solution, all spectroscopic evidence (^1^H, ^13^C, ^1^H–^1^H correlation spectroscopy, ^1^H–^1^H nuclear Overhauser effect spectroscopy, ^1^H–^13^C heteronuclear multiple bond correlation, ^1^H–^13^C heteronuclear single quantum coherence and ^31^P NMR, HRMS, and IR.) confirm the formation of a *κ-C-*(2*H*-azirin-2-yl)gold complex **8**. Quantum chemical calculations (Fig. 4c) suggest a mechanism via initial triplet nitrene addition to acetylene (Δ*G*^‡^ = +51 kJ mol^**−**1^), thereby generating the triplet-diradical **2**^**NCHCH**^ (Δ*G* = −116 kJ mol^**−**1^). The cyclization to the closed-shell metallated azirine **8** probably proceeds stepwise via *κ*-*N* **2**^**NCHCH**^ to *κ*-*C* **2**^**CHCHN**^ isomerization (Δ*G*^‡^ = −81 kJ mol^**−**1^; Δ*G* = −216 kJ mol^**−**1^), followed by intersystem crossing to the singlet spin state and radical recombination (Δ*G*^‡^ = −162 kJ mol^**−**1^). However, the concerted singlet pathway was predicted to be only marginally higher in energy (Δ*G*^‡^ = −76 kJ mol^**−**1^).

The diverse reactivity portfolio summarized in Fig. 4 as well as the activation of O_2_ discussed in Fig. 3 can be rationalized on the basis of an electrophilic/radical character of aura-nitrene **2**, even if an ambiphilic character cannot be ruled out in its reaction with aldehydes as postulated by Schneider et al. for the (PNP)Pt and Pd congeners^44,45^.

## Conclusions

We report a bona fide aura-nitrene and provide a detailed analysis of its electronic structure and bonding. The species features a nitrogen-centred triplet diradical with a single Au–N *σ*-bond. The highly electronegative, cationic gold(III) centre enhances the diradical character of the nitrene, resulting in spin densities that exceed those reported for palladium and platinum analogues. In contrast to early transition-metal nitrido complexes featuring strong M≡N triple bonds, the aura-nitrene displays a weak Au–N bond, owing to the absence of stabilizing *π*-type interactions.

We demonstrate that, upon light irradiation of the corresponding azide precursors, aura-nitrene **2** can be generated in crystallo via N_2_ extrusion. Furthermore, we characterize a nitrene-to-nitro transformation driven by an unusual O_2_ activation by crystallographic snapshots. In addition, we show that the photogeneration of aura-nitrene **2** can be efficiently replicated in the solid state, thus enabling a diverse reactivity portfolio that includes the intra- and intermolecular activation of C–H bonds, the fixation of CO and an intriguing alkyne addition/sigmatropic rearrangement, as well as the aforementioned N-to-NO_2_ reaction.

These transformations highlight the electrophilic and diradical nature of the aura-nitrene while demonstrating gold’s potential in NAT chemistry beyond conventional pathways. By characterizing a gold metallonitrene—alongside the Liu group’s recent discovery of a gold metallocarbene^77^—we establish a direct link between gold carbenes and late transition-metal nitrides. Our findings emphasize the pivotal role of gold(III)-stabilized frameworks in unlocking new reactivity paradigms, expanding the frontiers of C–N bond formation.

## Online content

Any methods, additional references, Nature Portfolio reporting summaries, source data, extended data, supplementary information, acknowledgements, peer review information; details of author contributions and competing interests; and statements of data and code availability are available at https://doi.org/10.1038/s41557-026-02152-3.

## Supplementary information

Supplementary Information Supplementary Figs. 1–94, Tables 1–29, Scheme 1 and Discussion.

Supplementary Data 1 Coordinates for structures calculated by density functional theory.

## Data Availability

All data supporting the findings of this study are available within the Article and its Supplementary Information. Crystallographic data for the structures reported in this Article have been deposited at the Cambridge Crystallographic Data Centre, under deposition numbers CCDC 2449336 (**1**, dichloromethane/pentane), 2449337 (**2**), 2449338 (**1**, dichloromethane/hexane), 2449339 (**3**), 2449341 (**1**, dichloromethane/cyclopentane), 2449342 (mixture of **1** and **3** (70:30)), 2449343 (mixture of **1** and **3** (50:50)), 2449344 (mixture of **1** and **3** (25:75)), 2449345 (**4**), 2449346 (**5**) and 2449347 (**6**). See Supplementary Information section 8 for more details on how each of these crystals was obtained. Copies of the data can be obtained free of charge at https://www.ccdc.cam.ac.uk/structures.
