# Supplementary material for: A photogenerated triplet aura-nitrene for gold-mediated nitrene transfer
Source: Nat Chem. 2026 Jul 21;18(8):1413–21. doi: 10.1038/s41557-026-02152-3 (PMC13423833; doi:10.1038/s41557-026-02152-3)
Supplement: Supplementary file 1 — Supplementary Figs. 1–94, Tables 1–29, Scheme 1 and Discussion. [file 41557_2026_2152_MOESM1_ESM.pdf]

nature chemistry

Supplementary information https://doi.org/10.1038/s41557-026-02152-3

A photogenerated triplet aura-nitrene for gold-mediated nitrene transfer

In the format provided by the authors and unedited

Contents	Page
1. General information and experimental methods	S2
2. Synthesis and characterization	S4
3. Spectroscopic characterization	S12
4. Mass spectrometric analysis	S36
5. Photocrystallographic characterization	S37
6. *In crystallo* activation of O_2	S39
7. Solid-state reactions	S41
8. X-ray diffraction analyses	S46
9. Density functional and *ab-initio* calculations	S59
10. References	S112

1 General information and experimental methods

General Information. Unless otherwise stated, reactions were performed under nitrogen (99.999%) or argon (99.999%) atmosphere using Schlenk techniques, gloveboxes, and dry solvents. Commercial chemicals were used as received. Sodium azide-1-^{15}N (98% ^{15}N) was purchased from *Merck*. Molecular oxygen (99.999%) and acetylene (99.5%) were purchased from *PanGas*, carbon monoxide (99.997%) was purchased from *Linde*, and ^{13}C-labelled carbon monoxide (99.9%, isotopic content 99.3%) was purchased from *Eurisotop*. NMR (^{1}H, ^{13}C, ^{15}N, ^{31}P and 2D experiments) were recorded on either AV2 400, AV2 500 or Avance Neo 500 MHz *Bruker* spectrometers. Chemical shifts are given in ppm. ^{1}H and ^{13}C NMR spectra are referenced to residual solvent peaks.[1] ^{15}N and ^{31}P NMR spectra are referenced to NH_3 and 85% H_3PO_4, respectively. Multiplicities are abbreviated: singlet (s), doublet (d), triplet (t), quartet (q), septuplet (sept), multiplet (m), and broad (br). Couplings constants *J* are given in Hz.

MS. High resolution electrospray ionization mass spectrometry (HR-ESI-MS) was measured in *QExactive* or *timsTOF* instruments. *Dionex Ultimate 3000* UHPLC system (*ThermoFischer Scientifics*, Germering, Germany) connected to a *QExactive* MS with a heated ESI source (*ThermoFisher Scientific*, Bremen, Germany); onflow injection of 1 μL sample (*c* = ca. 50 μg mL^{-1} in the indicated solvent) with an *XRS* auto-sampler (*CTC*, Zwingen, Switzerland); flow rate 120 μL min^{-1}; ESI: spray voltage 3.0 kV, capillary temperature 280 °C, sheath gas 30 L min^{-1}, aux gas 8 L min^{-1}, s-lens RF level 55.0, aux gas temperature 250 °C (N_2); full scan MS in the alternating (+)/(−)-ESI mode; mass ranges 80–1’200 *m/z*, 133–2’000 *m/z*, or 200–3’000 *m/z* at 70’000 resolution (full width half-maximum); automatic gain control (AGC) target of $3.00 \cdot 10^{6}$; maximum allowed ion transfer time (IT) 30 ms; mass calibration to <2 ppm accuracy with *Pierce*® ESI calibration solutions. (*ThermoFisher Scientific*, Rockford, USA); lock masses: ubiquitous erucamide (*m/z* 338.34174, (+)-ESI) and palmitic acid (*m/z* 255.23295, (−)-ESI). *TimsTOF Pro* TIMS-QTOF-MS instrument (*Bruker Daltonics* GmbH, Bremen, Germany). The samples were dissolved in (e.g. MeOH) at a concentration of ca. 50 µg ml^{-1} and analyzed via continuous flow injection (2 µL min^{-1}). The mass spectrometer was operated in the positive (or negative) electrospray ionization mode at 4’000 V (-4’000 V) capillary voltage and −500 V (500 V) endplate offset with a N_2 nebulizer pressure of 0.4 bar and a dry gas flow of 4 l min^{-1} at 180 °C. Mass spectra were acquired in a mass range from *m/z* 50 to 2’000 at ca. 20’000 resolution (*m/z* 622) and at 1.0 Hz rate. The mass analyzer was calibrated between *m/z* 118 and 2’721 using an *Agilent* ESI-L low concentration tuning mix solution (*Agilent*, USA) at a resolution of 20’000 giving a mass accuracy below 2 ppm. All solvents used were purchased of the best LC-MS quality.

IR. Infrared spectra were recorded on a *JASCO* FT/IR-4100 spectrometer at room temperature.

UV-Vis. UV-Vis spectra in solution were recorded on a Lambda 465 *Perkin Elmer* UV/Visible spectrometer. Solid-state UV-Vis spectra were recorded on a Lambda 1050+ *Perkin Elmer* UV/visible/NIR spectrometer with a 150 mm integrating sphere, using *Hëllma Analytics* High Precision Cell made of high-performance quartz glass, with light path 0.1 mm. Diffuse absorbance was collected to ensure better quality data.

EPR. EPR data collected by continuous wave electron paramagnetic resonance (CW-EPR) spectroscopy experiments at 10 K on a Bruker Elexsys E500 spectrometer operating at X-band frequencies, using an ER4102ST microwave resonator and equipped with an Oxford helium (ESR900) cryostat. All CW-EPR spectra were acquired at room temperature with the following spectrometer parameters: microwave frequency = 9.4 GHz, sweep width = 250 mT and 895 mT, center field = 328.2 mT and 450 mT, respectively, modulation frequency = 100 kHz, modulation amplitude = 3 G, microwave power = 2.007 mW, power attenuation = 20 dB, conversion time = 82 ms, time constant = 40.96 ms. All measured g-factors were offset-corrected against a known standard (i.e., free radical 1,1-diphenyl-2-picrylhydrazyl).

X-ray. Single-crystal X-ray diffraction data were collected at 160(1) K on a *Rigaku OD Synergy-Hypix* or -*Pilatus*, or on a *Rigaku OD SuperNova/Atlas* area-detector using molybdenum (λ = 0.71073 Å) or copper (λ = 1.54184 Å) X-ray radiation and an *Oxford Instruments Cryojet XL cooler*. The *in crystallo* experiments to characterize the structure of the gold-nitrene were carried out using synchrotron radiation (λ = 0.65255 Å) at the Swiss Light Source located at the Paul Scherrer Institute (PSI).

Safety Statement. All compounds containing azides are potentially explosive. Proper safety precautions must be taken during storage and usage.

2 Synthesis and characterization

2.1 Synthetic procedures

(P^N^C)Au-Cl.[2] 8-(di-*tert*-buthylphosphino)-2-arylquinoline (370 mg, 1.06 mmol) and chloro(dimethyl sulfide)gold(I) (310 mg, 1.06 mmol) were placed in a Schlenk flask under N_2 atmosphere and 10 mL of dichloromethane were added. The mixture was stirred at 25 °C for 1 h. After completion of the reaction, the solvent was concentrated under reduced pressure, and pentane was added to afford an off-white solid, which was washed with pentane and dried under vacuum. When the off-white solid was dried, Selectfluor (450 mg, 1.27 mmol) was added into the Schlenk flask followed by 8 mL of acetonitrile, and the resulting mixture was stirred at 25 °C for 1 h. After completion of the reaction, the solvent was concentrated under reduced pressure and subsequently diluted with dichloromethane. The resulting solution was washed with deionized water, dried over anhydrous $MgSO_4$, filtered, and concentrated under reduced pressure. The resulting crude was treated with cold diethyl ether to afford the corresponding (P^N^C)gold(III)-chloride complex as yellow solid, which was washed with cold diethyl ether and cold pentane and subsequently dried under vacuum. Yield: 430 mg (61%).

(P^N^C)Au-OH.[2] The (P^N^C)Au-Cl (80 mg, 0.120 mmol) and Ag_2O (280 mg, 1.2 mmol) were placed in a vial and reagent grade (non-dry) dichloromethane (4 mL) with a drop of deionized water was added. The resulting mixture was stirred at 25 °C for 15 h protected from light. After completion of the reaction, the mixture was filtered through Celite and concentrated under reduced pressure. The resulting crude was treated with cold diethyl ether to afford the corresponding (P^N^C)gold(III)-hydroxide complex as a pale yellow solid, which was washed with cold diethyl ether and cold pentane, and subsequently dried under vacuum. Note: several reactions of this type (up to six) can be run in parallel, with the crude products combined during work-up for isolation. This approach often yields better results than simply scaling up the reaction. Yield: 71 mg (91%).

(P^N^C)Au-N_3 (1). Trimethylsilylazide (0.1 mL, 0.77 mmol) was added to a solution of (P^N^C)gold(III)-hydroxide (100 mg, 0.154 mmol) in dichloromethane (1.5 mL) and stirred at room temperature for 1 h. The solution was concentrated under reduced pressure and the resulting crude was treated with diethyl ether to afford complex **1** as a pale-yellow solid, which was washed with diethyl ether and pentane, and subsequently dried under vacuum. Yield: 93 mg (89%). Suitable crystals for X-ray diffraction analysis were obtained by slow vapor diffusion of pentane, hexane or cyclopentane into a dichloromethane solution of **1**.

(P^N^C)Au-$^{15}N_3$ (1-^{15}N). NaN_3 (1-^{15}N) (5 mg, 0.08 mmol) was added to a solution of (P^N^C)gold(III)-chloride (34 mg, 0.05 mmol) in dichloromethane (1.5 mL) and stirred at room temperature for 3 h. Then, the solution was filtered through celite, concentrated under reduced pressure and the resulting crude was treated with diethyl ether to afford the complex **1-^{15}N** as a pale-yellow solid, which was washed with diethyl ether and pentane, and subsequently dried under vacuum. Yield: 24 mg (70%).

(P^N^C)Au-NO_2 (3). Complex **3** was synthesized *via* a solid-state reaction, following the general procedure described in section 7. Independent synthesis. Adapted from a literature procedure.[3] A solution of (P^N^C)gold(III)-chloride (60 mg, 0.09 mmol) in dichloromethane (2 mL) was treated with $AgBF_4$ (17 mg, 0.009 mmol). After stirring for 30 min, the mixture was filtered through celite. Next, $NaNO_2$ (60 mg, 0.9 mmol) was added, and the resulting mixture was stirred for 2 h. The reaction was filtered through celite and concentrated under reduced pressure. The resulting crude was treated with diethyl ether to afford compound **3** as an off-white solid, which was washed with diethyl ether and pentane, and subsequently dried under vacuum. Yield: 22 mg (37%).

(P^{NH_2}^N^C)Au-OH (4). Complex **4** was synthesized *via* a solid-state reaction, following the general procedure described in section 7. Suitable crystals for X-ray diffraction analysis were obtained by slow vapor diffusion of cyclopentane into a dichloromethane solution of a mixture of compounds **1**, **3** and **4**.

(P^N^C)Au-NCO (5). Complex **5** and **5-^{13}C** were synthesized *via* a solid-state reaction, following the general procedure described in section 7. Suitable crystals for X-ray diffraction analysis were obtained by slow vapor diffusion of cyclopentane into a dichloromethane solution of **5**.

(P^N^C)Au-NHC(O)CH_3 (6). Complex **6** was synthesized *via* a solid-state reaction, following the general procedure described in section 7. Independent Synthesis. A solution of (P^N^C)gold(III)-hydroxide (20 mg, 0.031 mmol) in dichloromethane (2 mL) was treated with an excess of acetamide (0.31 mmol) and triethylamine (0.31 mmol) and stirred at room temperature for 16 h. The solution was filtered through celite and concentrated under reduced pressure. The resulting crude was treated with diethyl ether to afford compound **6** as a pale-yellow solid, which was washed with diethyl ether and pentane, and subsequently dried under vacuum. Yield: 13 mg (62%). Suitable crystals for X-ray diffraction analysis were obtained by slow vapor diffusion of pentane into an acetonitrile solution of **6**.

(P^N^C)Au-NHC(O)CH_2CH_3 (7). Complex **7** was synthesized *via* a solid-state reaction, following the general procedure described in section 7. Independent Synthesis. A

solution of (P^N^C)gold(III)-hydroxide (20 mg, 0.031 mmol) in dichloromethane (2 mL) was treated with an excess of propionamide (0.31 mmol) and triethylamine (0.31 mmol) and stirred at room temperature for 16 h. Then, the solution was filtered through celite and concentrated under reduced pressure. The resulting crude was treated with diethyl ether to afford compound **7** as a pale red solid, which was washed with diethyl ether and pentane, and subsequently dried under vacuum. Yield: 13 mg (61%).

2.2 Characterization of the compounds

(P^N^C)Au-Cl[2]

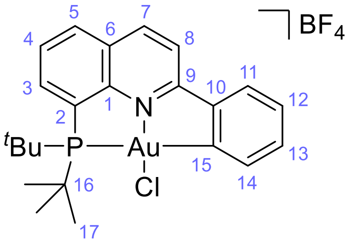

^{1}H NMR (400.13 MHz, CD_2Cl_2, 298 K): δ 9.05 (dd, $^3J_{HH}$ = 8.8, $^5J_{HP}$ = 1.4, 1H, H^7), 8.52-8.45 (m, 2H, H^3 and H^5), 8.40 (d, $^3J_{HH}$ = 8.8, 1H, H^8), 8.10 (ddd, $^3J_{HH}$ = 7.8, $^3J_{HH}$ = 7.7, $^4J_{HP}$ = 1.6, 1H, H^4), 8.04-7.99 (m, 2H, H^{11} and H^{14}), 7.67 (dddd, $^3J_{HH}$ = 7.8, $^3J_{HH}$ = 7.5, $^5J_{HP}$ = 3.8, $^4J_{HH}$ = 1.5, 1H, H^{13}), 7.59 (dd, $^3J_{HH}$ = 7.5, $^3J_{HH}$ = 7.5, 1H, H^{12}), 1.59 (d, $^3J_{HP}$ = 16.9, 18H, H^{17}). ^{13}C{^{1}H} NMR (125.81 MHz, CD_2Cl_2, 298 K): δ 169.4 (d, $^2J_{CP}$ = 122.7, C^{15}), 169.2 (d, $^3J_{CP}$ = 6.0, C^9), 151.3 (d, $^2J_{CP}$ = 12.0, C^1), 146.8 (s, C^{10}), 146.4 (s, C^7), 142.5 (d, $^2J_{CP}$ = 1.1, C^3), 135.4 (d, $^4J_{CP}$ = 2.0, C^5), 134.8 (d, $^4J_{CP}$ = 9.2, C^{13}), 130.8 (s, C^{14}), 130.8 (d, $^3J_{CP}$ = 7.1, C^6), 130.6 (d, $^3J_{CP}$ = 6.5, C^4), 130.5 (s, C^{12}), 129.4 (d, $^4J_{CP}$ = 5.9, C^{11}), 125.6 (d, $^1J_{CP}$ = 37.1, C^2), 120.8 (s, C^8), 40.3 (d, $^1J_{CP}$ = 14.5, C^{16}), 29.9 (d, $^2J_{CP}$ = 3.3, C^{17}). ^{19}F{^{1}H} NMR (470.71 MHz, CD_2Cl_2, 298 K): δ -152.7 (br, B*F*$_4$). ^{31}P{^{1}H} NMR (202.52 MHz, CD_2Cl_2, 298 K): δ 80.0 (s, *P*). (+)-HR-ESI-MS (electrospray, m/z): calcd for $C_{23}H_{27}NAuClP$ [M]$^+$, 580.12297; found, 580.12407.

(P^N^C)Au-OH[2]

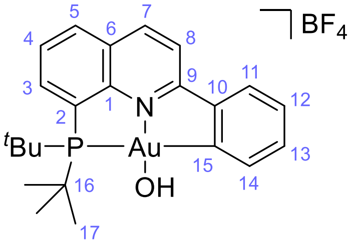

^{1}H NMR (500.30 MHz, CD_2Cl_2, 298 K): δ 8.98 (dd, $^3J_{HH}$ = 8.8, $^5J_{HP}$ = 1.3, 1H, H^7), 8.45-8.41 (m, 2H, H^3 and H^5), 8.36 (d, $^3J_{HH}$ = 8.8, 1H, H^8), 8.08-8.03 (m, 2H, H^4 and H^{11}), 7.71-7.66 (m, 1H, H^{13}), 7.63-7.58 (m, 2H, H^{12} and H^{14}), 1.75 (d, $^3J_{HP}$ = 2.8, 1H, O*H*), 1.59 (d, $^3J_{HP}$ = 16.7, 18H, H^{17}). ^{13}C{^{1}H} NMR (125.82 MHz, CD_2Cl_2, 298 K): δ 168.1 (d, $^3J_{CP}$ = 5.8, C^9), 165.7 (d, $^2J_{CP}$ = 123.0, C^{15}), 151.4 (d, $^2J_{CP}$ = 11.9, C^1), 146.7 (s, C^{10}), 145.3 (s, C^7), 141.7 (s, C^3), 134.8 (d, $^4J_{CP}$ = 2.0, C^5), 133.8 (d, $^4J_{CP}$ = 9.0, C^{13}), 130.5 (d, $^3J_{CP}$ = 7.5, C^6), 130.4 (d, $^3J_{CP}$ = 6.6, C^4), 130.2 (s, C^{12}), 129.1 (d, $^4J_{CP}$ = 5.3, C^{11}), 128.4 (d, $^3J_{CP}$ = 1.5, C^{14}), 126.0 (d, $^1J_{CP}$ = 37.4, C^2), 120.4 (s, C^8), 39.4 (d, $^1J_{CP}$ = 15.1, C^{16}), 29.6 (d, $^2J_{CP}$ =

3.7, C^{17}). $^{19}F\{^1H\}$ NMR (376.50 MHz, CD_2Cl_2, 298 K): δ -152.8 (br, BF_4). $^{31}P\{^1H\}$ NMR (202.52 MHz, CD_2Cl_2, 298 K): δ 72.2 (s, *P*). (+)-HR-ESI-MS (electrospray, m/z): calcd for $C_{23}H_{28}ONAuP$ $[M]^+$, 562.15685; found, 562.15703. IR (ATR, cm^{-1}): *v* (OH) 3559 (br).

(P^N^C)Au-N_3 (1)

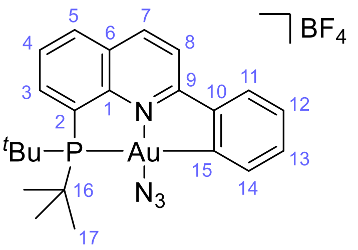

^1H NMR (400.13 MHz, CD_2Cl_2, 298 K): δ 9.02 (dd, $^3J_{HH}$ = 8.9, $^5J_{HP}$ = 1.1, H^7), 8.47 (d, $^3J_{HH}$ = 8.3, H^5), 8.44 (dd, $^3J_{HH}$ = 8.2, $^3J_{HP}$ = 7.7, H^3), 8.38 (d, $^3J_{HH}$ = 8.8, H^8), 8.11-8.06 (m, H^4 and H^{11}), 7.94 (dd, $^3J_{HH}$ = 9.5, $^4J_{HP}$ = 7.7, H^{14}), 7.74 (ddd, $^3J_{HH}$ = 7.9, $^3J_{HH}$ = 7.5, $^5J_{HP}$ = 3.3, H^{13}), 7.65 (dd, $^3J_{HH}$ = 7.7, $^3J_{HH}$ = 7.6, H^{12}), 1.58 (d, $^3J_{HP}$ = 16.8, 18H, H^{17}). $^{13}C\{^1H\}$ NMR (100.62 MHz, CD_2Cl_2, 298 K): δ 168.0 (d, $^3J_{CP}$ = 5.4, C^9), 166.6 (d, $^2J_{CP}$ = 120.0, C^{15}), 151.6 (d, $^2J_{CP}$ = 11.7, C^1), 147.1 (s, C^{10}), 146.2 (s, C^7), 142.3 (d, $^2J_{CP}$ = 1.3, C^3), 135.3 (d, $^4J_{CP}$ = 2.1, C^5), 135.1 (d, $^4J_{CP}$ = 9.6, C^{13}), 130.7 (s, C^{12}), 130.6 (d, $^3J_{CP}$ = 7.0, C^6), 130.6 (d, $^3J_{CP}$ = 6.7, C^4), 129.6 (d, $^3J_{CP}$ = 3.8, C^{14}), 129.5 (d, $^4J_{CP}$ = 5.4, C^{11}), 125.1 (d, $^1J_{CP}$ = 38.3, C^2), 120.6 (s, C^8), 40.0 (d, $^1J_{CP}$ = 14.4, C^{16}), 29.7 (d, $^2J_{CP}$ = 3.4, C^{17}). $^{31}P\{^1H\}$ NMR (161.99 MHz, CD_2Cl_2, 298 K): δ 76.2 (s, *P*). Impurities of water (1.54 ppm) and grease (0.08 ppm) were found in the ^1H NMR spectrum. (+)-HR-ESI-MS (*m/z*): calcd for $C_{23}H_{27}N_4AuP$ $[M]^+$, 587.16334; found, 587.16270. IR (ATR, cm^{-1}): $\tilde{v}$ (N_3) 2048 (s).

(P^N^C)Au-$^{15}N_3$ (1-^{15}N)

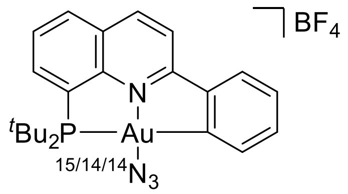

^1H and $^{31}P\{^1H\}$ NMR spectra were identical to the non-labelled Au-N_3. $^{15}N\{^1H\}$ NMR (50.71 MHz, CD_2Cl_2, 298 K): δ 188.4 (s), 64.2 (s). (+)-HR-ESI-MS (*m/z*): calcd for $C_{23}H_{27}N_3AuP^{15}N$ $[M]^+$, 588.16039; found, 588.16021. IR (ATR, cm^{-1}): $\tilde{v}$ (N_3) 2049 (s), 2026 (s).

(P^N^C)Au-NO_2 (3)

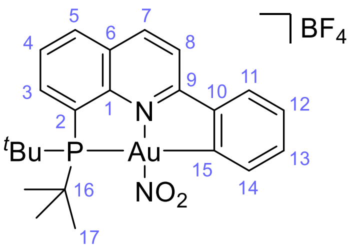

^1H NMR (500.30 MHz, CD_2Cl_2, 298 K): δ 9.05 (dd, $^3J_{HH}$ = 8.8, $^5J_{HP}$ = 1.5, 1H, H^7), 8.48-8.44 (m, 2H, H^5 and H^3), 8.37 (d, $^3J_{HH}$ = 8.8, 1H, H^8), 8.12 (ddd, $^3J_{HH}$ = 7.8, $^3J_{HH}$ = 7.7, $^4J_{HP}$ = 1.8, 1H, H^4), 8.03-7.99 (m, 1H, H^{11}), 7.66-7.60 (m, 2H, H^{12} and H^{13}), 7.48-7.43 (m, 1H, H^{14}), 1.59 (d, $^3J_{HP}$ = 17.4, 18H, H^{17}). $^{13}C\{^1H\}$ NMR (125.81 MHz, CD_2Cl_2, 298 K): δ 169.7 (d, $^3J_{CP}$ = 5.9, C^9), 167.0 (d, $^2J_{CP}$ = 101.3, C^{15}), 151.7 (d, $^2J_{CP}$ = 11.0, C^1), 147.0 (s, C^7), 145.5 (s, C^{10}), 142.8 (s, C^3), 135.6 (d, $^4J_{CP}$ = 2.2, C^5), 135.7 (d, $^4J_{CP}$ = 8.5, C^{13}), 132.9 (d, $^3J_{CP}$ = 2.2, C^{14}), 130.8 (d, $^3J_{CP}$ = 7.0, C^4), 130.9 (s, C^{12}), 130.3 (d, $^3J_{CP}$ = 7.0, C^6), 129.4 (d, $^4J_{CP}$ = 5.0, C^{11}), 124.4

(d, $^{1}J_{CP}$ = 39.5, C^{2}), 120.9 (s, C^{8}), 40.6 (d, $^{1}J_{CP}$ = 15.3, C^{16}), 29.8 (d, $^{2}J_{CP}$ = 3.7, C^{17}). ^{31}P{^{1}H} NMR (161.99 MHz, CD_2Cl_2, 298 K): δ 81.8 (s, *P*). (+)-HR-ESI-MS (*m/z*): calcd for $C_{23}H_{27}O_2N_2AuP$ [M]$^{+}$, 591.14702; found, 591.14735. IR (ATR, cm^{-1}): $\tilde{\nu}$ (NO_2) 1329 (m).

(P^{NH_2}^N^C)Au-OH (4)

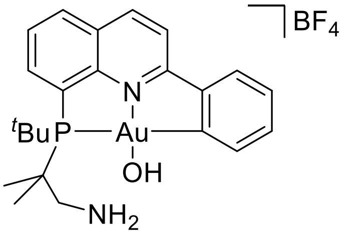

Complex **4** was obtained in a mixture of Au-N_3 **1** (30%), Au-NO_2 **3** (20%) and **4** (50 %). Only the signals that can be assigned to **4** are given. ^{1}H NMR (400.13 MHz, CD_2Cl_2, 298 K): δ 8.88 (dd, $^{3}J_{HH}$ = 9.0, 1H), 8.27 (d, $^{3}J_{HH}$ = 8.8, 1H), 7.60-7.54 (m, 2H, overlapped), 3.25 (dd, $^{2}J_{HH}$ = 13.5, $^{3}J_{HP}$ = 13.1, 1H), 2.85 (dd, $^{2}J_{HH}$ = 13.5, $^{3}J_{HP}$ = 23.5, 1H), 1.74 (d, $^{3}J_{HP}$ = 13.1, 3H), 1.52 (overlapped), 1.40 (d, $^{3}J_{HP}$ = 17.6, 3H). ^{13}C{^{1}H} NMR could not be measured due to very low concentration. ^{31}P{^{1}H} NMR (161.99 MHz, CD_2Cl_2, 298 K): δ 71.9 (s, *P*). (+)-HR-ESI-MS (*m/z*): calcd for $C_{23}H_{29}ON_2AuP$ [M]$^{+}$, 577.16832; found, 577.17660. IR (ATR, cm^{-1}): $\tilde{\nu}$ (NH_2, bend) 1669 (m).

(P^N^C)Au-NCO (5)

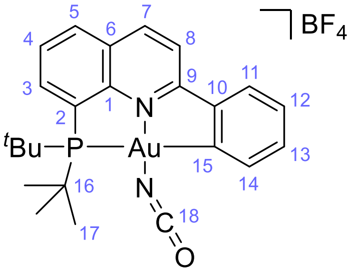

^{1}H NMR (500.30 MHz, CD_2Cl_2, 298 K): δ 9.06 (d, $^{3}J_{HH}$ = 8.8, H^{7}), 8.49 (d, $^{3}J_{HH}$ = 8.0, H^{5}), 8.46 (dd, $^{3}J_{HH}$ = 7.7, $^{3}J_{HP}$ = 7.7, H^{3}), 8.39 (d, $^{3}J_{HH}$ = 8.9, H^{8}), 8.11 (dd, $^{3}J_{HH}$ = 7.8, $^{3}J_{HH}$ = 7.8, H^{4}), 8.03 (dd, $^{3}J_{HH}$ = 7.4, $^{5}J_{HP}$ = 4.2, H^{11}), 7.80 (dd, $^{3}J_{HH}$ = 8.6, $^{4}J_{HP}$ = 8.4, H^{14}), 7.71 (ddd, $^{3}J_{HH}$ = 7.4, $^{3}J_{HH}$ = 7.2, $^{5}J_{HP}$ = 3.8, H^{13}), 7.64 (dd, $^{3}J_{HH}$ = 7.6, $^{3}J_{HH}$ = 7.6, H^{12}), 1.59 (d, $^{3}J_{HP}$ = 16.9, 18H, H^{17}). ^{13}C{^{1}H} NMR (125.82 MHz, CD_2Cl_2, 298 K): δ 169.7 (d, $^{3}J_{CP}$ = 5.8, C^{9}), 167.4 (d, $^{2}J_{CP}$ = 121.2, C^{15}), 151.9 (d, $^{2}J_{CP}$ = 12.0, C^{1}), 146.6 (s, C^{7}), 146.4 (d, $^{3}J_{CP}$ = 1.1, C^{10}), 142.3 (d, $^{2}J_{CP}$ = 1.1, C^{3}), 135.5 (d, $^{4}J_{CP}$ = 2.1, C^{5}), 135.2 (d, $^{4}J_{CP}$ = 9.5, C^{13}), 130.8 (d, $^{3}J_{CP}$ = 2.0, C^{14}), 130.8 (d, $^{3}J_{CP}$ = 6.9, C^{4}), 130.7 (s, C^{12}), 130.6 (d, $^{3}J_{CP}$ = 7.3, C^{6}), 130.0 (s, C^{18}), 129.5 (d, $^{4}J_{CP}$ = 5.6, C^{11}), 124.8 (d, $^{1}J_{CP}$ = 38.1, C^{2}), 120.7 (s, C^{8}), 40.1 (d, $^{1}J_{CP}$ = 14.3, C^{16}), 29.8 (d, $^{2}J_{CP}$ = 3.2, C^{17}). ^{31}P{^{1}H} NMR (161.99 MHz, CD_2Cl_2, 298 K): δ 80.3 (s, *P*). (+)-HR-ESI-MS (*m/z*): calcd for $C_{24}H_{27}ON_2AuP$ [M]$^{+}$, 587.15210; found, 587.15168. IR (ATR, cm^{-1}): $\tilde{\nu}$ (CO) 2206 (s).

(P^N^C)Au-N^{13}CO (5-^{13}C)

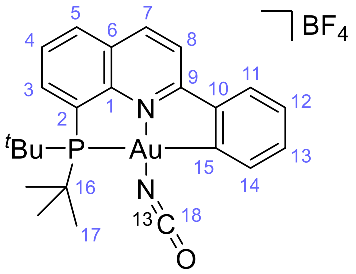

^{1}H NMR (400.13 MHz, CD_2Cl_2, 298 K): δ 9.06 (dd, $^3J_{HH}$ = 8.8, $^4J_{HP}$ = 1.5, 1H, H^7), 8.50-8.43 (m, 2H, H^3 and H^5), 8.39 (d, $^3J_{HH}$ = 8.9, 1H, H^8), 8.11 (ddd, $^3J_{HH}$ = 7.8, $^3J_{HH}$ = 7.8, $^4J_{HP}$ = 1.5, 1H H^4), 8.03 (ddd, $^3J_{HH}$ = 7.4, $^5J_{HP}$ = 4.2, $^4J_{HH}$ = 1.5, 1H, H^{11}), 7.82-7.78 (m, 1H, H^{14}), 7.71 (ddd, $^3J_{HH}$ = 7.4, $^3J_{HH}$ = 7.2, $^5J_{HP}$ = 3.8, 1H, H^{13}), 7.65-7.61 (m, 1H, H^{12}), 1.59 (d, $^3J_{HP}$ = 16.9, 18H, H^{17}). $^{13}C\{^1H\}$ NMR (100.62 MHz, CD_2Cl_2, 298 K): δ 169.7 (d, $^3J_{CP}$ = 5.8, C^9), 166.4 (d, $^3J_{CC}$ = 8.1, C^{15}), 146.6 (s, C^7), 146.4 (d, $^3J_{CP}$ = 1.1, C^{10}), 142.3 (d, $^2J_{CP}$ = 1.1, C^3), 135.5 (d, $^4J_{CP}$ = 2.1, C^5), 135.2 (d, $^4J_{CP}$ = 9.5, C^{13}), 130.8 (d, $^3J_{CP}$ = 2.0, C^{14}), 130.8 (d, $^3J_{CP}$ = 6.9, C^4), 130.7 (s, C^{12}), 130.6 (d, $^3J_{CP}$ = 7.3, C^6), 129.9 (s, C^{18}), 129.5 (d, $^4J_{CP}$ = 5.6, C^{11}), 124.8 (d, $^1J_{CP}$ = 38.1, C^2), 120.7 (s, C^8), 40.1 (d, $^1J_{CP}$ = 14.3, C^{16}), 29.8 (d, $^2J_{CP}$ = 3.2, C^{17}). C^1 not found. (+)-HR-ESI-MS (*m/z*): calcd for $C_{23}{}^{13}CH_{27}ON_2AuP$ $[M]^+$, 588.15546; found, 588.15536. IR (ATR, cm^{-1}): $\tilde{\nu}$ (CO) 2148 (s).

(P^N^C)Au-NHC(O)CH_3 (6)

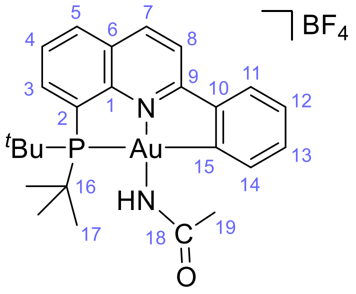

^{1}H NMR (400.13 MHz, CD_2Cl_2, 298 K): δ 8.91 (dd, $^3J_{HH}$ = 8.8, $^5J_{HP}$ = 1.3, 1H, H^7), 8.43-8.36 (m, 2H, H^3 and H^5), 8.30 (d, $^3J_{HH}$ = 8.8, 1H, H^8), 8.03 (ddd, $^3J_{HH}$ = 7.8, $^3J_{HH}$ = 7.8, $^4J_{HP}$ = 1.5, 1H, H^4), 7.95 (dd, $^3J_{HH}$ = 7.6, $^5J_{HP}$ = 3.9, H^{11}), 7.58-7.54 (m, 2H, H^{13} and H^{14}), 7.51-7.47 (m, 1H, H^{12}), 5.82 (br, 1H, NH), 2.27 (s, 3H, H^{19}), 1.56 (d, $^3J_{HP}$ = 16.7, 18H, H^{17}). $^{13}C\{^1H\}$ NMR (100.62 MHz, CD_2Cl_2, 298 K): δ 173.5 (br, C^{18}), 169.0 (d, $^3J_{CP}$ = 6.0, C^9), 165.7 (d, $^2J_{CP}$ = 124.6, C^{15}), 151.4 (d, $^2J_{CP}$ = 11.8, C^1), 146.7 (s, C^{10}), 145.4 (s, C^7), 141.9 (d, $^2J_{CP}$ = 1.4, C^3), 134.6 (d, $^4J_{CP}$ = 2.0, C^5), 134.5 (d, $^4J_{CP}$ = 8.9, C^{13}), 131.3 (d, $^3J_{CP}$ = 1.2, C^{14}), 130.2 (overlapped, C^6), 130.2 (d, $^3J_{CP}$ = 6.1, C^4), 129.9 (s, C^{12}), 128.8 (d, $^4J_{CP}$ = 5.8, C^{11}), 126.7 (d, $^1J_{CP}$ = 36.8, C^2), 120.2 (s, C^8), 39.6 (d, $^1J_{CP}$ = 14.1, C^{16}), 29.6 (d, $^2J_{CP}$ = 3.8, C^{17}), 25.2 (s, C^{19}). $^{31}P\{^1H\}$ NMR (161.99 MHz, CD_2Cl_2, 298 K): δ 74.5 (s, *P*). Traces of Et_2O (3.43 and 1.15 ppm; 66.1 and 15.4 ppm) and pentane (1.31 and 0.89 ppm; 34.6, 22.8 and 14.2 ppm) are found in the ^{1}H and $^{13}C\{^1H\}$ NMR spectra. (+)-HR-ESI-MS (*m/z*): calcd for $C_{25}H_{31}ON_2AuP$ $[M]^+$, 603.18340; found, 603.18270. IR (ATR, cm^{-1}): $\tilde{\nu}$ (NH) 3368 (br), (CO) 1594 (s).

(P^N^C)Au-NHC(O)CH_2CH_3 (7)

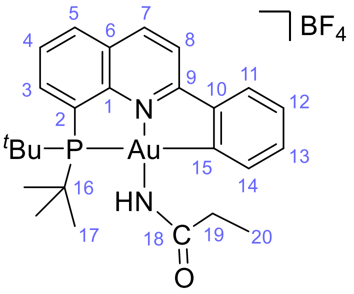

^{1}H NMR (400.13 MHz, CD_2Cl_2, 298 K): δ 8.91 (dd, $^3J_{HH}$ = 8.8, $^5J_{HP}$ = 1.0, 1H, H^7), 8.43-8.37 (m, 2H, H^3 and H^5), 8.31 (d, $^3J_{HH}$ = 8.8, 1H, H^8), 8.03 (ddd, $^3J_{HH}$ = 8.1, $^3J_{HH}$ = 8.0, $^4J_{HP}$ = 1.3, 1H, H^4), 7.96 (dd, $^3J_{HH}$ = 7.2, $^5J_{HP}$ = 4.0, H^{11}), 7.57-7.47 (m, 3H, H^{12}, H^{13} and H^{14}), 5.72 (br, 1H, NH), 2.54 (q, $^3J_{HH}$ = 7.6, 2H, H^{19}), 1.56 (d, $^3J_{HP}$ = 16.7, 18H, H^{17}), 1.25 (t, $^3J_{HH}$ = 7.6, 3H, H^{20}). ^{13}C{^{1}H} NMR (125.81 MHz, CD_2Cl_2, 298 K): δ 176.8 (br, C^{18}), 168.9 (d, $^3J_{CP}$ = 6.0, C^9), 165.6 (d, $^2J_{CP}$ = 124.9, C^{15}), 151.4 (d, $^2J_{CP}$ = 11.8, C^1), 146.8 (s, C^{10}), 145.4 (s, C^7), 141.9 (d, $^2J_{CP}$ = 1.4, C^3), 134.7 (d, $^4J_{CP}$ = 2.0, C^5), 134.4 (d, $^4J_{CP}$ = 9.0, C^{13}), 131.1 (d, $^3J_{CP}$ = 1.1, C^{14}), 130.2 (d, $^3J_{CP}$ = 6.9, C^6), 130.2 (d, $^3J_{CP}$ = 6.6, C^4), 130.0 (s, C^{12}), 128.8 (d, $^4J_{CP}$ = 5.8, C^{11}), 126.8 (d, $^1J_{CP}$ = 36.7, C^2), 120.2 (s, C^8), 39.7 (d, $^1J_{CP}$ = 14.0, C^{16}), 31.9 (s, C^{19}), 29.7 (d, $^2J_{CP}$ = 3.7, C^{17}), 11.2 (s, C^{20}). ^{31}P{^{1}H} NMR (161.99 MHz, CD_2Cl_2, 298 K): δ 74.3 (s, *P*). (+)-HR-ESI-MS (*m/z*): calcd for $C_{26}H_{33}ON_2AuP$ [M]$^+$, 617.19905; found, 617.19911. IR (ATR, cm^{-1}): $\tilde{\nu}$ (NH) 3365 (br), (CO) 1593 (s).

(P^N^C)Au-NC_2H_2 (8)

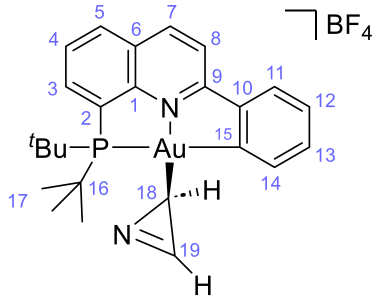

^{1}H NMR (400.13 MHz, CD_2Cl_2, 220 K): δ 10.40 (dd, $^3J_{HH}$ = 1.8, $^3J_{HP}$ = 1.8, 1H, H^{19}), 8.82 (dd, $^3J_{HH}$ = 8.9, $^5J_{HP}$ = 1.1, 1H, H^7), 8.37-8.28 (m, 3H, H^3, H^5, and H^8), 8.19 (dd, $^3J_{HH}$ = 7.9, $^3J_{HP}$ = 7.9, 1H, H^{14}), 8.09-8.06 (m, 1H, H^{11}), 7.95 (dd, $^3J_{HH}$ = 7.6, $^3J_{HP}$ = 7.3, 1H, H^4), 7.71-7.67 (m, 1H, H^{13}), 7.59-7.55 (m, 1H, H^{12}), 3.38 (dd, $^3J_{HH}$ = 1.8, $^3J_{HP}$ = 1.8, 1H, H^{18}), 1.60 (d, $^3J_{HP}$ = 16.3, 9H, H^{17a}), 1.41 (d, $^3J_{HP}$ = 16.7, 9H, H^{17b}). Purity (90 %). ^{13}C{^{1}H} NMR (100.62 MHz, CD_2Cl_2, 220 K): δ 172.3 (s, C^{19}), 164.5 (d, $^3J_{CP}$ = 6.1, C^9), 162.4 (d, $^2J_{CP}$ = 112.6, C^{15}), 149.8 (d, $^2J_{CP}$ = 10.7, C^1), 147.6 (s, C^{10}), 143.3 (s, C^7), 141.6 (s, C^3), 133.4 (d, $^4J_{CP}$ = 2.0, C^5), 133.5 (d, $^4J_{CP}$ = 8.1, C^{13}), 131.3 (d, $^3J_{CP}$ = 2.6, C^{14}), 129.1 (d, $^3J_{CP}$ = 6.6, C^4), 129.1 (s, C^{12}), 129.6 (d, $^3J_{CP}$ = 6.2, C^6), 128.2 (d, $^4J_{CP}$ = 5.3, C^{11}), 128.2 (d, $^1J_{CP}$ = 36.0, C^2), 119.3 (s, C^8), 38.2 (d, $^1J_{CP}$ = 15.9, C^{16a}), 37.9 (d, $^1J_{CP}$ = 15.6, C^{16b}), 33.1 (d, $^2J_{CP}$ = 5.7, C^{18}), 29.3 (d, $^2J_{CP}$ = 4.1, C^{17a}), 29.0 (d, $^2J_{CP}$ = 4.4, C^{17b}). ^{31}P{^{1}H} NMR (161.99 MHz, CD_2Cl_2, 298 K): δ 71.8 (s, *P*). (+)-HR-ESI-MS (*m/z*): calcd for $C_{25}H_{29}N_2AuP$ [M]$^+$, 585.17286; found, 585.17322. IR (ATR, cm^{-1}): $\tilde{\nu}$ (C=N) 1699 (br).

EPR Experiments.

An EPR tube was charged with 7 mg of complex **1**. The tube was sealed with a septum, and was purged with three vacuum/nitrogen cycles, through a needle. The tube was placed inside the spectrophotometer and cooled down to 10 K. A blank measurement was recorded. After that, the sample was warmed up to 80 K, removed from the spectrophotometer and placed in a liquid N_2 bath. Optimal conditions were found irradiating the sample at 80 K, using a PR160L-370 nm Gen 2 Kessil lamp. When the irradiation was completed, the sample was placed back inside the EPR cavity, cooled down to 10 K and EPR spectra acquired at X-band frequencies. This procedure was repeated after 15 and 60 min; however, no defined EPR signals were obtained, in line with the computed large ZFS parameters summarized in Supplementary Table 29.

3 Spectroscopic characterization

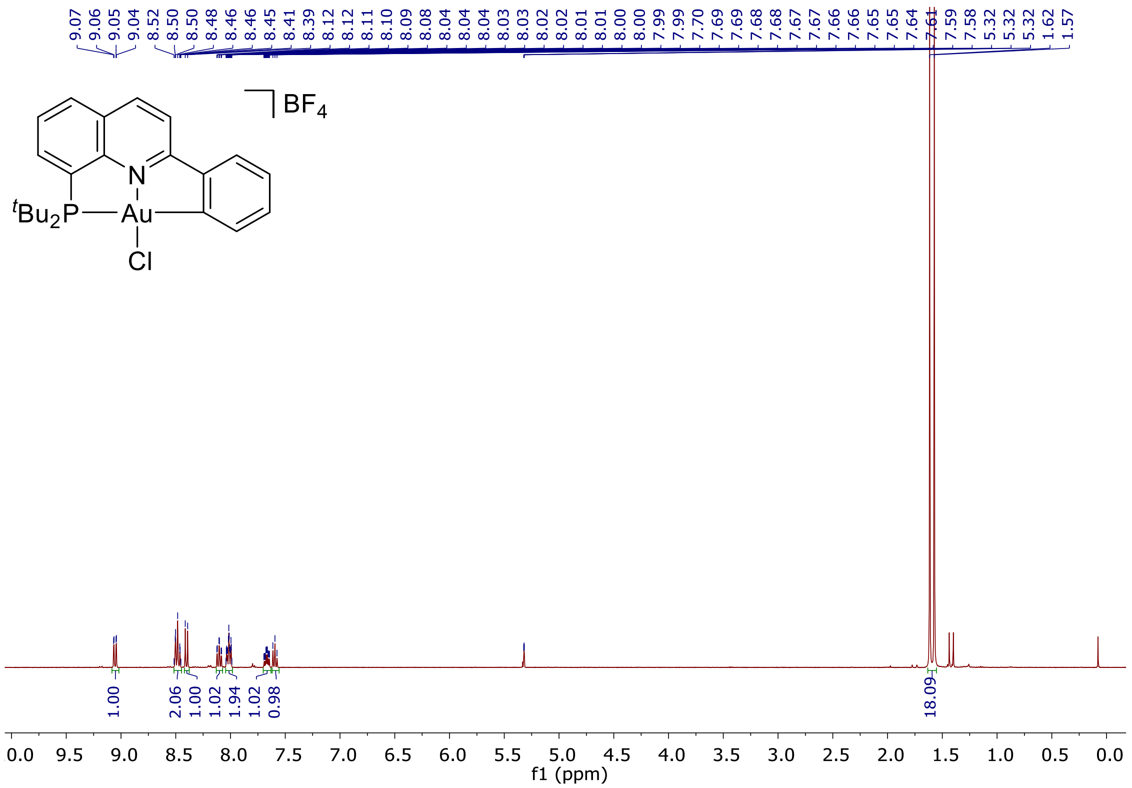

Supplementary Figure 1. ^{1}H NMR spectrum (400.13 MHz, CD_2Cl_2, 298 K) of **(PNC)AuCl**.

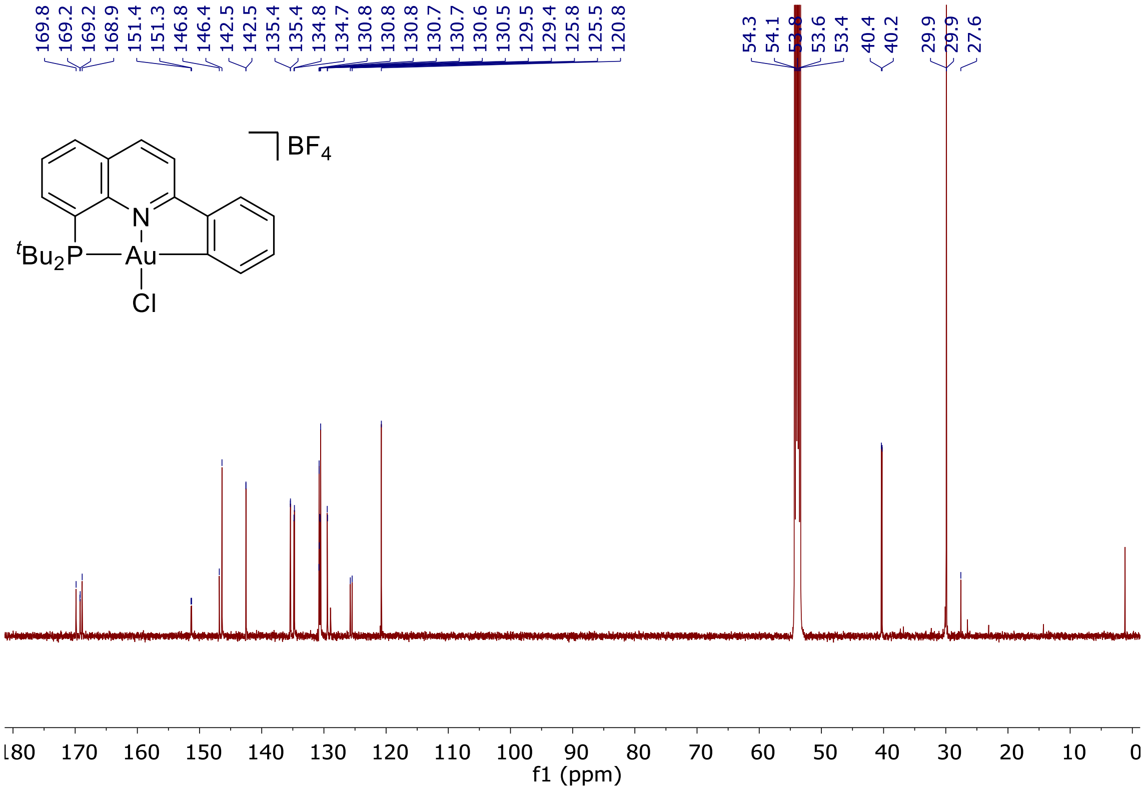

Supplementary Figure 2. $^{13}C\{^{1}H\}$ NMR spectrum (125.81 MHz, CD_2Cl_2, 298 K) of **(PNC)AuCl**.

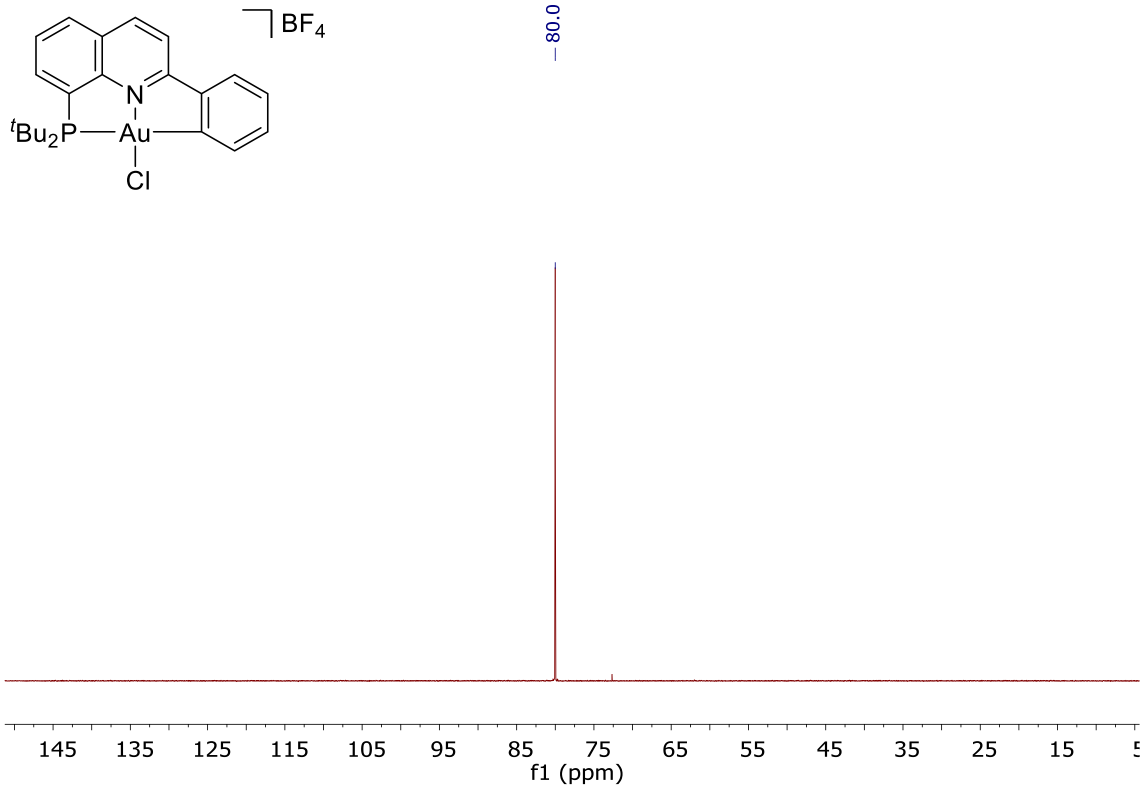

Supplementary Figure 3. $^{31}P\{^{1}H\}$ NMR spectrum (202.52 MHz, CD_2Cl_2, 298 K) of **(PNC)AuCl**.

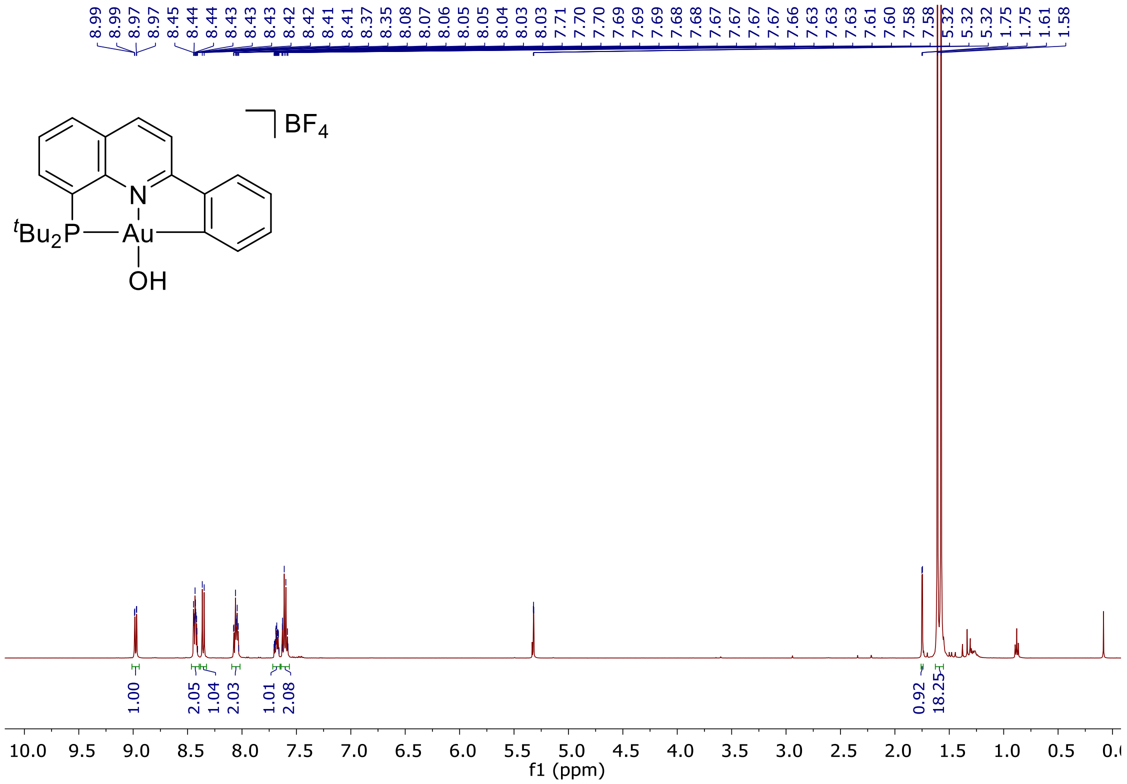

Supplementary Figure 4. ^{1}H NMR spectrum (500.30 MHz, CD_2Cl_2, 298 K) of **(PNC)AuOH**.

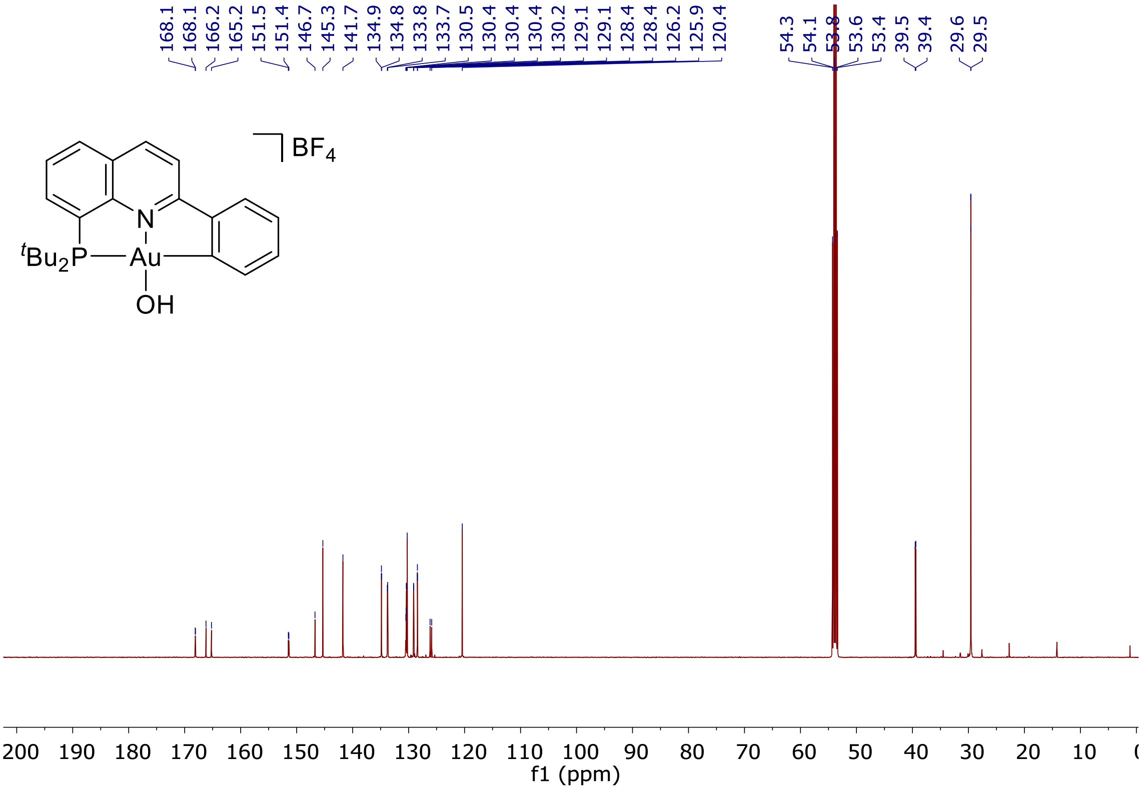

Supplementary Figure 5. $^{13}C\{^{1}H\}$ NMR spectrum (125.82 MHz, CD_2Cl_2, 298 K) of **(PNC)AuOH**.

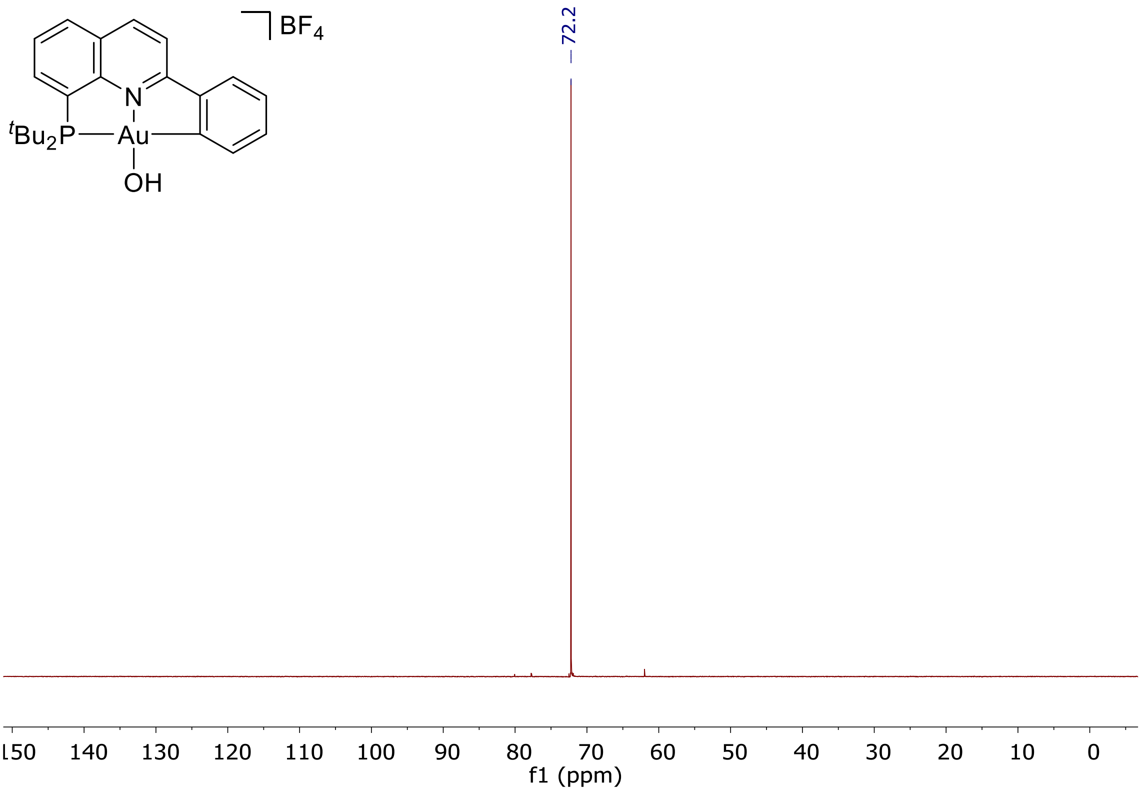

Supplementary Figure 6. $^{31}P\{^{1}H\}$ NMR spectrum (202.52 MHz, CD_2Cl_2, 298 K) of compound **(PNC)AuOH**.

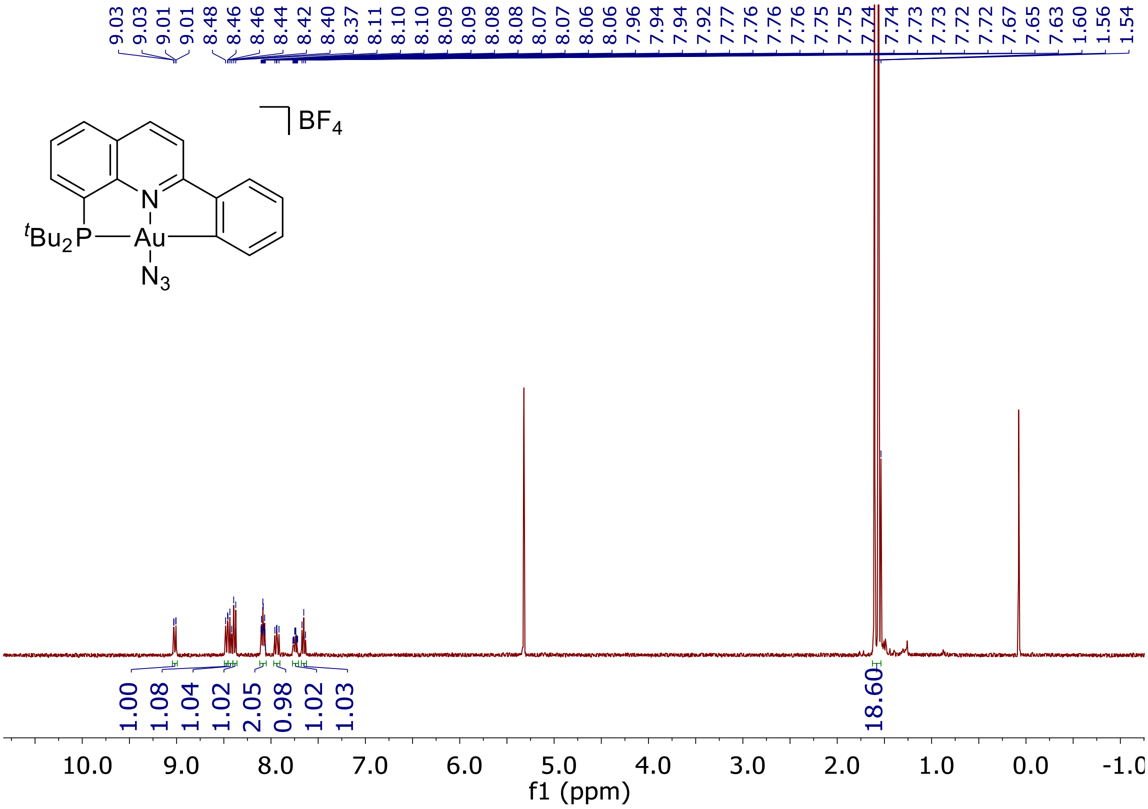

Supplementary Figure 7. ^{1}H NMR spectrum (400.13 MHz, CD_2Cl_2, 298 K) of **1**.

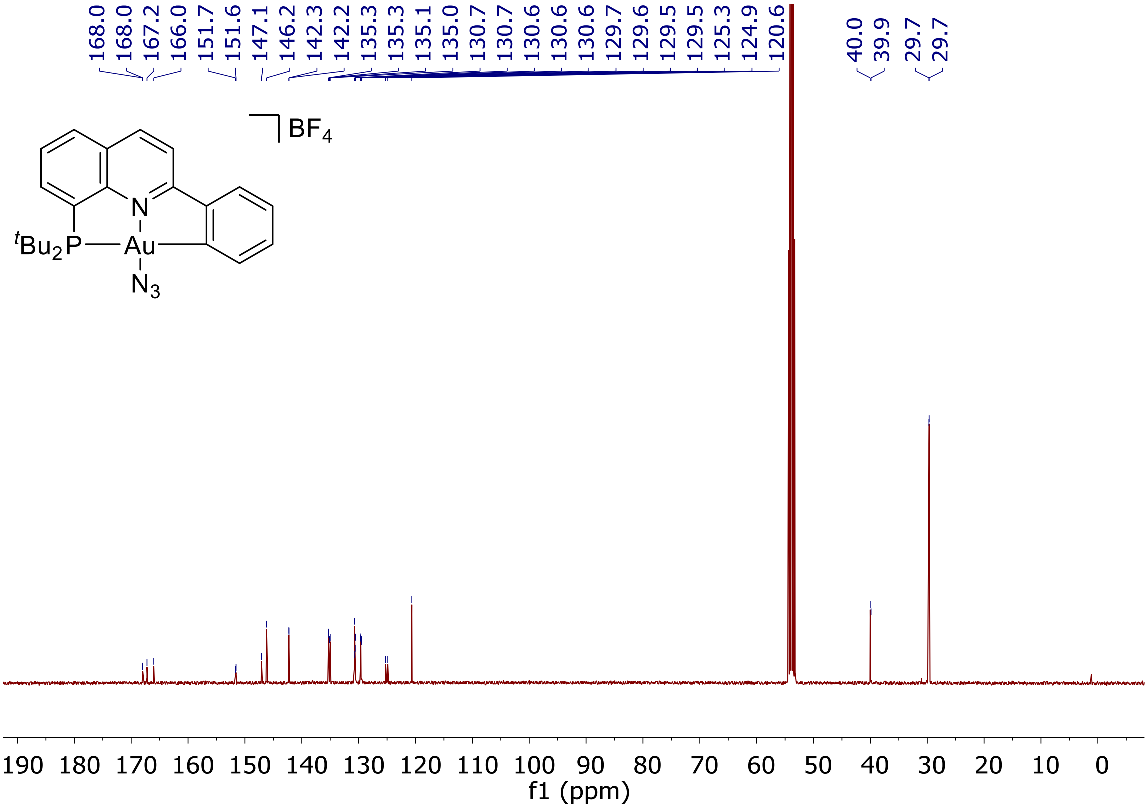

Supplementary Figure 8. $^{13}C\{^{1}H\}$ NMR spectrum (100.62 MHz, CD_2Cl_2, 298 K) of **1**.

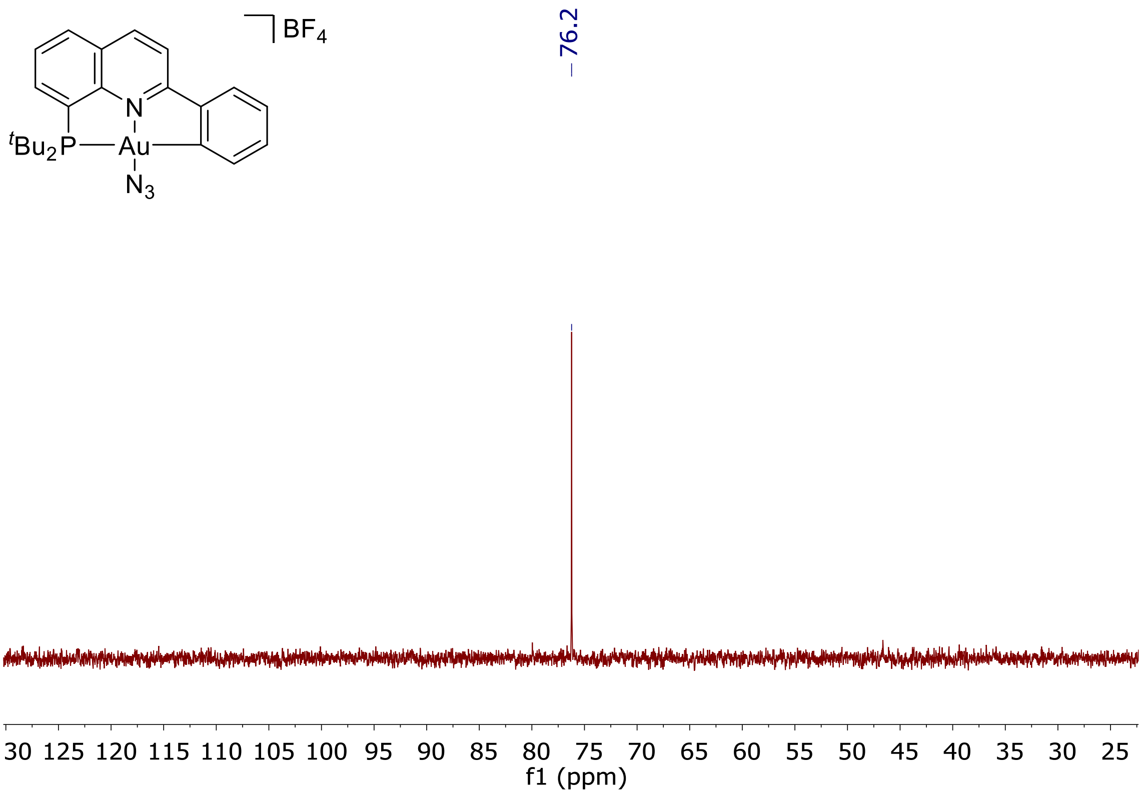

Supplementary Figure 9. $^{31}P\{^{1}H\}$ NMR spectrum (161.99 MHz, CD_2Cl_2, 298 K) of **1**.

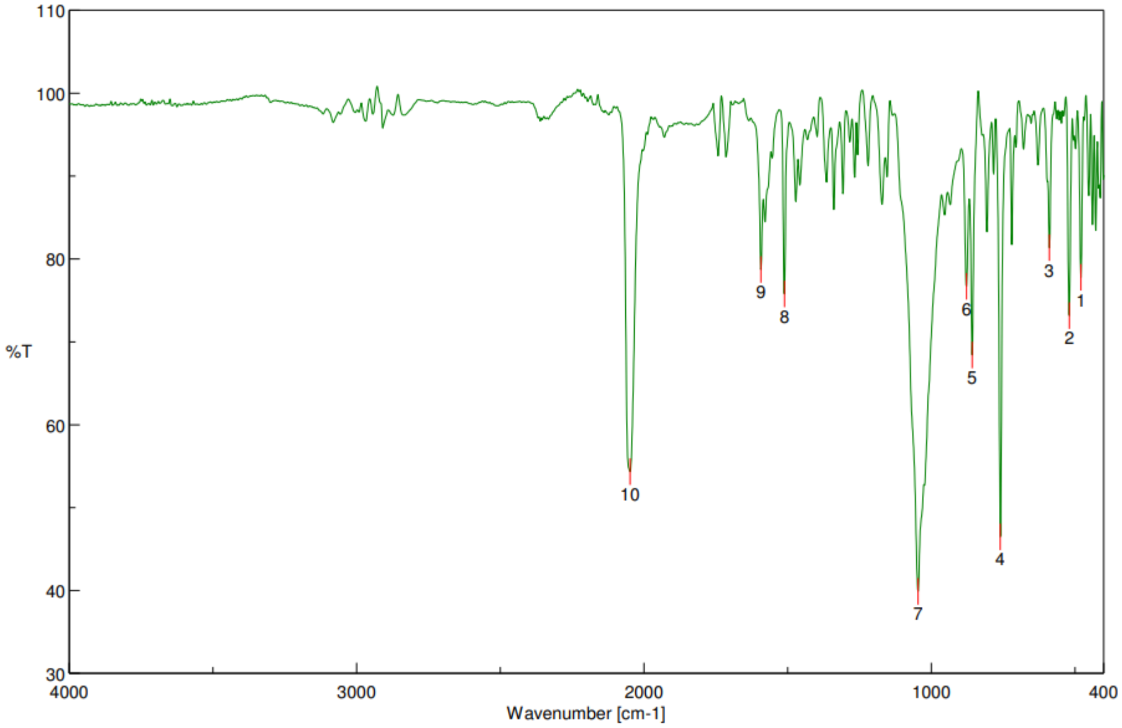

[Result of Peak Picking]

No.	Position	Intensity	No.	Position	Intensity	No.	Position	Intensity	No.	Position	Intensity
1	479.2	77.8	2	519.7	73.1	3	589.2	81.3	4	759.8	46.4
5	857.2	68.4	6	877.5	76.7	7	1046.2	39.9	8	1511.0	75.8
9	1592.9	78.7	10	2048.0	54.3						

Supplementary Figure 10. IR spectrum of **1**.

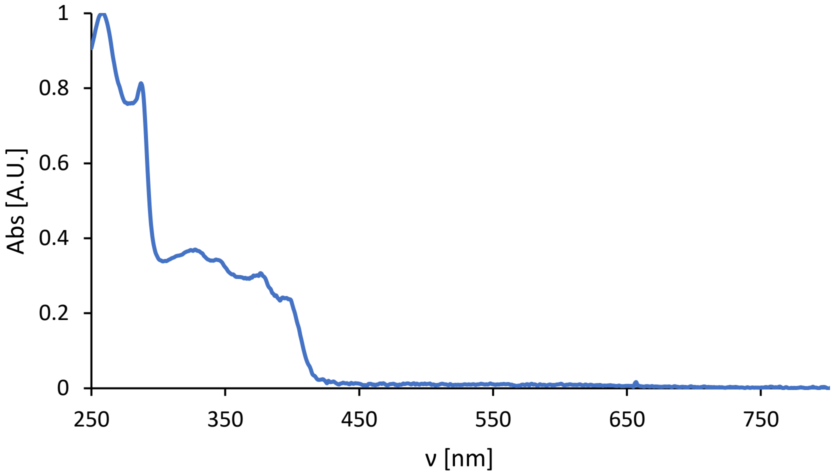

Supplementary Figure 11. UV-Vis spectrum of **1** in CH_2Cl_2 solution.

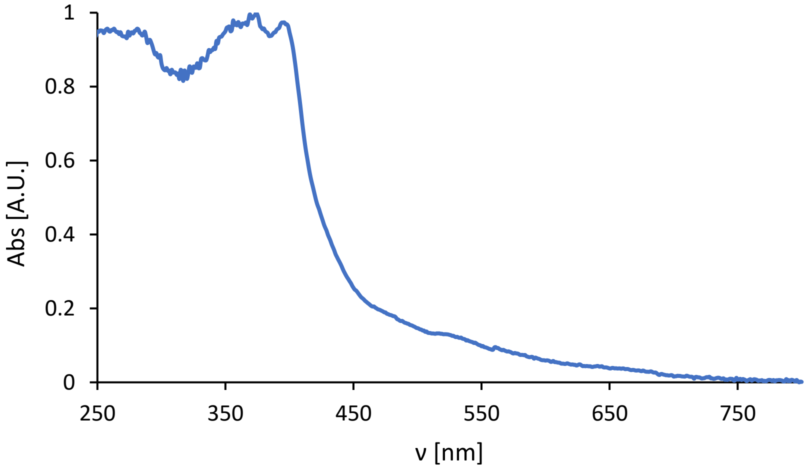

Supplementary Figure 12. Solid-state UV-Vis spectrum of **1**.

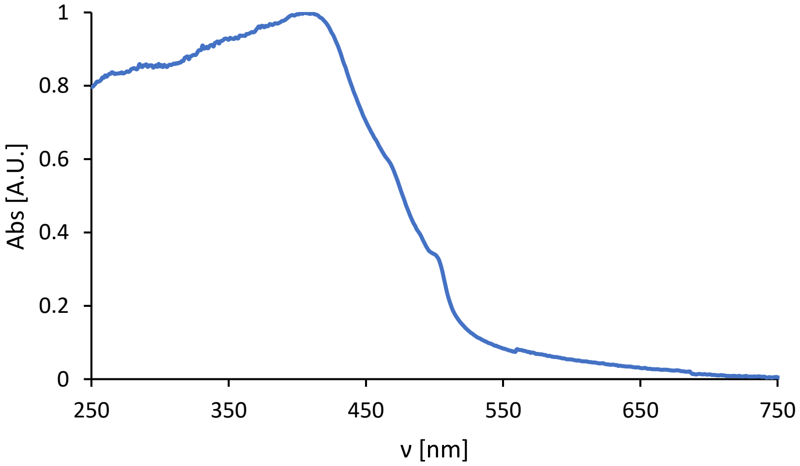

Supplementary Figure 13. Solid-state UV-Vis spectrum of **(P^N^C)Au-Cl**.

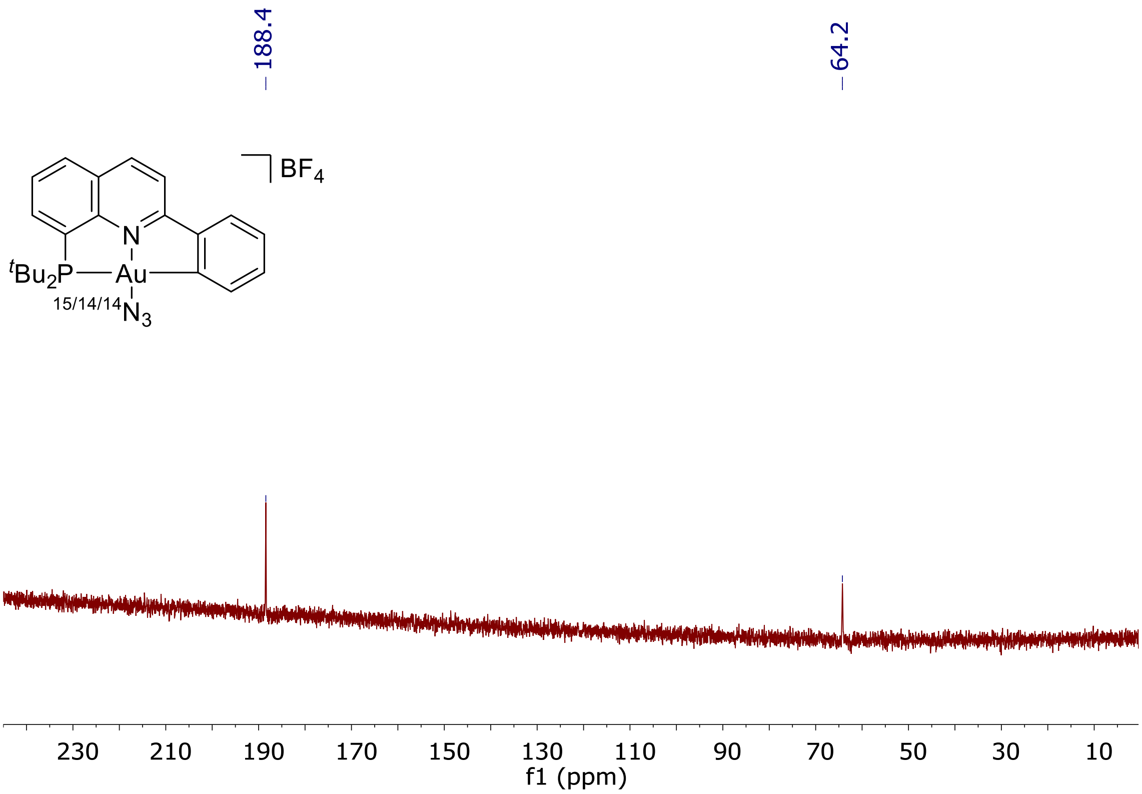

Supplementary Figure 14. $^{15}N\{^{1}H\}$ NMR spectrum (50.71 MHz, CD_2Cl_2, 298 K) of **1-^{15}N**.

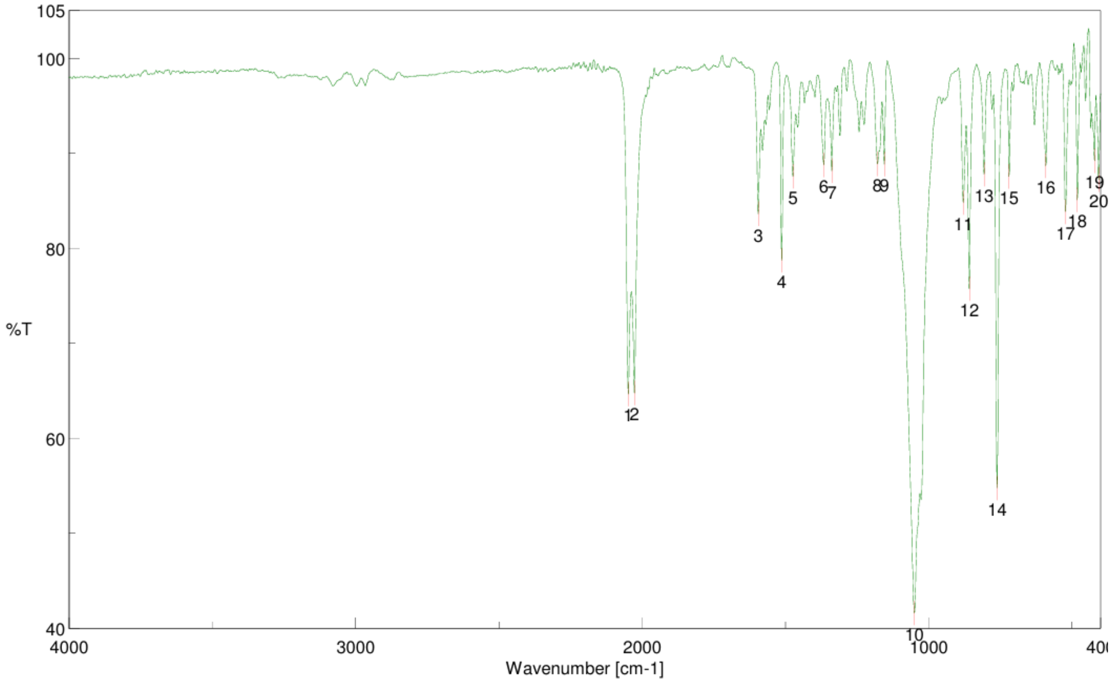

[Result of Peak Picking]

No.	Position	Intensity	No.	Position	Intensity	No.	Position	Intensity	No.	Position	Intensity
1	2049.0	64.7	2	2026.8	64.8	3	1594.8	83.6	4	1512.9	78.7
5	1474.3	87.6	6	1366.3	88.8	7	1338.4	88.2	8	1179.3	88.9
9	1155.2	88.8	10	1050.1	41.6	11	879.4	84.8	12	858.2	75.8
13	807.1	87.8	14	761.7	54.8	15	719.3	87.5	16	591.1	88.7
17	522.6	83.8	18	481.2	85.1	19	421.4	89.2	20	406.9	87.2

Supplementary Figure 15. IR spectrum of **1-^{15}N**.

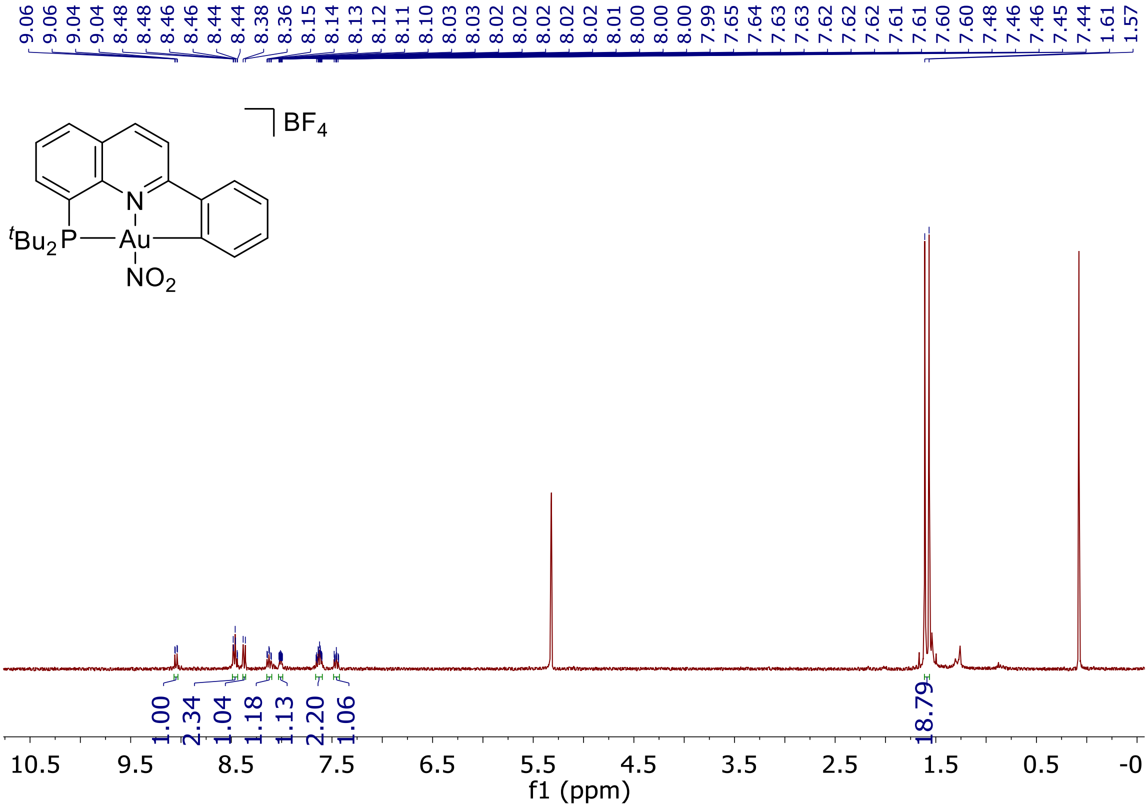

Supplementary Figure 16. ^{1}H NMR spectrum (400.13 MHz, CD_2Cl_2, 298 K) of **3**.

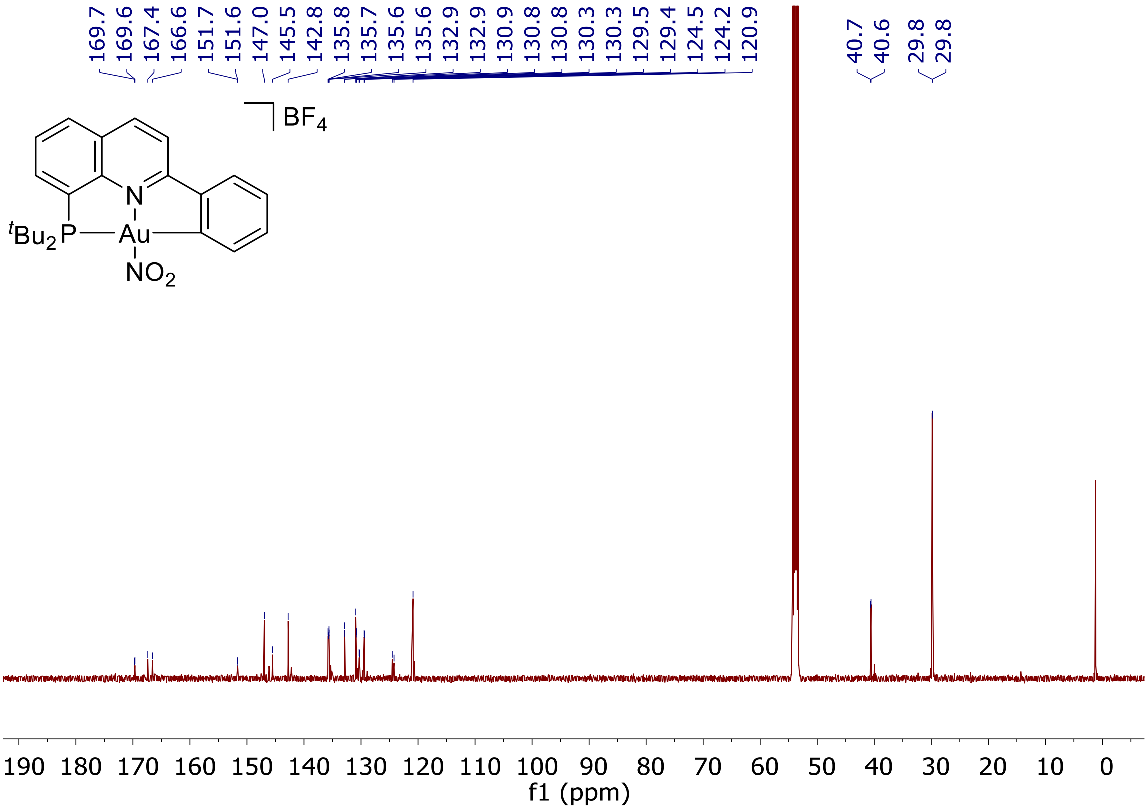

Supplementary Figure 17. $^{13}C\{^{1}H\}$ NMR spectrum (125.82 MHz, CD_2Cl_2, 298 K) of **3**.

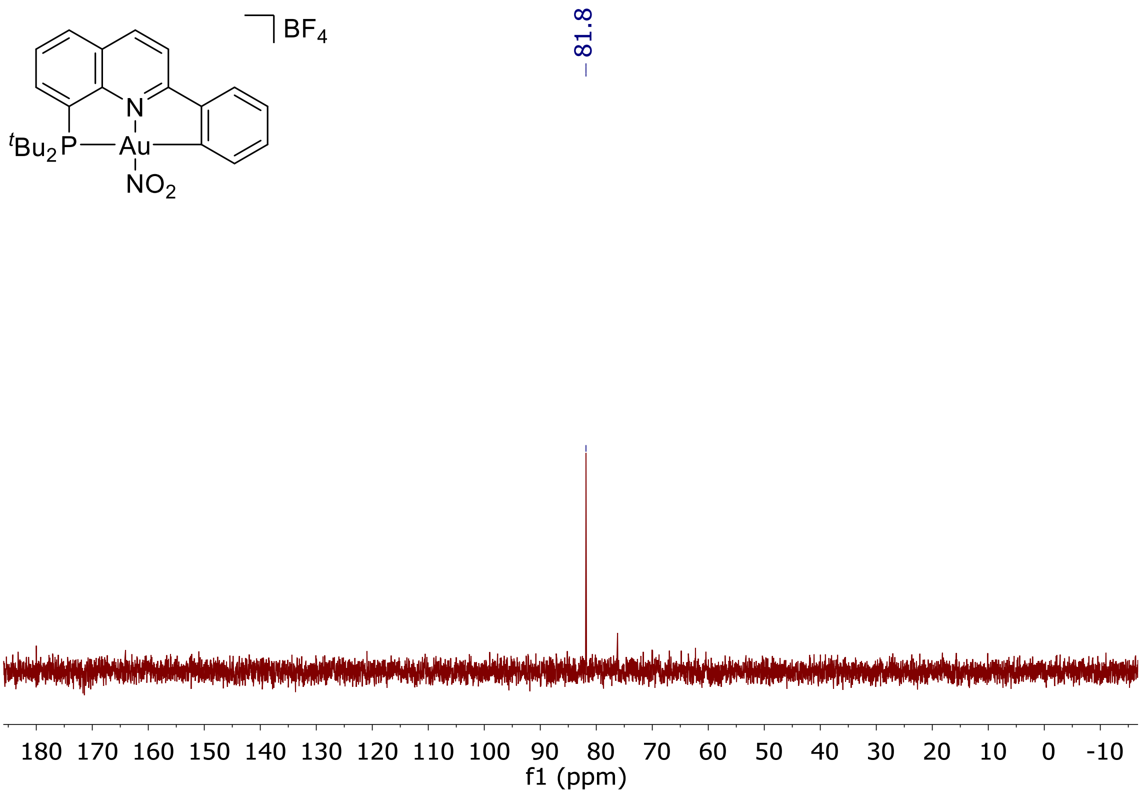

Supplementary Figure 18. ${}^{31}P\{{}^{1}H\}$ NMR spectrum (161.99 MHz, CD_2Cl_2, 298 K) of **3**.

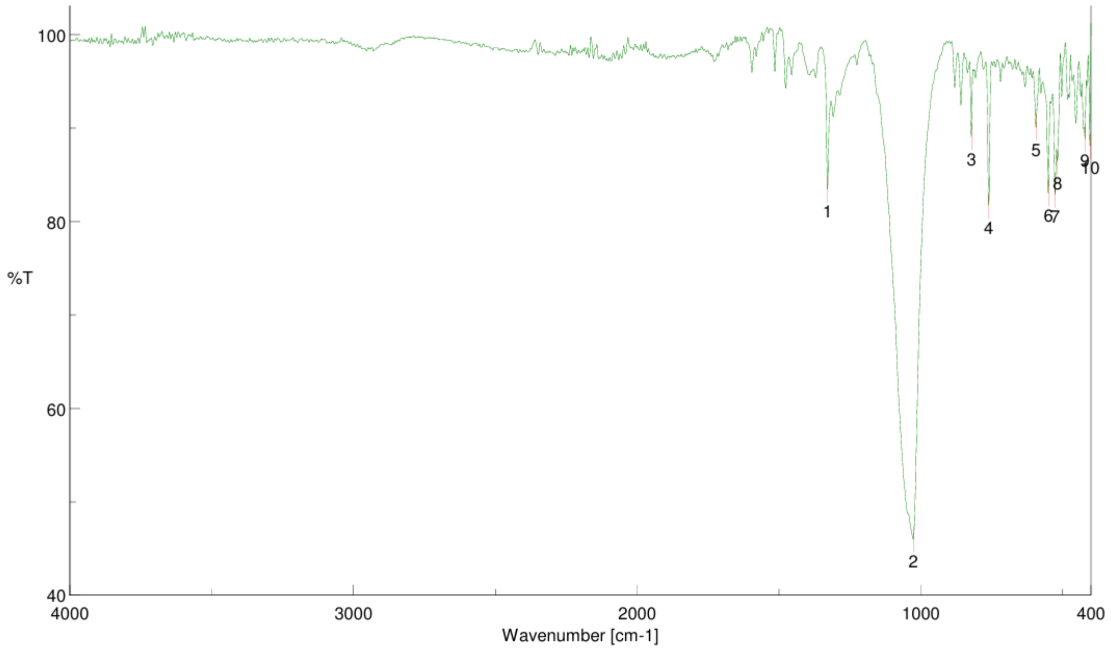

[Result of Peak Picking]

No.	Position	Intensity	No.	Position	Intensity	No.	Position	Intensity	No.	Position	Intensity
1	1328.7	83.4	2	1026.9	46.0	3	821.5	89.0	4	760.8	81.7
5	594.0	90.1	6	550.6	83.0	7	526.5	82.9	8	517.8	86.4
9	421.4	88.9	10	403.1	88.1						

Supplementary Figure 19. IR spectrum of **3**.

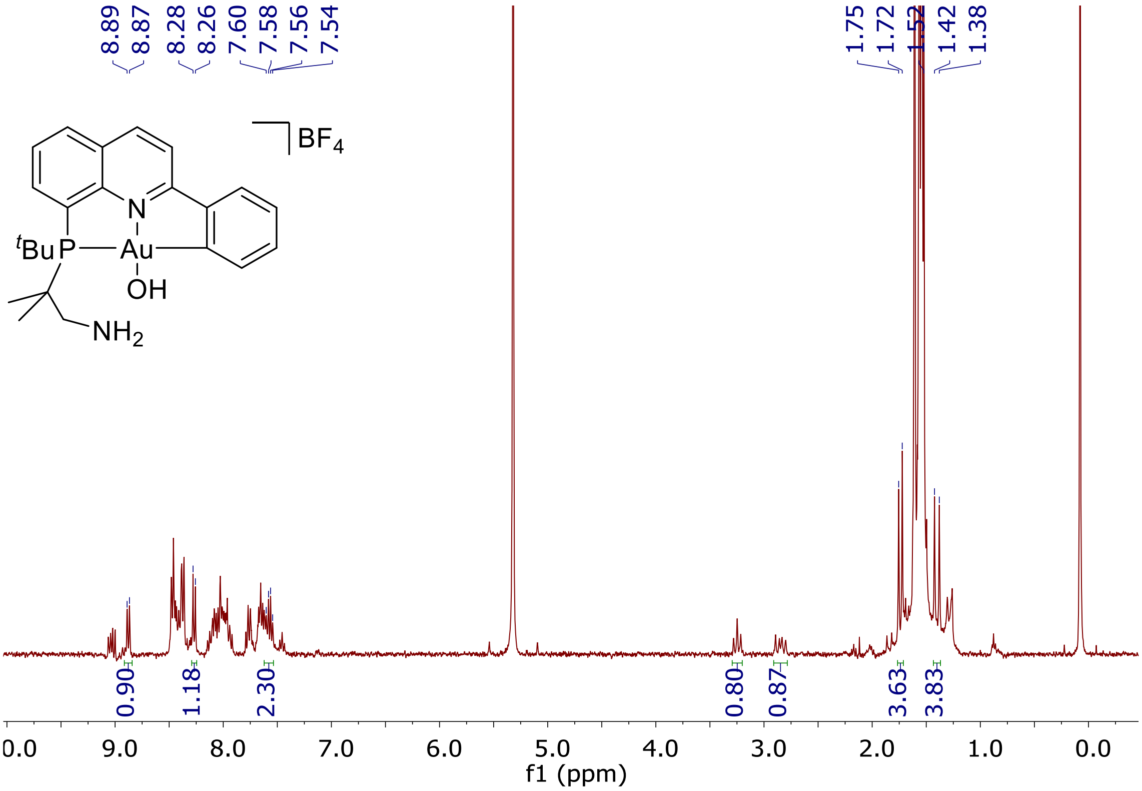

Supplementary Figure 20. ^{1}H NMR spectrum (400.13 MHz, CD_2Cl_2, 298 K) of **4** in a mixture with **1** and **3**. Only the signals that can be assigned to **4** are marked in the spectrum.

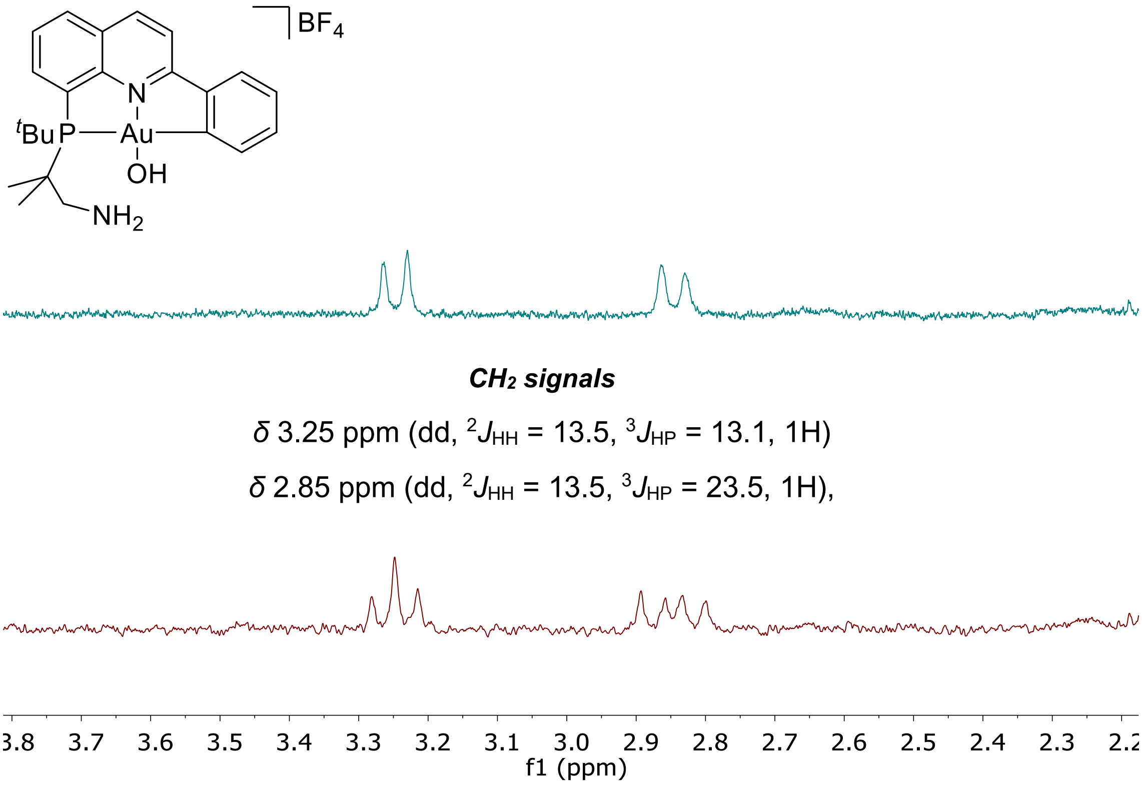

Supplementary Figure 21. $^{1}H\{^{31}P\}$ (top) vs ^{1}H (bottom) NMR spectra (400.13 MHz, CD_2Cl_2, 298 K) of **4**.

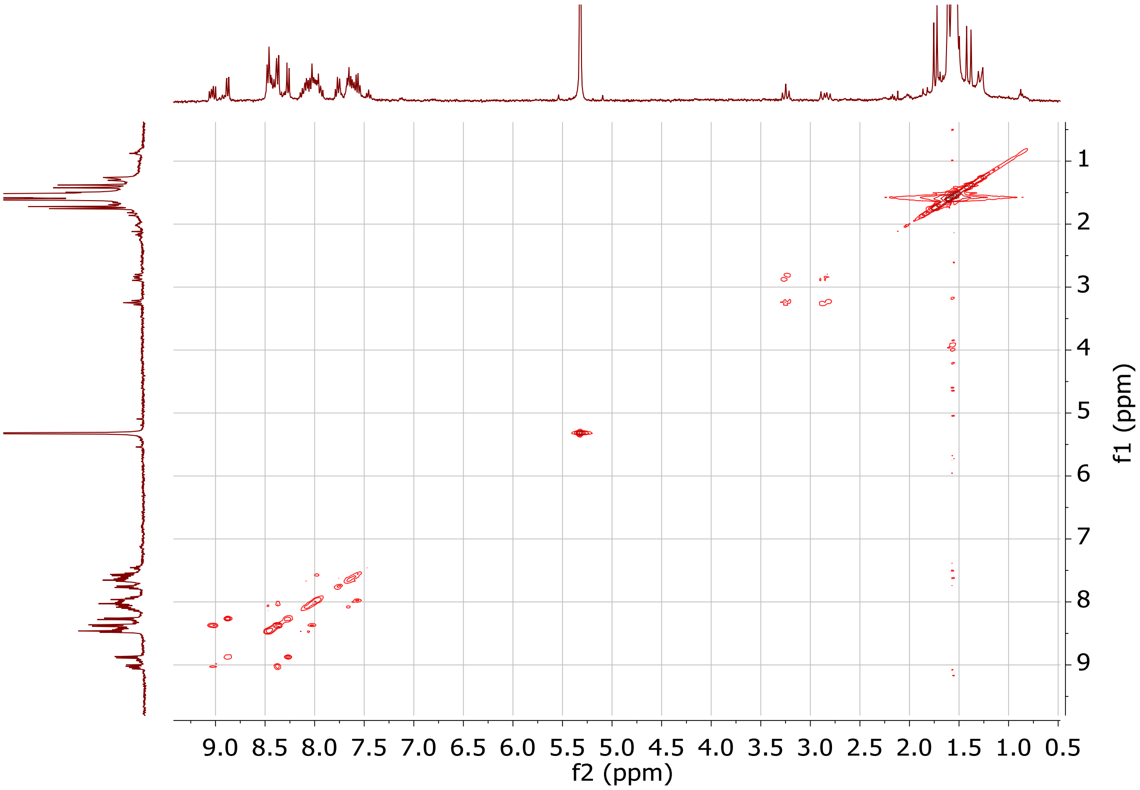

Supplementary Figure 22. ^{1}H-^{1}H COSY NMR spectrum (400.13 MHz, CD_2Cl_2, 298 K) of **4**.

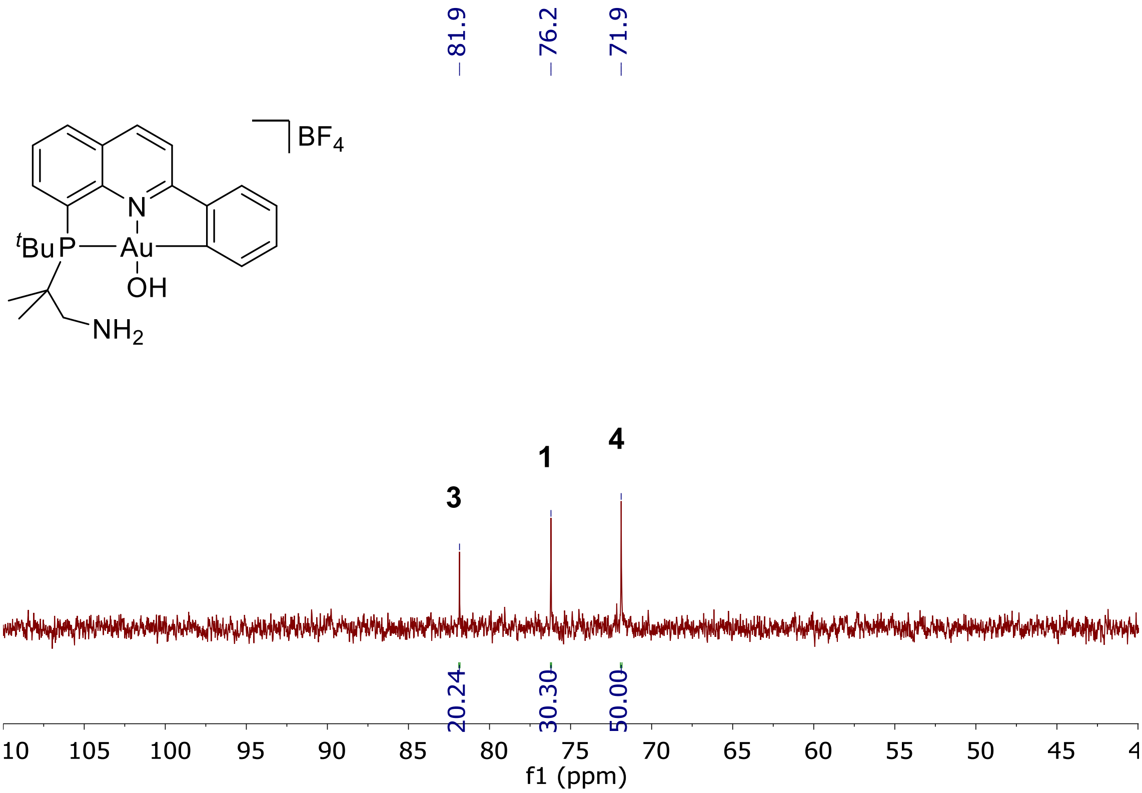

Supplementary Figure 23. ^{31}P{^{1}H} NMR spectrum (161.99 MHz, CD_2Cl_2, 298 K) with a mixture of **3** (81.9 ppm), **1** (76.2 ppm) and **4** (71.9 ppm).

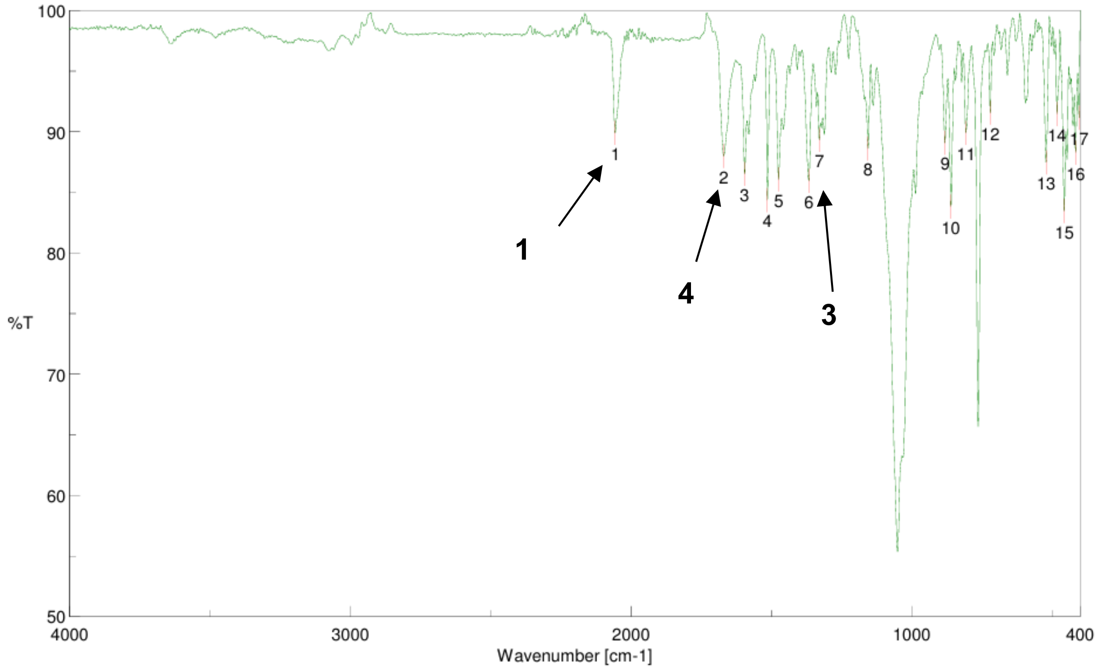

[Result of Peak Picking]

No.	Position	Intensity	No.	Position	Intensity	No.	Position	Intensity	No.	Position	Intensity
1	2055.8	89.9	2	1669.1	88.0	3	1594.8	86.5	4	1513.9	84.4
5	1473.4	86.1	6	1366.3	85.9	7	1328.7	89.3	8	1155.2	88.6
9	881.3	89.1	10	860.1	83.8	11	806.1	89.9	12	720.3	91.6
13	520.7	87.5	14	482.1	91.4	15	456.1	83.4	16	415.6	88.3
17	404.0	91.1									

Supplementary Figure 24. IR spectrum of **4**.

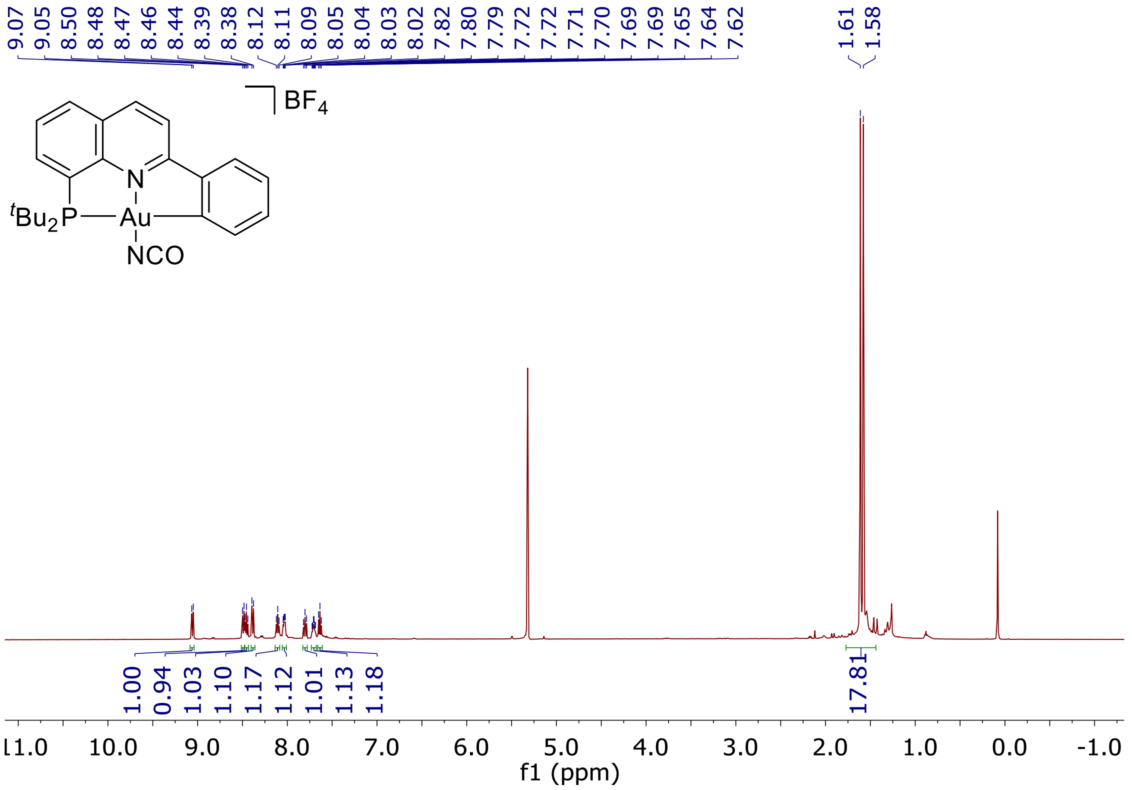

Supplementary Figure 25. ^{1}H NMR spectrum (500.30 MHz, CD_2Cl_2, 298 K) of **5**.

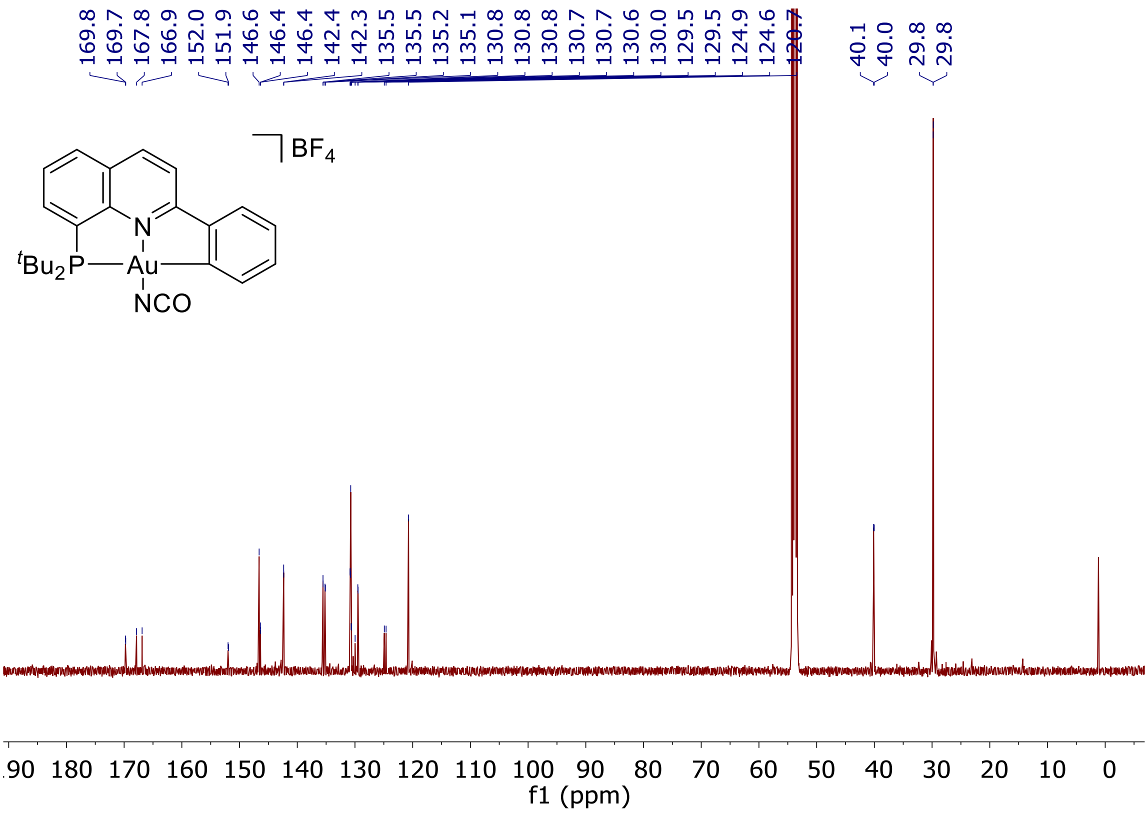

Supplementary Figure 26. $^{13}C\{^1H\}$ NMR spectrum (125.82 MHz, CD_2Cl_2, 298 K) of **5**.

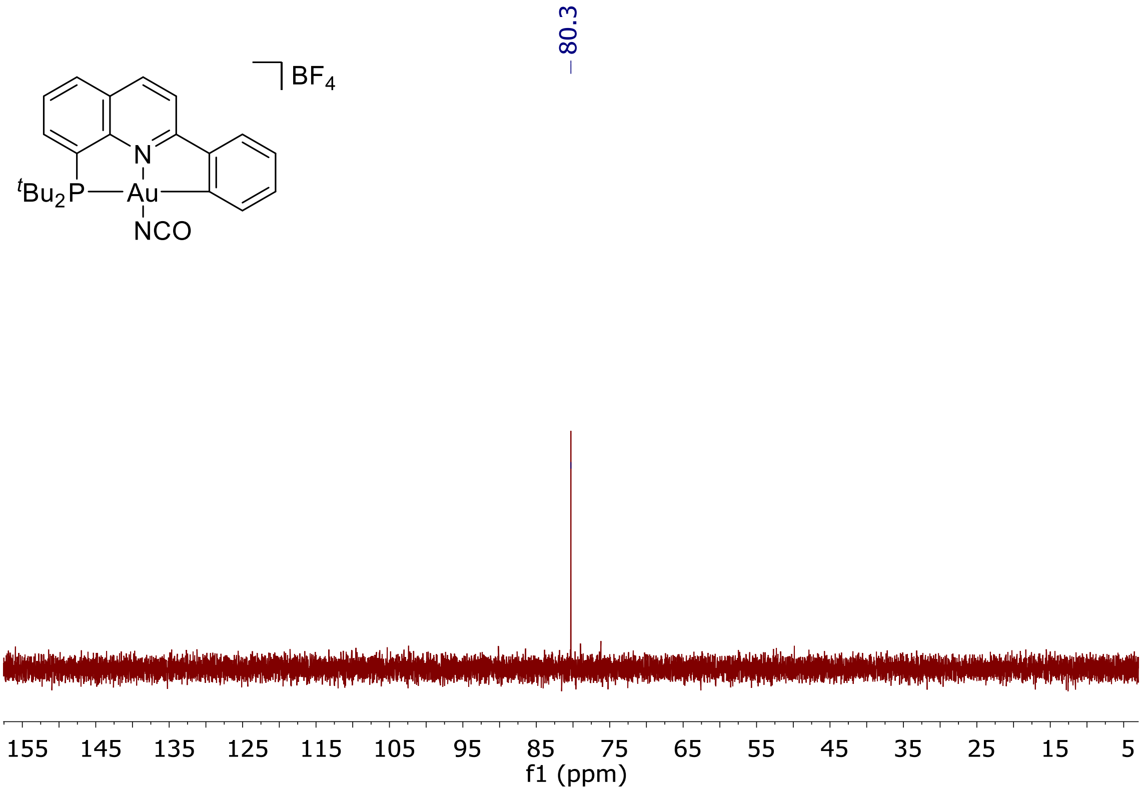

Supplementary Figure 27. $^{31}P\{^1H\}$ NMR spectrum (161.99 MHz, CD_2Cl_2, 298 K) of **5**.

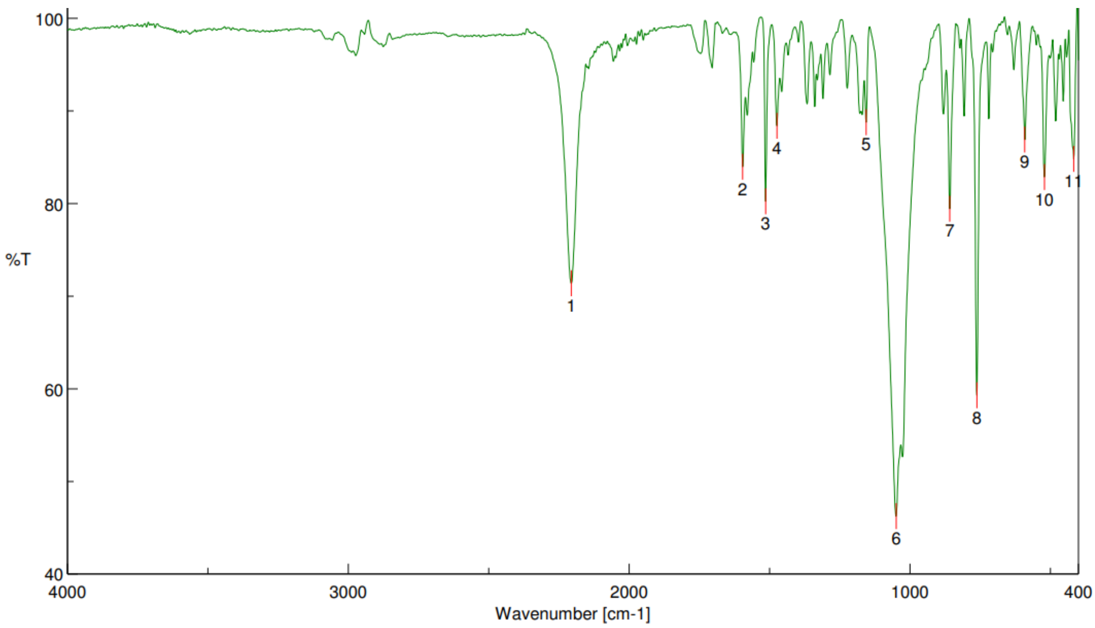

[Result of Peak Picking]

No.	Position	Intensity	No.	Position	Intensity	No.	Position	Intensity	No.	Position	Intensity
1	2206.2	71.4	2	1595.8	84.0	3	1513.9	80.2	4	1473.4	88.4
5	1156.1	88.7	6	1049.1	46.2	7	858.2	79.4	8	761.7	59.3
9	591.1	86.9	10	520.7	82.8	11	416.6	84.8			

Supplementary Figure 28. IR spectrum of **5**.

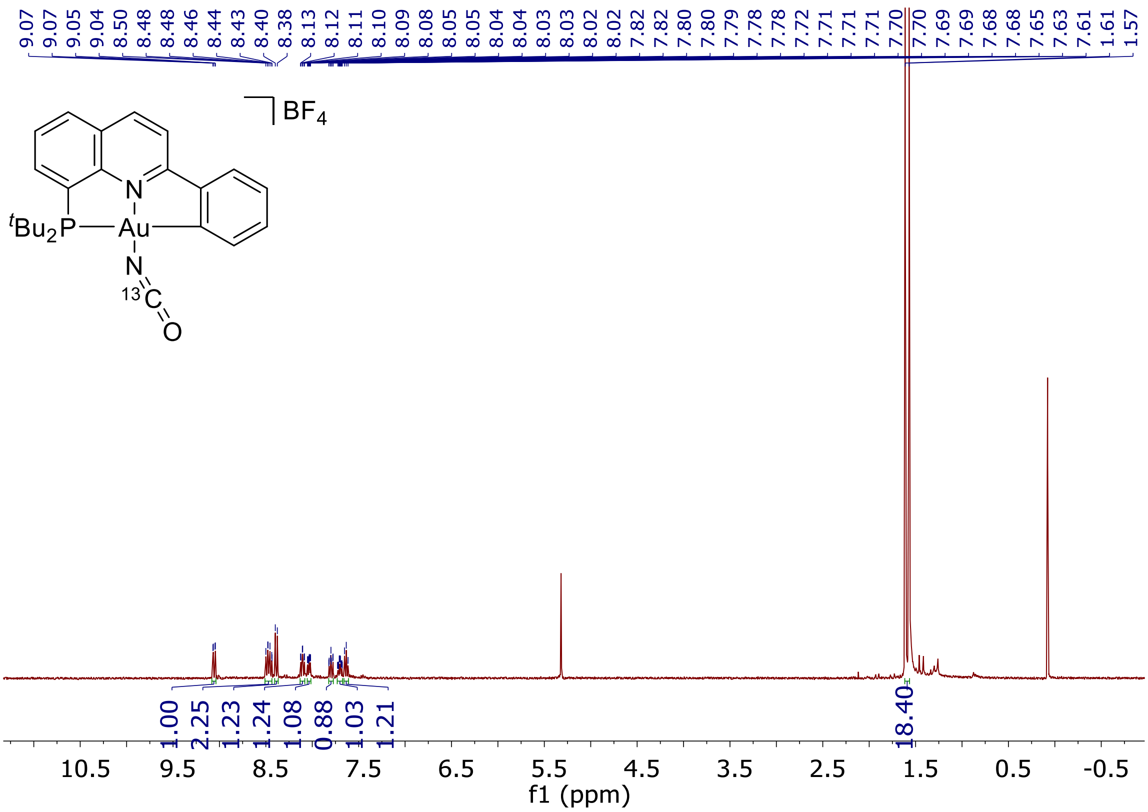

Supplementary Figure 29. ^{1}H NMR spectrum (400.13 MHz, CD_2Cl_2, 298 K) of **5-^{13}C**.

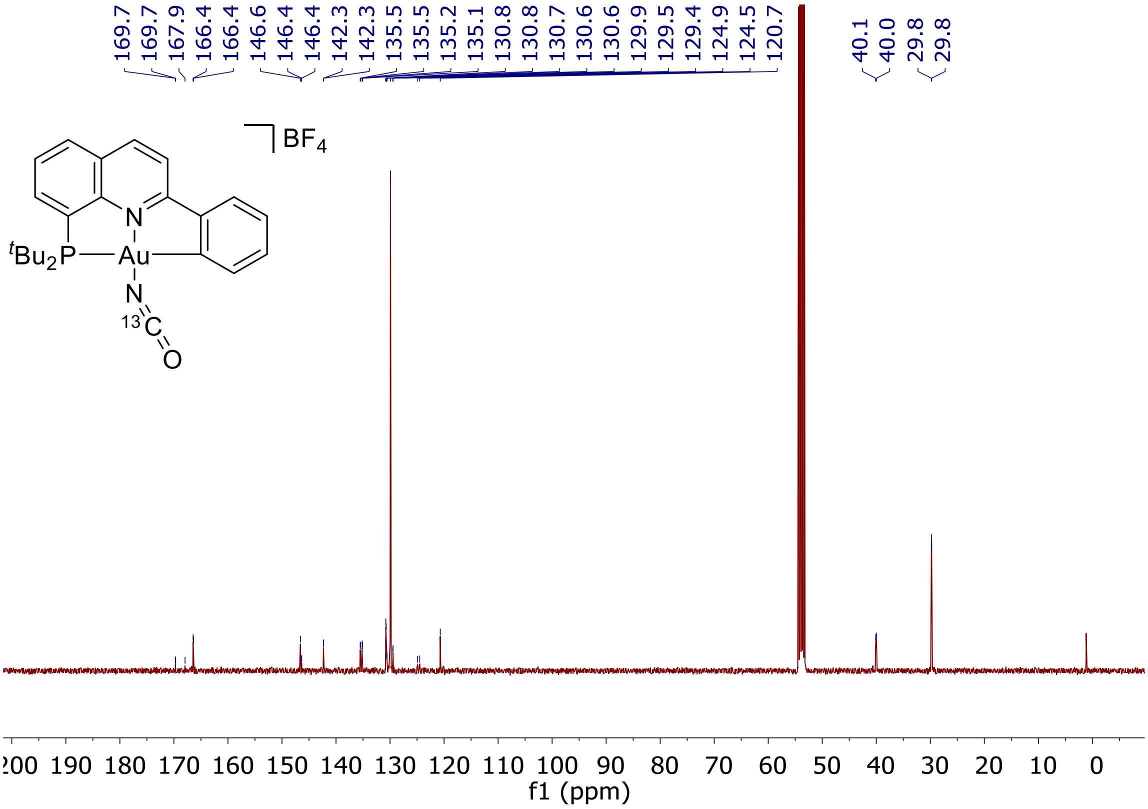

Supplementary Figure 30. $^{13}C\{^{1}H\}$ NMR spectrum (100.62 MHz, CD_2Cl_2, 298 K) of **5-^{13}C**.

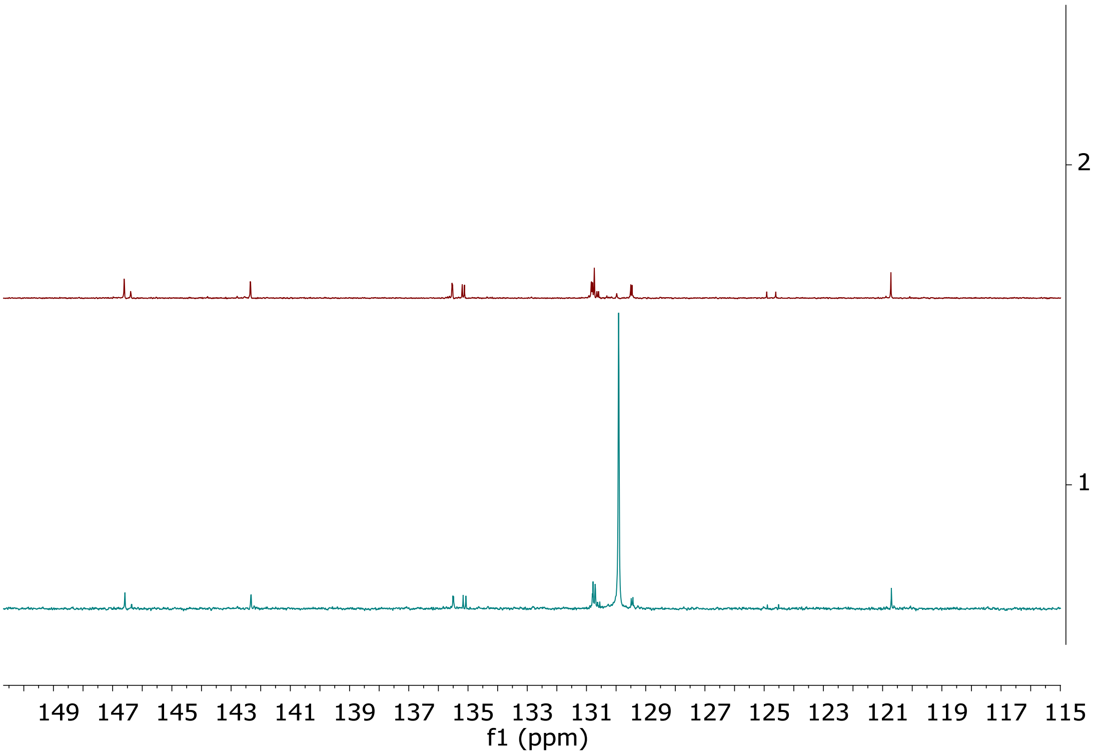

Supplementary Figure 31. Expanded region of the $^{13}C\{^{1}H\}$ NMR spectra of **5** (top) vs isotopically enriched **5-^{13}C** (bottom), showing the carbonyl carbon region.

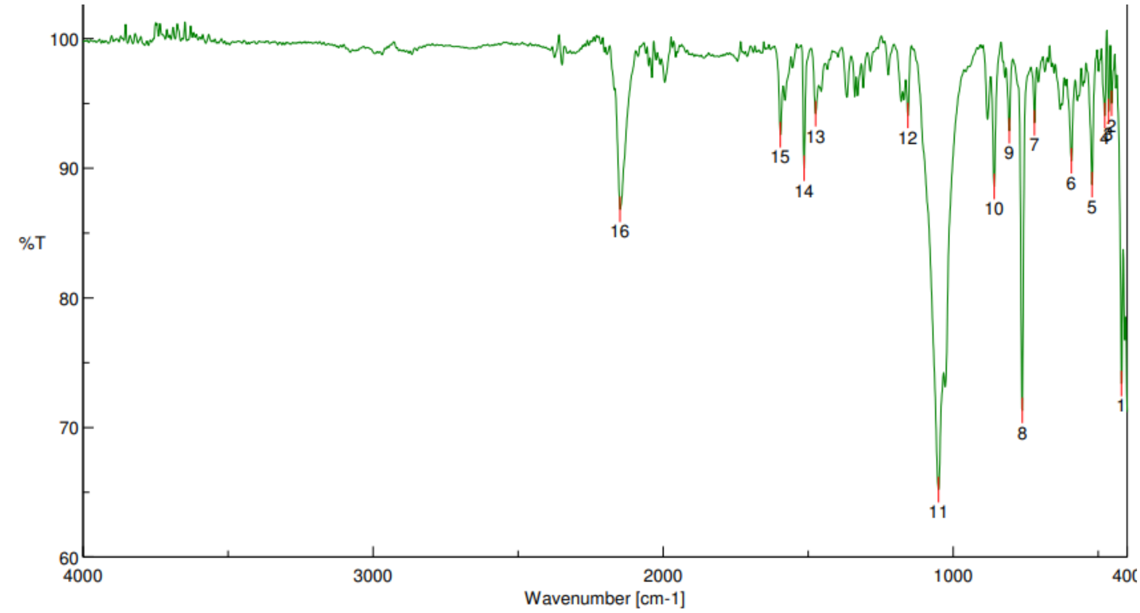

[Result of Peak Picking]

No.	Position	Intensity	No.	Position	Intensity	No.	Position	Intensity	No.	Position	Intensity
1	419.4	73.4	2	453.2	95.0	3	463.8	94.4	4	477.3	94.1
5	520.7	88.7	6	592.0	90.6	7	719.3	93.5	8	761.7	71.3
9	806.1	92.9	10	858.2	88.6	11	1050.1	65.2	12	1156.1	94.1
13	1474.3	94.2	14	1513.9	90.0	15	1595.8	92.6	16	2148.3	86.8

Supplementary Figure 32. IR spectrum of **5-^{13}C**.

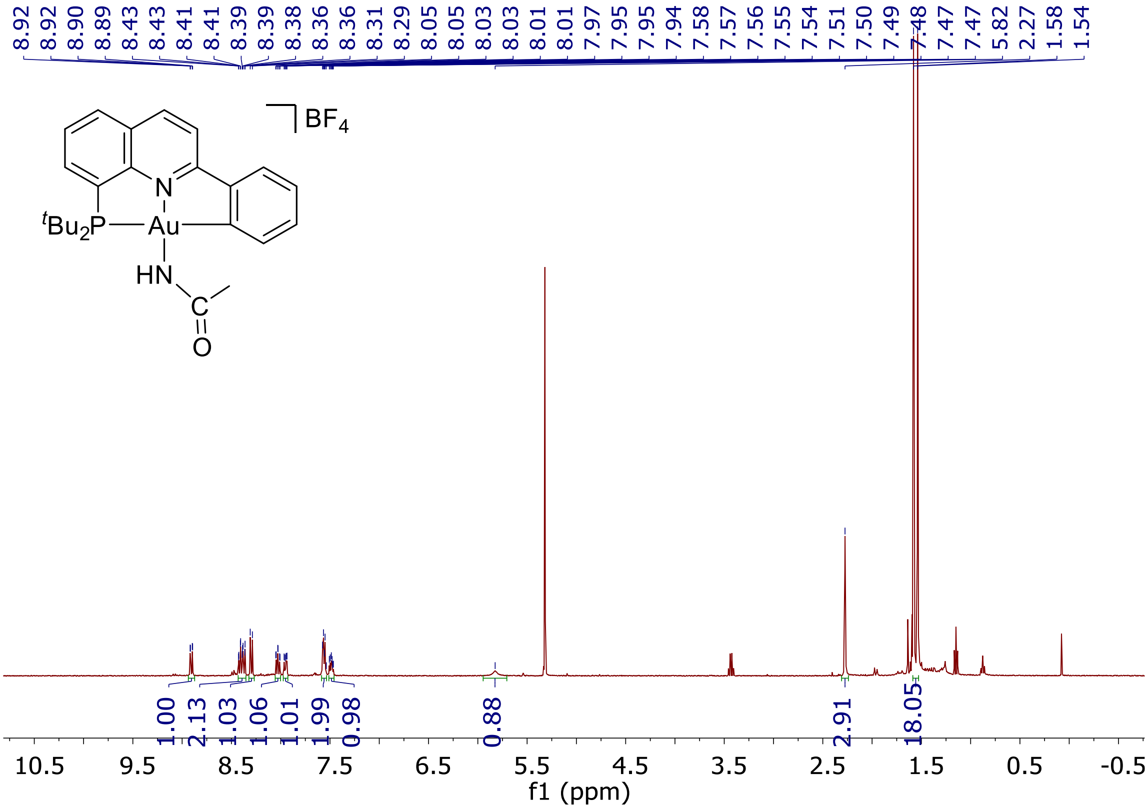

Supplementary Figure 33. ^{1}H NMR spectrum (400.13 MHz, CD_2Cl_2, 298 K) of **6**.

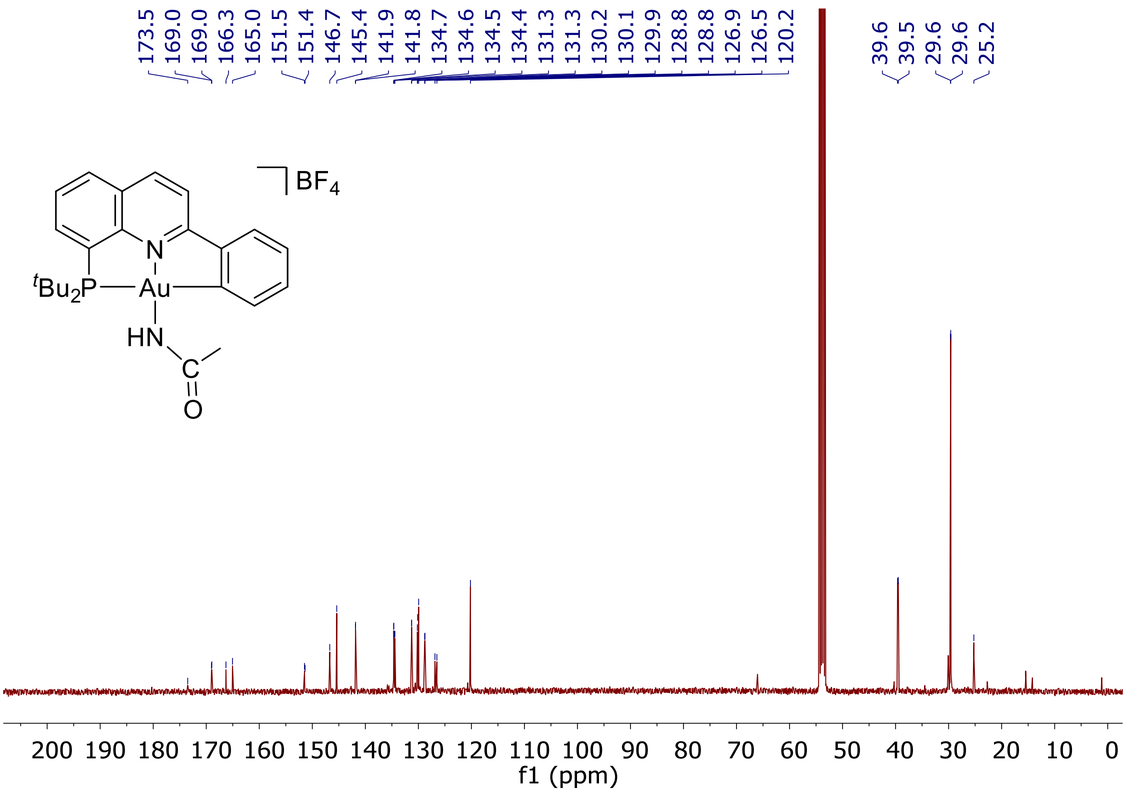

Supplementary Figure 34. $^{13}C\{^{1}H\}$ NMR spectrum (100.62 MHz, CD_2Cl_2, 298 K) of **6**.

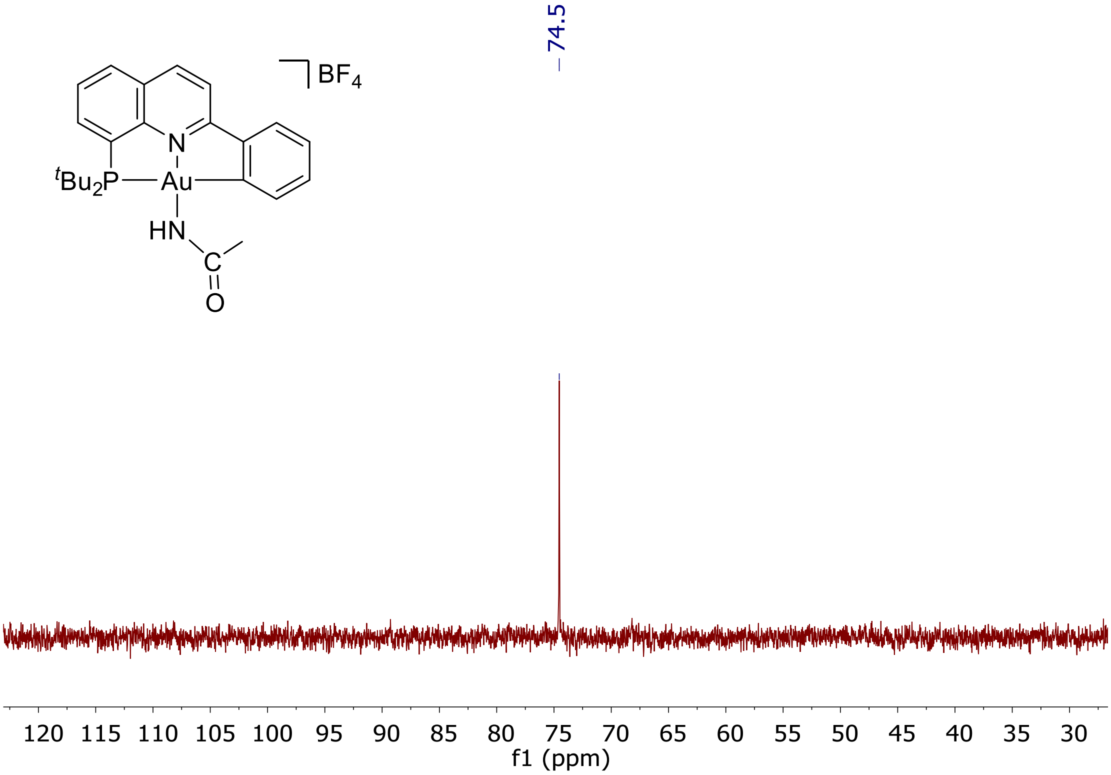

Supplementary Figure 35. $^{31}P\{^{1}H\}$ NMR spectrum (161.99 MHz, CD_2Cl_2, 298 K) of **6**.

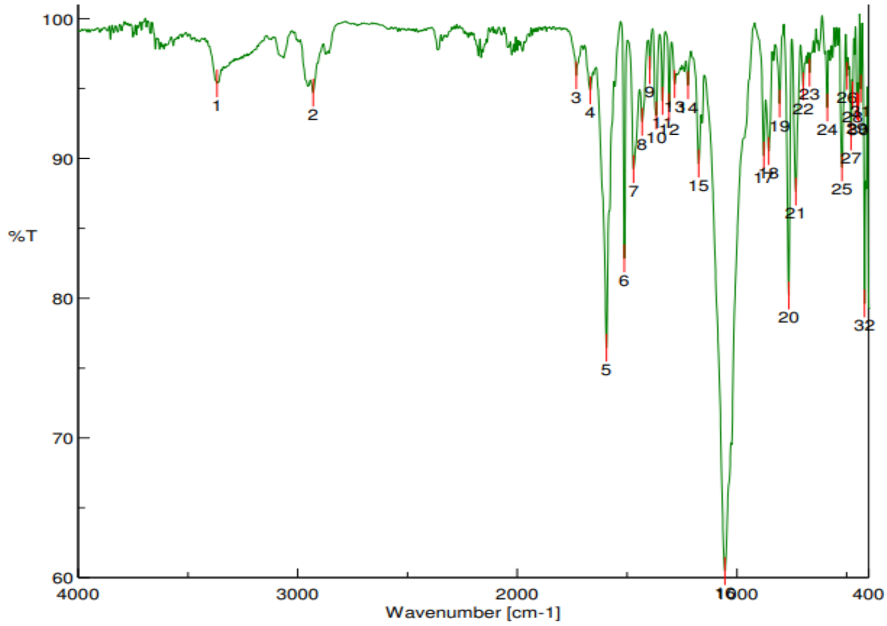

[Result of Peak Picking]

No.	Position	Intensity	No.	Position	Intensity	No.	Position	Intensity
1	3368.1	95.4	2	2929.3	94.7	3	1731.8	95.9
4	1668.1	94.9	5	1593.9	76.4	6	1512.9	82.8
7	1470.5	89.2	8	1430.9	92.6	9	1396.2	96.3
10	1367.3	93.0	11	1338.4	94.1	12	1308.5	93.7
13	1283.4	95.3	14	1222.7	95.2	15	1173.5	89.6
16	1054.9	60.5	17	877.5	90.2	18	855.3	90.5
19	805.1	93.9	20	763.7	80.2	21	731.9	87.6
22	698.1	95.1	23	669.2	96.1	24	588.2	93.6
25	521.7	89.4	26	498.5	95.9	27	480.2	91.6
28	472.5	94.5	29	453.2	93.7	30	446.4	93.6
31	435.8	95.0	32	418.5	79.6			

Supplementary Figure 36. IR spectrum of **6**.

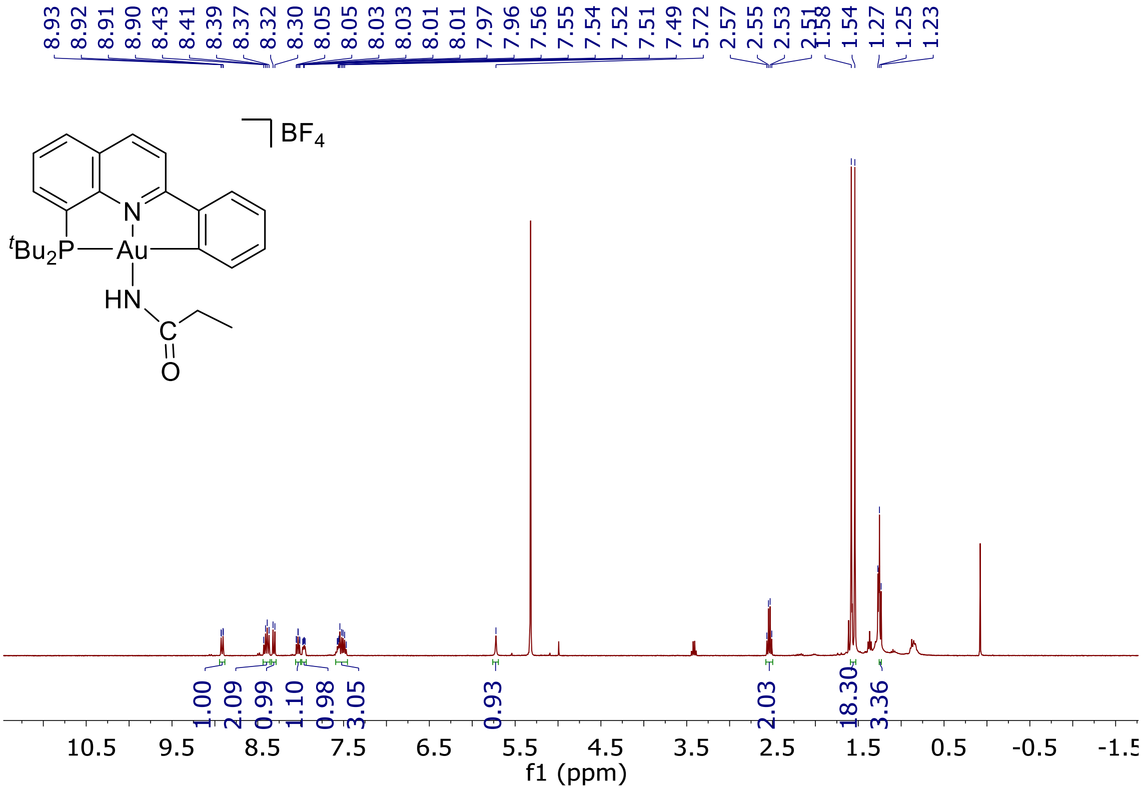

Supplementary Figure 37. ^{1}H NMR spectrum (400.13 MHz, CD_2Cl_2, 298 K) of **7**.

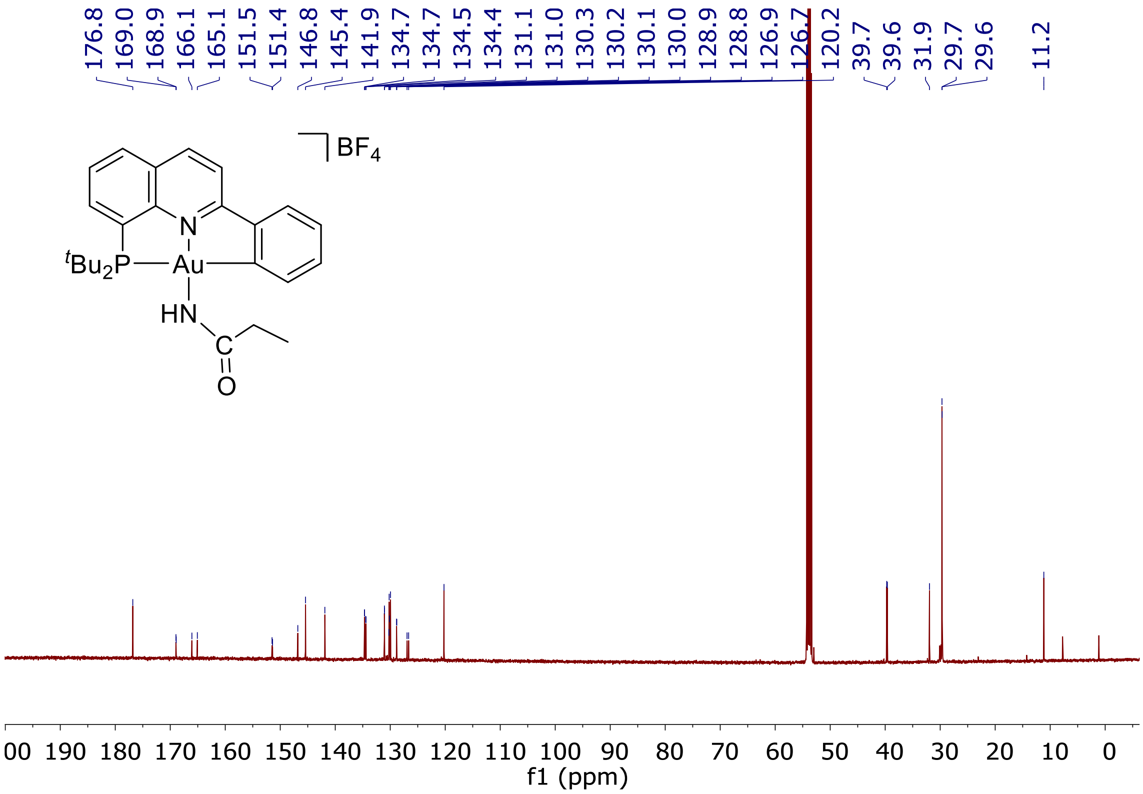

Supplementary Figure 38. $^{13}C\{^{1}H\}$ NMR spectrum (125.81 MHz, CD_2Cl_2, 298 K) of **7**.

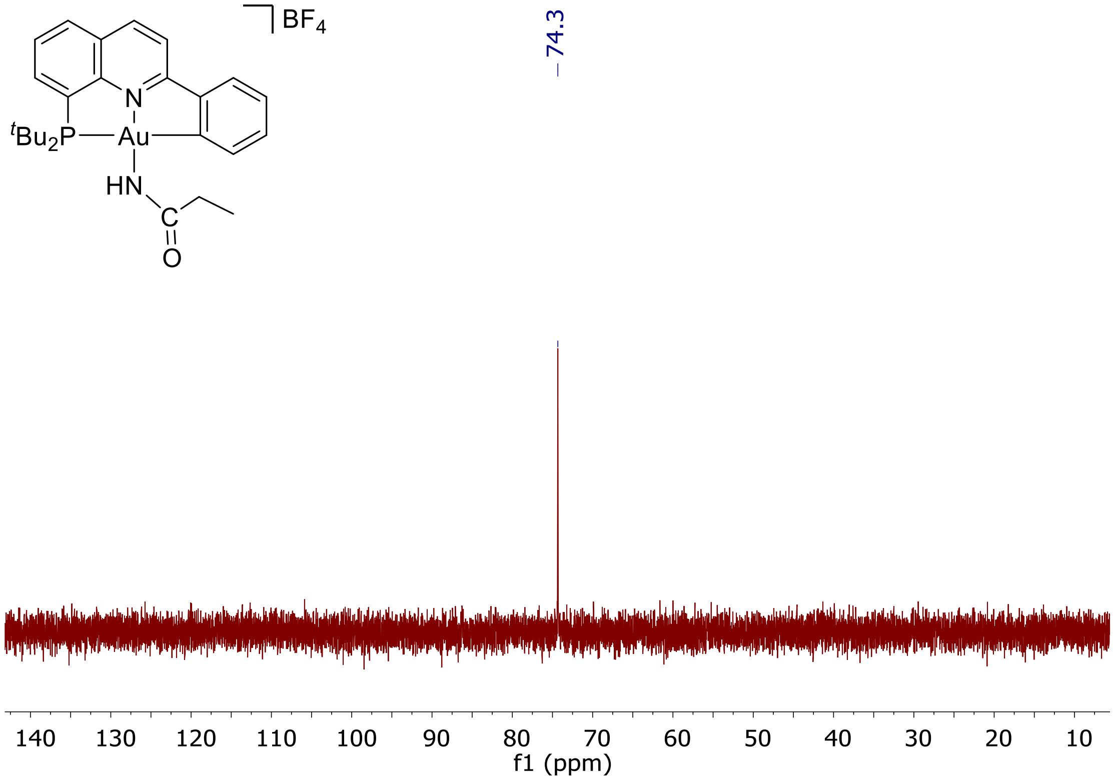

Supplementary Figure 39. $^{31}P\{^{1}H\}$ NMR spectrum (161.99 MHz, CD_2Cl_2, 298 K) of **7**.

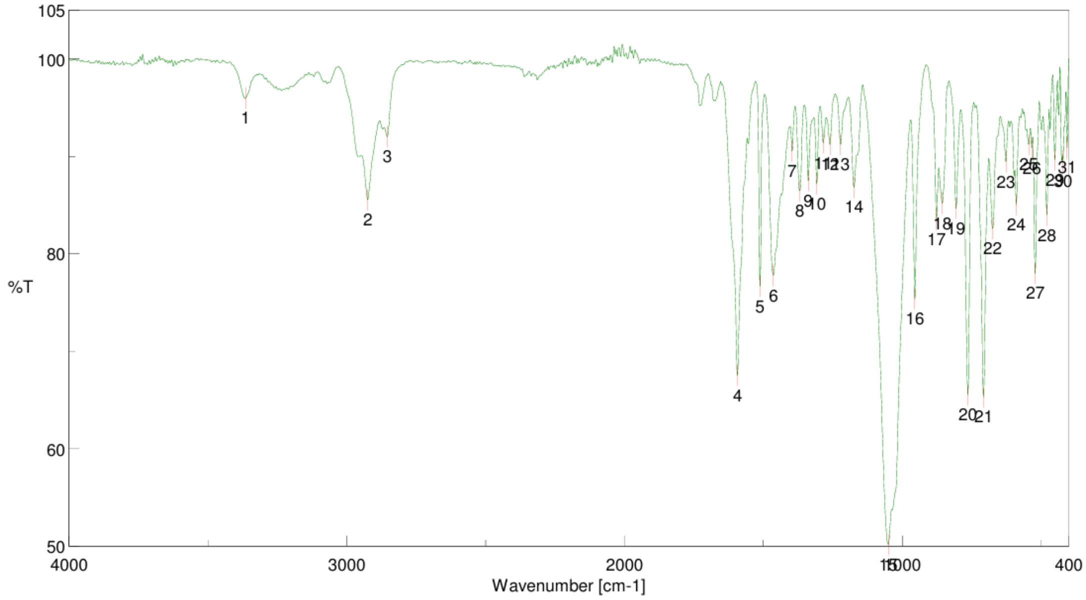

[Result of Peak Picking]

No.	Position	Intensity	No.	Position	Intensity	No.	Position	Intensity	No.	Position	Intensity
1	3365.2	96.0	2	2924.5	85.5	3	2854.1	92.0	4	1592.9	67.5
5	1511.9	76.6	6	1463.7	77.8	7	1396.2	90.5	8	1369.2	86.5
9	1338.4	87.5	10	1307.5	87.2	11	1284.4	91.4	12	1260.3	91.2
13	1221.7	91.2	14	1173.5	86.8	15	1050.1	50.2	16	954.6	75.4
17	876.5	83.5	18	855.3	85.2	19	806.1	84.6	20	763.7	65.5
21	706.8	65.3	22	675.0	82.6	23	626.8	89.4	24	589.2	85.0
25	544.8	91.2	26	533.2	90.8	27	520.7	78.0	28	479.2	84.0
29	450.3	89.6	30	420.4	89.5	31	405.9	90.9			

Supplementary Figure 40. IR spectrum of **7**.

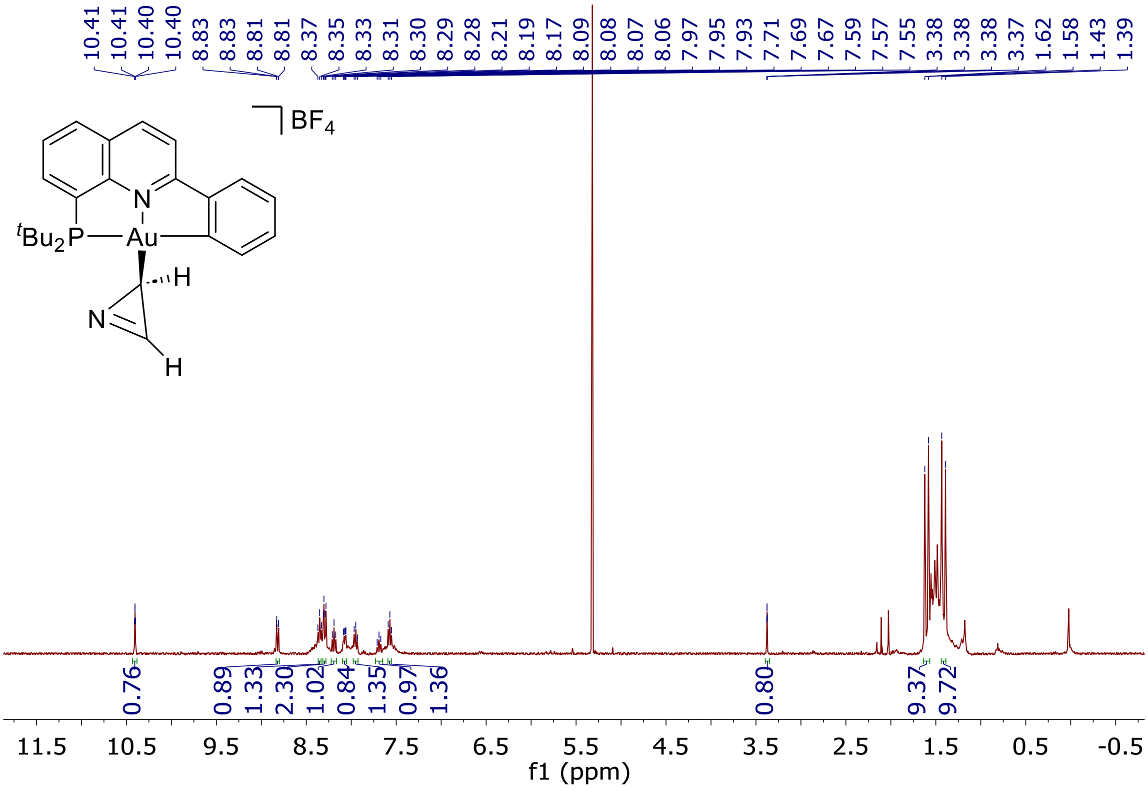

Supplementary Figure 41. ^{1}H NMR spectrum (400.13 MHz, CD_2Cl_2, 220 K) of **8**.

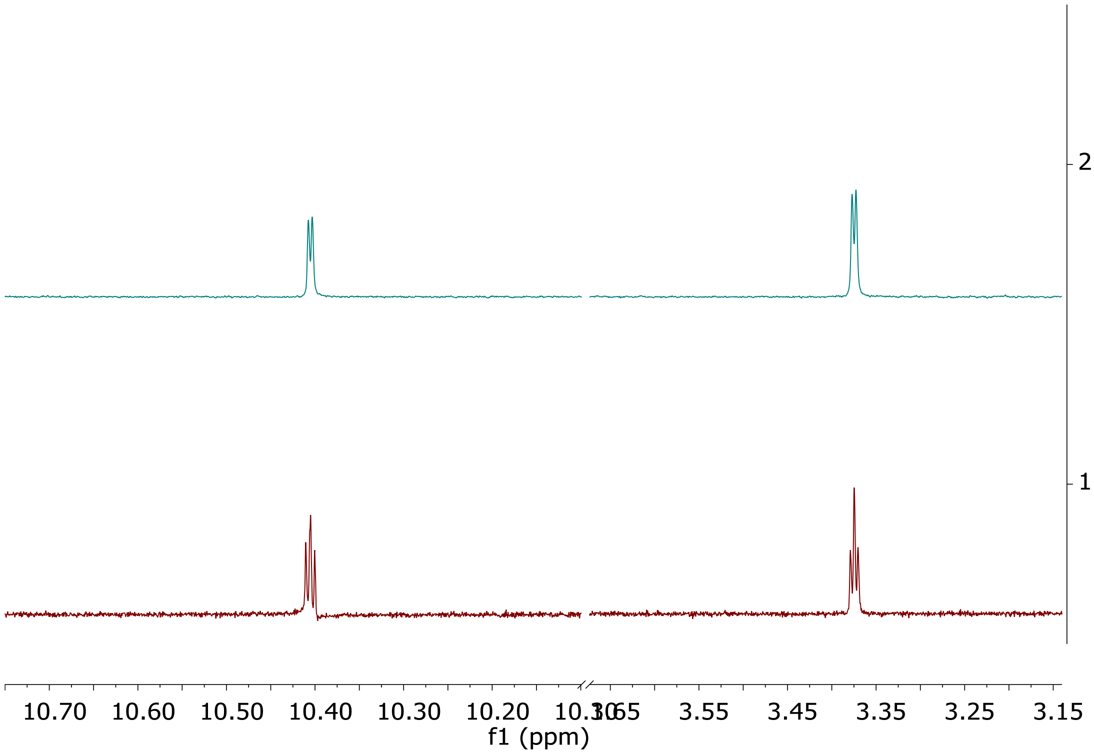

Supplementary Figure 42. H[18] and H[19] region of the $^{1}H\{^{31}P\}$ (top) vs ^{1}H (bottom) NMR spectra (400.13 MHz, CD_2Cl_2, 220 K) of **8**.

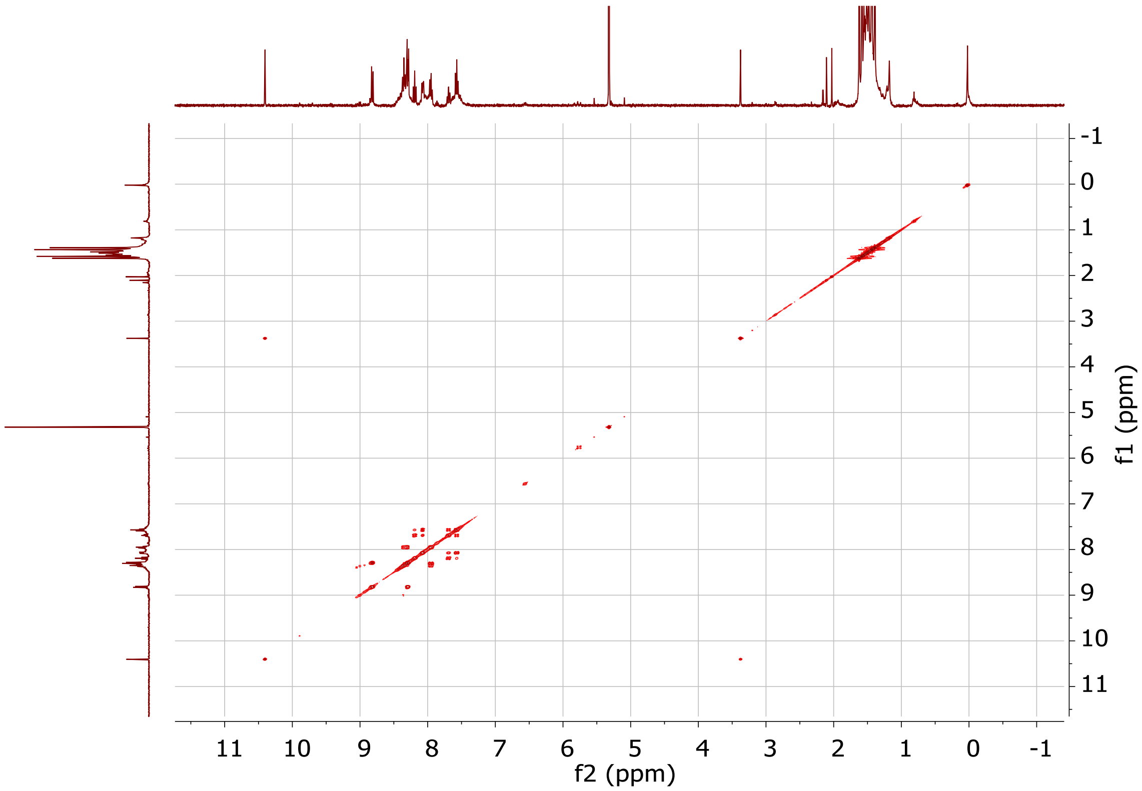

Supplementary Figure 43. ^{1}H-^{1}H COSY NMR spectrum (400.13 MHz, CD_2Cl_2, 220 K) of **8**.

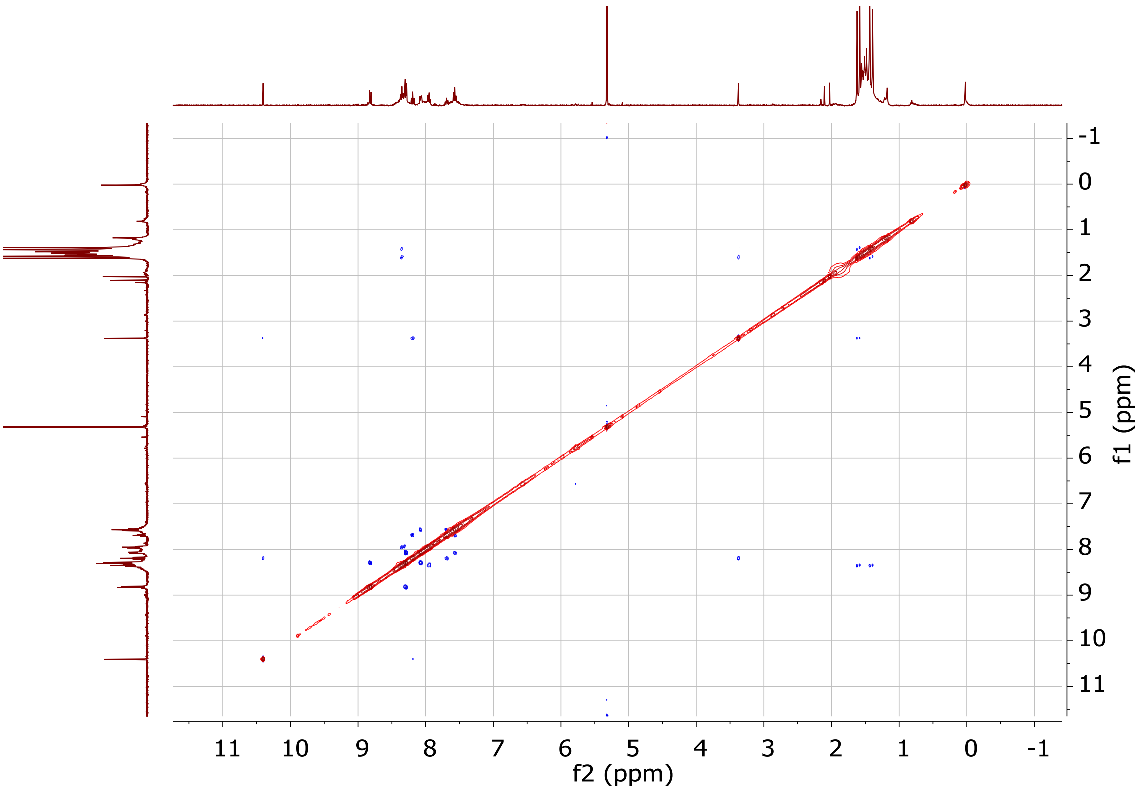

Supplementary Figure 44. ^1H-^1H NOESY NMR spectrum (400.13 MHz, CD_2Cl_2, 220 K) of **8**.

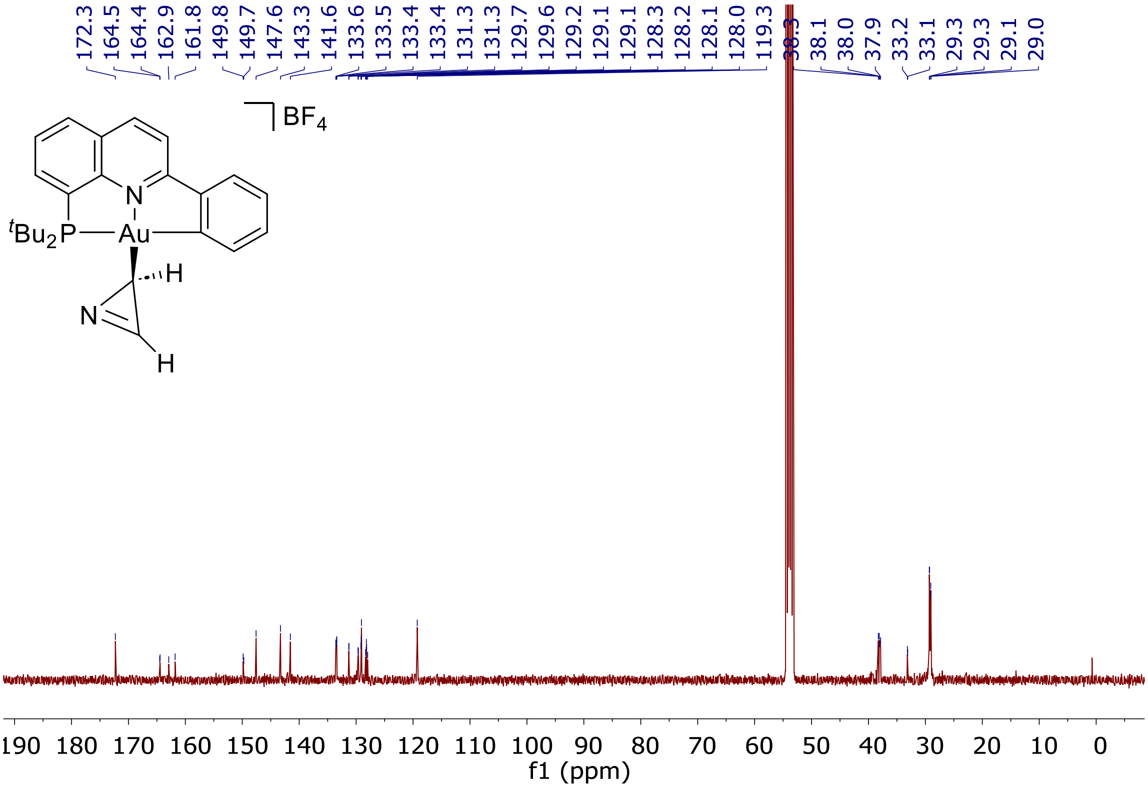

Supplementary Figure 45. $^{13}C\{^1H\}$ NMR spectrum (100.62 MHz, CD_2Cl_2, 220 K) of **8**.

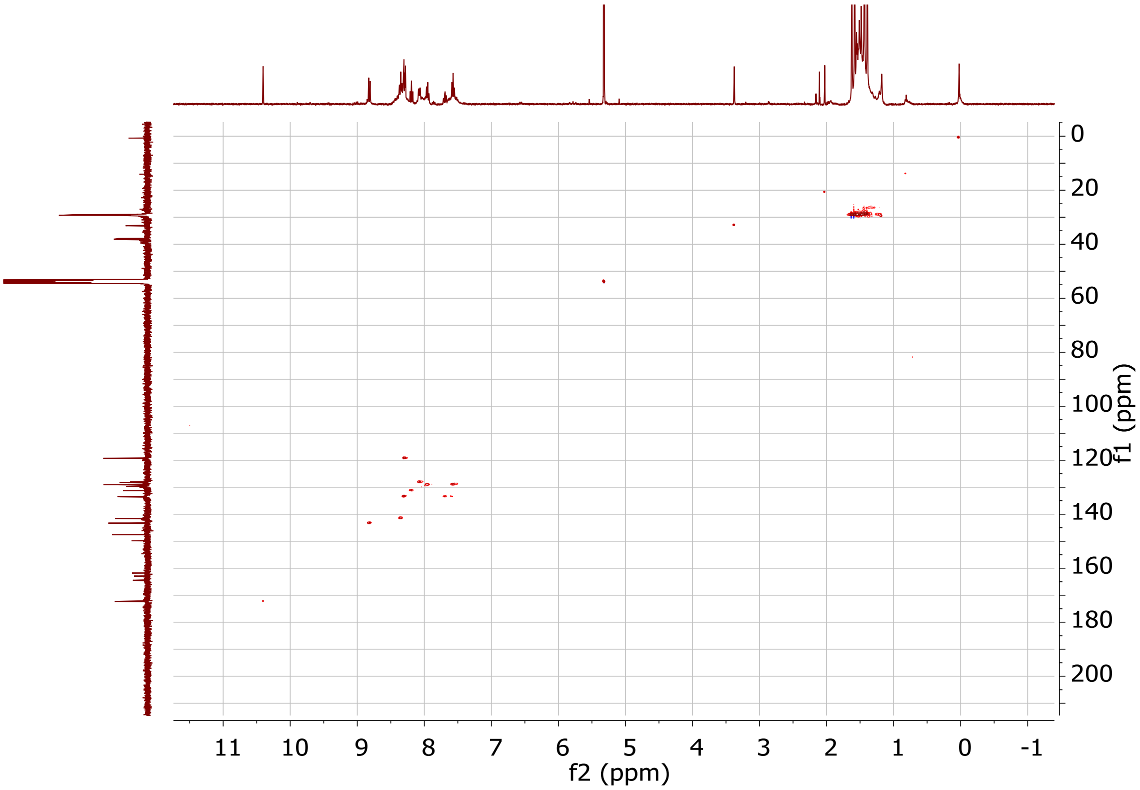

Supplementary Figure 46. ^{1}H-^{13}C HSQC NMR spectrum (400.13 MHz, 100.62 MHz, CD_2Cl_2, 220 K) of **8**.

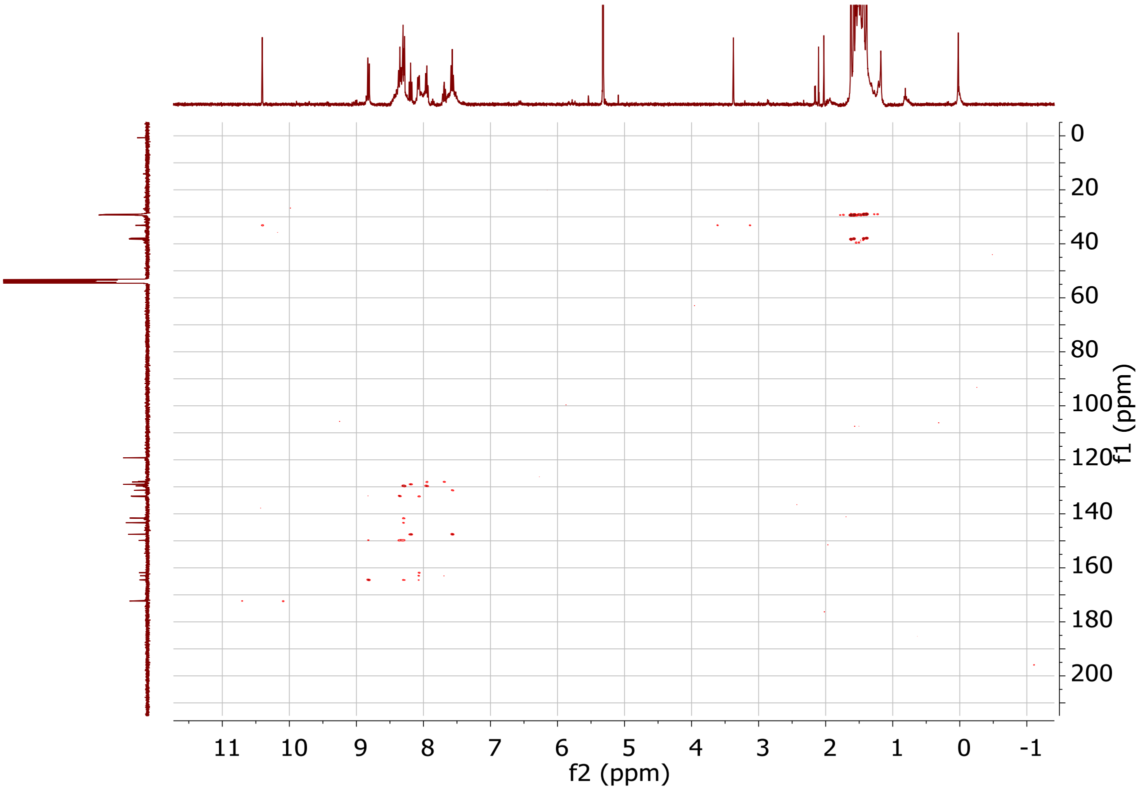

Supplementary Figure 47. ^{1}H-^{13}C HMBC NMR spectrum (400.13 MHz, 100.62 MHz, CD_2Cl_2, 220 K) of **8**.

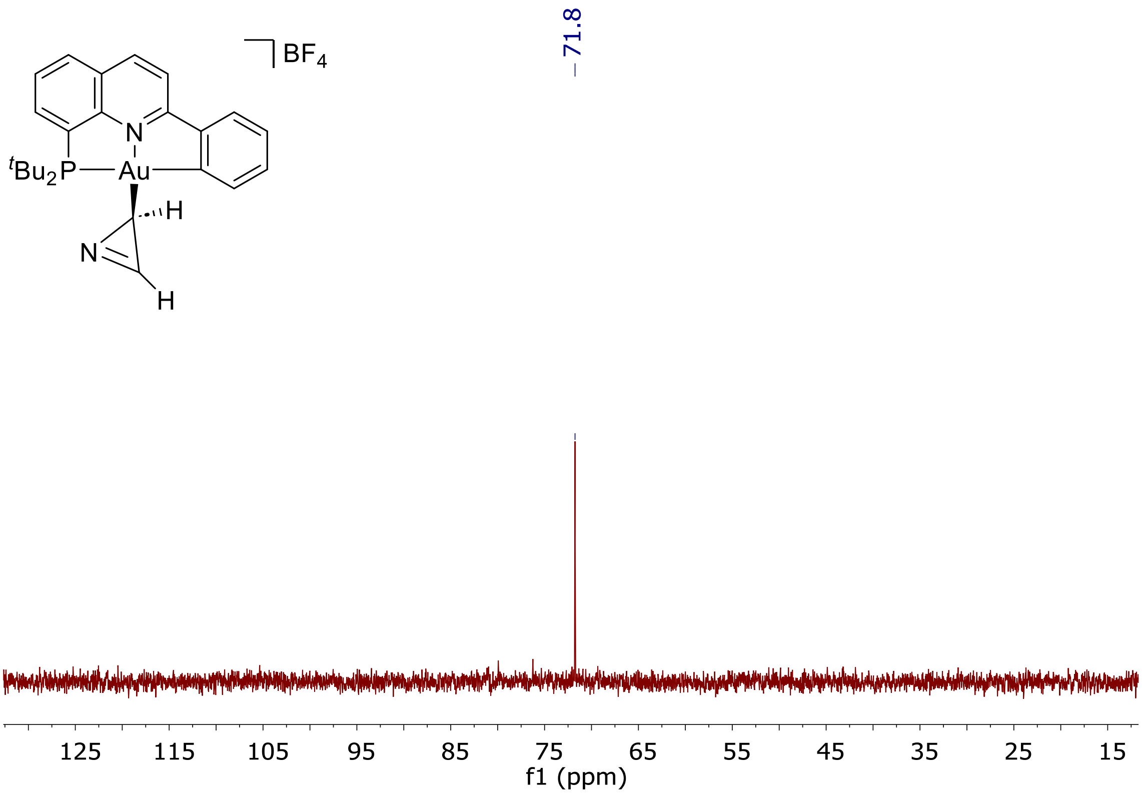

Supplementary Figure 48. $^{31}P\{^{1}H\}$ NMR spectrum (161.99 MHz, CD_2Cl_2, 298 K) of **8**.

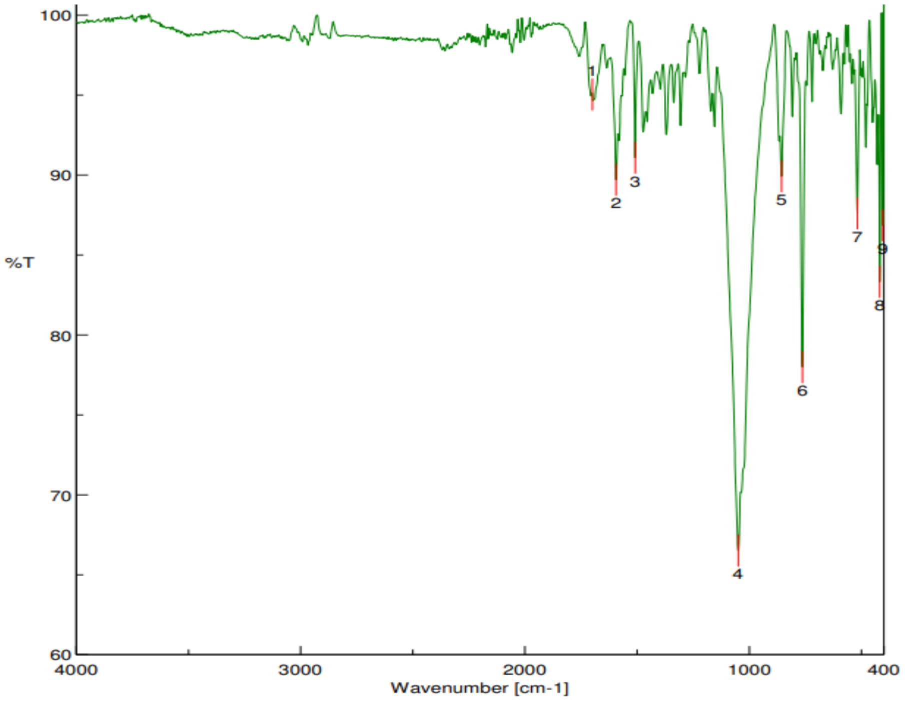

[Result of Peak Picking]

No.	Position	Intensity	No.	Position	Intensity	No.	Position	Intensity
1	1699.9	95.0	2	1593.9	89.7	3	1508.1	91.1
4	1049.1	66.5	5	856.2	89.9	6	763.7	78.0
7	518.8	87.6	8	418.5	83.3	9	405.0	86.8

Supplementary Figure 49. IR spectrum of **8**.

4 Mass spectrometric analysis

MS/MS spectra were acquired in a TIMS-QTOF-MS instrument (*Bruker Daltonics* GmbH, Bremen, Germany) at collision energy of 20 eV with a 4 *m/z* isolation width. Nitrogen was used as a collision gas. The fragment of compound **1-^{15}N** was initially detected by HRMS-ESI (*m/z* 588.16021, calcd. 588.16039). Subsequent MS/MS of this ion (Supplementary Figure 50) led to the formation of gold nitrene species **2** (*m/z* 559.15704, calcd. 559.15721) and **2-^{15}N** (*m/z* 560.15531, calcd. 560.15424) after the loss of ^{15}N≡^{14}N and ^{14}N≡^{14}N, respectively.

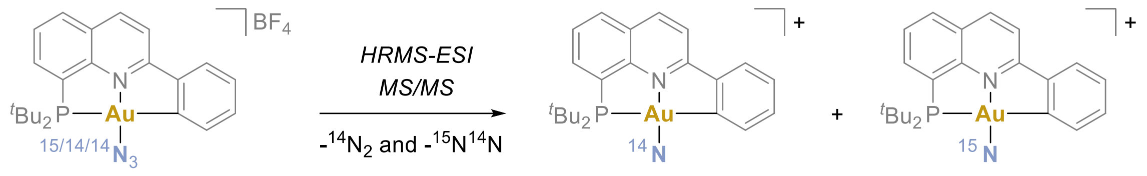

1-^{15}N **2** **2-^{15}N**

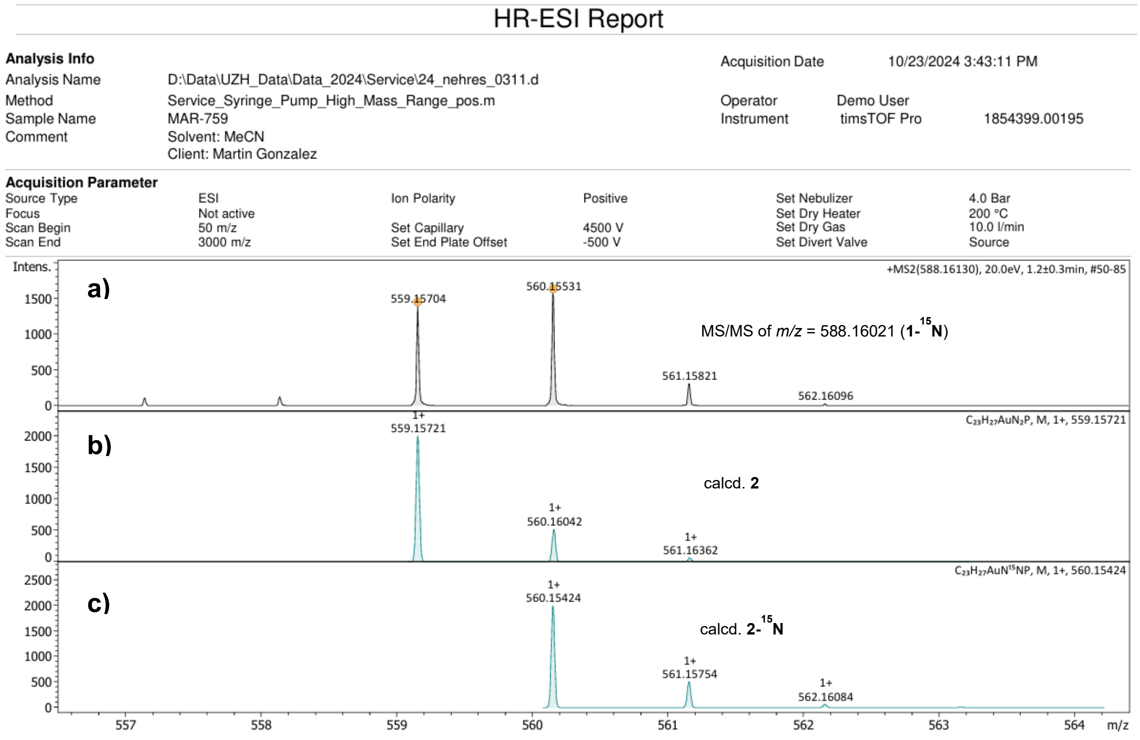

Supplementary Figure 50. a) HRMS-ESI, MS/MS spectra of compound **1-^{15}N** (*m/z* = 588.16021). b) Simulated MS spectrum for **2** (*m/z* = 559.15721). c) Simulated MS spectrum for **2-^{15}N** (*m/z* = 560.15424).

5 Photocrystallographic characterization

In crystallo experiments were conducted to characterize the structure of the gold-nitrene by X-ray diffraction analysis induced by light. The data was collected using synchrotron radiation (λ = 0.65255 Å) at the Swiss Light Source located at the Paul Scherrer Institute (PSI).

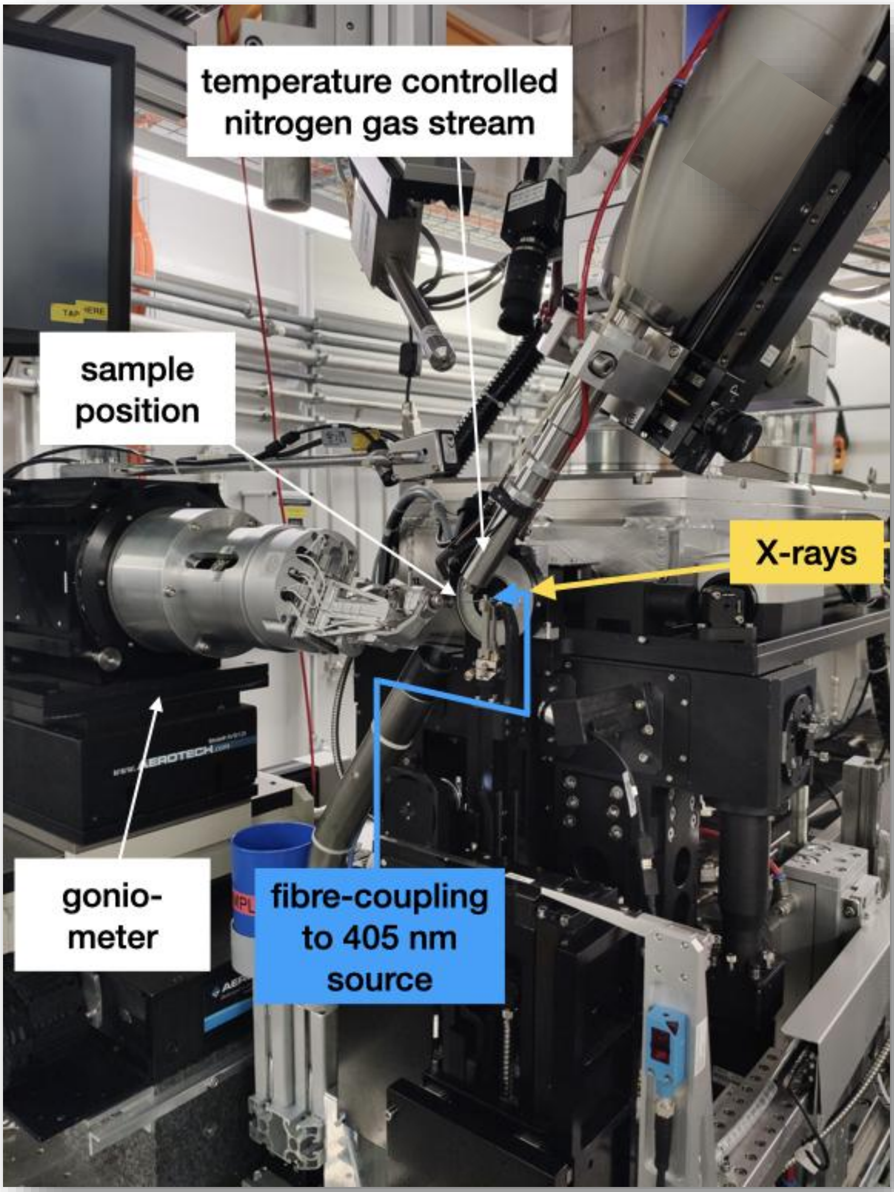

Supplementary Figure 51. Set-up of the X-ray photocrystallography experiment in the synchrotron using a 405 nm laser.

New crystals were used for each experiment to prevent sample degradation. A single crystal (~120 × 120 × 600 µm) was looped in a cryo-mount in silicon oil and then plunge-cooled in liquid nitrogen. The crystal was mounted on beamline PX II of the Swiss Light

Source (SLS) under a constant flow of nitrogen gas at 160 K. After extensive optimization of the conditions, the following parameters were identified as optimal for the characterization of compound **2**. The crystal was then illuminated for 2 min by a 405 nm, fibre-couple CW laser (Omicron Laserage LDM405.400.CWA) focused to 150 μm (FWHM).[4] To ensure uniform exposure to light, the face of the crystal was illuminated for 1 min and then the crystal was rotated 180° and the back face was illuminated for 1 min. The power of the laser was measured at 1.06 mW, with a power density of 12 W/cm^2. Higher power values led to crystal decomposition even with exposure times under 1 minute.

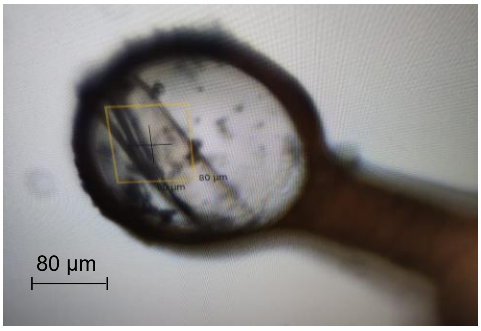

Supplementary Figure 52. Microscopy image of a single crystal looped in a cryo-mount.

After the 405 nm illumination, the laser was switched off, and the crystal was then exposed with 0.65255 Å X-rays with a focus of 20 × 75 μm (FWHM) and attenuated by 75% giving an approximate flux of 1.5 × 10^{12} photons s^{-1}. 360 diffraction images were recorded with 10 ms exposure using an EIGER2 16 Mpixel detector (Dectris) with a total rotation of 180° (0.5° per image). The data were reduced using the DIALS software suit[5] and phased using SHELXT.[6]

6 *In crystallo* activation of O_2

General procedure. A 4 mL vial was charged with ca. 1–2 mg of (P^N^C)Au-N_3 in crystalline form and closed with a screw cap equipped with a septum. The vial atmosphere was evacuated, and O_2 (1 atmosphere) was introduced. The vial was sealed with electric tape, placed in a photoreactor and irradiated. After the selected period of time, a monocrystal was measured by X-ray diffraction analysis to determine the conversion of the reaction. Subsequently, the reaction was characterized using NMR spectroscopy, followed by HRMS analysis.

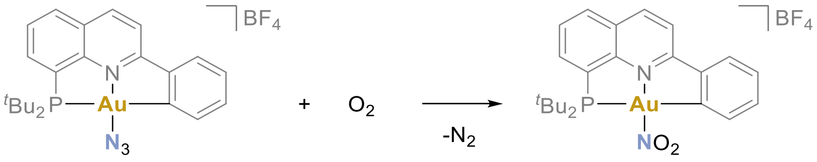

1 **3**

Supplementary Table 1. Optimization of the O_2 activation by the photogenerated gold-nitrene **2** in crystallo.

Entry	Light	time, T	Ratio **1** : **3**
1	White	40 h, RT	70 : 30
2	467 nm	16 h, RT	npd*
3	White	72 h, 5 °C	50 : 50
4	White	102 h, 5 °C	npd*
5	No	7 d, 5 °C	100: 0

*npd: not possible to determine

<u>Counterion</u>. The effect of counterions on sc-t-sc transformations has been shown to be significant. Among them, BAr^F stands out, as it has been reported to be crucial for retaining crystallinity during sc-t-sc reactions.[7,8] To explore the influence of other counterions in the reactions *in crystallo*, BF_4 was replaced with BAr^F counterion using $NaBAr^F$ (BAr^F = Tetrakis(3,5-bis(trifluoromethyl)phenyl)borate). Unfortunately, while high-quality monocrystals of **1** with BF_4 can be obtained from various solvent mixtures, complex **1-BAr^F** did not yield crystals of sufficient quality for X-ray diffraction analysis.

<u>In solution</u>. We sought to replicate this sc-t-sc reaction under controlled conditions in solution. To this end, azide complex **1** and **1-BAr^F** were dissolved in THF, toluene and dichloromethane, and then irradiated with various wavelengths (390, 427, 467, 525 nm) or white light in the presence of O_2 (1 bar, 99.999%) at temperatures from −40 to 60 °C for periods spanning from minutes to days. None of these attempts resulted in the

formation of significant amounts of complex **3** in the reaction media, and azide decomposition was observed, resulting in an untraceable mixture of signals.

Light source and temperature. Kessil lamps with different wavelengths were tested for the *in crystallo* experiments. However, most experiments resulted in a significant loss of crystallinity. To mitigate this, commercial white LEDs bulbs (Philips 60 W, 4000 K, (Supplementary Figure 53) were used without the external glass (Supplementary Figure 54a). Additionally, placing the photoreactor in a cold room at 5 °C further helped preserve the crystallinity of the samples (Supplementary Figure 54b).

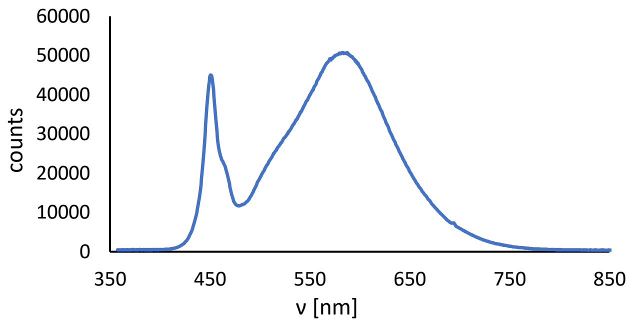

Supplementary Figure 53. Emission spectrum of the white LEDs used in this project.

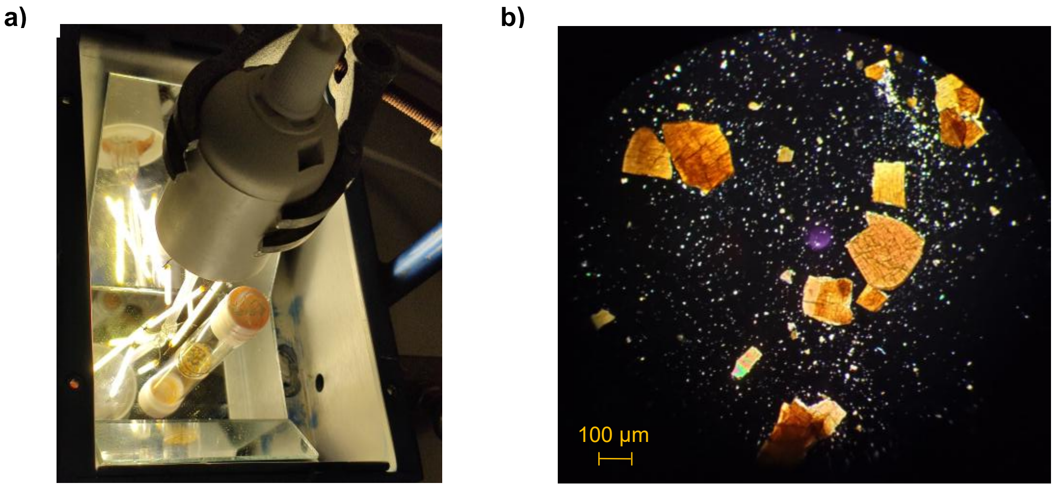

Supplementary Figure 54. a) Vial containing crystals of **1** under O_2 in a photoreactor with white LEDs. b) Microscope image of crystals after irradiation.

Power density of the light. The light power was measured using a S310C thermal sensor (*Thorlabs*, Germany). The light source was placed at 1.8 cm from the sensor, just before adjusting the background to 0 W/cm^2.

- White LEDs = 0.05 W/cm^2
- Kessil lamp 525 nm = 0.40 W/cm^2

7 Solid-state reactions

Unless otherwise specified, the optimized conditions found for the reactions *in crystallo* (white LEDs, 5 °C) were used in solid state.

General procedure with gaseous reagents. A 4 mL vial was charged with ca. 7 mg of (P^N^C)Au-N_3 in powder form and closed with a screw cap equipped with a septum (Supplementary Figure 55a). The vial atmosphere was evacuated, and the desired gas (1 atm) was introduced through the septum. The vial was sealed with electric tape, placed in a photoreactor and exposed to white light at 5 °C. The reaction was monitored over time using solid-state IR spectroscopy. The vial atmosphere was replenished with fresh gas daily until the reaction was complete. Completion was confirmed by the disappearance of the strong azide band 2048 cm^{-1} in the IR spectra. Subsequently, the product was fully characterized using NMR spectroscopy, followed by HRMS analysis. Crystallization was then attempted in order to obtain suitable crystals for X-ray diffraction analysis.

General procedure with liquid reagents. A 4 mL vial containing 7 mg of (P^N^C)Au-N_3 in powder form was placed uncapped inside of a 20 mL vial, which was closed with a screw cap equipped with a septum (Supplementary Figure 55b). The system was evacuated, and the reagent in liquid phase (ca. 2 mL) was added to the outer vial. The sealed system was placed in a photoreactor and exposed to white light at 5 °C. The reaction was monitored over time using solid-state IR spectroscopy. Completion was confirmed by the disappearance of the strong azide band 2048 cm^{-1} in the IR spectra. Subsequently, the product was dried under vacuum and characterized by NMR spectroscopy, followed by HRMS analysis. Crystallization was then attempted in order to obtain suitable crystals for X-ray diffraction analysis.

a)

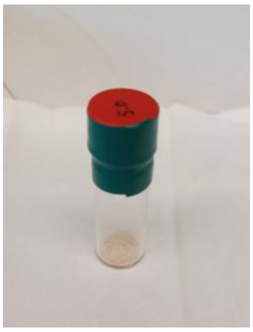

b)

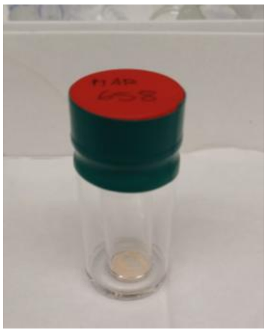

Supplementary Figure 55. a) Vial containing powder of **1** under the gas of choice, sealed with electric tape. b) Double-vial system with powder of **1** in the inner vial and the liquid reagent in the outer vial.

- **Molecular Oxygen**

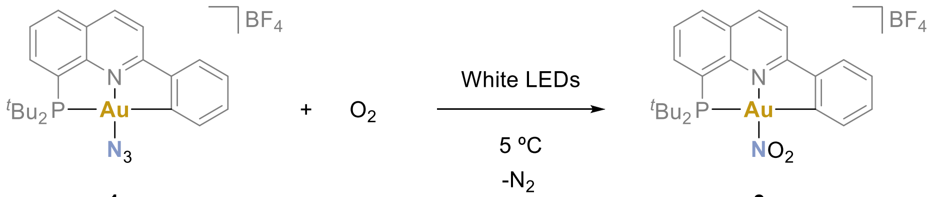

1 **3**

Supplementary Table 2. Optimization of the O_2 activation by the photogenerated gold-nitrene **2** in solid state.

Entry	Conditions	Ratio **1** : **3**
1	72 h	30 : 70
2	air instead of pure O_2, 72 h	50 : 50
3	72 h, fresh O_2 refilled every 24 h	10 : 90
4	72 h, no light	100 : 0

- **Water**

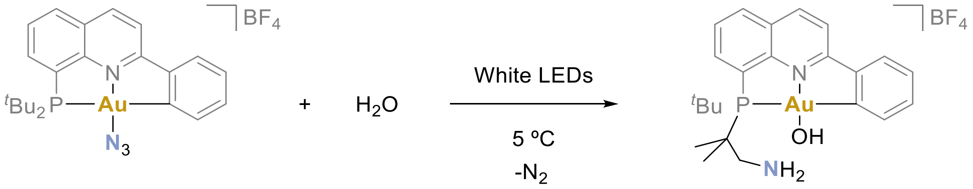

1 **4**

Supplementary Table 3. Optimization of the reaction of water with the photogenerated gold-nitrene **2** in solid state.

Entry	Conditions	Ratio **1** : **3*** : **4**
1	wet nitrogen, 102 h	30 : 20 : 50
2	72 h, no light	100 : 0

*Compound **3** was found in the mixture associated with the presence of oxygen traces in the system.

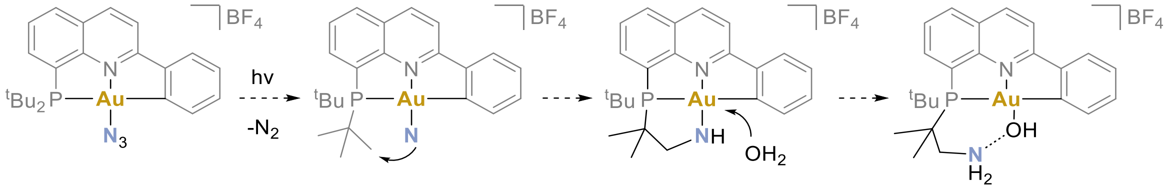

1 **4**

Supplementary Scheme 1. Hypothesis for the formation of **4** in the presence of water.

- **Carbon Monoxide**

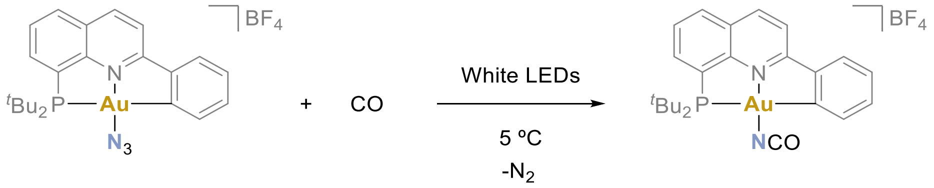

1 5

Supplementary Table 4. Optimization of the CO insertion by the photogenerated gold-nitrene **2** in solid state.

Entry	Conditions	Ratio **1** : **5**
1	72 h, fresh CO refilled every 24 h	0 : 100
2	72 h, no light	100 : 0
3	72 h, no light, 130 °C	90 : 10
4	24 h, 525 nm Kessil lamp	35 : 65

- **Acetaldehyde/Propionaldehyde**

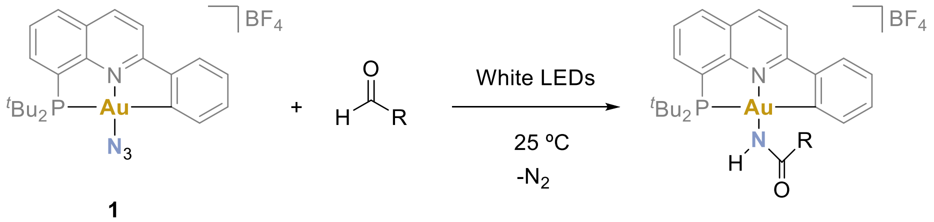

1

6, R : Me
7, R : Et

Supplementary Table 5. Optimization of the insertion of aldehydes into the photogenerated gold-nitrene **2** in solid state.

Entry	Conditions	Ratio **1** : **xx**
1	acetaldehyde, 7 d, 25 °C	30 : 70 (**6**)
2	propionaldehyde, 7 d, 25 °C	40 : 60 (**7**)
3	aldehyde, no light	100 : 0

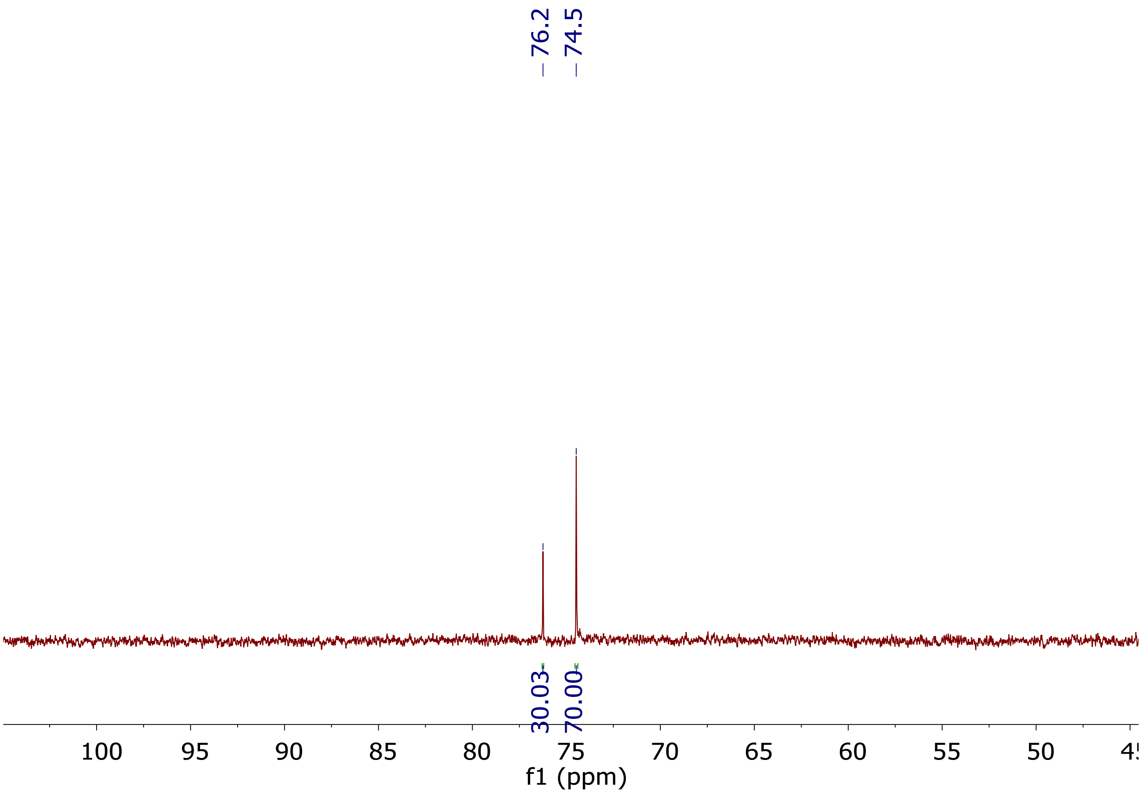

Supplementary Figure 56. $^{31}P\{^{1}H\}$ NMR spectrum (161.99 MHz, CD_2Cl_2, 298 K) corresponding to entry 1, Supplementary Table 5, with a mixture of **1** (76.2 ppm) and **6** (74.5 ppm).

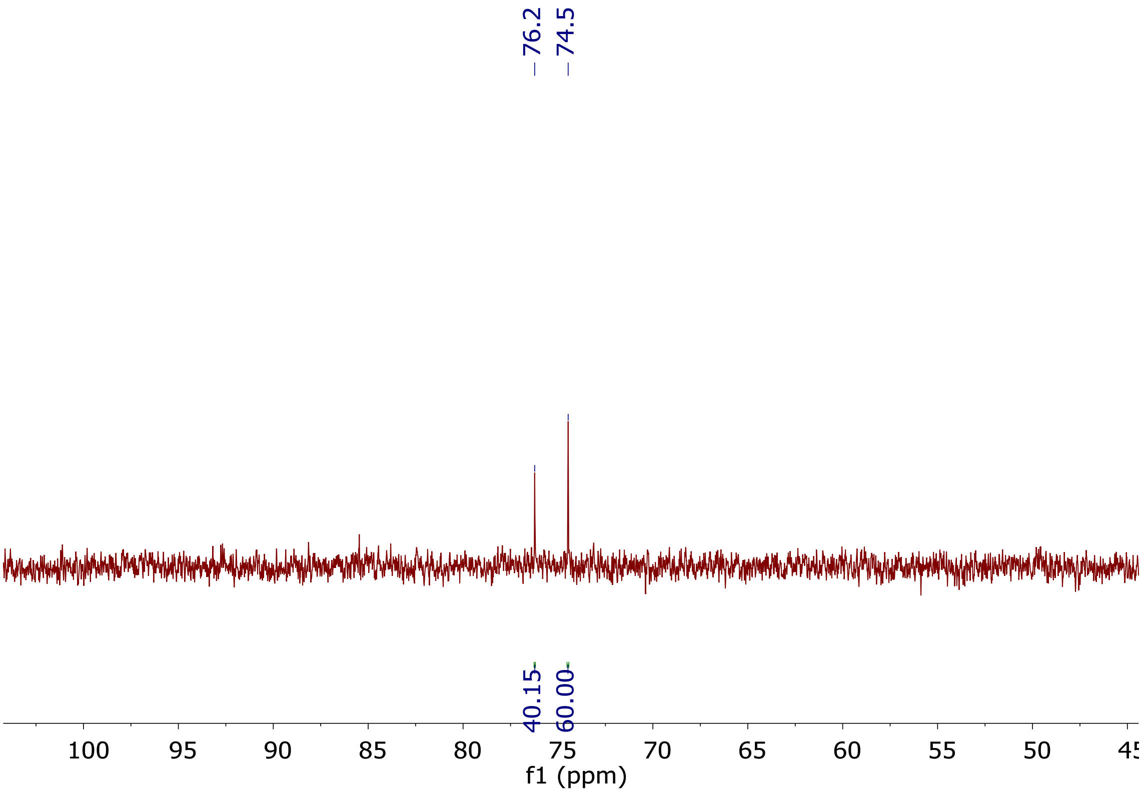

Supplementary Figure 57. $^{31}P\{^{1}H\}$ NMR spectrum (161.99 MHz, CD_2Cl_2, 298 K) corresponding to entry 2, Supplementary Table 5, with a mixture of **1** (76.2 ppm) and **7** (74.5 ppm).

- **Acetylene**

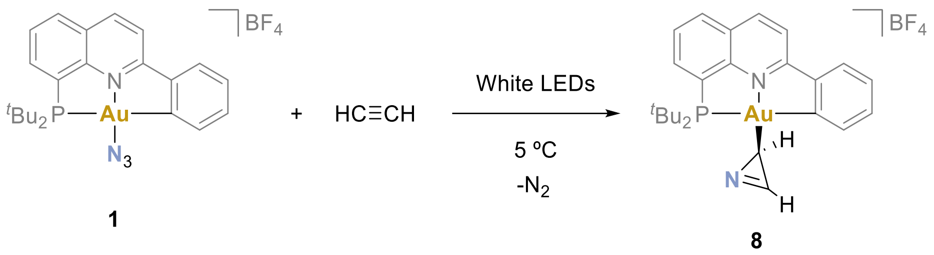

Supplementary Table 6. Optimization of the acetylene addition/skeletal rearrangement by the photogenerated gold-nitrene **2** in solid state.

Entry	Conditions	Ratio **1** : **8**
1	7 d, fresh acetylene refilled every 24 h	0 : 100
2	72 h, no light	100 : 0

Unsuccessful reagents: ethene, 1,3-butadiene, 2,3-dimethyl-1,3-butadiene, methyl acrylate and methyl vinyl ketone.

8 X-ray diffraction analyses

The crystallographic data is presented in the order shown below. The solvent pairs used for crystal growth are listed as solvent and antisolvent, respectively.

Synchrotron experiments

- Compound **1** (Dichloromethane / Pentane): x974_1 (CCDC 2449336)
- Compound **2**: x974_2 (CCDC 2449337)

SC-t-SC activation of O_2

Spontaneous reaction:

- Compound **1** (Dichloromethane / Hexane): NV2111 (CCDC 2449338)
- Compound **3**: MAR220601_019_2_HP (CCDC 2449339)

Controlled conditions:

- Compound **1** (Dichloromethane / Cyclopentane): MAR220913_323_dcm_cp (CCDC 2449341)
- Mixture of **1** and **3** (70 : 30): MAR382_sctsc (CCDC 2449342)
- Mixture of **1** and **3** (50 : 50): MAR392_sctsc_221110 (CCDC 2449343)

Solid-state reactions

- Mixture of **1** and **3** (25 :75) recrystallized in Dichloromethane / Pentane: MAR-574_new (CCDC 2449344)
- Compound **4** (Dichloromethane/ Cyclopentane): mar-231020_591 (CCDC 2449345)
- Compound **5** (Dichloromethane / Cyclopentane): mar_231110_584_dcm_cp_hp (CCDC 2449346)
- Compound **6** (Acetonitrile / Pentane): NV2118 (CCDC 2449347)

Compound **1** exhibited crystal polymorphism,[9] depending on the solvent mixture used: DCM/pentane, DCM/hexane, or DCM/cyclopentane. The *in crystallo* reactivity for the activation of O_2 remained unaffected and the same conclusions can be extracted from these experiments regardless of the crystal form used.

Various A- and/or B-level alerts were observed during crystal structure refinement, most of which arise from synchrotron data collection settings and single-crystal-to-single-crystal transformations. The alerts in CCDC 2449336, CCDC 2449337, CCDC 2449339, CCDC 2449345, CCDC 2449346 and CCDC 2449347 have been addressed in the corresponding CheckCIF report.

- Compound **1** (Dichloromethane / Pentane): x974_1 (CCDC 2449336)

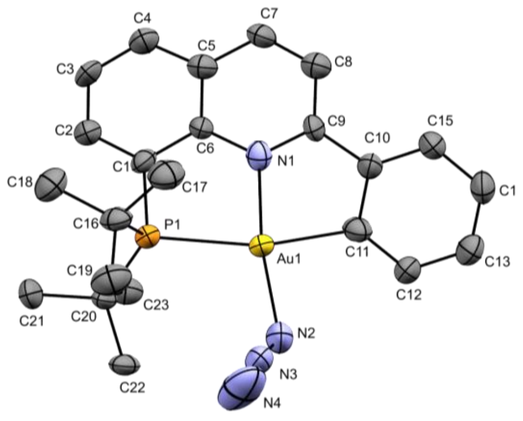

Supplementary Figure 58. Structure of compound **1**. The asymmetric unit contains two independent molecules, only one is shown for clarity. The ellipsoids are drawn at 50 % probability.

Supplementary Table 7. Crystal data and structure refinement for x974_1.

Identification code	x974_1
Empirical formula	$C_{46}H_{54}Au_2B_2F_8N_8P_2$
Formula weight	1348.46
Temperature/K	160.15
Crystal system	monoclinic
Space group	$P2_1/n$
a/Å	14.45176(9)
b/Å	13.39796(10)
c/Å	25.1742(3)
α/°	90
β/°	93.3018(7)
γ/°	90
Volume/Å^3	4866.24(7)
Z	4
ρ_{calc}g/cm^3	1.841
μ/mm^{-1}	4.953
F(000)	2624.0
Crystal size/mm^3	0.35 × 0.05 × 0.04
Radiation	synchrotron (λ = 0.65255)
2Θ range for data collection/°	2.914 to 46.296
Index ranges	-17 ≤ h ≤ 17, -15 ≤ k ≤ 15, -30 ≤ l ≤ 30
Reflections collected	29503
Independent reflections	8661 [R_{int} = 0.0873, R_{sigma} = 0.0736]
Data/restraints/parameters	8661/0/625
Goodness-of-fit on F^2	0.977
Final R indexes [I>=2σ (I)]	R_1 = 0.0564, wR_2 = 0.1392
Final R indexes [all data]	R_1 = 0.0742, wR_2 = 0.1519
Largest diff. peak/hole / e Å^{-3}	3.42/-4.12

- Compound **2**: x974_2 (CCDC 2449337)

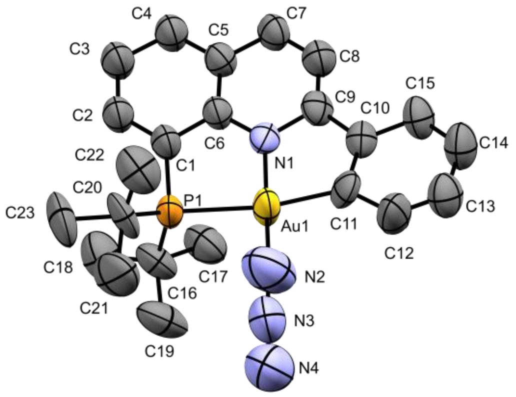

Supplementary Figure 59. Structure of compounds **1** and **2** (70 : 30 mixture). The asymmetric unit contains two independent molecules, only one is shown for clarity. The ellipsoids are drawn at 50 % probability.

Supplementary Table 8. Crystal data and structure refinement for x974_2.

Identification code	x974_2_1060uw_2min
Empirical formula	$C_{23}H_{27}AuBF_4N_{3.4}P$
Formula weight	665.82
Temperature/K	160.15
Crystal system	monoclinic
Space group	$P2_1/n$
a/Å	14.969(8)
b/Å	13.382(5)
c/Å	24.588(10)
α/°	90
β/°	92.162(13)
γ/°	90
Volume/Å^3	4922(4)
Z	8
ρ_{calc}g/cm^3	1.797
μ/mm^{-1}	4.895
F(000)	2590.0
Crystal size/mm^3	0.35 × 0.05 × 0.04
Radiation	synchrotron (λ = 0.65255)
2Θ range for data collection/°	2.878 to 46.294
Index ranges	-18 ≤ h ≤ 18, -15 ≤ k ≤ 15, -29 ≤ l ≤ 29
Reflections collected	30119
Independent reflections	8741 [R_{int} = 0.1057, R_{sigma} = 0.1056]
Data/restraints/parameters	8741/554/621
Goodness-of-fit on F^2	1.147
Final R indexes [I>=2σ (I)]	R_1 = 0.1347, wR_2 = 0.3447
Final R indexes [all data]	R_1 = 0.1948, wR_2 = 0.3787
Largest diff. peak/hole / e Å^{-3}	6.08/-4.29

Crystallographic data of x974_1 and x974_2_1060uW were collected at separate locations on the same crystal at the PX II beamline of the SLS at PSI. The *DIALS* software suit[5] was used for indexing, integration, scaling and absorption correction.[10] The structure was solved with the dual-space algorithm using *SHELXT*[6] and was refined by full-matrix least-squares methods on F^2 with *SHELXL-2018*[11] using the *Olex2* GUI.[12] The graphical output was produced with the help of the program *Mercury*.[13] Given the reduced quality of the crystal after the crystal to crystal transition, the whole structure had to be treated with extended *RIGU* restraints.[14] Additionally, a number of other restraints (ISOR, DFIX and SIMU) had to applied. The nitrene and azide moieties were refined as a disorder with a 70:30 ratio. An independent refinement of the nitrene and α-azide nitrogen atoms was not possible.

- Compound **1** (Dichloromethane / Hexane): NV2111 (CCDC 2449338)

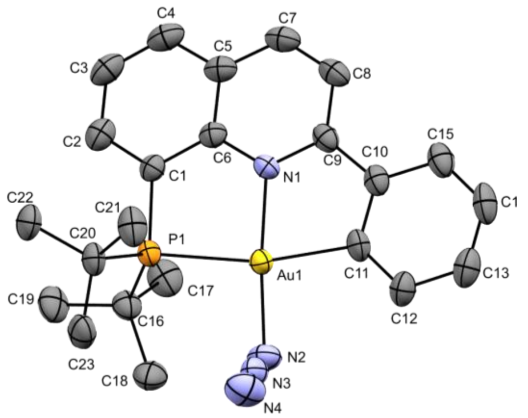

Supplementary Figure 60. Structure of compound **1**. The asymmetric unit contains one molecule. The ellipsoids are drawn at 50 % probability.

Supplementary Table 9. Crystal data and structure refinement for NV2111.

Identification code	NV2111
Empirical formula	$C_{23}H_{27}AuBF_4N_4P$
Formula weight	674.23
Temperature/K	160(2)
Crystal system	monoclinic
Space group	$P2_1/n$
a/Å	13.5158(3)
b/Å	12.5406(2)
c/Å	14.9286(3)
α/°	90
β/°	110.085(2)
γ/°	90
Volume/Å^3	2376.46(9)
Z	4
ρ_{calc}g/cm^3	1.884
μ/mm^{-1}	12.723
F(000)	1312.0
Crystal size/mm^3	0.19 × 0.14 × 0.04
Diffractometer	SuperNova
Radiation	Cu Kα (λ = 1.54184)
2Θ range for data collection/°	7.622 to 152.314
Index ranges	-17 ≤ h ≤ 16, -14 ≤ k ≤ 15, -16 ≤ l ≤ 18
Reflections collected	24652
Independent reflections	4941 [R_{int} = 0.0432, R_{sigma} = 0.0211]
Data/restraints/parameters	4941/0/313
Goodness-of-fit on F^2	1.023
Final R indexes [I>=2σ (I)]	R_1 = 0.0250, wR_2 = 0.0638
Final R indexes [all data]	R_1 = 0.0265, wR_2 = 0.0655
Largest diff. peak/hole / e Å^{-3}	1.99/-1.15

- Compound **3**: MAR220601_019_2_HP (CCDC 2449339)

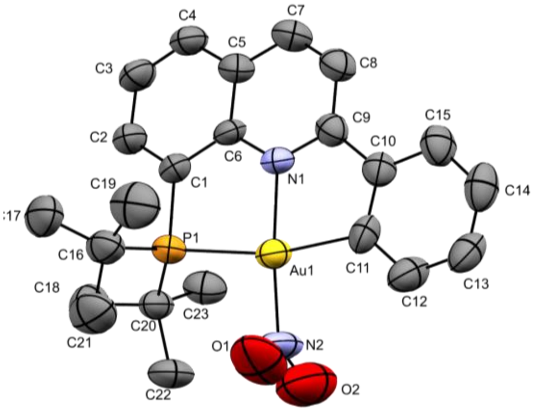

Supplementary Figure 61. Structure of compound **3**. The asymmetric unit contains one molecule. The ellipsoids are drawn at 50 % probability.

Supplementary Table 10. Crystal data and structure refinement for MAR220601_019_2_HP.

Identification code	MAR220601_019_2_HP
Empirical formula	$C_{23}H_{27}AuBF_4N_2O_2P$
Formula weight	678.21
Temperature/K	159.99(10)
Crystal system	monoclinic
Space group	$P2_1/c$
a/Å	12.4446(5)
b/Å	13.3094(5)
c/Å	14.8110(5)
α/°	90
β/°	91.934(4)
γ/°	90
Volume/Å^3	2451.75(16)
Z	4
ρ_{calc}g/cm^3	1.837
μ/mm^{-1}	12.375
F(000)	1320.0
Crystal size/mm^3	0.106 × 0.066 × 0.009
Diffractometer	Synergy
Radiation	Cu Kα (λ = 1.54184)
2Θ range for data collection/°	7.108 to 160.404
Index ranges	-15 ≤ h ≤ 15, -13 ≤ k ≤ 16, -17 ≤ l ≤ 18
Reflections collected	18891
Independent reflections	5024 [R_{int} = 0.0425, R_{sigma} = 0.0312]
Data/restraints/parameters	5024/0/313
Goodness-of-fit on F^2	1.090
Final R indexes [I>=2σ (I)]	R_1 = 0.0548, wR_2 = 0.1544
Final R indexes [all data]	R_1 = 0.0632, wR_2 = 0.1612
Largest diff. peak/hole / e Å^{-3}	3.08/-2.43

- Compound **1** (Dichloromethane / Cyclopentane): MAR220913_323_dcm_cp (CCDC 2449341)

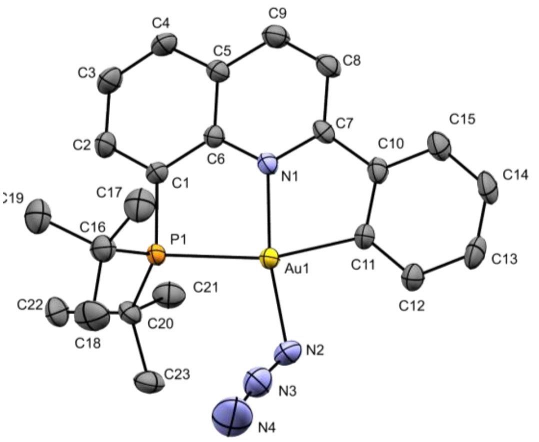

Supplementary Figure 62. Structure of **1**. The asymmetric unit contains two independent molecules, only one is shown for clarity. The ellipsoids are drawn at 50 % probability.

Supplementary Table 11. Crystal data and structure refinement for mar220913_323_dcm_cyclop_hp.

Identification code	mar220913_323_dcm_cyclop_hp
Empirical formula	$C_{23}H_{27}AuBF_4N_4P$
Formula weight	674.23
Temperature/K	160.15
Crystal system	monoclinic
Space group	$P2_1/n$
a/Å	14.48100(10)
b/Å	13.40070(10)
c/Å	25.1391(2)
α/°	90
β/°	93.2680(10)
γ/°	90
Volume/Å^3	4870.45(6)
Z	8
ρ_{calc}g/cm^3	1.839
μ/mm^{-1}	12.416
F(000)	2624.0
Crystal size/mm^3	0.302 × 0.104 × 0.033
Diffractometer	Synergy
Radiation	CuKα (λ = 1.54184)
2Θ range for data collection/°	6.88 to 160.516
Index ranges	-13 ≤ h ≤ 18, -17 ≤ k ≤ 17, -31 ≤ l ≤ 30
Reflections collected	70612
Independent reflections	10492 [R_{int} = 0.0580, R_{sigma} = 0.0354]
Data/restraints/parameters	10492/0/626
Goodness-of-fit on F^2	1.053
Final R indexes [I>=2σ (I)]	R_1 = 0.0391, wR_2 = 0.1082
Final R indexes [all data]	R_1 = 0.0404, wR_2 = 0.1093
Largest diff. peak/hole / e Å^{-3}	2.04/-1.79

- Mixture of **1** and **3** (70 : 30): MAR382_sctsc (CCDC 2449342)

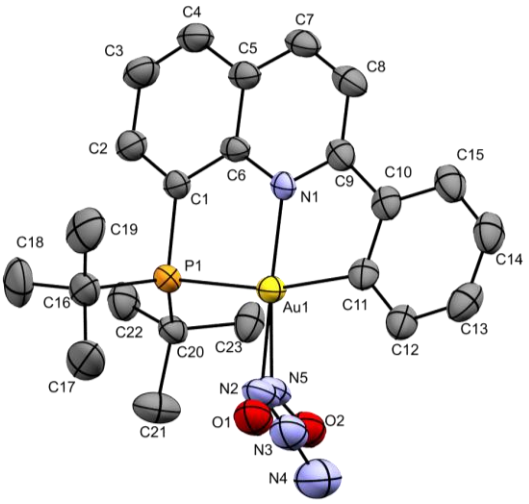

Supplementary Figure 63. Structure of compounds **1** and **3** (70 : 30 mixture). The asymmetric unit contains one molecule. The ellipsoids are drawn at 50 % probability.

Supplementary Table 12. Crystal data and structure refinement for MAR-382-SCtSC-2.

Identification code	MAR-382-SCtSC-2
Empirical formula	$C_{23}H_{27}AuBF_4N_{3.38}O_{0.62}P$
Formula weight	675.46
Temperature/K	160.00(10)
Crystal system	monoclinic
Space group	$P2_1/c$
a/Å	11.9892(3)
b/Å	12.7298(4)
c/Å	15.6905(4)
α/°	90
β/°	90.718(2)
γ/°	90
Volume/Å^3	2394.50(11)
Z	4
ρ_{calc}g/cm^3	1.874
μ/mm^{-1}	6.263
F(000)	1314.0
Crystal size/mm^3	0.227 × 0.187 × 0.092
Diffractometer	Synergy
Radiation	Mo Kα (λ = 0.71073)
2Θ range for data collection/°	4.12 to 64.648
Index ranges	-17 ≤ h ≤ 17, -18 ≤ k ≤ 18, -22 ≤ l ≤ 22
Reflections collected	22262
Independent reflections	7592 [R_{int} = 0.0391, R_{sigma} = 0.0459]
Data/restraints/parameters	7592/64/341
Goodness-of-fit on F^2	1.027
Final R indexes [I>=2σ (I)]	R_1 = 0.0395, wR_2 = 0.0916
Final R indexes [all data]	R_1 = 0.0740, wR_2 = 0.1036
Largest diff. peak/hole / e Å^{-3}	2.28/-0.91

- Mixture of **1** and **3** (50 : 50): MAR392_sctsc_221110 (CCDC 2449343)

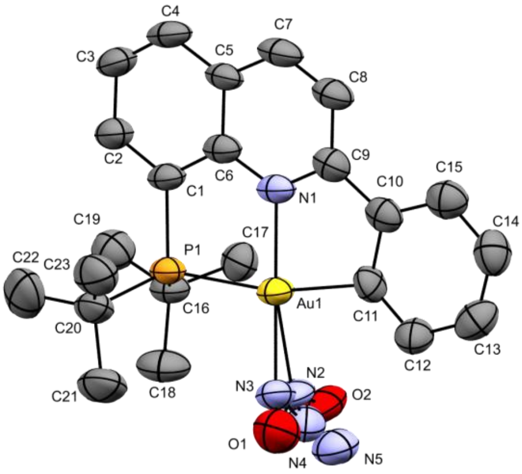

Supplementary Figure 64. Structure of compounds **1** and **3** (50 : 50 mixture). The asymmetric unit contains one molecule. The ellipsoids are drawn at 50 % probability.

Supplementary Table 13. Crystal data and structure refinement for mar-392-sc-t-sc_221110.

Identification code	mar-392-sc-t-sc_221110
Empirical formula	$C_{23}H_{27}AuBF_4N_{2.75}OP$
Formula weight	672.79
Temperature/K	162.15
Crystal system	monoclinic
Space group	$P2_1/c$
a/Å	12.0716(5)
b/Å	12.7726(6)
c/Å	15.5826(6)
α/°	90
β/°	90.597(4)
γ/°	90
Volume/Å^3	2402.47(18)
Z	4
ρ_{calc}g/cm^3	1.860
μ/mm^{-1}	12.600
F(000)	1309.0
Crystal size/mm^3	0.062 × 0.039 × 0.034
Diffractometer	Synergy
Radiation	CuKα (λ = 1.54184)
2Θ range for data collection/°	7.324 to 153.922
Index ranges	-14 ≤ h ≤ 15, -16 ≤ k ≤ 15, -19 ≤ l ≤ 14
Reflections collected	24660
Independent reflections	4993 [R_{int} = 0.0938, R_{sigma} = 0.0517]
Data/restraints/parameters	4993/35/341
Goodness-of-fit on F^2	1.031
Final R indexes [I>=2σ (I)]	R_1 = 0.0406, wR_2 = 0.0971
Final R indexes [all data]	R_1 = 0.0655, wR_2 = 0.1133
Largest diff. peak/hole / e Å^{-3}	1.41/-0.93

- Mixture of **1** and **3** (25 :75) recrystallized in Dichloromethane / Pentane: MAR-574_new (CCDC 2449344)

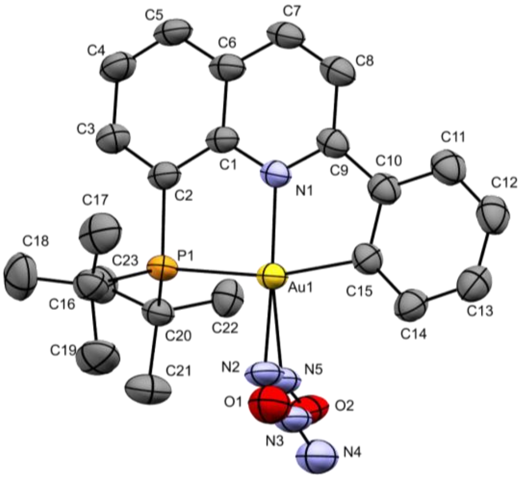

Supplementary Figure 65. Structure of compounds **1** and **3** (25 : 75 mixture). The asymmetric unit contains one molecule. The ellipsoids are drawn at 50 % probability.

Supplementary Table 14. Crystal data and structure refinement for MAR-574_new.

Identification code	MAR-574_new
Empirical formula	$C_{23}H_{27}AuBF_4N_{2.49}O_{1.51}P$
Formula weight	677.23
Temperature/K	159.99(10)
Crystal system	monoclinic
Space group	$P2_1/c$
a/Å	12.0701(2)
b/Å	12.7709(2)
c/Å	15.4875(2)
α/°	90
β/°	90.2570(10)
γ/°	90
Volume/Å^3	2387.31(6)
Z	4
ρ_{calc}g/cm^3	1.884
μ/mm^{-1}	12.699
F(000)	1318.0
Crystal size/mm^3	0.149 × 0.079 × 0.042
Diffractometer	Synergy
Radiation	Cu Kα (λ = 1.54184)
2Θ range for data collection/°	7.324 to 148.982
Index ranges	-14 ≤ h ≤ 15, -13 ≤ k ≤ 15, -19 ≤ l ≤ 18
Reflections collected	30105
Independent reflections	4876 [R_{int} = 0.0386, R_{sigma} = 0.0188]
Data/restraints/parameters	4876/90/341
Goodness-of-fit on F^2	1.158
Final R indexes [I>=2σ (I)]	R_1 = 0.0310, wR_2 = 0.0876
Final R indexes [all data]	R_1 = 0.0331, wR_2 = 0.0890
Largest diff. peak/hole / e Å^{-3}	0.95/-1.19

- Compound **4** (Dichloromethane/ Cyclopentane): mar-231020_591 (CCDC 2449345)

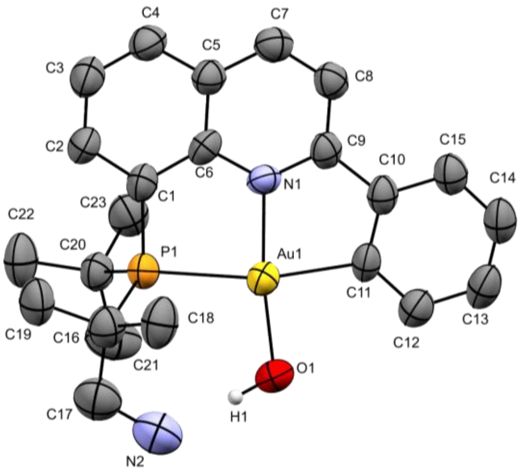

Supplementary Figure 66. Structure of compound **4**. The asymmetric unit contains one molecule. The ellipsoids are drawn at 50 % probability.

Supplementary Table 15. Crystal data and structure refinement for mar231020_591_yellow_hp.

Identification code	mar231020_591_yellow_hp
Empirical formula	$C_{24}H_{31}AuBCl_2F_4N_2OP$
Formula weight	749.15
Temperature/K	160.15
Crystal system	monoclinic
Space group	$P2_1/n$
a/Å	13.17583(19)
b/Å	13.43262(19)
c/Å	15.2513(2)
α/°	90
β/°	93.3525(13)
γ/°	90
Volume/Å^3	2694.64(7)
Z	4
ρ_{calc}g/cm^3	1.847
μ/mm^{-1}	13.081
F(000)	1464.0
Crystal size/mm^3	0.31 × 0.195 × 0.026
Diffractometer	Synergy
Radiation	CuKα (λ = 1.54184)
2Θ range for data collection/°	8.624 to 161.906
Index ranges	-16 ≤ h ≤ 16, -16 ≤ k ≤ 17, -17 ≤ l ≤ 19
Reflections collected	69176
Independent reflections	5866 [R_{int} = 0.1196, R_{sigma} = 0.0469]
Data/restraints/parameters	5866/1/332
Goodness-of-fit on F^2	1.099
Final R indexes [I>=2σ (I)]	R_1 = 0.0711, wR_2 = 0.2069
Final R indexes [all data]	R_1 = 0.0755, wR_2 = 0.2126
Largest diff. peak/hole / e Å^{-3}	5.37/-1.58

- Compound **5** (Dichloromethane / Cyclopentane): mar_231110_584_dcm_cp_hp (CCDC 2449346)

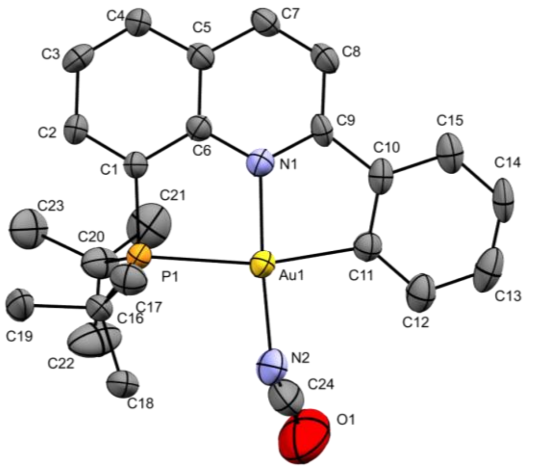

Supplementary Figure 67. Structure of compound **5**. The asymmetric unit contains two independent molecules, only one is shown for clarity. The ellipsoids are drawn at 50 % probability.

Supplementary Table 16. Crystal data and structure refinement for MAR231110_584_DCM_Cp_HP.

Identification code	MAR231110_584_DCM_Cp_HP
Empirical formula	$C_{24}H_{27}BN_2OF_4PAu$
Formula weight	674.22
Temperature/K	160.00(10)
Crystal system	monoclinic
Space group	$P2_1/n$
a/Å	14.6408(3)
b/Å	13.3220(2)
c/Å	25.1868(5)
α/°	90
β/°	93.088(2)
γ/°	90
Volume/Å^3	4905.42(16)
Z	8
ρ_{calc}g/cm^3	1.826
μ/mm^{-1}	6.114
F(000)	2624.0
Crystal size/mm^3	0.57 × 0.21 × 0.13
Diffractometer	Synergy
Radiation	Mo Kα (λ = 0.71073)
2Θ range for data collection/°	4.388 to 67.68
Index ranges	$-20 \le h \le 19$, $-20 \le k \le 20$, $-38 \le l \le 36$
Reflections collected	66408
Independent reflections	17181 [R_{int} = 0.0463, R_{sigma} = 0.0421]
Data/restraints/parameters	17181/24/634
Goodness-of-fit on F^2	1.149
Final R indexes [I>=2σ (I)]	R_1 = 0.0706, wR_2 = 0.1807
Final R indexes [all data]	R_1 = 0.1027, wR_2 = 0.1940
Largest diff. peak/hole / e Å^{-3}	5.45/-2.14

- Compound **6** (Acetonitrile / Pentane): NV2118 (CCDC 2449347)

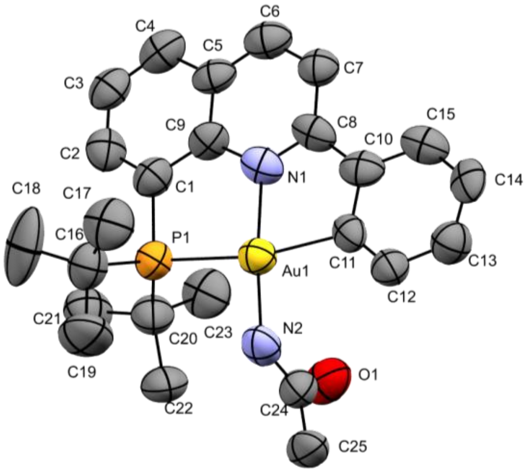

Supplementary Figure 68. Structure of compound **6**. The asymmetric unit contains two independent molecules, only one is shown for clarity. The ellipsoids are drawn at 50 % probability.

Supplementary Table 17. Crystal data and structure refinement for nv2118-final.

Identification code	nv2118-final
Empirical formula	$C_{56}H_{73}Au_2B_2F_8N_7O_3P_2$
Formula weight	1521.70
Temperature/K	160(2)
Crystal system	monoclinic
Space group	$P2_1/c$
a/Å	30.3957(8)
b/Å	14.2933(6)
c/Å	13.7396(5)
α/°	90
β/°	97.247(3)
γ/°	90
Volume/Å^3	5921.6(4)
Z	4
ρ_{calc}g/cm^3	1.707
μ/mm^{-1}	10.324
F(000)	3008.0
Crystal size/mm^3	0.08 × 0.02 × 0.02
Diffractometer	SuperNova
Radiation	Cu Kα (λ = 1.54184)
2Θ range for data collection/°	11.158 to 133.196
Index ranges	-36 ≤ h ≤ 29, -17 ≤ k ≤ 16, -16 ≤ l ≤ 16
Reflections collected	31172
Independent reflections	9357 [R_{int} = 0.1532, R_{sigma} = 0.1373]
Data/restraints/parameters	9357/24/738
Goodness-of-fit on F^2	0.963
Final R indexes [I>=2σ (I)]	R_1 = 0.0888, wR_2 = 0.1846
Final R indexes [all data]	R_1 = 0.1429, wR_2 = 0.2103
Largest diff. peak/hole / e Å^{-3}	2.00/-1.39

9 Density functional and ab-initio calculations

The calculations were performed with ORCA 5.0.4.[15-17] Geometry optimizations were performed with the PBE0[18] functional in combination with the ZORA-def2-SVP[19] basis set except for gold (SARC-ZORA-TZVP),[20] and the D4[21] dispersion correction. Scalar relativistic effects were modelled by the zero-order regular approximation (ZORA).[22,23] Initial explorations with the r^2SCAN-3c[24] composite electronic-structure method revealed that this method struggled to locate transition states, in some cases due to abundant conical intersections, yet ground state energies proved consistent with the PBE0 data. The transition states were initially located by the NEB-CI/NEB-TS methods,[25] thus also verifying the connection between the transition states and respective ground states (in selected cases, additional reaction coordinate scans were performed to further corroborate the connectivity). All singlet structures were computed with the unrestricted formalism, antiferromagnetic states were explored with the broken-symmetry formalism based on corresponding high-spin states. For clarity, “true” antiferromagnetic diradical (tetraradical, respectively) states are designated in the following with *oss* (*open-shell singlet*), whereas both closed-shell and spin-polarized singlet states are designated with *s* (*singlet*). All computed structures were verified as true minima by the absence of imaginary eigenvalues in the harmonic vibrational frequency analysis (or the presence of only one imaginary eigenvalue in case of transition states). Tighter than default convergence criteria were chosen for both the optimization of the structural parameters (*tightopt*) as well as the SCF (*tightscf*).

The energies of all structures were refined through single-point calculations at the ZORA-PBE0-D4/ZORA-def2-TZVPP//PBE0-D4/ZORA-def2-SVP and ZORA-ωB97X-V/ZORA-def2-TZVPP//PBE0-D4/ZORA-def2-SVP levels of theory, effects of implicit solvation in THF were explored by ZORA-PBE0-D4(CPCM=THF)/ZORA-def2-TZVPP/PBE0-D4/ZORA-def2-SVP calculations.[26,27] These two functionals were chosen as they rank among the most accurate hybrid functionals for energies and barriers in transition metal chemistry.[28] The *RIJCOSX*[29,30] approximation in combination with the auxiliary basis sets (*SARC/J*[31] and *autoaux*[32] for Aux/C, where applicable) was used to speed up all calculations. NBO calculations were performed by NBO v7.0,[33] and were calculated at the ZORA-PBE0-D4/ZORA-def2-TZVPP//PBE0-D4/ZORA-def2-SVP as well as PBE0-D4/def2-TZVPP//PBE0-D4/def2-SVP levels of theory (def2-ECP for Au) for enhanced comparability with the literature.[34] Intrinsic Bond Orbitals (IBOs)[35] were calculated at the PBE0-D4/def2-TZVPP//PBE0-D4/def2-SVP level of theory. UV-Vis absorption spectra were modelled by TD-DFT (Tamm-Dancoff approximation, 30 roots) at the ZORA-PBE0(CPCM=CH_2Cl_2)/ZORA-def2-TZVPP//PBE0-D4/ZORA-def2-SVP level of theory.

Zero-Field-Splitting (ZFS) was computed (i) by QDPT at the NEVPT2//ZORA-saCASSCF(18,13) level of theory (1 quintet, 5 triplet, 5 singlet roots) as well as (ii) at the X2C-PBE0/X2C-TZVPPall//PBE0-D4/def2-SVP level of theory (Supplementary Table 29).

Furthermore, state-of-the art ZORA-DLPNO-CCSD(T_1)/def2-TZVPP single points were computed.[36] These computations were conducted with the PBE0 reference and default frozen-core as well as *normalpno* settings, as *tightpno* proved computationally too demanding, and the improved iterative T_1 triples correction.[37,38] Whereas the energies of open-shell ground/transition states are deemed reliable (with similar energies to these obtained at the ZORA-ωB97X-V/ZORA-def2-TZVPP//PBE0-D4/ZORA-def2-SVP level of theory), some excited singlet states afford unreliable energies (with large singles norms, *etc.*; Supplementary Tables 25–28). In the energy profiles shown below, energies of excited singlet states hence are shown only for the DFT calculations.

The CASSCF calculations for the aura-nitrene were performed using the structural parameters obtained by r^2SCAN-3c. Two active spaces were explored in detail, namely 12 electrons in 13 orbitals, *i.e.* CAS(12,13), which included the nitrene's *p*(x), *p*(y) and *p*(z) orbitals, the gold atom's $d(x^2\text{-}y^2)$ orbital, as well as the π-system of the ligand (Supplementary Figure 74-75). Furthermore, CAS(18,13) was explored (Supplementary Figure 76-77), where additionally the nitrene's *s* orbital, the metal's *d*(xz) and *d*(yz) orbitals, as well as one orbital relating to the σ-donors of the ancillary pincer ligand were included at the expense of pincer π-orbitals. Including also the other 5*d* orbitals in the active space led to difficult-to-converge wavefunctions due to their low energy (n_{occ} = 2.0). The active spaces were built up from various reference orbitals (HF, PBE0, QROs), and the most appropriate active spaces were selected based on their mixing, symmetry and energies. State-averaging was applied as indicated. Vertical energies obtained by both active spaces are consistent. Molecular orbitals were visualized with IBOView[39] and ChemCraft.[40]

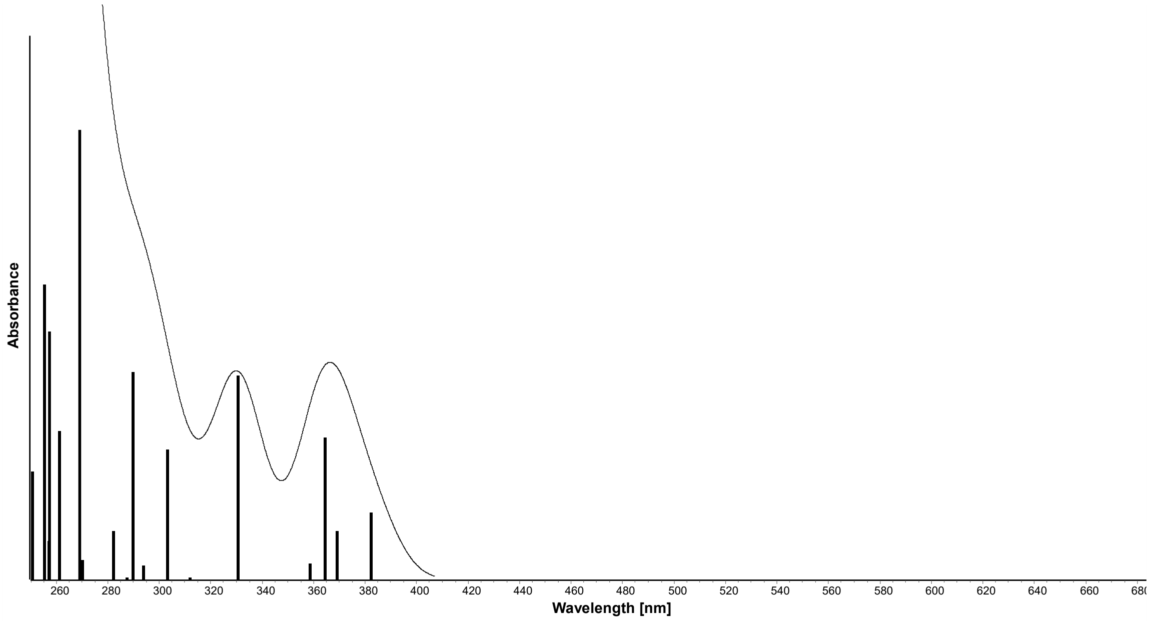

Supplementary Figure 69. Calculated absorption spectrum (TD-DFT) of **1**.

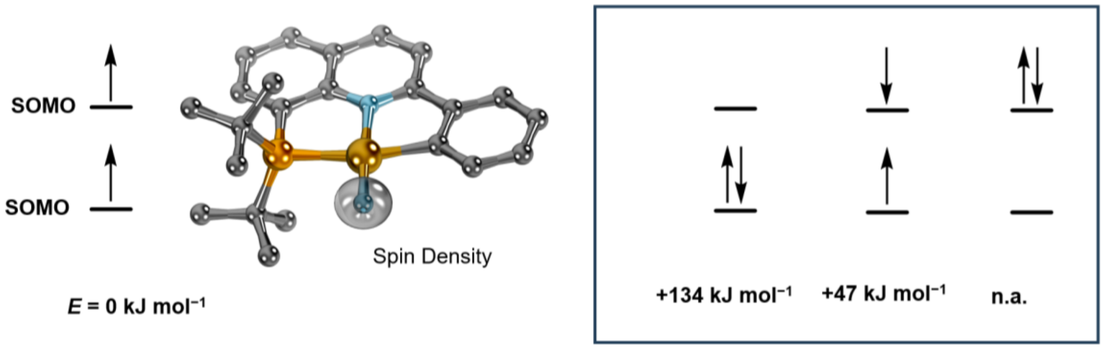

Supplementary Figure 70. Spin-density in triplet ground state of the aura-nitrene **2** (left), as well as adiabatic energy gaps Δ*E* (right) to closed-shell and open-shell singlet states (ZORA-PBE0-D4/def2-TZVPP//ZORA-PBE0-D4/def2-SVP).

Supplementary Table 18. Population analysis of aura-nitrene **2** (ZORA-PBE0-D4/ZORA-def2-TZVPP//ZORA-PBE0-D4/ZORA-def2-SVP). In case of the NBO analysis, additionally the values obtained at the PBE0-D4/def2-TZVPP//PBE0-D4/def2-SVP using the def2-ECP for gold are given for comparison with the computed values reported for Schneider's Pd- and Pt nitrenes.[41]

	Löwdin Atomic Spin Density Au in [a.u]	+0.14
	Löwdin Atomic Spin Density N^1 in [a.u]	+1.81
	Hirshfeld Atomic Spin Density Au in [a.u]	+0.16
	Hirshfeld Atomic Spin Density N^1 in [a.u]	-1.75
	NPA Atomic Spin Density Au in [a.u]	+0.02
	NPA Atomic Spin Density N^1 in [a.u]	+1.96
	NPA Atomic Spin Density Au, ECP, in [a.u]	+0.02

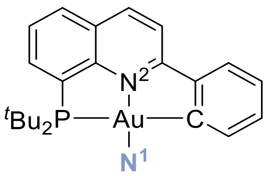	NPA Atomic Spin Density N^1, ECP, in [a.u]	+1.97
	Löwdin Atomic Partial Charge Au in [a.u]	-0.19
	Löwdin Atomic Partial Charge N^1 in [a.u]	+0.01
	Hirshfeld Atomic Partial Charge Au in [a.u]	+0.39
	Hirshfeld Atomic Partial Charge N^1 in [a.u]	-0.16
	ChElPG Atomic Partial Charge Au in [a.u]	+0.37
	ChElPG Atomic Partial Charge N^1 in [a.u]	-0.30
	Bader Atomic Partial Charge Au in [a.u.]	+0.61
	Bader Atomic Partial Charge N in [a.u.]	-0.33
	NPA Atomic Partial Charge Au in [a.u]	+0.96
	NPA Atomic Partial Charge N^1 in [a.u]	-0.32
	NPA Atomic Partial Charge Au, ECP, in [a.u]	+1.00
	NPA Atomic Partial Charge N^1, ECP, in [a.u]	-0.34
	Wiberg Bond Index (NAO basis) Au–N^1	0.75
	Wiberg Bond Index (NAO basis), ECP, Au–N^1	0.74
	Mayer Bond Order Au–N^2	1.07
	Mayer Bond Order Au–N^1	0.59
	Mayer Bond Order Au–P	0.81
	Mayer Bond Order Au–C	0.80

Supplementary Table 19. Comparison of spin-distribution in aura-nitrene **2** (ZORA-PBE0-D4/ZORA-def2-TZVPP//ZORA-PBE0-D4/ZORA-def2-SVP) with the corresponding hypothetical copper (**2**^{Cu}) and silver (**2**^{Ag}) complexes; values are given in [a.u.].

**Bader_N**			**-0.33**
Spin Population	Cu	Ag	Au
Loewdin_M	0.05	0.04	0.14
Loewdin_N	1.89	1.99	1.81
Hirshfeld_M	0.09	0.09	0.16
Hirshfeld_N	1.8	1.9	1.75
NPA_M	-0.04	-0.02	0.02

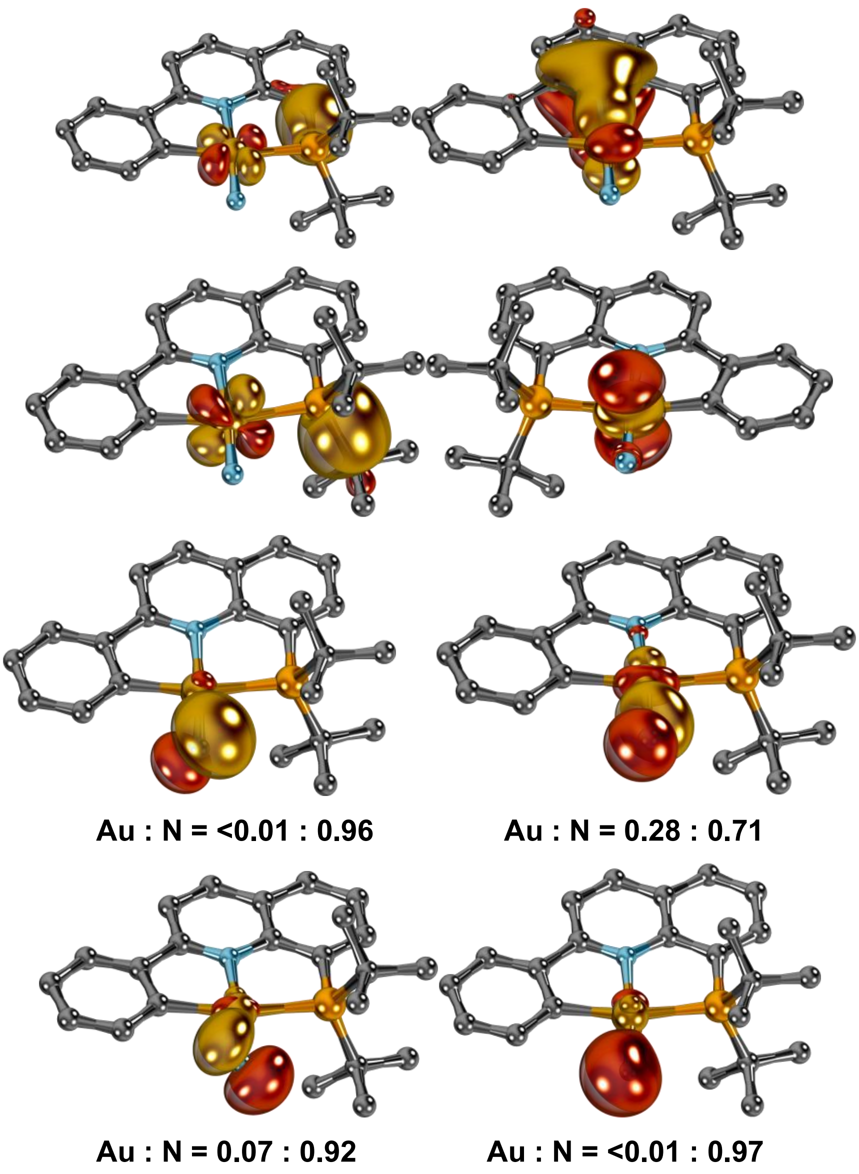

Supplementary Figure 71. Pertinent α-Intrinsic Bond Orbitals (IBOs) of **2** (PBE0/def2-TZVPP//PBE0-D4/def2-SVP). The *d*-orbitals show mixing with ligand-based orbitals, yet only the orbital with the major metal-contribution is shown for clarity. Mixing of nitrene- with gold-centered valence orbitals is quantified with the default Mulliken population analysis.

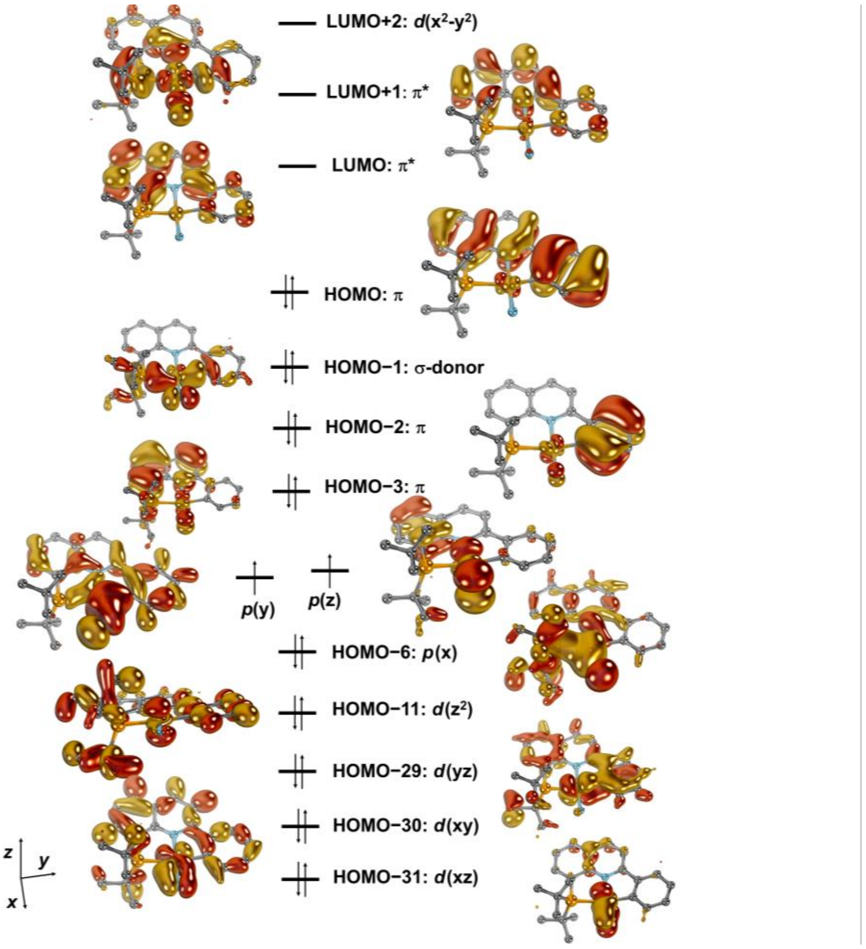

Supplementary Figure 72. Pertinent canonical molecular orbitals of aura-nitrene **2** (plotted orbitals relate to the α-MOs) as obtained at the PBE0-D4/def2-TZVPP//PBE0-D4/def2-SVP level of theory. The calculations indicate a moderate degree of spin contamination ($\langle S^2 \rangle$ 2.015), a non-aufbau electron configuration, and (arguably undesired) mixing of the p(x) and p(y) orbitals. Note that the coordinate systems is flipped in respect to the CASSCF-derived MO-diagrams below.

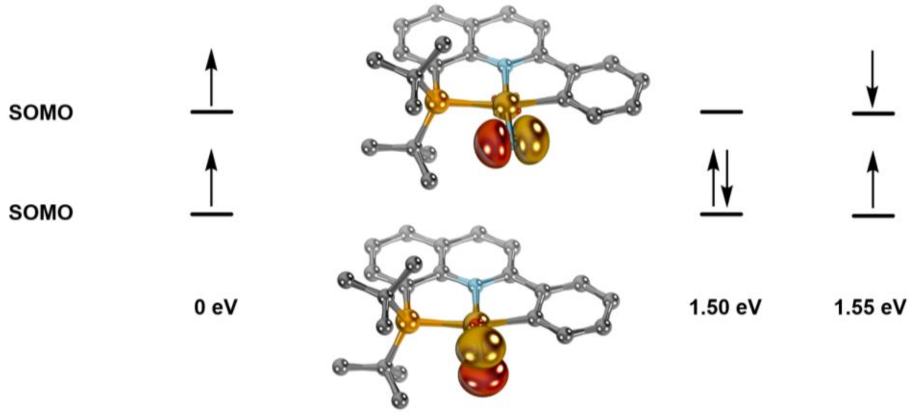

Supplementary Figure 73. The two singly-occupied natural orbitals of the aura-nitrene **2** (left; ZORA-CASSCF(12,13)/def2-TZVPP; no state-averaging), and the vertical energy gaps ΔE to closed-shell and open-shell singlet states (right; NEVPT2//ZORA-CASSCF(12,13)/def2-TZVPP with state averaging: 15 triplet roots, 10 singlet roots).

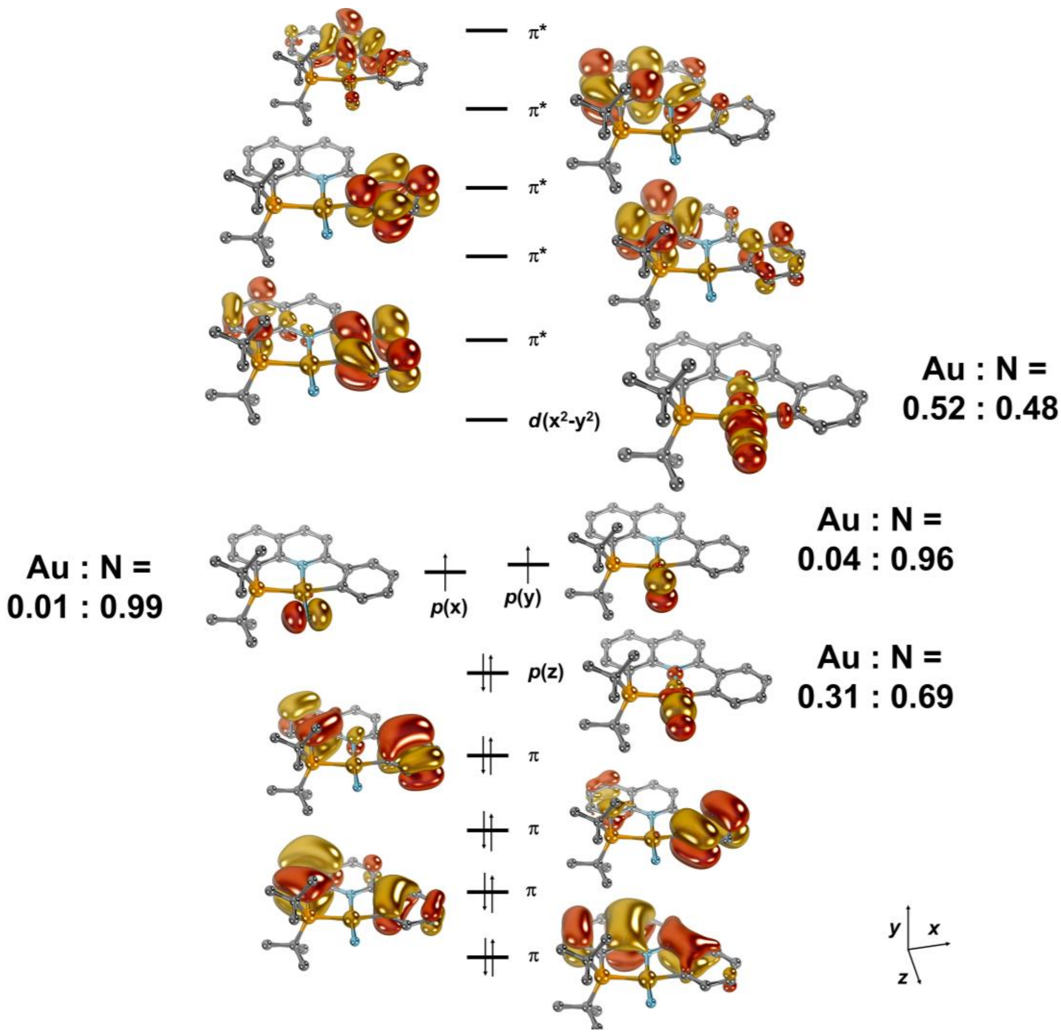

Supplementary Figure 74. Entire active space (ZORA-CASSCF(12,13)/def2-TZVPP *without* state-averaging) for aura-nitrene **2**. The orbitals' order follows their occupancy and not energy; the in-plane *p*(x) orbital is higher in energy than the out-of-plane *p*(y) orbital. Orbital compositions are given using Löwdin's population analysis.

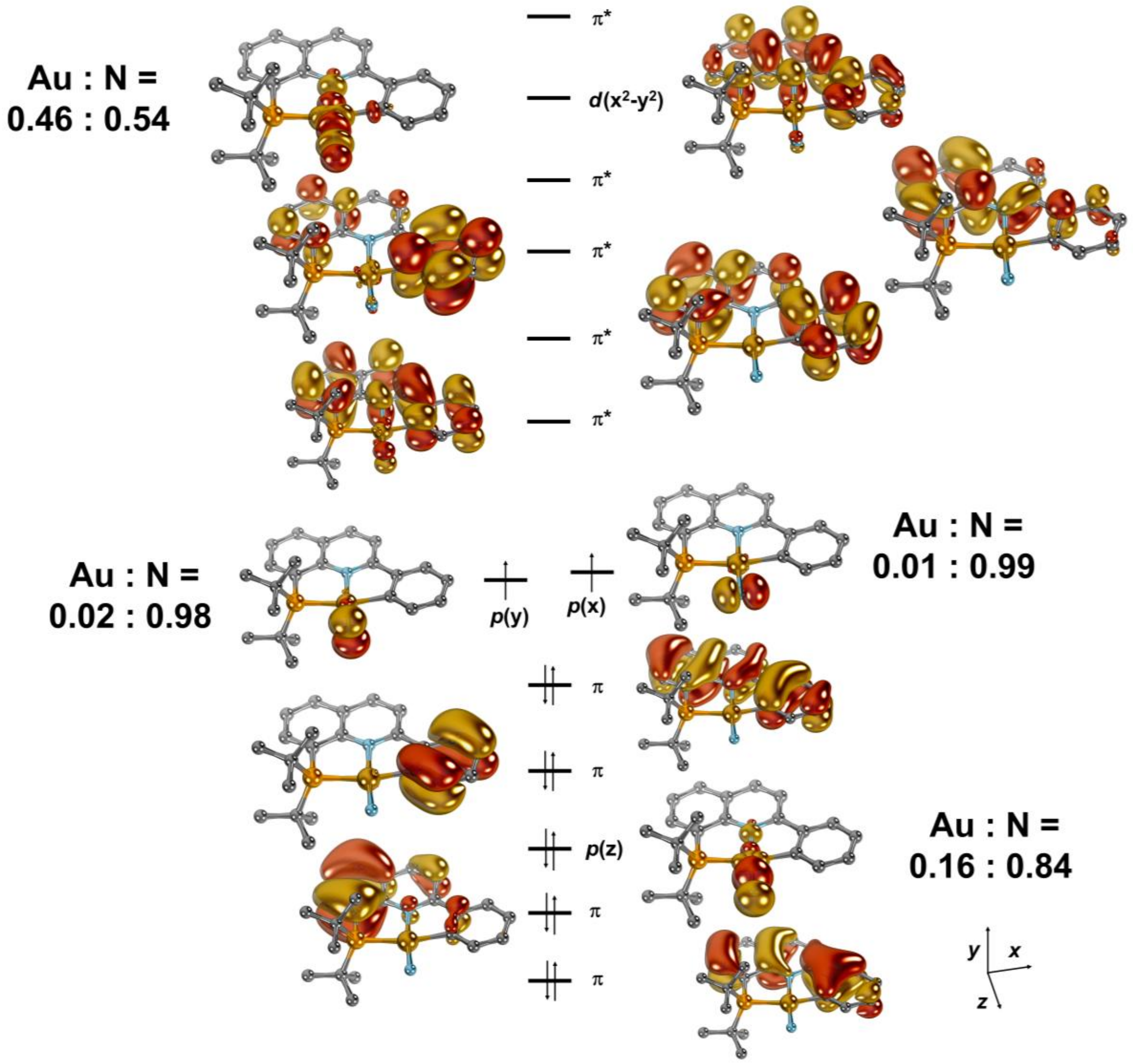

Supplementary Figure 75. Entire active space (ZORA-CASSCF(12,13)/def2-TZVPP *with* state-averaging 15 triplet, 10 singlet roots) for aura-nitrene **2**. The orbitals' order follows their occupancy and not energy; the in-plane *p*(x) orbital is higher in energy than the out-of-plane *p*(y) orbital. Orbital compositions are given using Löwdin's population analysis.

Supplementary Table 20. Configurations of selected states and their weight (ZORA-CASSCF(12,13)/def2-TZVPP *without* and *with* state-averaging: 15 triplet roots, 10 singlet roots) for the aura-nitrene **2**.

```
--------------------------------------------
CAS-SCF  STATES  FOR  BLOCK   1  MULT=  3
NROOTS= 1 NO STATE AVERAGING
--------------------------------------------
ROOT   0:  E=  -21009.5262496794 Eh
      0.85059 [     0]: 2222211000000
      0.02510 [   167]: 2222011200000
      0.01208 [    36]: 2222111100000
      0.01192 [  7512]: 2202211000200
      0.00850 [  3654]: 2211211010100
      0.00641 [127107]: 0222211000020
      0.00640 [ 33918]: 2022211002000
      0.00628 [  1293]: 2220211020000
      0.00601 [ 77388]: 1122211001010
      0.00530 [ 14948]: 2121211011000
      0.00332 [ 18807]: 2112211010100
      0.00267 [ 58420]: 1221211000020

--------------------------------------------
CAS-SCF STATES FOR BLOCK  1 MULT= 3
STATE AVERAGING
--------------------------------------------

ROOT   0:
      0.83911 [     0]: 2222211000000
      0.02453 [  7515]: 2202211000020
      0.00912 [     7]: 2222201100000
      0.00908 [  2742]: 2212211000010
      0.00811 [  1298]: 2220211002000
      0.00458 [  1294]: 2220211011000
      0.00347 [   526]: 2221111101000
      0.00272 [  1293]: 2220211020000
      0.00268 [ 33908]: 2022211110000
      0.00264 [   182]: 2222011000200
--------------------------------------------
CAS-SCF STATES FOR BLOCK  2 MULT= 1
--------------------------------------------
ROOT   0:
      0.54772 [     0]: 2222220000000
      0.30123 [     8]: 2222202000000
      0.01473 [  7615]: 2202220000020
      0.00878 [  7713]: 2202202000020
      0.00852 [     2]: 2222210100000
      0.00528 [  1314]: 2220220002000
      0.00298 [  1310]: 2220220011000
      0.00291 [  1412]: 2220202002000
ROOT   1:
      0.85124 [     1]: 2222211000000
      0.02385 [  7642]: 2202211000020
      0.00820 [  1341]: 2220211002000
      0.00652 [     9]: 2222201100000
      0.00464 [  1337]: 2220211011000
      0.00350 [   562]: 2221111101000
      0.00275 [  1336]: 2220211020000
      0.00271 [ 34245]: 2022211110000
      0.00268 [   197]: 2222011000200
      0.00252 [   182]: 2222011200000
ROOT   2:
      0.55176 [     8]: 2222202000000
      0.29469 [     0]: 2222220000000
      0.01511 [  7713]: 2202202000020
      0.01026 [  7590]: 2202222000000
      0.00709 [  7615]: 2202220000020
      0.00530 [  1412]: 2220202002000
      0.00299 [  1408]: 2220202011000
      0.00283 [  1314]: 2220220002000
```

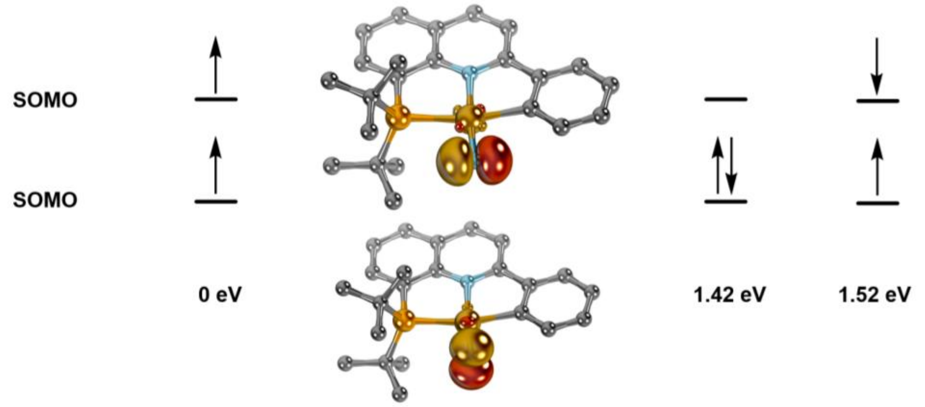

Supplementary Figure 76. The two singly-occupied natural orbitals of the aura-nitrene **2** (left; ZORA-CASSCF(18,13)/def2-TZVPP; no state-averaging), and the vertical energy gaps Δ*E* to closed-shell and open-shell singlet states (right; NEVPT2//ZORA-CASSCF(12,13)/def2-TZVPP with state averaging: 15 triplet roots, 10 singlet roots).

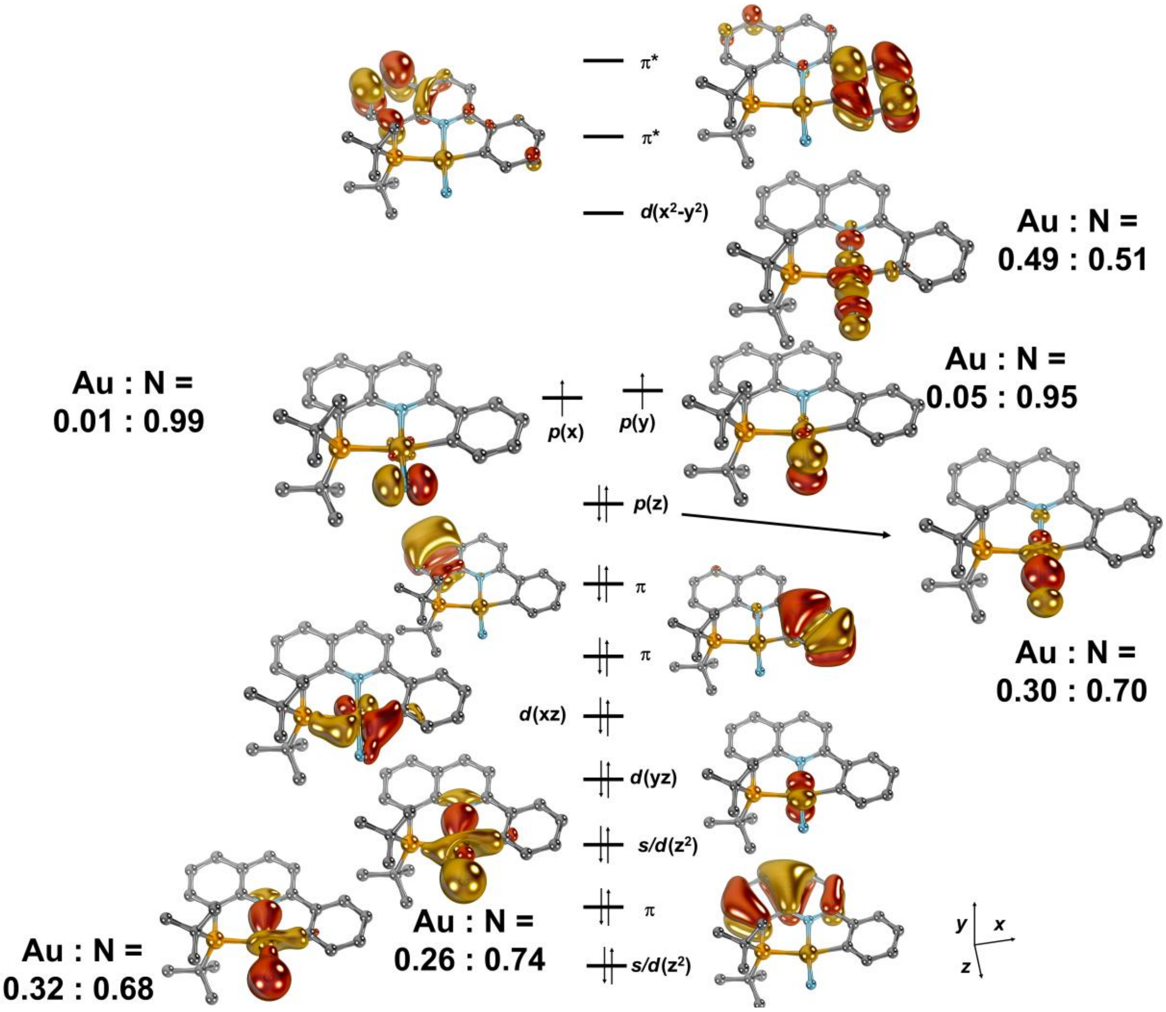

Supplementary Figure 77. Entire active space (ZORA-CASSCF(18,13)/def2-TZVPP without state-averaging. The orbitals' order follows their occupancy and not energy; the in-plane *p*(x) orbital is higher in energy than the out-of-plane *p*(y) orbital. Orbital compositions are given using Löwdin's population analysis.

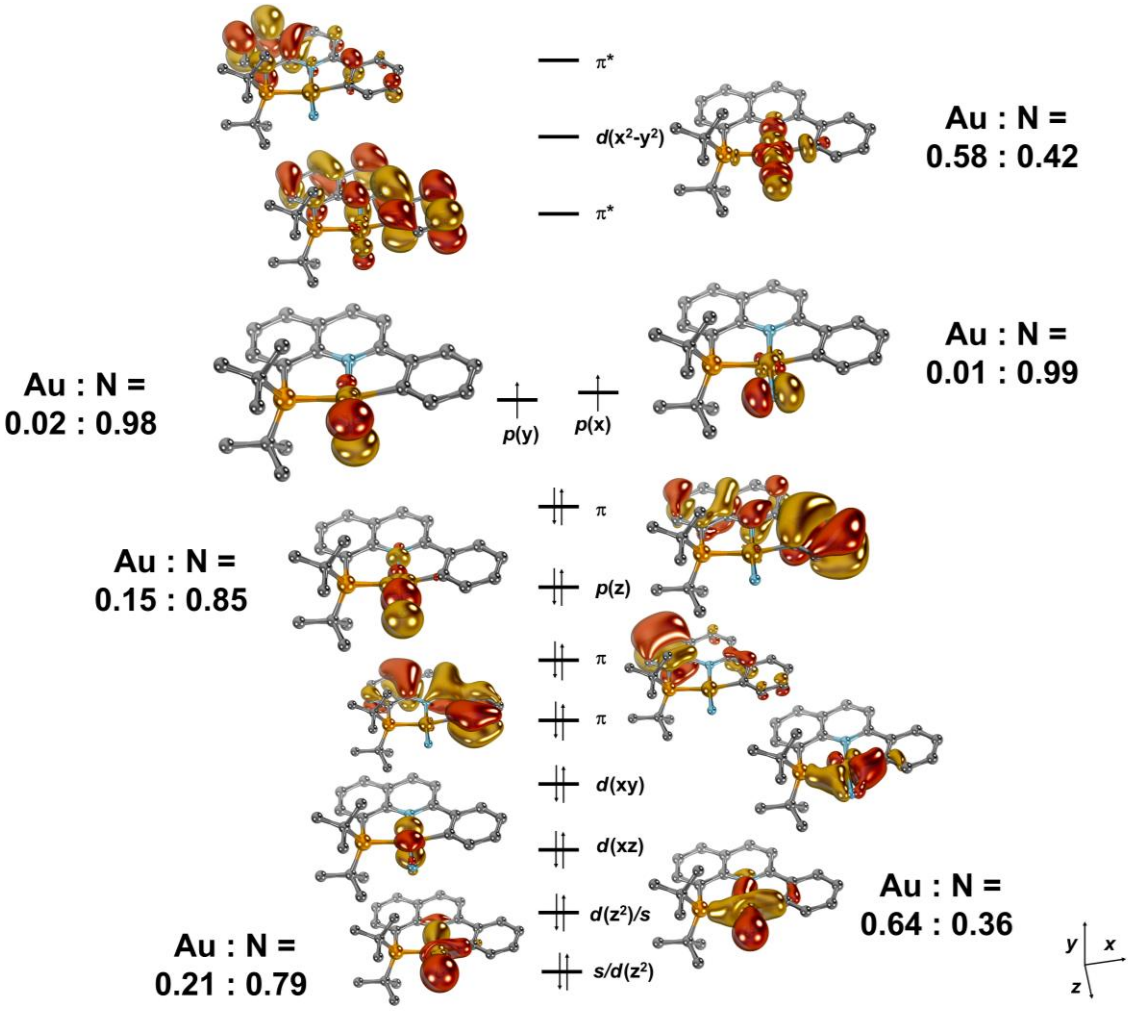

Supplementary Figure 78. Entire active space (ZORA-CASSCF(18,13)/def2-TZVPP with state-averaging: 1 quintet, 10 triplet, 10 singlet roots) for the aura-nitrene **2**. The orbitals' order follows their occupancy and not energy; the in-plane *p*(x) orbital is higher in energy than the out-of-plane *p*(y) orbital. Orbital compositions are given using Löwdin's population analysis.

Supplementary Table 21. Configurations of selected states and their weight (ZORA-CASSCF(18,13)/def2-TZVPP *without* and *with* state-averaging: 1 quintet, 10 triplet, 10 singlet roots) for the aura-nitrene **2**.

```
---------------------------------------------
CAS-SCF STATES FOR BLOCK  2 MULT= 3
NO STATE AVERAGING
---------------------------------------------

ROOT   0:
     0.88988 [ 51908]: 2222222211000
     0.01922 [ 51695]: 2222220211020
     0.01585 [ 51179]: 2222202211002
     0.01013 [ 51827]: 2222221211010
     0.00929 [ 51859]: 2222222011200
     0.00883 [ 51904]: 2222222201100
     0.00529 [ 51182]: 2222202211101
     0.00484 [ 48990]: 2221221212010
     0.00434 [ 45225]: 2212221221010

---------------------------------------------
CAS-SCF STATES FOR BLOCK  3 MULT= 1
STATE AVERAGING
---------------------------------------------

ROOT   0:
     0.69837 [ 52623]: 2222222220000
     0.19396 [ 52618]: 2222222202000
     0.01454 [ 49588]: 2221221221010
     0.01386 [ 52400]: 2222220220020
     0.01242 [ 51864]: 2222202220002
     0.00828 [ 52621]: 2222222210100
     0.00739 [ 52574]: 2222222020200
     0.00424 [ 52378]: 2222220202020
     0.00419 [ 51867]: 2222202220101
     0.00346 [ 51842]: 2222202202002
     0.00292 [ 45721]: 2212221212010
ROOT   1:
     0.89910 [ 52622]: 2222222211000
     0.01868 [ 52391]: 2222220211020
     0.01602 [ 51855]: 2222202211002
     0.00933 [ 52565]: 2222222011200
     0.00883 [ 49579]: 2221221212010
     0.00779 [ 45730]: 2212221221010
     0.00575 [ 52617]: 2222222201100
     0.00533 [ 51858]: 2222202211101
     0.00251 [ 52221]: 2222212111101
ROOT   2:
     0.69334 [ 52618]: 2222222202000
     0.17679 [ 52623]: 2222222220000
     0.01493 [ 45721]: 2212221212010
     0.01451 [ 52378]: 2222220202020
     0.01238 [ 51842]: 2222202202002
     0.00702 [ 52552]: 2222222002200
     0.00622 [  8073]: 0222222222000
     0.00608 [ 52407]: 2222220222000
     0.00603 [ 15267]: 1122222222000
     0.00568 [ 49588]: 2221221221010
     0.00406 [ 51845]: 2222202202101
     0.00326 [ 52400]: 2222220220020
     0.00314 [ 51864]: 2222202220002
     0.00268 [ 24183]: 1222221222000
```

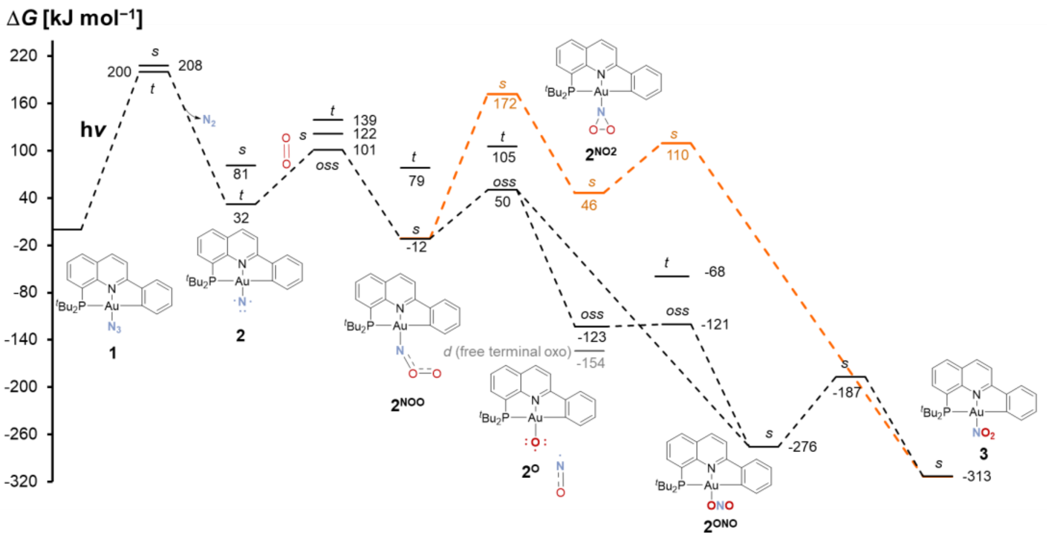

Supplementary Figure 79. Disfavored mechanisms and intermediates for dioxygen addition based on ZORA-PBE0-D4/def2-TZVPP//ZORA-PBE0-D4/def2-SVP; *s*, singlet (closed-shell or spin-polarized open-shell); *oss* (open-shell singlet, antiferromagnetic coupling); *t*, triplet; *d*, doublet.

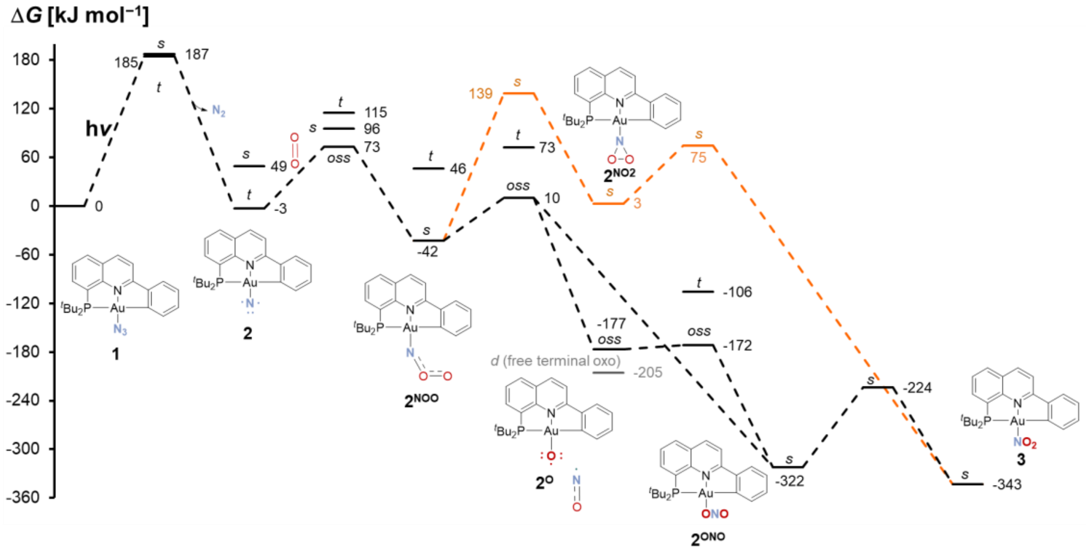

Supplementary Figure 80. Disfavored mechanisms and intermediates for dioxygen activation based on ZORA-ωB97X-V/def2-TZVPP//ZORA-PBE0-D4/def2-SVP; *s*, singlet (closed-shell or spin-polarized open-shell); *oss* (open-shell singlet, antiferromagnetic coupling); *t*, triplet; *d*, doublet.

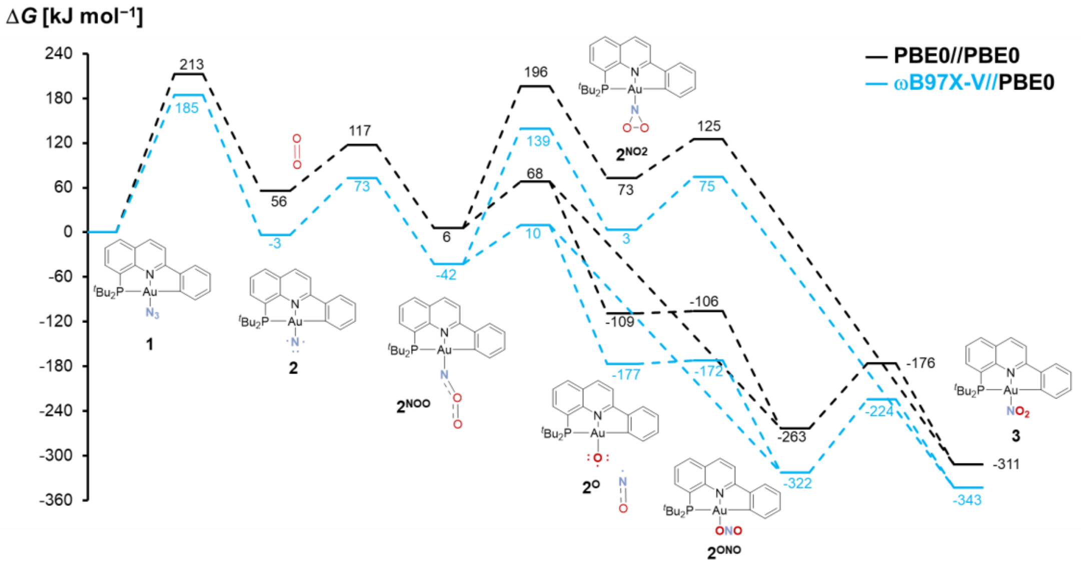

Supplementary Figure 81. Comparison of single-point approaches: Most favorable pathways for dioxygen addition as obtained at the ZORA-ωB97X-V/def2-TZVPP//ZORA-PBE0-D4/def2-SVP (blue) and ZORA-PBE0-D4/def2-TZVPP//ZORA-PBE0-D4/def2-SVP (black) level of theories.

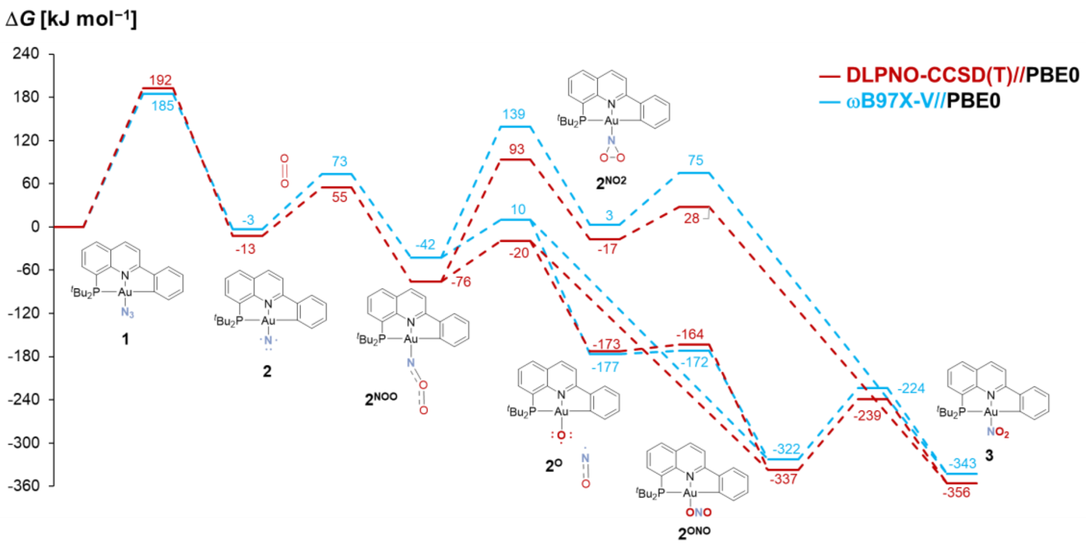

Supplementary Figure 82. Comparison of single-point approaches: Most favorable pathway for dioxygen addition as obtained at the ZORA-DLPNO-CCSD(T)/def2-TZVPP//ZORA-PBE0-D4/def2-SVP (red) and ZORA-ωB97X-V/def2-TZVPP//ZORA-PBE0-D4/def2-SVP (blue) levels of theories.

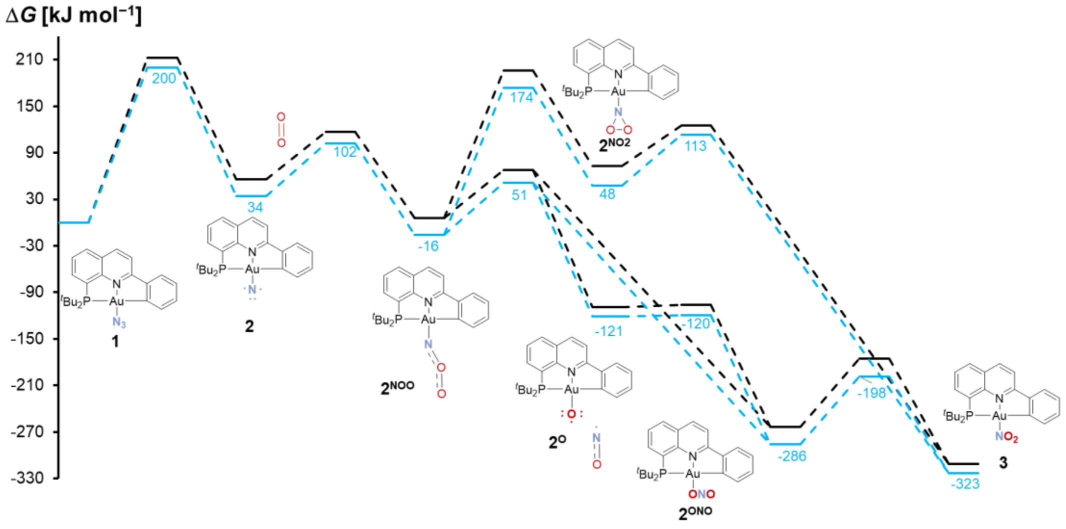

Supplementary Figure 83. The polarity of the reaction medium has only a moderate effect on computed kinetics and thermodynamics (black, ZORA-PBE0-D4/def2-TZVPP//ZORA-PBE0-D4/def2-SVP; blue, ZORA-PBE0-D4(CPCM=THF)/def2-TZVPP//ZORA-PBE0-D4/def2-SVP).

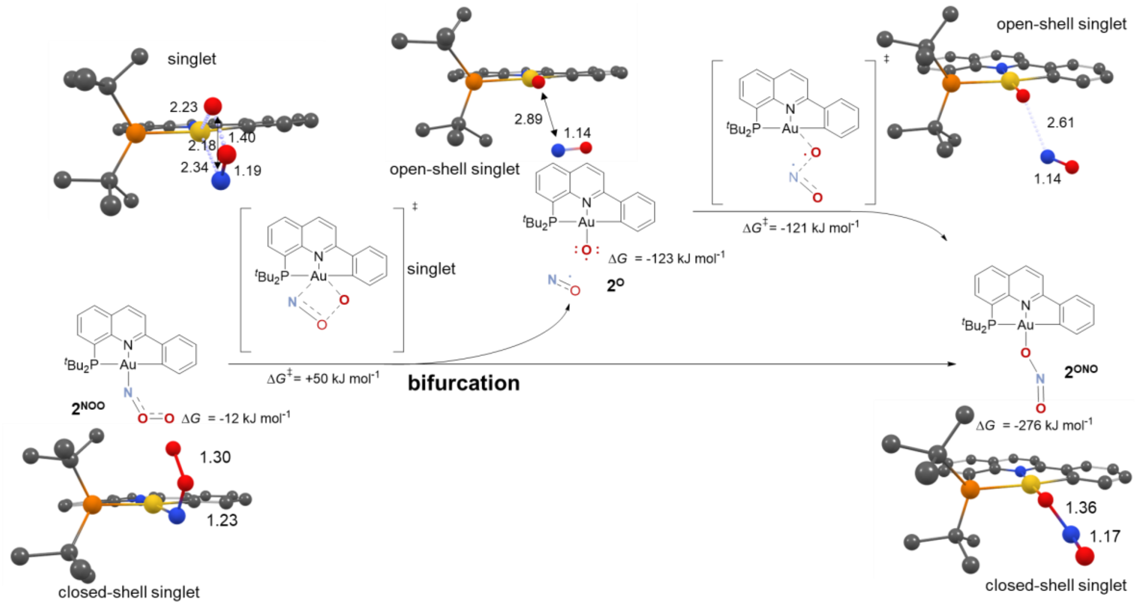

Supplementary Figure 84. Structural parameters of pertinent ground- and transition states (dioxygen addition, ZORA-PBE0-D4/def2-TZVPP//ZORA-PBE0-D4/def2-SVP).

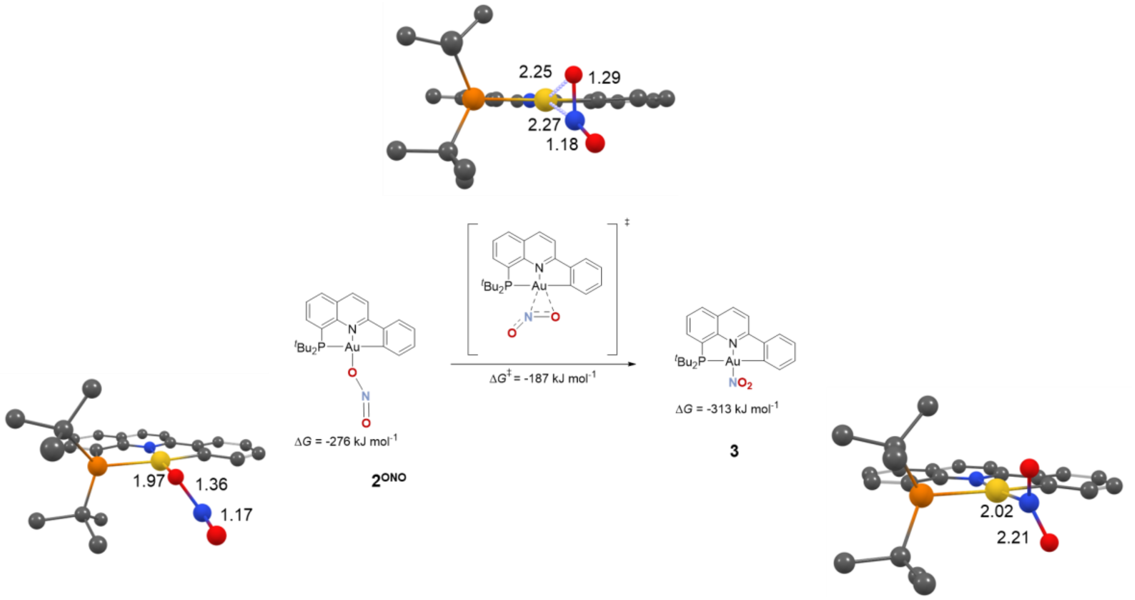

Supplementary Figure 85. Structural parameters of further pertinent ground- and transition states (dioxygen addition, ZORA-PBE0-D4/def2-TZVPP//ZORA-PBE0-D4/def2-SVP).

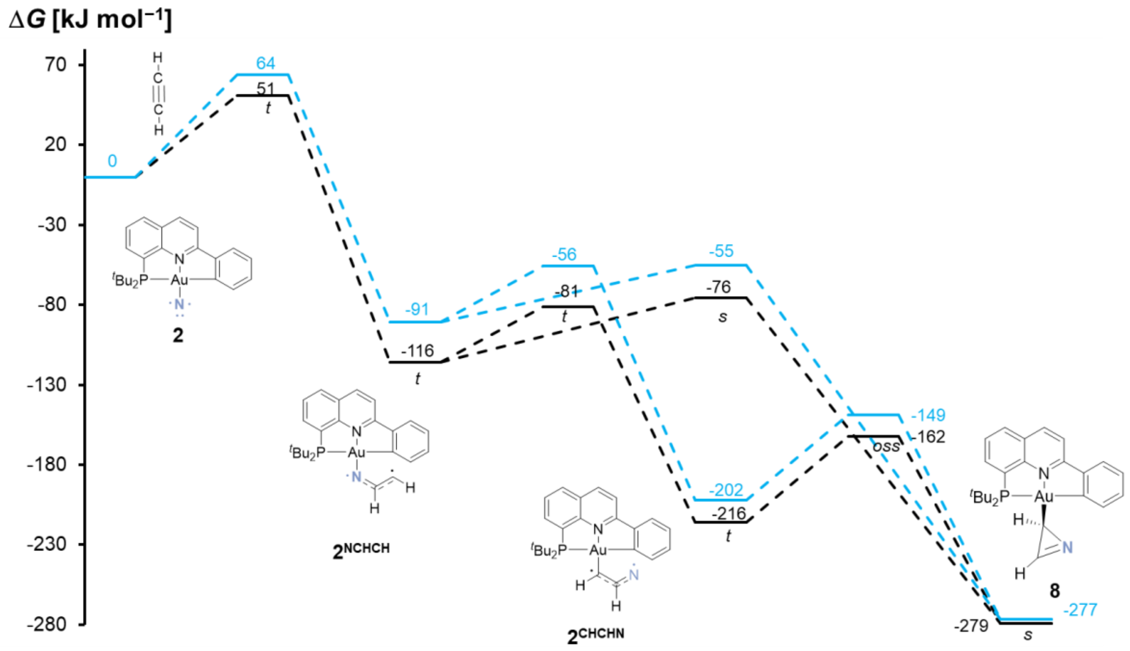

Supplementary Figure 86. Comparison of single-point approaches: Most favorable pathway(s) for acetylene activation as obtained at the ZORA-ωB97X-V/def2-TZVPP//ZORA-PBE0-D4/def2-SVP (blue) and ZORA-PBE0-D4/def2-TZVPP//ZORA-PBE0-D4/def2-SVP (black) levels of theories. Note that two pathways after the rate-determining transition state are essentially isoergic.

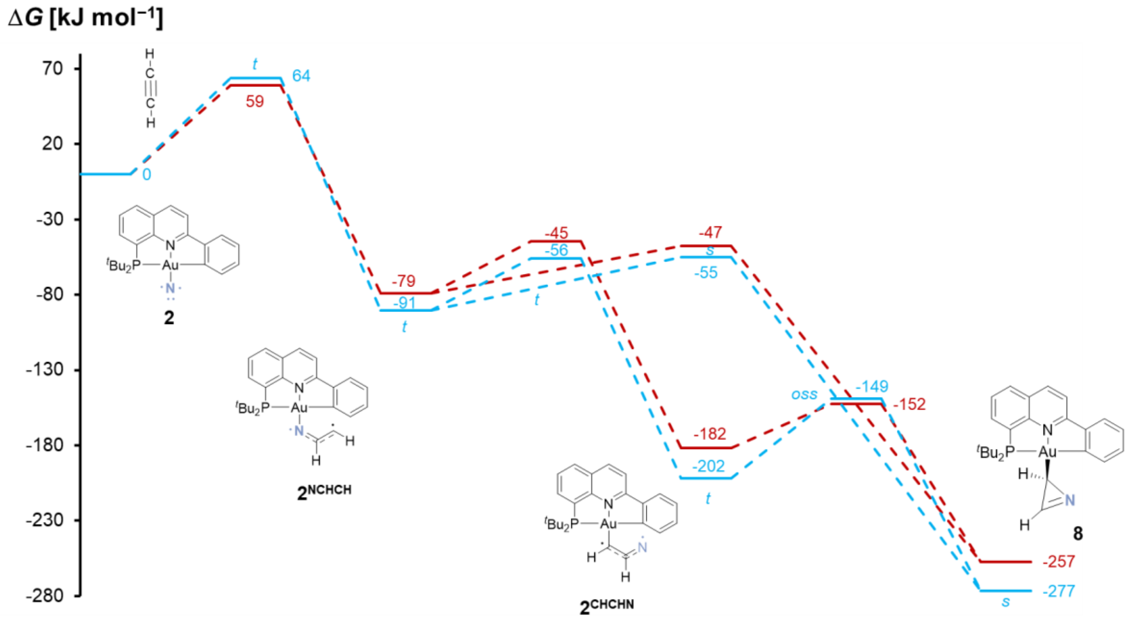

Supplementary Figure 87. Comparison of single-point approaches: Most favorable pathway(s) for acetylene activation as obtained at the ZORA-ωB97X-V/def2-TZVPP//ZORA-PBE0-D4/def2-SVP (blue) and ZORA-DLPNO-CCSD(T)/def2-TZVPP//ZORA-PBE0-D4/def2-SVP (red) levels of theories. Note that two pathways after the rate-determining transition state are essentially isoergic.

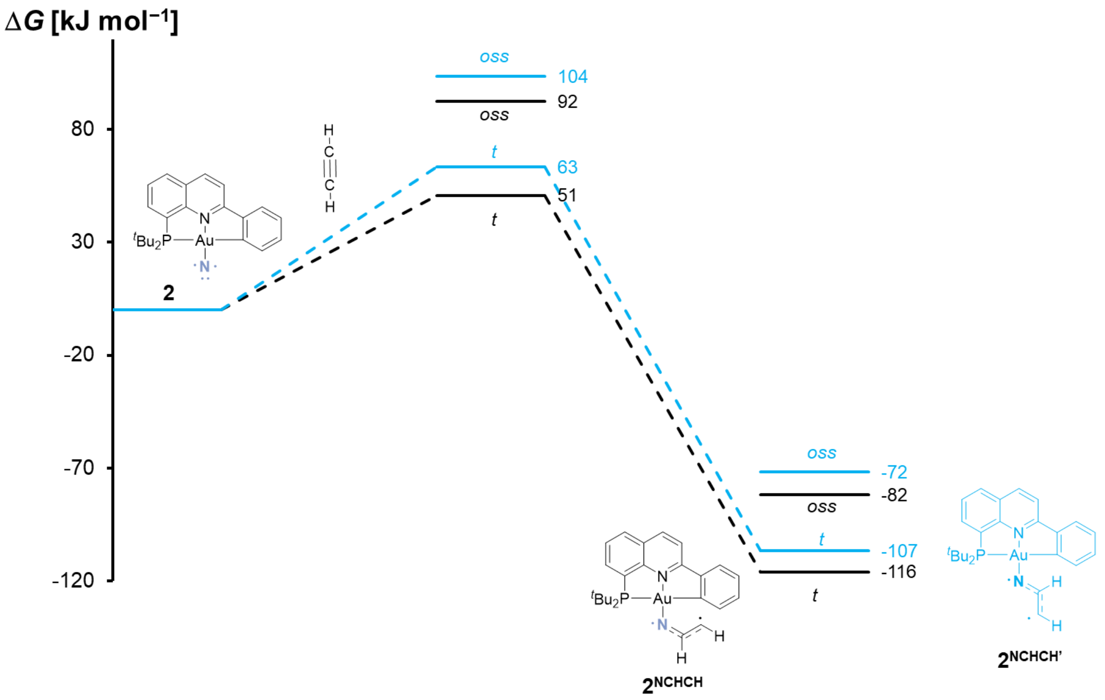

Supplementary Figure 88. Disfavored conformers for acetylene activation based on ZORA-PBE0-D4/def2-TZVPP//ZORA-PBE0-D4/def2-SVP; *oss* (open-shell singlet, antiferromagnetic coupling); *t*, triplet.

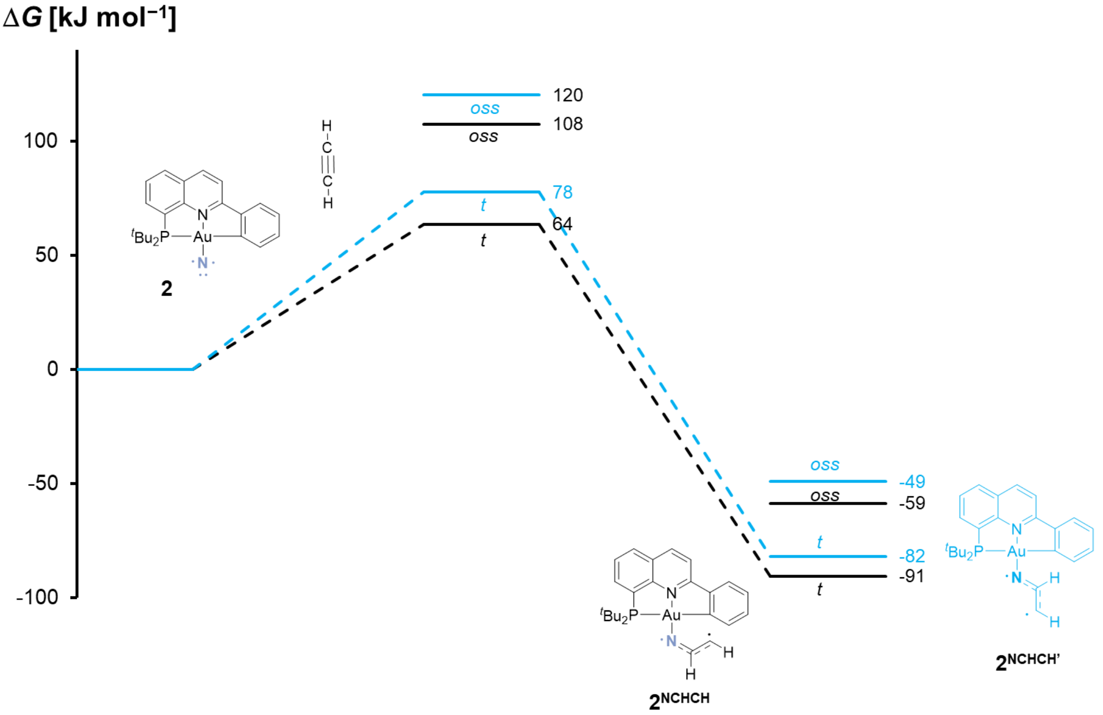

Supplementary Figure 89. Disfavored conformers for acetylene activation based on ZORA-ωB97X-V/def2-TZVPP//ZORA-PBE0-D4/def2-SVP; *oss* (open-shell singlet, antiferromagnetic coupling); *t*, triplet.

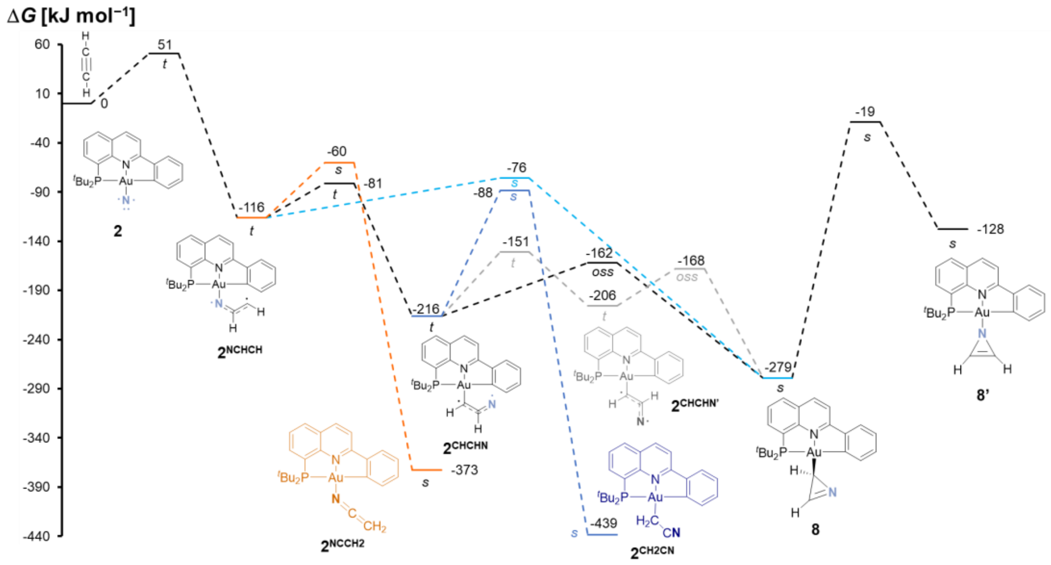

Supplementary Figure 90. Disfavored mechanisms and intermediates for azirine formation based on ZORA-PBE0-D4/def2-TZVPP//ZORA-PBE0-D4/def2-SVP; *s*, singlet (closed-shell or spin-polarized open-shell); *oss* (open-shell singlet, antiferromagnetic coupling); *t*, triplet.

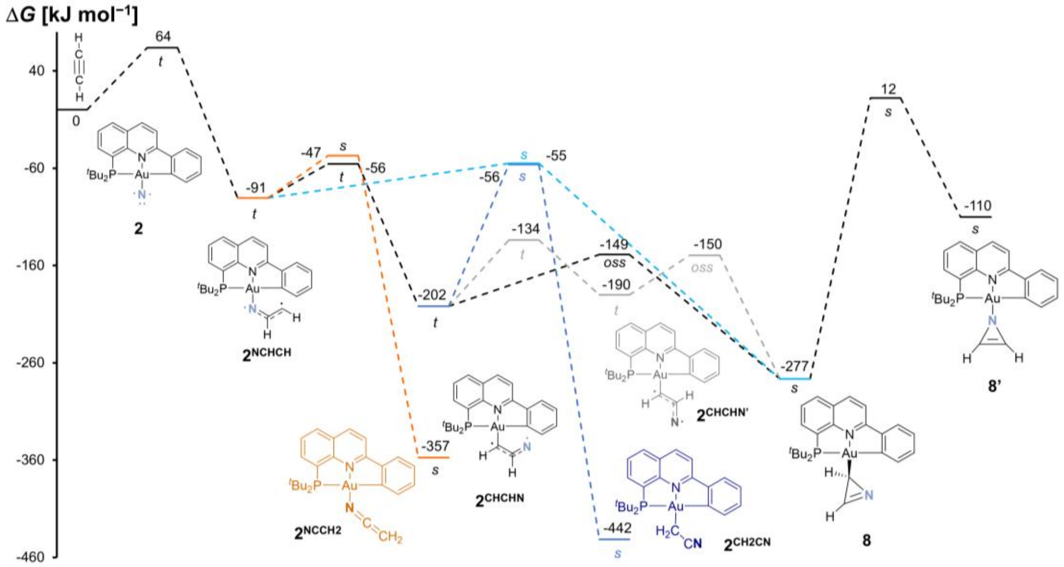

Supplementary Figure 91. Disfavored mechanisms and intermediates for azirine formation based on ZORA-ωB97X-V/def2-TZVPP//ZORA-PBE0-D4/def2-SVP; *s*, singlet (closed-shell or spin-polarized open-shell); *oss* (open-shell singlet, antiferromagnetic coupling); *t*, triplet.

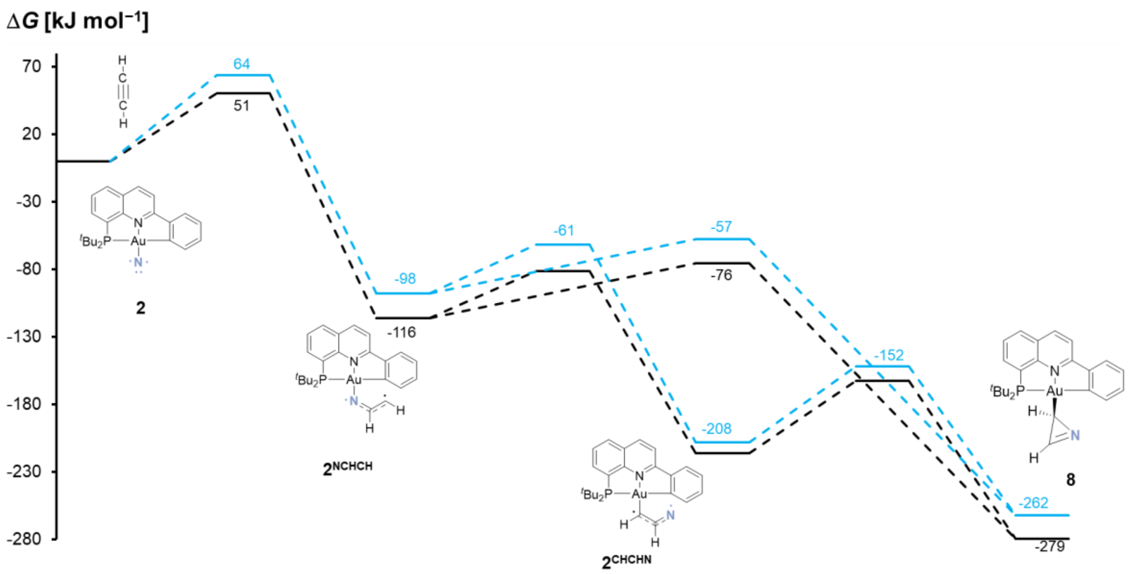

Supplementary Figure 92. The polarity of the reaction medium has only a moderate effect on computed kinetics and thermodynamics (black, ZORA-PBE0-D4/def2-TZVPP//ZORA-PBE0-D4/def2-SVP; blue, ZORA-PBE0-D4(CPCM=THF)/def2-TZVPP//ZORA-PBE0-D4/def2-SVP).

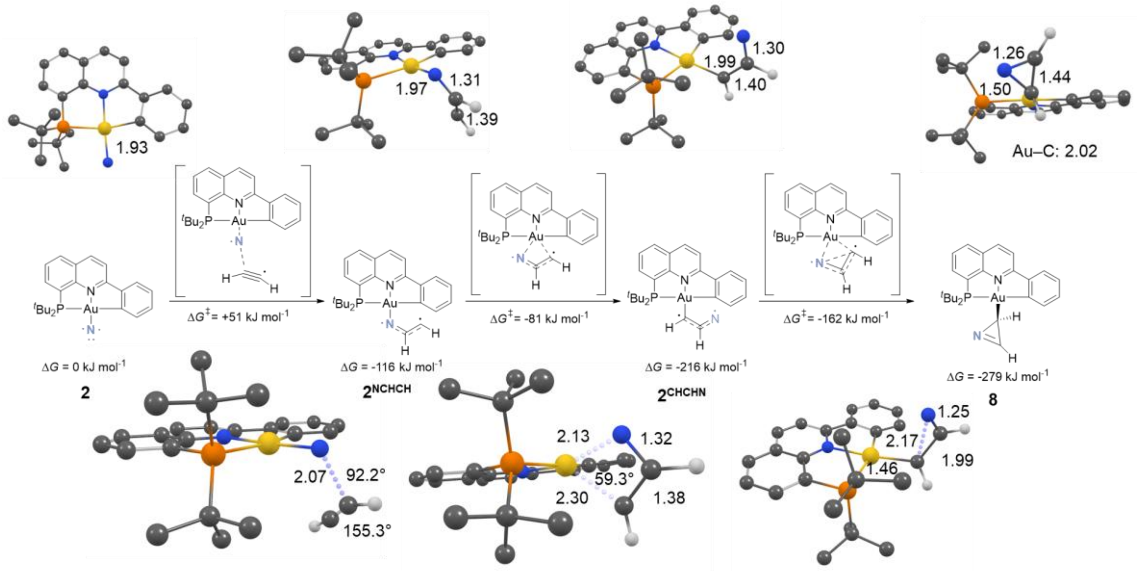

Supplementary Figure 93. Structural parameters of pertinent ground- and transition states (azirine formation, ZORA-PBE0-D4/def2-TZVPP//ZORA-PBE0-D4/def2-SVP).

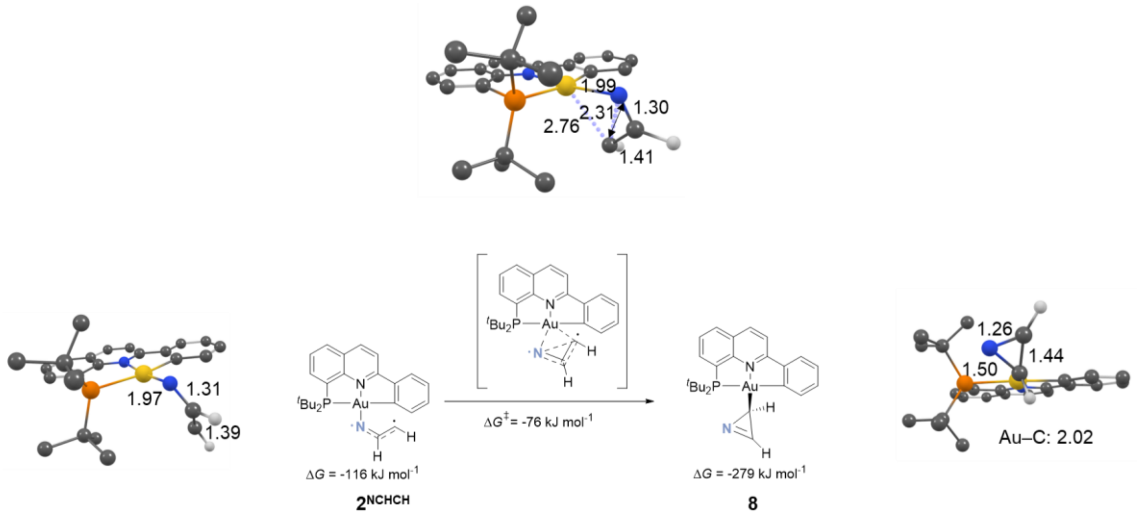

Supplementary Figure 94. Structural parameters of further pertinent ground- and transition states (azirine formation, ZORA-PBE0-D4/def2-TZVPP//ZORA-PBE0-D4/def2-SVP).

Supplementary Table 22. The conceptual DFT analysis of frontier orbitals in aura nitrene **2** suggest enhanced electrophilicity in respect to Schneider's platina-nitrene. Values are given in [eV].[42] The global electrophilicity ω is calculated based on the two α-SOMOs as well as the two energetically lowest unoccupied β-orbitals.[43]

ZORA-PBE0/ZORA-def2-TZVPP//ZORA-PBE0-D4/ZORA-def2-SVP					
	**ε SOMO1, α**	**ε SOMO2, α**	**ε LUMO1, β**	**ε LUMO2, β**	**Global Electrophilicity *ω***
**Au**	-10.036	-9.8728	-5.9034	-5.169	1.9847
**Pt**	-5.258	-5.0543	-1.3038	-0.326	1.87525
**PBE0/def2-TZVPP//PBE0-D4/def2-SVP**					
	**ε SOMO1, α**	**ε SOMO2, α**	**ε LUMO1, β**	**ε LUMO2, β**	**Global Electrophilicity *ω***
**Au**	-10.0418	-9.8778	-5.895	-5.1633	1.9914
**Pt**	-5.3136	-5.0969	-1.2946	-0.3482	1.90115

Supplementary Table 23. Comparison of partitioning methods for atomic spin-density (given in [a.u.], ZORA-PBE0-D4/def2-TZVPP//ZORA-PBE0-D4/def2-SVP) in aura-nitrene **2** as well as corresponding Cu- and Ag congeners.

**Atomic Spin Population**	**Cu**	**Ag**	**Au**
Loewdin_M	0.05	0.04	0.14
Loewdin_N	1.89	1.99	1.81
Hirshfeld_M	0.09	0.09	0.16
Hirshfeld_N	1.8	1.9	1.75
NPA_M	-0.04	-0.02	0.02
NPA_N	2.03	2.09	1.96
Spin Contamination	0.04	0.04	0.01

Supplementary Table 24. Computed (ZORA-B3LYP/ZORA-def2-TZVPP//ZORA-PBE0-D4/ZORA-def2-SVP, SARC-ZORA-TZVP on Au and Ni) *d*-vacancy in aura-nitrene **2** according to Supplementary Table 2 in *Chem. Sci.* **2023**, *14*, 6915.

**Complex**	**Cu**
**2**	1.07
Ni-complex **5** in *Chem. Sci.* **2023**, *14*, 6915.	0.86

Supplementary Table 25. Energies [Eh] of ground states (dioxygen activation). DLPNO-CCSD(T) energies of excited singlet states deemed unreliable are marked in red.

**Denominator**	***NImag***	***E***	***G***	***E*(SP, PBE0)**	***E*(SP, PBE0, CPCM)**	***E*(SP, ωB97X-V,)**	***E*(SP, CCSD(T))**
**N_2**	0	-109.41080	-109.42347	-109.54624	-109.54674	-109.63974	-109.48784
**NO**	0	-129.78311	-129.79830	-129.94437	-129.94506	-130.04284	-129.87170
**O_2**	0	-150.22109	-150.23698	-150.41115	-150.41131	-150.51411	-150.32324
**1_singlet**	0	-21128.50468	-21128.09665	-21129.78370	-21129.84487	-21130.78893	-21123.84157
**1_triplet**	0	-21128.42081	-21128.02006	-21129.70010	-21129.75987	-21130.70298	-21123.75678
**2_triplet**	0	-21019.04924	-21018.65182	-21020.20208	-21020.26198	-21021.12712	-21014.35856
**2_UKS**	0	-21018.99586	-21018.59716	-21020.15113	-21020.21241	-21021.07906	-21014.31554
**2_UKS_BS**	0	-21019.03002	-21018.63167	-21020.18416	-21020.24419	-21021.10805	-21014.29732
**2^{NOO}_singlet**	0	-21169.31402	-21168.90803	-21170.65422	-21170.71675	-21171.68063	-21164.70591
**2^{NOO}_triplet**	0	-21169.27536	-21168.87363	-21170.61557	-21170.67805	-21171.64253	-21164.65879
**2^{ONO}_singlet**	0	-21169.41536	-21169.01042	-21170.75378	-21170.81840	-21171.78621	-21164.80543
**2^{ONO}_triplet**	0	-21169.32519	-21168.92601	-21170.66366	-21170.72641	-21171.68623	-21164.71239
**3_singlet**	0	-21169.43670	-21169.02872	-21170.77101	-21170.83563	-21171.79715	-21164.81238
**3_triplet**	0	-21169.34574	-21168.94356	-21170.68015	-21170.74296	-21171.69651	-21164.71879
**2^{O}_free**	0	-21039.56048	-21039.16224	-21040.74104	-21040.80224	-21041.67689	-21034.88893
**2^{O}+NO_singlet_UKS**	0	-21169.33346	-21168.92984	-21170.67321	-21170.73341	-21171.69744	-21164.74054
**2^{O}+NO_triplet**	0	-21169.25899	-21168.86443	-21170.67967	-21170.73910	-21171.71259	-21164.74482
**2^{O}+NO_singlet_BS**	0	-21169.35135	-21168.95162	-21170.69034	-21170.75054	-21171.72549	-21164.74275
**2^{NO2}_singlet**	0	-21169.28708	-21168.88235	-21170.63097	-21170.69120	-21171.66204	-21164.68362
**2^{NO2}_triplet**	0	-21169.19572	-21168.79630	-21170.53957	-21170.59796	-21171.56034	-21164.58853
**2^{NO2}_isomer_singlet**	0	-21169.28528	-21168.88044	-21170.62924	-21170.68957	-21171.65914	-21164.68108

Supplementary Table 26. Energies [Eh] and imaginary eigenvalues [cm^{-1}] of transition states (dioxygen activation). DLPNO-CCSD(T) energies of excited singlet states deemed unreliable are marked in red.

Denominator	***Imag.***	***E***	***G***	***E*(SP, PBE0)**	***E*(SP, PBE0, CPCM)**	***E*(ωB97X-V, SP)**	***E*(SP, CCSD(T))**
**ts_1-2_singlet**	*i*147	-21128.40769	-21128.00566	-21129.69589	-21129.75450	-21130.71431	-21123.78311
**ts_1-2_singlet_BS**	*i*686	-21128.41300	-21128.01049	-21129.69885	-21129.75803	-21130.71207	-21123.76910
**ts_1-2_triplet**	*i*635	-21128.41738	-21128.01570	-21129.70107	-21129.76244	-21130.71228	-21123.76836
**ts_2-2^{NOO}_singlet_UKS2**	*i*165	-21169.23994	-21168.83964	-21170.58022	-21170.64016	-21171.60457	-21164.63954

**ts_2-2^{NOO}_singlet_BS**	*i*451	-21169.26476	-21168.86554	-21170.60459	-21170.66488	-21171.62980	-21164.65615
**ts_2-2^{NOO}_singlet_UKS**	*i*441	-21169.15828	-21168.76302	-21170.59273	-21170.66488	-21171.61731	-21164.68619
**ts_2-2^{NOO}_triplet**	*i*425	-21169.24973	-21168.85041	-21170.59026	-21170.65046	-21171.61389	-21164.64942
**ts_2^{NOO}-2^{O}_free_triplet**	*i*138	-21169.26412	-21168.86356	-21170.60421	-21170.66434	-21171.63143	-21164.65310
**ts_2^{NOO}-2^{O}+NO_singlet**	*i*304	-21169.28925	-21168.88432	-21170.62964	-21170.69011	-21171.65962	-21164.68447
**ts_2^{NOO}-2^{NO2}_singlet**	*i*901	-21169.23814	-21168.83556	-21170.58092	-21170.64096	-21171.60816	-21164.64150
**ts_2^{ONO}-3_singlet**	*i*382	-21169.38229	-21168.97701	-21170.72021	-21170.78543	-21171.74908	-21164.76811
**ts_2^{NO2}-3_singlet**	*i*1972	-21169.26464	-21168.86247	-21170.60420	-21170.66372	-21171.63217	-21164.66637
**ts_2^{O}+NO-2^{ONO}_triplet**	*i*513	-21169.33227	-21168.93159	-21170.67033	-21170.73254	-21171.69940	-21164.72124
**ts_2^{O}+NO-2^{ONO}_singlet**	*i*90	-21169.35035	-21168.95047	-21170.68957	-21170.75002	-21171.72378	-21164.73924

Supplementary Table 27. Energies [Eh] of ground states (acetylene activation). DLPNO-CCSD(T) energies of excited singlet states deemed unreliable are marked in red.

**Denominator**	***NImag***	***E***	***G***	***E*(SP, PBE0)**	***E*(SP, PBE0, CPCM)**	***E*(SP, ωB97X-V,)**	***E*(SP, CCSD(T))**
**Acetylene**	0	-77.22179	-77.21353	-77.30927	-77.31409	-77.38392	-77.23868
**2^{NCHCH}_singlet**	0	-21096.33061	-21095.90421	-21097.56323	-21097.62105	-21098.55410	-21092.00590
**2^{NCHCH}_triplet**	0	-21096.34515	-21095.91854	-21097.57645	-21097.63423	-21098.56647	-21092.04575
**2$^{NCHCH'}$_singlet**	0	-21096.32602	-21095.89960	-21097.55941	-21097.61851	-21098.55044	-21091.99678
**2$^{NCHCH'}$_triplet**	0	-21096.34066	-21095.91436	-21097.57258	-21097.63136	-21098.56284	-21092.04180
**2^{CHCHN}_singlet**	0	-21096.36980	-21095.94236	-21097.60387	-21097.66613	-21098.59808	-21092.04354
**2^{CHCHN}_triplet**	0	-21096.38117	-21095.95448	-21097.61462	-21097.67637	-21098.60895	-21092.08483
**8_singlet**	0	-21096.41220	-21095.98076	-21097.64350	-21097.70170	-21098.64214	-21092.11839
**8_triplet**	0	-21096.31491	-21095.88828	-21097.54673	-21097.60842	-21098.53830	-21092.01081
**2$^{CHCHN'}$_singlet**	0	-21096.36401	-21095.93711	-21097.59960	-21097.66489	-21098.59808	-21092.03839
**2$^{CHCHN'}$_triplet**	0	-21096.37536	-21095.94912	-21097.61026	-21097.67496	-21098.60403	-21092.08014
**2^{CH2CN}_singlet**	0	-21096.46889	-21096.03728	-21097.70429	-21097.77262	-21098.70515	-21092.18798
**2^{CH2CN}_triplet**	0	-21096.39136	-21095.96926	-21097.62317	-21097.67934	-21098.62139	-21092.09686
**8'_singlet**	0	-21096.34969	-21095.92108	-21097.58284	-21097.64211	-21098.57596	-21092.05591
**8'_triplet**	0	-21096.30982	-21095.88235	-21097.53914	-21097.59973	-21098.52822	-21091.99914
**2^{NCCH2}_singlet**	0	-21096.44348	-21096.01396	-21097.67712	-21097.73723	-21098.67101	-21092.14976
**2^{NCCH2}_triplet**	0	-21096.39141	-21095.96939	-21097.62315	-21097.67934	-21098.62146	-21092.09678

Supplementary Table 28. Energies [Eh] and imaginary eigenvalues [cm^{-1}] of transition states (acetylene activation). DLPNO-CCSD(T) energies of excited singlet states deemed unreliable are marked in red.

Denominator	***Imag.***	***E***	***G***	***E*(SP, PBE0)**	***E*(SP, PBE0, CPCM)**	***E*(ωB97X-V, SP)**	***E*(SP, CCSD(T))**
**ts_2-2^{NCHCH}_singlet**	*i*390	-21096.25467	-21095.83154	-21097.49361	-21097.55338	-21098.48748	-21091.94231
**ts_2-2^{NCHCH}_triplet**	*i*464	-21096.27070	-21095.84862	-21097.50841	-21097.56805	-21098.50315	-21091.98851
**ts_2-2$^{NCHCH'}$_singlet**	*i*503	-21096.26517	-21095.84319	-21097.50349	-21097.56412	-21098.49769	-21091.98334
**ts_2-2$^{NCHCH'}$_triplet**	*i*436	-21096.24990	-21095.82683	-21097.48928	-21097.55007	-21098.48260	-21091.93156

**ts_2^{NCHCH}-2^{CHCHN}_triplet**	*i*281	-21096.33183	-21095.90479	-21097.56369	-21097.62082	-21098.55362	-21092.03298
**ts_2^{CHCHN}-8_singlet**	*i*349	-21096.36028	-21095.93289	-21097.59487	-21097.65564	-21098.58945	-21092.07446
**ts_2^{CHCHN}-8_triplet**	*i*591	-21096.29028	-21095.86753	-21097.52348	-21097.58240	-21098.51435	-21091.99161
**ts_2^{NCHCH}-8_singlet**	*i*222	-21096.32763	-21095.90088	-21097.56117	-21097.61902	-21098.55309	-21092.03379
**ts_2^{NCHCH}-2^{NCCH2}_singlet**	*i*839	-21096.31814	-21095.89347	-21097.55320	-21097.61342	-21098.54802	-21092.02654
**ts_8'-8_singlet**	*i*878	-21096.30398	-21095.87947	-21097.53736	-21097.59716	-21098.52530	-21092.00700
**ts_2^{CHCHN}-2^{CH2CN}_singlet**	*i*1532	-21096.32607	-21095.90113	-21097.56424	-21097.62924	-21098.55171	-21092.04091
**ts_$2^{CHCHN'}$-8_singlet**	*i*138	-21096.36123	-21095.93416	-21097.59672	-21097.66061	-21098.58947	-21092.07260

Supplementary Table 29. Computed Zero-Field-Splitting (ZFS) for **2**.

**Level of Theory**	***D* [cm^{-1}]**	***D*/*E***
X2C-PBE0/X2C-TZVPPall	−26.9	0.24
ZORA-PBE0/ZORA-def2-TZVPP	−897.9	0.23
ZORA-CASSCF(18,13)/ZORA-def2-TZVPP	−12.3	0.33
NEVPT2//ZORA-CASSCF(18,13)/ZORA-def2-TZVPP	−13.8	0.31

XYZ Coordinates

2
O2
O 10.19390 2.65582 8.14721
O 10.45367 2.32154 9.25670
2
NO
N 10.19890 2.64939 8.16856
O 10.44867 2.32797 9.23534
2
N2 -109.5462448
N 10.20373 2.64317 8.18922
N 10.44383 2.33420 9.21468
4
acetylene
H 3.31144 4.33146 -8.39605
C 3.31144 4.33146 -7.32448
C 3.31144 4.33146 -6.11532
H 3.31144 4.33146 -5.04375
56
1_triplet
Au 8.42797 3.41805 6.34779
P 7.64466 1.26611 5.58385
N 6.36959 3.96925 5.66432
N 10.48169 3.06781 6.83372
N 10.85778 2.72335 7.92178
N 11.21657 2.38684 8.96585
C 5.97956 1.70487 4.96111
C 5.11252 0.80329 4.36414
H 5.40726 -0.23400 4.23123
C 3.83204 1.19110 3.91951
H 3.18285 0.45130 3.45571
C 3.40318 2.49003 4.07824
H 2.41106 2.79183 3.74524
C 4.25033 3.44828 4.67698
C 5.54802 3.05523 5.10919
C 3.88309 4.80438 4.88032
H 2.89509 5.13749 4.56465
C 4.75292 5.68623 5.46783
H 4.46292 6.72074 5.62575
C 6.03917 5.23567 5.86650
C 7.06564 6.06718 6.50442
C 8.29072 5.45663 6.84996
C 9.28761 6.21117 7.45863
H 10.24479 5.76488 7.72168
C 9.07489 7.56618 7.73317
H 9.86207 8.14843 8.21049
C 7.86669 8.17235 7.39470
H 7.70475 9.22720 7.60715
C 6.86599 7.42708 6.78036
H 5.92923 7.91529 6.51805
C 8.64858 0.63021 4.12020
C 8.42496 1.65823 3.00493
H 7.38904 1.66887 2.64925
H 9.06975 1.40371 2.15415
H 8.69176 2.67434 3.32440
C 10.13170 0.61832 4.50262
H 10.48833 1.60572 4.81489
H 10.71414 0.32135 3.62097
H 10.35894 -0.09936 5.29681
C 8.22841 -0.76371 3.65261
H 8.41213 -1.53152 4.41149
H 8.82994 -1.03030 2.77344
H 7.17946 -0.81060 3.34411
C 7.36782 0.07819 7.01844
C 6.71374 0.92672 8.11604
H 7.36733 1.73806 8.46056
H 6.49659 0.28607 8.98053
H 5.76483 1.36624 7.78484
C 6.44557 -1.09495 6.68070
H 5.42547 -0.76563 6.45942

H	6.38674	-1.74888	7.56078
H	6.80972	-1.70578	5.84888
C	8.72389	-0.44054	7.49911
H	9.18274	-1.13256	6.78514
H	8.58007	-0.99034	8.43795
H	9.43250	0.36996	7.70389

56

1_singlet

Au	8.12845	3.29112	6.55065
P	7.54069	1.14003	5.68046
N	6.37916	3.88309	5.74261
N	9.77945	2.55558	7.37121
N	10.64487	3.20624	7.91743
N	11.51319	3.71061	8.45032
C	5.90900	1.62484	5.00699
C	5.03517	0.76282	4.36210
H	5.29178	-0.28728	4.25218
C	3.80805	1.21101	3.83622
H	3.14761	0.50350	3.33933
C	3.45114	2.53583	3.94959
H	2.50541	2.89339	3.54519
C	4.31079	3.45438	4.59180
C	5.54593	2.99320	5.12340
C	4.00065	4.83144	4.73210
H	3.05908	5.20525	4.33189
C	4.86948	5.68184	5.36177
H	4.63185	6.73533	5.47194
C	6.08793	5.18342	5.88089
C	7.08901	5.97153	6.58233
C	8.23156	5.27579	7.05336
C	9.21525	5.98034	7.73043
H	10.10999	5.49585	8.11293
C	9.07765	7.35743	7.94148
H	9.86072	7.89424	8.47481
C	7.95595	8.04182	7.47818
H	7.85799	9.11233	7.64600
C	6.96054	7.34995	6.79882
H	6.08810	7.89083	6.43742
C	8.60644	0.63494	4.21488
C	8.43916	1.75987	3.18598
H	7.41606	1.82735	2.79974
H	9.10333	1.55968	2.33568
H	8.72410	2.73767	3.59593
C	10.07132	0.57886	4.66285
H	10.39053	1.50259	5.15641
H	10.69713	0.43213	3.77326
H	10.27168	-0.25157	5.34498
C	8.19068	-0.70336	3.60567
H	8.32005	-1.53673	4.30412
H	8.83493	-0.90652	2.73998
H	7.15838	-0.70063	3.23956
C	7.25554	-0.13905	7.02516
C	6.60284	0.63541	8.17718
H	7.27729	1.39230	8.59459
H	6.35965	-0.06907	8.98303
H	5.66631	1.12052	7.87335
C	6.33388	-1.28268	6.59909
H	5.30950	-0.94152	6.41696
H	6.28869	-2.00879	7.42131
H	6.69485	-1.81871	5.71562
C	8.61528	-0.68348	7.47247
H	9.05293	-1.35815	6.72892
H	8.46912	-1.26623	8.39109
H	9.32910	0.11905	7.69276

56

2^{NO2}_triplet

Au	2.26542	9.85066	5.45395
P	2.25927	7.55592	4.76575
O	0.19851	8.84061	7.21117
O	-0.33577	9.98808	6.54682
N	3.60290	10.07107	3.94441
N	0.98802	9.96553	7.00713
C	4.08189	11.39046	3.75680
C	5.00922	11.63220	2.71623
H	5.37678	12.64380	2.56443
C	5.44139	10.61723	1.91214
H	6.15675	10.80057	1.11402
C	5.36698	8.19109	1.35099
H	6.09324	8.34936	0.55551
C	4.86885	6.90591	1.59723
H	5.21154	6.06808	0.99312
C	3.93004	6.68701	2.61139
H	3.56052	5.67883	2.77038
C	3.47065	7.73887	3.39850
C	3.99585	9.04965	3.16732
C	4.95287	9.27587	2.11519
C	3.55762	12.32718	4.66663
C	2.61817	11.83535	5.65996
C	2.08997	12.68642	6.60611
H	1.40571	12.30126	7.36279
C	2.44229	14.04446	6.59880
H	2.02308	14.71930	7.34304
C	3.34883	14.54667	5.62489
H	3.61077	15.60309	5.63613
C	3.89836	13.71825	4.68142
H	4.59572	14.11773	3.94877
C	0.63971	6.98537	3.97868
C	-0.30659	6.44836	5.05419
H	-1.28363	6.26422	4.58908
H	-0.45606	7.15836	5.87111
H	0.03655	5.49536	5.47020
C	0.84505	5.92272	2.89851
H	1.41264	6.30299	2.04355
H	-0.14335	5.62542	2.52453
H	1.32985	5.01629	3.27698
C	0.05008	8.25168	3.34525
H	-0.21540	8.99867	4.10301
H	-0.86824	7.98667	2.80528
H	0.73465	8.70780	2.61807
C	3.04481	6.39298	6.02108
C	3.02254	4.92920	5.58020
H	2.00669	4.54081	5.45687
H	3.50652	4.32433	6.35827
H	3.58157	4.75805	4.65465
C	2.30625	6.55379	7.35412
H	2.37714	7.57689	7.73975
H	2.77813	5.89351	8.09310
H	1.24926	6.28014	7.29630
C	4.49117	6.87235	6.19022
H	5.09339	6.70643	5.29066

H	4.95481	6.31125	7.01159
H	4.54550	7.93717	6.45334

56

2^{NO2}_isomer_singlet

Au	2.22282	9.84432	5.43828
P	2.28159	7.55100	4.73567
O	-0.03395	10.55929	7.09950
O	0.96937	10.12721	8.02684
N	3.60600	10.10186	3.94230
N	0.78787	9.48005	6.81050
C	4.07273	11.34747	3.80676
C	5.00863	11.62888	2.78184
H	5.39007	12.63853	2.66393
C	5.41989	10.62475	1.94577
H	6.13907	10.83089	1.15398
C	5.31587	8.23096	1.26838
H	6.03516	8.41502	0.47168
C	4.80212	6.96976	1.47358
H	5.11193	6.14066	0.84094
C	3.87288	6.74343	2.50786
H	3.49358	5.73491	2.65030
C	3.44385	7.76777	3.33750
C	3.98006	9.06913	3.13582
C	4.91984	9.30506	2.09537
C	3.54589	12.31522	4.76100
C	2.58631	11.84199	5.69044
C	2.06241	12.71683	6.62882
H	1.32723	12.37392	7.35111
C	2.47638	14.05322	6.65119
H	2.05664	14.73037	7.39366
C	3.41681	14.52287	5.73563
H	3.73349	15.56357	5.75980
C	3.95275	13.65572	4.79048
H	4.69025	14.03000	4.08312
C	0.64166	6.95060	4.04433
C	-0.26614	6.57099	5.21876
H	-1.27951	6.39791	4.83404
H	-0.32463	7.36482	5.97258
H	0.05666	5.64397	5.70473
C	0.77233	5.78043	3.06934
H	1.26594	4.90741	3.50799
H	1.29754	6.06518	2.15164
H	-0.23751	5.46536	2.77499
C	0.05844	8.16099	3.30402
H	-0.15515	8.99559	3.98278
H	-0.89082	7.86960	2.83631
H	0.72105	8.51829	2.50520
C	3.13482	6.43592	5.98795
C	3.09876	4.95894	5.59819
H	2.08092	4.55598	5.58149
H	3.66087	4.38471	6.34650
H	3.57077	4.76572	4.62863
C	2.46321	6.64447	7.35088
H	2.54422	7.68264	7.69159
H	2.97566	6.01633	8.09080
H	1.40581	6.36685	7.35806
C	4.58467	6.92726	6.07036
H	5.14755	6.72998	5.15185
H	5.08818	6.40071	6.89112
H	4.64509	8.00163	6.28972

56

2^{NO2}_singlet

Au	2.29891	9.83461	5.48538
P	2.29268	7.54222	4.77825
O	0.17295	8.88226	7.18456
O	-0.17505	10.20868	6.78282
N	3.58244	10.09682	3.90893
N	1.14170	9.87601	7.12669
C	4.00163	11.35421	3.72198
C	4.89823	11.63395	2.66310
H	5.23773	12.65258	2.50077
C	5.33681	10.61359	1.86012
H	6.03444	10.81626	1.04837
C	5.33481	8.18862	1.29332
H	6.03933	8.36538	0.48193
C	4.87823	6.91827	1.56442
H	5.21975	6.07335	0.97016
C	3.95984	6.70203	2.61083
H	3.61554	5.68734	2.78948
C	3.49105	7.74221	3.39847
C	3.98053	9.05066	3.13569
C	4.89792	9.28135	2.07374
C	3.48239	12.33198	4.67250
C	2.61653	11.83856	5.67543
C	2.10392	12.69337	6.63735
H	1.45637	12.31356	7.42744
C	2.43272	14.05307	6.59838
H	2.02598	14.72626	7.35157
C	3.27945	14.55024	5.60744
H	3.53178	15.60848	5.58665
C	3.80869	13.69410	4.64688
H	4.47455	14.09508	3.88488
C	0.66980	6.98665	3.99102
C	-0.28024	6.44332	5.06031
H	-1.25484	6.25882	4.59022
H	-0.43609	7.15223	5.87727
H	0.06234	5.48948	5.47459
C	0.86986	5.93448	2.89926
H	1.35065	5.02235	3.26926
H	1.44032	6.32030	2.04854
H	-0.11902	5.64487	2.52064
C	0.08441	8.26243	3.37264
H	-0.16894	9.00695	4.13775
H	-0.84095	8.01047	2.83854
H	0.76579	8.72037	2.64370
C	3.06971	6.36977	6.02500
C	3.04473	4.90685	5.58106
H	2.02825	4.52374	5.44669
H	3.51840	4.29702	6.36151
H	3.61105	4.73521	4.65983
C	2.32234	6.53247	7.35394
H	2.39273	7.55493	7.74206
H	2.78584	5.87115	8.09715
H	1.26508	6.26043	7.28870
C	4.51622	6.84434	6.20480
H	5.12170	6.68807	5.30564
H	4.97613	6.27353	7.02155
H	4.57130	7.90597	6.48039

56

2^{O}+NO_triplet

Au	-0.27888	1.13935	-0.92883
P	0.92537	0.22128	0.89189
O	1.57759	2.32579	-4.09148
O	0.52397	2.86131	-0.93689
N	-1.18613	-0.63980	-0.97584
N	1.96345	1.60319	-3.29893
C	-2.17571	-0.79261	-1.98463
C	-2.86228	-2.02299	-2.07364
H	-3.62101	-2.14550	-2.84240
C	-2.58696	-3.04736	-1.21213
H	-3.11594	-3.99477	-1.28144
C	-1.24538	-3.88066	0.71721
H	-1.76090	-4.83816	0.66645
C	-0.25552	-3.67259	1.68917
H	-0.00803	-4.47063	2.38654
C	0.41560	-2.44917	1.77346
H	1.17658	-2.31685	2.53774
C	0.11052	-1.41501	0.89223
C	-0.89207	-1.62363	-0.10721
C	-1.58221	-2.88333	-0.19006
C	-2.35091	0.35222	-2.78707
C	-1.52611	1.50445	-2.48300
C	-1.63996	2.66277	-3.22187
H	-1.01663	3.52532	-2.99068
C	-2.56009	2.72601	-4.28011
H	-2.64775	3.63707	-4.86944
C	-3.37655	1.60771	-4.59133
H	-4.08451	1.67721	-5.41515
C	-3.28241	0.44513	-3.86864
H	-3.91532	-0.40297	-4.11988
C	0.48189	1.08034	2.49972
C	1.17736	2.44413	2.51122
H	0.76637	3.03964	3.33649
H	1.00874	3.00459	1.58238
H	2.25526	2.35460	2.68204
C	0.84623	0.26781	3.74252
H	1.91306	0.03499	3.80782
H	0.27263	-0.66263	3.80725
H	0.59064	0.86370	4.62843
C	-1.03972	1.26982	2.44692
H	-1.34597	1.92657	1.62409
H	-1.36663	1.74120	3.38265
H	-1.57268	0.31518	2.35602
C	2.74379	-0.02888	0.49077
C	3.53975	-0.62155	1.65248
H	3.60040	0.06096	2.50665
H	4.56781	-0.80083	1.31115
H	3.14515	-1.58495	1.99287
C	3.33674	1.32307	0.07818
H	2.77747	1.79399	-0.73779
H	4.36165	1.15474	-0.27664
H	3.39078	2.02947	0.91102
C	2.76154	-0.98153	-0.71116
H	2.39005	-1.98070	-0.45767
H	3.79773	-1.08778	-1.05657
H	2.17886	-0.58969	-1.55434

56

2^{O}+NO_singlet_UKS

Au	-0.19466	1.07271	-1.02016
P	0.94198	0.20329	0.86552
O	1.46670	3.11050	-2.77933
O	0.75563	2.73431	-1.05698
N	-1.17141	-0.70200	-1.02168
N	1.40829	2.35624	-3.58675
C	-2.06266	-0.87003	-2.00621
C	-2.76216	-2.09616	-2.10609
H	-3.48068	-2.24227	-2.90724
C	-2.53049	-3.08508	-1.18542
H	-3.06795	-4.03043	-1.24938
C	-1.32281	-3.87324	0.84681
H	-1.85676	-4.82165	0.81024
C	-0.39938	-3.62520	1.83746
H	-0.19509	-4.37600	2.59796
C	0.29419	-2.39803	1.87207
H	1.02288	-2.23894	2.66321
C	0.06800	-1.40938	0.92792
C	-0.89918	-1.65640	-0.08726
C	-1.59266	-2.89572	-0.13734
C	-2.24515	0.28941	-2.87548
C	-1.45092	1.42501	-2.58374
C	-1.66883	2.60969	-3.27377
H	-1.11807	3.50826	-2.99932
C	-2.62582	2.65886	-4.29282
H	-2.79105	3.58976	-4.83356
C	-3.37521	1.52728	-4.61614
H	-4.11508	1.57171	-5.41280
C	-3.19754	0.34713	-3.90130
H	-3.81555	-0.51697	-4.13889
C	0.53816	1.16596	2.42252
C	1.06730	2.59484	2.24067
H	0.59758	3.23692	2.99676
H	0.83600	3.00337	1.24725
H	2.14908	2.65503	2.39104
C	1.09146	0.54328	3.70255
H	2.18541	0.51204	3.71406
H	0.70323	-0.46473	3.88425
H	0.77960	1.16362	4.55312
C	-0.99504	1.19435	2.47518
H	-1.42599	1.68868	1.59523
H	-1.30363	1.77193	3.35581
H	-1.43088	0.19229	2.56603
C	2.74253	-0.15888	0.48569
C	3.42213	-1.07448	1.50274
H	3.42499	-0.65763	2.51430
H	4.47082	-1.20597	1.20469
H	2.97052	-2.07203	1.52967
C	3.47583	1.18294	0.37957
H	2.90466	1.91764	-0.20331
H	4.44165	1.01688	-0.11508
H	3.68712	1.61089	1.36448
C	2.72561	-0.84793	-0.88523
H	2.15421	-1.78437	-0.87525
H	3.75673	-1.09476	-1.16941
H	2.31309	-0.19755	-1.66637

56

2^{O}+NO_singlet_BS

Au	-0.28790	1.14831	-0.93119
P	0.92019	0.22470	0.89032
O	1.58719	2.34580	-4.08484
O	0.51277	2.86370	-0.93303

N	-1.20901	-0.64502	-0.99289
N	1.93380	1.60723	-3.28930
C	-2.14068	-0.78619	-1.94660
C	-2.84245	-2.01045	-2.05457
H	-3.59918	-2.12750	-2.82466
C	-2.56166	-3.03279	-1.18695
H	-3.09626	-3.97886	-1.26240
C	-1.24197	-3.89595	0.74161
H	-1.76358	-4.85014	0.68415
C	-0.27153	-3.68317	1.69541
H	-0.01493	-4.46902	2.40271
C	0.39603	-2.44416	1.76402
H	1.15719	-2.30828	2.52842
C	0.10086	-1.41326	0.88613
C	-0.89575	-1.63153	-0.10377
C	-1.57463	-2.87727	-0.17889
C	-2.33544	0.38183	-2.79453
C	-1.53594	1.50858	-2.49597
C	-1.65075	2.66879	-3.24569
H	-1.03669	3.53822	-3.01613
C	-2.56506	2.71350	-4.30308
H	-2.65819	3.62316	-4.89453
C	-3.35737	1.60505	-4.60753
H	-4.06403	1.65177	-5.43364
C	-3.24734	0.43951	-3.85734
H	-3.87142	-0.41698	-4.10617
C	0.48364	1.07656	2.50248
C	1.17880	2.44074	2.51753
H	0.77021	3.03372	3.34569
H	1.00812	3.00517	1.59148
H	2.25720	2.35072	2.68453
C	0.85087	0.26248	3.74373
H	1.91730	0.02659	3.80463
H	0.27477	-0.66633	3.81065
H	0.60039	0.85789	4.63139
C	-1.03809	1.26716	2.45501
H	-1.34736	1.92285	1.63221
H	-1.36178	1.74003	3.39110
H	-1.57201	0.31279	2.36711
C	2.73583	-0.02868	0.48394
C	3.53160	-0.64004	1.63634
H	3.59539	0.03045	2.49973
H	4.55872	-0.81838	1.29183
H	3.13389	-1.60667	1.96409
C	3.33545	1.32506	0.08655
H	2.77021	1.81509	-0.71404
H	4.35475	1.15485	-0.28294
H	3.40502	2.01608	0.93110
C	2.74823	-0.96649	-0.72970
H	2.36879	-1.96593	-0.48939
H	3.78418	-1.07653	-1.07457
H	2.17034	-0.55979	-1.56904

54

2^{O}_free_oxo

Au	2.30014	9.76262	5.44044
P	2.31102	7.50094	4.72532
N	3.57768	10.06525	3.90923
O	1.12797	9.55693	6.90744
C	3.99146	11.32890	3.73824
C	4.90782	11.62346	2.70061
H	5.24240	12.64657	2.55682
C	5.36780	10.61576	1.89439
H	6.07768	10.83187	1.09698
C	5.38318	8.19717	1.29627
H	6.09734	8.38926	0.49692
C	4.92746	6.92025	1.53834
H	5.27841	6.08801	0.93186
C	4.00033	6.68213	2.57240
H	3.65551	5.66398	2.73498
C	3.52702	7.71050	3.37186
C	4.00469	9.02808	3.13248
C	4.93313	9.27825	2.08654
C	3.43428	12.29074	4.67959
C	2.55317	11.76857	5.65405
C	1.96869	12.60569	6.59177
H	1.28939	12.20382	7.34214
C	2.25965	13.97367	6.56482
H	1.80133	14.63466	7.29907
C	3.13062	14.49836	5.60806
H	3.34953	15.56416	5.59791
C	3.71951	13.66289	4.66528
H	4.39500	14.08599	3.92388
C	0.67922	7.03261	3.92620
C	-0.35240	6.81277	5.03522
H	-1.34840	6.74839	4.57871
H	-0.37020	7.64021	5.75620
H	-0.18234	5.87547	5.57510
C	0.78408	5.80233	3.02452
H	1.43257	5.98040	2.15998
H	-0.21606	5.57531	2.63280
H	1.13396	4.91082	3.55362
C	0.28205	8.24921	3.07960
H	0.09777	9.13786	3.69508
H	-0.65038	8.01916	2.54817
H	1.03809	8.49344	2.32293
C	3.02573	6.34320	6.01941
C	3.07219	4.88278	5.57122
H	2.07318	4.46856	5.40093
H	3.53434	4.28907	6.37071
H	3.67932	4.73255	4.67220
C	2.18878	6.47607	7.29740
H	2.07797	7.51775	7.62298
H	2.69860	5.92753	8.09976
H	1.18990	6.04355	7.19309
C	4.44202	6.86713	6.28647
H	5.09490	6.77805	5.41099
H	4.88702	6.27537	7.09637
H	4.43624	7.91423	6.61589

56

2^{ONO}_triplet

Au	-0.41293	1.01630	-0.92020
P	0.81056	0.15280	0.91495
O	1.58617	4.27713	-1.66505
O	0.35756	2.83366	-0.76984
N	-1.28011	-0.76047	-0.95783
N	1.06795	3.24104	-1.85243
C	-2.23553	-0.95638	-1.98497
C	-2.89498	-2.20404	-2.06340
H	-3.62122	-2.36335	-2.85630
C	-2.63643	-3.19478	-1.15936

H	-3.14490	-4.15397	-1.21951
C	-1.38244	-3.93783	0.86100
H	-1.89027	-4.89997	0.82164
C	-0.44374	-3.68565	1.86498
H	-0.22224	-4.45265	2.60456
C	0.22043	-2.45440	1.92523
H	0.95479	-2.29078	2.70927
C	-0.04901	-1.45796	0.99314
C	-1.00705	-1.70969	-0.03630
C	-1.68284	-2.97679	-0.10202
C	-2.42335	0.16581	-2.81263
C	-1.62814	1.34839	-2.50678
C	-1.77134	2.49376	-3.26070
H	-1.18097	3.38018	-3.03925
C	-2.67649	2.51578	-4.33219
H	-2.78767	3.41812	-4.93079
C	-3.45384	1.36423	-4.64747
H	-4.14914	1.40512	-5.48400
C	-3.33829	0.21471	-3.91223
H	-3.94067	-0.65516	-4.16342
C	0.48963	1.10564	2.49752
C	1.33731	2.38163	2.46185
H	1.02811	3.02554	3.29535
H	1.19324	2.94773	1.53347
H	2.40456	2.17331	2.59452
C	0.77233	0.29488	3.76254
H	1.80092	-0.07429	3.81776
H	0.08271	-0.54887	3.87098
H	0.61716	0.94940	4.63024
C	-1.00009	1.46924	2.44850
H	-1.22830	2.15462	1.62349
H	-1.26919	1.98068	3.38171
H	-1.64211	0.58283	2.36534
C	2.60296	-0.20545	0.46739
C	3.42484	-0.66101	1.67212
H	3.53931	0.12770	2.42286
H	4.43273	-0.92578	1.32611
H	3.00882	-1.55243	2.15470
C	3.20964	1.05836	-0.15327
H	2.71211	1.33008	-1.09021
H	4.26240	0.85558	-0.38830
H	3.18255	1.92659	0.51095
C	2.56485	-1.30899	-0.59603
H	2.23397	-2.27085	-0.19033
H	3.57827	-1.44428	-0.99460
H	1.91738	-1.04500	-1.44277

56

2^{ONO}_singlet

Au	-0.41248	1.01843	-0.93342
P	0.81841	0.14985	0.90541
O	1.56700	4.28730	-1.68172
O	0.37596	2.82113	-0.77449
N	-1.30413	-0.76802	-0.97360
N	1.02766	3.26550	-1.88258
C	-2.22022	-0.94044	-1.93964
C	-2.91251	-2.17141	-2.02103
H	-3.65132	-2.31810	-2.80321
C	-2.64936	-3.16040	-1.11041
H	-3.17897	-4.11072	-1.16535
C	-1.38906	-3.94863	0.88601
H	-1.91186	-4.90315	0.84878
C	-0.44672	-3.70173	1.85956
H	-0.21286	-4.46057	2.60328
C	0.22380	-2.46360	1.89731
H	0.96923	-2.30228	2.67220
C	-0.04306	-1.46453	0.97412
C	-1.01195	-1.71775	-0.03432
C	-1.69118	-2.96517	-0.08188
C	-2.41479	0.20342	-2.81686
C	-1.63406	1.34812	-2.52900
C	-1.76657	2.48985	-3.30180
H	-1.16974	3.37540	-3.09558
C	-2.67336	2.49820	-4.36834
H	-2.77646	3.39563	-4.97654
C	-3.44359	1.37187	-4.66036
H	-4.14383	1.39114	-5.49297
C	-3.31824	0.22254	-3.88789
H	-3.92421	-0.64997	-4.12542
C	0.48446	1.08793	2.49355
C	1.32472	2.36949	2.47031
H	1.00554	3.00875	3.30357
H	1.18547	2.93799	1.54260
H	2.39204	2.16669	2.61070
C	0.76457	0.27246	3.75617
H	1.79459	-0.09226	3.81470
H	0.07838	-0.57513	3.85747
H	0.60200	0.92128	4.62679
C	-1.00676	1.44443	2.43904
H	-1.23537	2.12977	1.61385
H	-1.28287	1.95364	3.37141
H	-1.64443	0.55524	2.35208
C	2.61412	-0.19920	0.47063
C	3.43271	-0.66115	1.67535
H	3.53677	0.12097	2.43445
H	4.44472	-0.91533	1.33358
H	3.02044	-1.55985	2.14770
C	3.22014	1.07264	-0.13560
H	2.72340	1.35441	-1.06984
H	4.27359	0.87455	-0.37121
H	3.18977	1.93332	0.53800
C	2.58884	-1.29207	-0.60411
H	2.25219	-2.25827	-0.21358
H	3.60693	-1.42416	-0.99168
H	1.95254	-1.01808	-1.45617

56

2^{NOO}_triplet

Au	8.26400	3.42507	6.35612
P	7.65211	1.22986	5.62681
N	6.39358	3.93979	5.71355
N	10.13126	3.13173	6.92455
O	10.18633	2.36919	8.06673
O	9.80364	3.00761	9.12748
C	5.98522	1.67246	4.99581
C	5.11950	0.78225	4.37886
H	5.41002	-0.25618	4.24590
C	3.85092	1.18348	3.91558
H	3.20162	0.45322	3.43725
C	3.43523	2.48688	4.07215
H	2.45370	2.80316	3.72225
C	4.28298	3.43450	4.68692

C	5.56551	3.02295	5.14148
C	3.92340	4.79464	4.87344
H	2.94365	5.13249	4.53780
C	4.79138	5.67607	5.46343
H	4.51432	6.71651	5.60444
C	6.06406	5.22562	5.88863
C	7.09017	6.04893	6.51761
C	8.30188	5.40121	6.85418
C	9.32849	6.10931	7.45791
H	10.26072	5.61259	7.72071
C	9.15592	7.46991	7.73471
H	9.96051	8.02657	8.21292
C	7.96468	8.11750	7.40613
H	7.84108	9.17587	7.62636
C	6.93149	7.41274	6.79706
H	6.00792	7.93098	6.54538
C	8.67738	0.64342	4.16630
C	8.47715	1.70333	3.07671
H	7.44458	1.73941	2.71223
H	9.12403	1.46020	2.22427
H	8.76061	2.70661	3.42155
C	10.15123	0.62680	4.58823
H	10.48262	1.59215	4.98871
H	10.76225	0.41110	3.70242
H	10.37080	-0.14533	5.33066
C	8.25998	-0.73369	3.65165
H	8.38480	-1.51842	4.40520
H	8.90202	-0.99867	2.80133
H	7.22797	-0.75162	3.28611
C	7.39200	0.01239	7.04018
C	6.86585	0.86165	8.20497
H	7.61123	1.58018	8.56574
H	6.62217	0.19821	9.04484
H	5.94813	1.40384	7.94271
C	6.37174	-1.07675	6.70584
H	5.36627	-0.66966	6.55898
H	6.31929	-1.76656	7.55813
H	6.64907	-1.67113	5.82845
C	8.73004	-0.62616	7.42176
H	9.08424	-1.33022	6.66132
H	8.58548	-1.19734	8.34782
H	9.51300	0.11348	7.61650

56

2[NOO]_singlet_bs

Au	8.27834	3.37941	6.34385
P	7.63643	1.19462	5.61162
N	6.39487	3.90114	5.76923
N	10.19652	3.09453	6.76634
O	10.39245	2.71880	7.91892
O	9.39424	2.51377	8.73026
C	5.99070	1.66785	4.95219
C	5.13223	0.80329	4.29154
H	5.43286	-0.22381	4.10312
C	3.85827	1.21753	3.85447
H	3.21331	0.50853	3.33965
C	3.43075	2.50573	4.08581
H	2.44360	2.83053	3.76048
C	4.27435	3.42821	4.74359
C	5.56424	3.00587	5.16526
C	3.90482	4.77354	5.00096
H	2.91753	5.11841	4.69593
C	4.77564	5.63420	5.61653
H	4.49385	6.66559	5.80608
C	6.05996	5.17759	5.99667
C	7.09953	5.99032	6.61582
C	8.33381	5.34884	6.87175
C	9.38057	6.05734	7.44096
H	10.34031	5.58045	7.63152
C	9.20471	7.40596	7.76877
H	10.02733	7.96019	8.21812
C	7.98867	8.04412	7.52541
H	7.86133	9.09289	7.78565
C	6.93674	7.34193	6.94700
H	5.99395	7.85279	6.75998
C	8.69206	0.58997	4.17751
C	8.51065	1.63411	3.06961
H	7.49012	1.64901	2.67158
H	9.18928	1.39209	2.24185
H	8.76562	2.64601	3.41147
C	10.15700	0.57939	4.62782
H	10.49898	1.56325	4.96777
H	10.77979	0.29348	3.77049
H	10.35042	-0.14435	5.42545
C	8.29887	-0.79718	3.67030
H	8.46586	-1.57726	4.42009
H	8.93047	-1.04161	2.80619
H	7.26042	-0.84852	3.32780
C	7.28539	-0.01085	7.01462
C	6.54919	0.81447	8.07741
H	7.17205	1.62745	8.46667
H	6.30043	0.15643	8.91996
H	5.60580	1.22715	7.69801
C	6.39920	-1.18191	6.58443
H	5.39711	-0.85651	6.28740
H	6.27443	-1.84347	7.45133
H	6.83422	-1.78355	5.78104
C	8.60958	-0.52955	7.57761
H	9.14776	-1.16284	6.86446
H	8.38959	-1.14875	8.45677
H	9.25901	0.28301	7.91928

56

2[NOO]_singlet

Au	8.27832	3.37922	6.34378
P	7.63651	1.19449	5.61153
N	6.39473	3.90093	5.76951
N	10.19658	3.09420	6.76603
O	10.39260	2.71890	7.91870
O	9.39447	2.51422	8.73022
C	5.99038	1.66747	4.95290
C	5.13163	0.80268	4.29294
H	5.43209	-0.22453	4.10488
C	3.85752	1.21682	3.85619
H	3.21234	0.50764	3.34189
C	3.43016	2.50515	4.08715
H	2.44295	2.82991	3.76199
C	4.27400	3.42782	4.74439
C	5.56398	3.00555	5.16585
C	3.90456	4.77324	5.00148
H	2.91725	5.11807	4.69651
C	4.77551	5.63401	5.61672

H	4.49380	6.66546	5.80606
C	6.05985	5.17741	5.99683
C	7.09949	5.99019	6.61585
C	8.33379	5.34871	6.87173
C	9.38055	6.05722	7.44095
H	10.34026	5.58028	7.63150
C	9.20469	7.40583	7.76876
H	10.02732	7.96007	8.21809
C	7.98865	8.04400	7.52541
H	7.86130	9.09276	7.78568
C	6.93670	7.34180	6.94703
H	5.99392	7.85269	6.76005
C	8.69156	0.59055	4.17670
C	8.51026	1.63592	3.06993
H	7.48925	1.65268	2.67319
H	9.18746	1.39359	2.24109
H	8.76714	2.64715	3.41234
C	10.15661	0.57889	4.62664
H	10.49866	1.56185	4.96912
H	10.77919	0.29550	3.76832
H	10.35030	-0.14706	5.42217
C	8.29760	-0.79584	3.66811
H	8.46353	-1.57672	4.41730
H	8.92958	-1.03998	2.80419
H	7.25933	-0.84599	3.32486
C	7.28634	-0.01113	7.01462
C	6.55038	0.81387	8.07781
H	7.17314	1.62699	8.46691
H	6.30209	0.15568	8.92038
H	5.60673	1.22632	7.69880
C	6.40014	-1.18236	6.58474
H	5.39751	-0.85713	6.28938
H	6.27699	-1.84471	7.45126
H	6.83422	-1.78314	5.78019
C	8.61090	-0.52950	7.57711
H	9.14909	-1.16246	6.86371
H	8.39134	-1.14896	8.45619
H	9.25997	0.28336	7.91875

54

2_UKS_BS

Au	2.22460	9.77923	5.43055
P	2.28139	7.51099	4.71266
N	3.58960	10.06564	3.91240
N	0.95800	9.60743	6.84028
C	4.01867	11.32279	3.76323
C	4.95680	11.61608	2.74363
H	5.31046	12.63438	2.61184
C	5.41022	10.60863	1.93151
H	6.13317	10.82311	1.14534
C	5.38705	8.19491	1.30070
H	6.11163	8.37988	0.50901
C	4.90250	6.92550	1.52610
H	5.24130	6.09254	0.91366
C	3.96274	6.69329	2.55113
H	3.60155	5.67892	2.70033
C	3.50019	7.72148	3.35689
C	4.00667	9.03249	3.13259
C	4.95113	9.27560	2.09943
C	3.44902	12.28571	4.70342
C	2.51977	11.78468	5.64781
C	1.93623	12.64091	6.57005
H	1.22128	12.25853	7.29761
C	2.26991	13.99956	6.56229
H	1.81042	14.67063	7.28655
C	3.18545	14.49990	5.63624
H	3.44012	15.55782	5.63771
C	3.77523	13.64809	4.70768
H	4.48764	14.05311	3.99119
C	0.65746	6.99709	3.92560
C	-0.36064	6.75825	5.04335
H	-1.35866	6.66916	4.59550
H	-0.39317	7.58668	5.76270
H	-0.16518	5.82753	5.58638
C	0.77657	5.76485	3.02857
H	1.40802	5.95258	2.15382
H	-0.22403	5.51427	2.65272
H	1.15285	4.88390	3.55730
C	0.22926	8.20125	3.07627
H	0.03315	9.08997	3.68833
H	-0.70265	7.95173	2.55280
H	0.97587	8.45635	2.31386
C	3.03391	6.37249	6.00486
C	3.09469	4.91030	5.56453
H	2.09894	4.47790	5.42258
H	3.58828	4.32913	6.35444
H	3.68036	4.76601	4.65052
C	2.21879	6.49739	7.29689
H	2.12328	7.53586	7.63533
H	2.73571	5.93670	8.08620
H	1.21301	6.07820	7.20526
C	4.44729	6.91704	6.24538
H	5.09079	6.81585	5.36448
H	4.90830	6.34836	7.06288
H	4.43537	7.97158	6.55085

54

2_UKS

Au	2.25613	9.73992	5.48763
P	2.30355	7.49868	4.71640
N	3.57963	10.05441	3.91042
N	1.12591	9.40249	6.86618
C	4.00423	11.30977	3.74197
C	4.92869	11.60618	2.71005
H	5.27268	12.62699	2.57229
C	5.38311	10.60062	1.89844
H	6.09661	10.81472	1.10386
C	5.36721	8.18283	1.28712
H	6.08295	8.36786	0.48742
C	4.89243	6.91113	1.52180
H	5.22906	6.07762	0.90900
C	3.96509	6.67856	2.55680
H	3.60649	5.66431	2.71182
C	3.51182	7.71160	3.36237
C	4.00279	9.02489	3.12834
C	4.93355	9.26626	2.08307
C	3.44544	12.27057	4.68352
C	2.55134	11.76223	5.65243
C	1.97172	12.61567	6.58387
H	1.28238	12.23377	7.33386
C	2.27967	13.97814	6.55443
H	1.82309	14.64495	7.28444

C	3.16402	14.48879	5.60304
H	3.39804	15.55129	5.58990
C	3.74613	13.63999	4.66990
H	4.43300	14.04903	3.93138
C	0.65594	7.04505	3.93800
C	-0.37880	6.85414	5.04750
H	-1.37385	6.80253	4.58746
H	-0.38698	7.69336	5.75674
H	-0.22683	5.92097	5.59861
C	0.75488	5.79527	3.06035
H	1.40567	5.95059	2.19348
H	-0.24712	5.57173	2.67212
H	1.09319	4.91049	3.60802
C	0.27248	8.24894	3.06775
H	0.08918	9.14733	3.66821
H	-0.65792	8.01118	2.53632
H	1.03344	8.47658	2.31106
C	3.03763	6.36136	6.02156
C	3.19533	4.92179	5.52777
H	2.23522	4.45052	5.29529
H	3.65497	4.33360	6.33239
H	3.85617	4.84088	4.65855
C	2.15384	6.39826	7.27055
H	1.93691	7.42524	7.60076
H	2.69307	5.90074	8.08673
H	1.20825	5.86683	7.13351
C	4.41013	6.96357	6.34600
H	5.08230	6.96726	5.48018
H	4.87928	6.35437	7.12882
H	4.32719	7.98643	6.73377

54

2_triplet

Au	2.21651	9.78627	5.42065
P	2.27839	7.51484	4.71062
N	3.59177	10.07014	3.91487
N	0.91564	9.62965	6.83325
C	4.02145	11.32763	3.77011
C	4.96408	11.62112	2.75479
H	5.31934	12.63918	2.62542
C	5.41936	10.61399	1.94299
H	6.14569	10.82900	1.16006
C	5.39623	8.20117	1.30773
H	6.12413	8.38648	0.51917
C	4.90988	6.93190	1.53011
H	5.25091	6.09925	0.91847
C	3.96577	6.69928	2.55117
H	3.60468	5.68463	2.69921
C	3.49935	7.72661	3.35563
C	4.00906	9.03722	3.13517
C	4.95815	9.28112	2.10629
C	3.44686	12.29002	4.70865
C	2.50872	11.78887	5.64503
C	1.92041	12.64487	6.56394
H	1.19826	12.26290	7.28474
C	2.25784	14.00296	6.56198
H	1.79472	14.67388	7.28408
C	3.18169	14.50274	5.64409
H	3.43914	15.55999	5.64979
C	3.77652	13.65136	4.71799
H	4.49552	14.05635	4.00804

C	0.65900	6.98768	3.92336
C	-0.35540	6.74033	5.04307
H	-1.35249	6.63523	4.59657
H	-0.39872	7.57133	5.75856
H	-0.14760	5.81521	5.59141
C	0.78335	5.75806	3.02370
H	1.41284	5.95122	2.14870
H	-0.21629	5.50315	2.64808
H	1.16469	4.87775	3.54976
C	0.22059	8.19001	3.07654
H	0.02292	9.07785	3.68956
H	-0.71229	7.93678	2.55661
H	0.96316	8.44921	2.31155
C	3.03439	6.37384	5.99897
C	3.04961	4.90480	5.57859
H	2.04069	4.49234	5.47562
H	3.55666	4.32364	6.36001
H	3.60006	4.73368	4.64727
C	2.24954	6.53678	7.30573
H	2.21569	7.57782	7.64635
H	2.75002	5.94820	8.08531
H	1.22104	6.17249	7.23206
C	4.46556	6.88497	6.20440
H	5.09245	6.73873	5.31815
H	4.92222	6.32911	7.03310
H	4.48858	7.94866	6.47620

56

3_triplet

Au	2.23406	9.81434	5.40189
P	2.25971	7.53021	4.72849
O	-0.30021	9.53073	6.51149
O	1.20779	9.81888	8.00885
N	3.57167	10.06334	3.92356
N	0.84295	9.69966	6.86574
C	4.03916	11.38538	3.74762
C	4.97697	11.63732	2.71806
H	5.33778	12.65221	2.57288
C	5.42800	10.62821	1.91653
H	6.15091	10.82110	1.12750
C	5.38369	8.20252	1.34587
H	6.11548	8.37209	0.55785
C	4.89758	6.91340	1.58188
H	5.25372	6.08121	0.97783
C	3.95238	6.68110	2.58887
H	3.59228	5.66853	2.74488
C	3.47749	7.72649	3.37384
C	3.98713	9.04278	3.15132
C	4.95154	9.28252	2.11093
C	3.49804	12.31343	4.65585
C	2.55459	11.80717	5.64619
C	2.02928	12.66022	6.59539
H	1.34836	12.29127	7.35965
C	2.38099	14.01889	6.59174
H	1.96016	14.68913	7.33913
C	3.28413	14.52989	5.61707
H	3.54123	15.58746	5.63143
C	3.83330	13.70658	4.67144
H	4.52809	14.10905	3.93815
C	0.64194	6.96509	3.94833
C	-0.32540	6.51734	5.04683

H -1.30196 6.32616 4.58339
H -0.47823 7.28436 5.81306
H -0.00785 5.58405 5.52357
C 0.83934 5.84093 2.93052
H 1.41926 6.16840 2.06181
H -0.15033 5.54044 2.56264
H 1.30702 4.94815 3.35873
C 0.09080 8.20694 3.23566
H -0.18713 8.99335 3.94670
H -0.81672 7.92499 2.68621
H 0.80030 8.61366 2.50303
C 3.03042 6.39827 6.01794
C 3.02779 4.92997 5.59297
H 2.01554 4.53133 5.47104
H 3.51429 4.33978 6.38057
H 3.59216 4.75406 4.67121
C 2.26207 6.57020 7.33326
H 2.31000 7.59607 7.71302
H 2.72524 5.92282 8.08897
H 1.21070 6.27848 7.25773
C 4.46746 6.89723 6.20670
H 5.08614 6.73494 5.31757
H 4.92462 6.34599 7.03824
H 4.50270 7.96319 6.46798
56
3_singlet
Au 2.23028 9.82471 5.40180
P 2.25944 7.53277 4.72985
O -0.30996 9.58030 6.49029
O 1.19919 9.79672 8.00022
N 3.59156 10.08423 3.91862
N 0.83359 9.71438 6.85555
C 4.03381 11.33874 3.76067
C 4.97264 11.61340 2.73785
H 5.33369 12.62800 2.59905
C 5.41726 10.59621 1.93434
H 6.14263 10.79772 1.14700
C 5.38007 8.18208 1.32276
H 6.10901 8.35939 0.53341
C 4.88940 6.91666 1.55542
H 5.22765 6.07679 0.95226
C 3.94279 6.69921 2.57601
H 3.57588 5.68837 2.73110
C 3.47727 7.73534 3.37096
C 3.99532 9.03873 3.14365
C 4.94615 9.26954 2.11250
C 3.48528 12.31397 4.69468
C 2.55223 11.82814 5.64218
C 2.00941 12.69117 6.58269
H 1.30919 12.33312 7.33449
C 2.37767 14.04148 6.57691
H 1.94709 14.71652 7.31505
C 3.28958 14.52770 5.64042
H 3.56969 15.57908 5.64583
C 3.84774 13.66723 4.70127
H 4.56591 14.05669 3.98208
C 0.64430 6.96348 3.95080
C -0.32245 6.52010 5.05203
H -1.30150 6.33318 4.59229
H -0.46982 7.28731 5.81942
H -0.00712 5.58559 5.52790
C 0.83550 5.83629 2.93490
H 1.40859 6.16192 2.06089
H -0.15600 5.53362 2.57379
H 1.30697 4.94506 3.36222
C 0.09229 8.20410 3.23635
H -0.17915 8.99478 3.94548
H -0.81972 7.92345 2.69372
H 0.79825 8.60572 2.49752
C 3.03709 6.40187 6.01468
C 3.05493 4.93608 5.58162
H 2.04797 4.52744 5.44896
H 3.54143 4.34578 6.36911
H 3.62852 4.77242 4.66317
C 2.26233 6.55588 7.32868
H 2.28380 7.58267 7.70861
H 2.73943 5.92046 8.08582
H 1.21867 6.23845 7.25122
C 4.46663 6.91801 6.21451
H 5.09092 6.77745 5.32550
H 4.92932 6.36151 7.03944
H 4.48581 7.98029 6.49203
56
ts_1-2_triplet
Au 8.22151 3.33615 6.40519
P 7.58884 1.17034 5.62648
N 6.38588 3.90062 5.71321
N 10.03027 2.93031 7.03531
N 9.91326 2.56473 8.57672
N 10.38053 3.25753 9.36211
C 5.93125 1.64249 4.99413
C 5.04824 0.76870 4.37893
H 5.31824 -0.27566 4.24767
C 3.78886 1.19659 3.91296
H 3.12357 0.47906 3.43730
C 3.40291 2.51000 4.06159
H 2.42954 2.84647 3.70779
C 4.27049 3.44172 4.67382
C 5.54079 3.00266 5.13548
C 3.94244 4.81082 4.85325
H 2.97289 5.17075 4.51105
C 4.82735 5.67349 5.44648
H 4.57330 6.72036 5.58317
C 6.08455 5.19352 5.88509
C 7.12319 5.99120 6.52880
C 8.30847 5.31058 6.89772
C 9.34584 5.99979 7.50686
H 10.26312 5.48665 7.79122
C 9.20979 7.36881 7.76190
H 10.02420 7.90793 8.24349
C 8.04397 8.04605 7.40451
H 7.94843 9.11089 7.60668
C 7.00118 7.36268 6.78733
H 6.09863 7.90449 6.51046
C 8.62157 0.62105 4.15561
C 8.43497 1.71799 3.10040
H 7.40410 1.77553 2.73363
H 9.08234 1.49532 2.24278
H 8.72765 2.70662 3.47792
C 10.09298 0.57706 4.58380

H	10.42680	1.51622	5.04007
H	10.70707	0.40306	3.69094
H	10.30316	-0.23407	5.28634
C	8.19586	-0.73389	3.59210
H	8.33482	-1.54701	4.31225
H	8.82550	-0.96366	2.72243
H	7.15800	-0.73974	3.24245
C	7.31939	-0.07124	7.01046
C	6.68642	0.73682	8.15043
H	7.36752	1.50288	8.53966
H	6.45293	0.05572	8.97892
H	5.74684	1.21520	7.84595
C	6.38352	-1.21870	6.62621
H	5.36037	-0.87395	6.44496
H	6.34039	-1.91968	7.46988
H	6.72917	-1.78439	5.75503
C	8.67781	-0.62082	7.45274
H	9.10800	-1.30467	6.71354
H	8.53429	-1.19352	8.37799
H	9.40222	0.17179	7.67122

56

ts_1-2_singlet_BS

Au	8.22794	3.41696	6.39024
P	7.60646	1.21230	5.66820
N	6.35077	3.92245	5.73621
N	10.09105	3.22098	6.92253
N	10.21080	2.13352	8.31563
N	10.73089	2.14073	9.29816
C	5.95048	1.66052	5.00574
C	5.09779	0.77639	4.36252
H	5.39074	-0.26058	4.22634
C	3.83895	1.18111	3.87558
H	3.20021	0.45418	3.37827
C	3.42017	2.48330	4.03179
H	2.44662	2.80272	3.66294
C	4.25688	3.42658	4.66798
C	5.52888	3.01087	5.14835
C	3.89872	4.78803	4.84552
H	2.92631	5.12900	4.49219
C	4.76320	5.66793	5.44285
H	4.49143	6.71156	5.57002
C	6.02738	5.21258	5.88771
C	7.05635	6.04210	6.50622
C	8.27061	5.39914	6.84255
C	9.30110	6.11462	7.43145
H	10.23317	5.61326	7.69019
C	9.13434	7.47975	7.68736
H	9.94443	8.04406	8.14705
C	7.93993	8.12263	7.36023
H	7.81819	9.18418	7.56595
C	6.90129	7.40948	6.77016
H	5.97534	7.92468	6.52090
C	8.65488	0.64413	4.21572
C	8.50935	1.74566	3.15866
H	7.48404	1.83126	2.78155
H	9.15890	1.50524	2.30742
H	8.82520	2.72569	3.53952
C	10.11599	0.57856	4.67727
H	10.43813	1.50200	5.17420
H	10.75104	0.43310	3.79401

H	10.30432	-0.25981	5.35353
C	8.21944	-0.70202	3.63924
H	8.28871	-1.51347	4.37146
H	8.89038	-0.95990	2.80922
H	7.20434	-0.67711	3.22936
C	7.30056	-0.04801	7.03704
C	6.77583	0.77403	8.22092
H	7.52050	1.48829	8.59017
H	6.52905	0.09494	9.04728
H	5.86113	1.32523	7.96700
C	6.26224	-1.10773	6.66512
H	5.26605	-0.67838	6.52004
H	6.18919	-1.81893	7.49826
H	6.53683	-1.68401	5.77487
C	8.61712	-0.73368	7.41020
H	8.95013	-1.43314	6.63620
H	8.45839	-1.31572	8.32722
H	9.42305	-0.02159	7.60808

56

ts_1-2_singlet

Au	8.27976	3.28826	6.30189
P	7.44172	1.11142	5.79759
N	6.45087	3.92058	5.55318
N	9.82705	2.46460	6.88503
N	11.50496	3.52786	7.58484
N	12.52541	3.30541	7.92866
C	5.83235	1.63428	5.10295
C	4.88297	0.74959	4.61389
H	5.07614	-0.31961	4.62152
C	3.65285	1.20250	4.09834
H	2.93196	0.47923	3.72315
C	3.36716	2.54925	4.07031
H	2.41868	2.90723	3.67254
C	4.30297	3.48797	4.55786
C	5.54186	3.02541	5.07794
C	4.07012	4.88832	4.55560
H	3.12890	5.26839	4.16066
C	5.01577	5.75099	5.04178
H	4.84150	6.82277	5.03993
C	6.23392	5.23841	5.55146
C	7.32288	6.03048	6.10120
C	8.46004	5.31935	6.55333
C	9.52463	6.01773	7.10472
H	10.40415	5.49437	7.46445
C	9.47332	7.41134	7.20246
H	10.31604	7.94745	7.63647
C	8.35791	8.11660	6.75187
H	8.32644	9.20130	6.83085
C	7.28386	7.42817	6.20282
H	6.41481	7.98406	5.85616
C	8.42532	0.29472	4.41943
C	8.62806	1.39548	3.37031
H	7.67690	1.79179	2.99385
H	9.16796	0.96652	2.51644
H	9.22988	2.22512	3.75882
C	9.78000	-0.14612	4.97702
H	10.27320	0.65724	5.54201
H	10.43218	-0.40835	4.13415
H	9.69656	-1.03223	5.61334
C	7.69557	-0.88900	3.78175

H	7.46401	-1.68484	4.49631
H	8.35631	-1.32266	3.02014
H	6.77670	-0.58527	3.27035
C	7.09114	0.13970	7.36923
C	6.26012	1.08846	8.24270
H	6.81714	1.99508	8.50853
H	6.01213	0.57041	9.17784
H	5.31641	1.37948	7.76620
C	6.30649	-1.14655	7.10764
H	5.32162	-0.95911	6.66792
H	6.13455	-1.64230	8.07184
H	6.85505	-1.85428	6.47821
C	8.41436	-0.17666	8.07049
H	8.97115	-0.97233	7.56762
H	8.19010	-0.52602	9.08644
H	9.06133	0.70762	8.15236

56

ts_2^{NO2}-3_singlet

Au	-0.50040	1.14022	0.82946
P	-0.43949	-1.15366	0.13134
O	-2.75896	1.82738	2.50107
O	-1.57343	1.22851	3.50133
N	0.86843	1.40181	-0.67987
N	-1.92624	0.82856	2.24234
C	1.32425	2.65103	-0.82078
C	2.25196	2.93468	-1.85270
H	2.62444	3.94689	-1.97789
C	2.66842	1.92899	-2.68474
H	3.38208	2.13721	-3.48099
C	2.58800	-0.47209	-3.34341
H	3.30273	-0.28745	-4.14409
C	2.08910	-1.73713	-3.12512
H	2.40652	-2.56825	-3.75132
C	1.16519	-1.96504	-2.08619
H	0.79725	-2.97650	-1.93409
C	0.72711	-0.93847	-1.26426
C	1.24926	0.36683	-1.47905
C	2.18310	0.60486	-2.52432
C	0.80057	3.61657	0.13842
C	-0.14215	3.13549	1.08038
C	-0.65546	3.99819	2.03554
H	-1.37471	3.64243	2.76979
C	-0.24893	5.33699	2.05830
H	-0.65775	6.00782	2.81251
C	0.67176	5.81726	1.12765
H	0.98169	6.86001	1.15220
C	1.19869	4.95967	0.16834
H	1.92245	5.34333	-0.54828
C	-2.07669	-1.74489	-0.57350
C	-3.00324	-2.09706	0.59444
H	-4.01447	-2.26250	0.20091
H	-3.06089	-1.29159	1.33598
H	-2.69859	-3.02039	1.09895
C	-1.94852	-2.92896	-1.53178
H	-1.47595	-3.80387	-1.07439
H	-1.40325	-2.66383	-2.44364
H	-2.95796	-3.23197	-1.83983
C	-2.63594	-0.53704	-1.33651
H	-2.84213	0.31140	-0.67256
H	-3.58570	-0.82102	-1.80774
H	-1.96150	-0.20151	-2.13476
C	0.39752	-2.27080	1.39173
C	0.35243	-3.74947	1.00892
H	-0.66863	-4.14409	0.98927
H	0.90625	-4.32435	1.76286
H	0.82727	-3.95133	0.04253
C	-0.28308	-2.05214	2.74886
H	-0.19155	-1.01510	3.09145
H	0.21474	-2.68566	3.49414
H	-1.34373	-2.31777	2.74727
C	1.85002	-1.78900	1.48211
H	2.41721	-1.99334	0.56780
H	2.34484	-2.31561	2.30809
H	1.91607	-0.71421	1.69785

56

ts_2^{O}+NO-2^{ONO}_triplet

Au	-0.44788	1.08565	0.94915
P	-0.57107	-1.16152	0.19259
O	-0.03386	0.38782	4.53934
O	-1.52725	0.77734	2.53697
N	0.69008	1.39649	-0.67545
N	-0.60561	1.19694	3.92510
C	1.13781	2.64995	-0.84374
C	1.96470	2.94391	-1.95396
H	2.32974	3.95685	-2.09509
C	2.29630	1.94986	-2.83626
H	2.93209	2.16753	-3.69355
C	2.12676	-0.43527	-3.53191
H	2.75917	-0.23737	-4.39612
C	1.62853	-1.69852	-3.30175
H	1.86108	-2.51343	-3.98397
C	0.81389	-1.94429	-2.17928
H	0.43306	-2.95071	-2.02448
C	0.49237	-0.93811	-1.28167
C	0.99583	0.36926	-1.52239
C	1.82051	0.62551	-2.65077
C	0.70321	3.60023	0.16893
C	-0.11511	3.08002	1.19837
C	-0.60695	3.91513	2.18839
H	-1.24620	3.52083	2.97542
C	-0.27212	5.27382	2.16789
H	-0.65650	5.93219	2.94566
C	0.54743	5.79304	1.16420
H	0.80273	6.85076	1.16227
C	1.03541	4.96189	0.16205
H	1.66723	5.38034	-0.61939
C	-2.29359	-1.61864	-0.39718
C	-3.27539	-1.35029	0.75027
H	-4.29562	-1.50691	0.37729
H	-3.21198	-0.32132	1.12376
H	-3.12940	-2.02469	1.59847
C	-2.40445	-3.07029	-0.86143
H	-2.24032	-3.78022	-0.04412
H	-1.71678	-3.30692	-1.68078
H	-3.42158	-3.24099	-1.23792
C	-2.59829	-0.66232	-1.55638
H	-2.49800	0.39012	-1.25988
H	-3.63893	-0.81204	-1.87050
H	-1.95764	-0.84136	-2.42678
C	0.29829	-2.34333	1.35986

C	0.71459	-3.65635	0.69582
H	-0.12735	-4.20692	0.26482
H	1.16241	-4.30079	1.46361
H	1.47620	-3.50438	-0.07642
C	-0.62507	-2.60705	2.55276
H	-1.01204	-1.68026	2.99324
H	-0.04861	-3.12489	3.32984
H	-1.46788	-3.25481	2.28827
C	1.55158	-1.59396	1.83093
H	2.19509	-1.29017	0.99537
H	2.14119	-2.26454	2.46918
H	1.30447	-0.71209	2.43360

56

ts_2^{O}+NO-2^{ONO}_singlet

Au	-0.38478	1.05781	-0.87897
P	0.82215	0.15787	0.95719
O	1.86796	3.59844	-3.54839
O	0.42157	2.76986	-0.92187
N	-1.31766	-0.73069	-0.90596
N	1.80180	2.54371	-3.12324
C	-2.25369	-0.88163	-1.85415
C	-2.96644	-2.10147	-1.93693
H	-3.72700	-2.22610	-2.70203
C	-2.69071	-3.11028	-1.05209
H	-3.23333	-4.05311	-1.10844
C	-1.36923	-3.95066	0.88499
H	-1.89900	-4.90131	0.84647
C	-0.39240	-3.72953	1.83020
H	-0.13856	-4.50513	2.54970
C	0.28526	-2.49484	1.87444
H	1.05074	-2.35253	2.63322
C	-0.00549	-1.47636	0.98054
C	-1.00854	-1.70384	-0.00099
C	-1.69779	-2.94522	-0.05138
C	-2.43989	0.27094	-2.72481
C	-1.62582	1.39402	-2.45371
C	-1.73083	2.53981	-3.22690
H	-1.10308	3.40488	-3.02048
C	-2.65130	2.57379	-4.27922
H	-2.73762	3.47237	-4.88842
C	-3.45827	1.46861	-4.55663
H	-4.16941	1.50690	-5.37931
C	-3.35698	0.31720	-3.78393
H	-3.99203	-0.53697	-4.01202
C	0.40075	1.03126	2.56231
C	1.09200	2.39744	2.55413
H	0.68296	3.00233	3.37341
H	0.91901	2.94716	1.61974
H	2.17075	2.31326	2.72103
C	0.78042	0.23588	3.81203
H	1.84795	0.00320	3.86687
H	0.20694	-0.69293	3.89771
H	0.53665	0.84354	4.69324
C	-1.12196	1.21723	2.52456
H	-1.44002	1.85977	1.69469
H	-1.43941	1.70303	3.45614
H	-1.65380	0.26024	2.45512
C	2.63228	-0.11330	0.53558
C	3.42608	-0.75994	1.66994
H	3.50017	-0.11009	2.54798
H	4.44996	-0.94123	1.31740
H	3.01894	-1.72944	1.97639
C	3.25103	1.23683	0.15731
H	2.68511	1.75171	-0.62745
H	4.26316	1.05547	-0.22650
H	3.34339	1.90929	1.01451
C	2.62199	-1.02751	-0.69605
H	2.21104	-2.01975	-0.47793
H	3.65548	-1.16264	-1.03934
H	2.05952	-0.58734	-1.52972

56

ts_2^{ONO}-3_singlet

Au	-0.38293	1.01451	-0.99911
P	0.85528	0.18057	0.86008
O	1.69632	3.57556	-1.96417
O	0.03189	3.20509	-0.68559
N	-1.29090	-0.75537	-0.99595
N	0.92822	2.79784	-1.51240
C	-2.21737	-0.93940	-1.95290
C	-2.90232	-2.17489	-2.01971
H	-3.64613	-2.32775	-2.79611
C	-2.62797	-3.15634	-1.10555
H	-3.15296	-4.10964	-1.14887
C	-1.35457	-3.92058	0.88870
H	-1.87571	-4.87632	0.86155
C	-0.41118	-3.66166	1.85756
H	-0.17489	-4.41134	2.60972
C	0.25713	-2.42250	1.87976
H	0.99944	-2.25158	2.65465
C	-0.00949	-1.43400	0.94445
C	-0.98467	-1.69781	-0.05271
C	-1.66311	-2.94674	-0.08641
C	-2.43447	0.19726	-2.82722
C	-1.65305	1.34114	-2.55027
C	-1.81517	2.50174	-3.28903
H	-1.23100	3.39337	-3.07079
C	-2.75240	2.51967	-4.32786
H	-2.88362	3.42874	-4.91294
C	-3.51873	1.38974	-4.62192
H	-4.24082	1.41915	-5.43530
C	-3.36710	0.22867	-3.87375
H	-3.97696	-0.64261	-4.10592
C	0.47642	1.12364	2.43911
C	1.31568	2.40500	2.45040
H	0.97330	3.03594	3.28077
H	1.19735	2.98738	1.52927
H	2.37887	2.20212	2.61757
C	0.72152	0.30815	3.70918
H	1.74992	-0.05507	3.79833
H	0.03341	-0.53952	3.79310
H	0.53359	0.95831	4.57361
C	-1.01307	1.47893	2.34423
H	-1.21867	2.16556	1.51483
H	-1.31395	1.98420	3.27108
H	-1.64636	0.58859	2.23867
C	2.66169	-0.19907	0.48724
C	3.41156	-0.76044	1.69491
H	3.49984	-0.03110	2.50691
H	4.43226	-1.01480	1.38028
H	2.96394	-1.67903	2.08871

C	3.35347	1.07546	-0.00783
H	2.89913	1.46916	-0.92129
H	4.39658	0.82828	-0.24320
H	3.37053	1.87400	0.73895
C	2.64228	-1.22435	-0.65287
H	2.22538	-2.18984	-0.34612
H	3.67382	-1.39700	-0.98507
H	2.08073	-0.85899	-1.52276

56

ts_2^{NOO}-2^{NO2}_singlet

Au	-0.38115	1.11833	0.94898
P	-0.41714	-1.16952	0.19951
O	-2.99590	1.33264	1.62102
O	-2.39096	1.66192	2.86501
N	0.88529	1.38575	-0.64408
N	-1.46170	0.83070	2.59047
C	1.34395	2.63210	-0.80349
C	2.22717	2.91609	-1.87256
H	2.60048	3.92661	-2.00813
C	2.60318	1.91186	-2.72511
H	3.28336	2.11876	-3.55042
C	2.47903	-0.48348	-3.39007
H	3.15840	-0.29834	-4.22082
C	1.97866	-1.74493	-3.15903
H	2.25776	-2.57399	-3.80587
C	1.10006	-1.97263	-2.08118
H	0.72432	-2.98029	-1.92699
C	0.71203	-0.95028	-1.22901
C	1.23048	0.35257	-1.46193
C	2.11773	0.59082	-2.54719
C	0.86533	3.60066	0.17571
C	-0.02121	3.11889	1.16716
C	-0.53204	3.98950	2.11578
H	-1.22726	3.64234	2.87750
C	-0.16405	5.33961	2.09180
H	-0.56941	6.01857	2.84049
C	0.71599	5.81914	1.12277
H	0.99996	6.86947	1.11330
C	1.22849	4.95370	0.16255
H	1.90756	5.34102	-0.59461
C	-2.08521	-1.68135	-0.49795
C	-3.05514	-1.91760	0.66282
H	-4.06420	-2.03659	0.24734
H	-3.09013	-1.07040	1.35700
H	-2.82489	-2.83381	1.21557
C	-2.02078	-2.91710	-1.39639
H	-1.62649	-3.80063	-0.88474
H	-1.43796	-2.73933	-2.30585
H	-3.04291	-3.15922	-1.71567
C	-2.54653	-0.47469	-1.32712
H	-2.69783	0.41777	-0.70914
H	-3.51011	-0.71713	-1.79338
H	-1.84312	-0.23528	-2.13449
C	0.44984	-2.32227	1.40921
C	0.63824	-3.74343	0.88098
H	-0.31385	-4.25207	0.69947
H	1.17266	-4.32610	1.64295
H	1.24456	-3.78180	-0.02974
C	-0.34296	-2.35358	2.72056
H	-0.56252	-1.35328	3.10843
H	0.25920	-2.87906	3.47294
H	-1.28724	-2.89669	2.62488
C	1.81605	-1.67108	1.65922
H	2.42885	-1.61988	0.75176
H	2.36302	-2.27363	2.39556
H	1.72104	-0.65936	2.07420

56

ts_2^{NOO}-2^{O}+NO_singlet

Au	-0.29224	1.01444	-1.06326
P	0.90304	0.16288	0.80605
O	1.07374	3.30070	-1.58945
O	0.04527	3.18462	-0.64482
N	-1.30040	-0.73906	-0.99059
N	1.34095	2.28991	-2.15777
C	-2.25807	-0.89357	-1.91498
C	-2.98617	-2.10644	-1.95948
H	-3.76253	-2.23685	-2.70769
C	-2.70476	-3.10018	-1.05981
H	-3.25830	-4.03785	-1.08701
C	-1.35991	-3.91468	0.87133
H	-1.90209	-4.85905	0.86294
C	-0.36398	-3.68456	1.79410
H	-0.10733	-4.44652	2.52703
C	0.32986	-2.45849	1.79814
H	1.10768	-2.31033	2.54258
C	0.04025	-1.45603	0.88570
C	-0.98309	-1.69369	-0.07036
C	-1.69190	-2.92598	-0.08087
C	-2.45911	0.25179	-2.78823
C	-1.61910	1.36604	-2.55857
C	-1.77856	2.52685	-3.29840
H	-1.16179	3.40218	-3.10556
C	-2.76009	2.57703	-4.29362
H	-2.88519	3.48799	-4.87705
C	-3.57909	1.47478	-4.54364
H	-4.33738	1.52534	-5.32232
C	-3.43640	0.31496	-3.79108
H	-4.09210	-0.53214	-3.98436
C	0.45272	1.09717	2.37527
C	1.27490	2.38784	2.43496
H	0.90189	2.99790	3.26786
H	1.16629	2.98212	1.52012
H	2.33641	2.19781	2.62730
C	0.65164	0.26798	3.64464
H	1.68117	-0.07646	3.78051
H	-0.02131	-0.59518	3.68281
H	0.40794	0.90187	4.50721
C	-1.03531	1.44037	2.22744
H	-1.21641	2.13362	1.39781
H	-1.37513	1.93423	3.14696
H	-1.65647	0.54614	2.08928
C	2.73206	-0.18365	0.52625
C	3.45738	-0.64406	1.79187
H	3.48149	0.13276	2.56281
H	4.49896	-0.87155	1.53013
H	3.03328	-1.55663	2.22307
C	3.39461	1.09122	-0.00759
H	3.00635	1.37097	-0.99305
H	4.46817	0.89901	-0.13019
H	3.28901	1.94728	0.66490

C	2.79896	-1.26715	-0.55537
H	2.41365	-2.23177	-0.20816
H	3.84758	-1.41003	-0.84592
H	2.24890	-0.97591	-1.45988

56

ts_2^{NOO}-2^{O}_free_triplet

Au	-0.36516	1.13718	0.90987
P	-0.43460	-1.15428	0.20505
O	-2.14393	1.13748	3.09059
O	-2.49615	1.37464	1.69894
N	0.87329	1.39143	-0.67207
N	-0.90313	0.86066	2.97444
C	1.33327	2.63957	-0.84616
C	2.21574	2.90673	-1.91901
H	2.58865	3.91605	-2.06561
C	2.58963	1.89118	-2.75909
H	3.26928	2.08689	-3.58751
C	2.45784	-0.51000	-3.39978
H	3.13555	-0.33374	-4.23379
C	1.95272	-1.76752	-3.15810
H	2.22574	-2.60228	-3.80018
C	1.07828	-1.98386	-2.07469
H	0.69905	-2.98856	-1.91048
C	0.69891	-0.95439	-1.22679
C	1.21749	0.34440	-1.47614
C	2.10232	0.57291	-2.56538
C	0.85230	3.61485	0.11933
C	-0.02714	3.12821	1.11254
C	-0.56146	3.98175	2.06247
H	-1.25093	3.61318	2.81889
C	-0.20908	5.33552	2.03629
H	-0.62619	6.01110	2.78166
C	0.66742	5.82736	1.06757
H	0.93430	6.88214	1.06018
C	1.19744	4.97342	0.10722
H	1.87459	5.37034	-0.64697
C	-2.10801	-1.65106	-0.49317
C	-3.11558	-1.76516	0.65478
H	-4.11939	-1.85220	0.21912
H	-3.11368	-0.87815	1.29756
H	-2.95079	-2.65739	1.26483
C	-2.07476	-2.94985	-1.29839
H	-1.74579	-3.80872	-0.70461
H	-1.45125	-2.87224	-2.19489
H	-3.09534	-3.16788	-1.63946
C	-2.50669	-0.48402	-1.40651
H	-2.63244	0.44879	-0.84422
H	-3.47404	-0.71641	-1.86997
H	-1.78527	-0.32064	-2.21660
C	0.42340	-2.31121	1.41484
C	0.82750	-3.65506	0.80822
H	-0.02334	-4.21802	0.41148
H	1.27426	-4.26735	1.60256
H	1.58538	-3.54942	0.02521
C	-0.48546	-2.53599	2.62673
H	-0.88075	-1.59949	3.03544
H	0.10804	-3.01314	3.41710
H	-1.32041	-3.20531	2.40002
C	1.68279	-1.55062	1.85170
H	2.34068	-1.31168	1.00668
H	2.25418	-2.18513	2.54137
H	1.44073	-0.62501	2.38688

56

ts_2-2^{NOO}_triplet

Au	8.24230	3.34411	6.39271
P	7.59861	1.18110	5.61552
N	6.38762	3.90406	5.71041
N	10.01218	2.94328	7.03167
O	9.79725	3.11028	8.95645
O	9.80991	2.05106	9.52145
C	5.93297	1.64513	4.99965
C	5.04709	0.76639	4.39629
H	5.31979	-0.27732	4.26385
C	3.78134	1.18962	3.94254
H	3.11320	0.46879	3.47592
C	3.39287	2.50249	4.09051
H	2.41502	2.83450	3.74486
C	4.26373	3.43900	4.69101
C	5.53911	3.00474	5.14231
C	3.93607	4.80860	4.86901
H	2.96289	5.16647	4.53498
C	4.82623	5.67478	5.44993
H	4.57195	6.72199	5.58390
C	6.08797	5.19686	5.87874
C	7.13461	5.99811	6.50854
C	8.32613	5.32231	6.86666
C	9.36864	6.01619	7.46221
H	10.28855	5.50129	7.73482
C	9.23412	7.38614	7.71139
H	10.05283	7.93025	8.18000
C	8.06236	8.05885	7.36470
H	7.96669	9.12448	7.56273
C	7.01361	7.37042	6.76295
H	6.10685	7.90958	6.49471
C	8.62439	0.64194	4.13610
C	8.41422	1.73533	3.08153
H	7.38014	1.77642	2.72173
H	9.05942	1.52188	2.21994
H	8.69339	2.72870	3.45677
C	10.09930	0.61792	4.55299
H	10.42323	1.56307	5.00495
H	10.71006	0.45002	3.65671
H	10.32503	-0.18738	5.25734
C	8.21259	-0.71890	3.57672
H	8.37845	-1.53048	4.29276
H	8.83087	-0.93607	2.69574
H	7.16888	-0.74205	3.24550
C	7.35307	-0.05735	7.00407
C	6.68055	0.73681	8.13101
H	7.32292	1.53722	8.51583
H	6.47259	0.05662	8.96690
H	5.72376	1.17154	7.81554
C	6.46064	-1.24043	6.62362
H	5.42771	-0.93346	6.42980
H	6.43333	-1.93417	7.47398
H	6.83346	-1.80187	5.76142
C	8.72872	-0.55198	7.45821
H	9.18006	-1.23636	6.73216
H	8.60685	-1.10874	8.39609
H	9.42911	0.26785	7.65659

56

ts_2-2^{NOO}_singlet_UKS2

Au	8.59959	4.08385	6.43716
P	7.10455	2.46947	5.53204
N	7.14737	5.42934	5.87864
N	10.00076	2.89697	6.98583
O	9.08338	3.56053	9.68311
O	9.97602	2.97444	9.15019
C	5.78519	3.63572	5.01671
C	4.60839	3.26073	4.38820
H	4.41846	2.21344	4.16650
C	3.63353	4.21071	4.02147
H	2.72202	3.87758	3.52965
C	3.83078	5.54785	4.28618
H	3.07856	6.28500	4.00926
C	5.01684	5.98109	4.92002
C	5.99729	5.01815	5.27967
C	5.29181	7.34089	5.22170
H	4.55469	8.09908	4.95989
C	6.46431	7.70277	5.83391
H	6.66942	8.74375	6.06553
C	7.41465	6.70721	6.16665
C	8.70418	6.93807	6.81434
C	9.50499	5.79757	7.06695
C	10.74734	5.93985	7.66678
H	11.36861	5.06569	7.85640
C	11.20128	7.21311	8.02681
H	12.17671	7.32335	8.49839
C	10.41603	8.34053	7.78598
H	10.77673	9.32724	8.06948
C	9.17046	8.20732	7.18031
H	8.57069	9.09670	6.99536
C	7.78850	1.64505	3.98762
C	7.99813	2.78233	2.98030
H	7.05348	3.22607	2.64724
H	8.50926	2.37909	2.09687
H	8.63393	3.58053	3.38588
C	9.14559	1.02168	4.33399
H	9.84084	1.74805	4.77086
H	9.59762	0.64053	3.40933
H	9.05934	0.18006	5.02691
C	6.85806	0.58218	3.40468
H	6.73793	-0.27502	4.07540
H	7.30083	0.20295	2.47421
H	5.86848	0.97602	3.14938
C	6.38563	1.36712	6.86780
C	6.07175	2.30838	8.03789
H	6.97046	2.78582	8.44623
H	5.61695	1.72310	8.84741
H	5.35568	3.09030	7.75588
C	5.10261	0.64856	6.44737
H	4.27914	1.34888	6.27267
H	4.79310	-0.01083	7.26868
H	5.23089	0.02125	5.56055
C	7.46121	0.35761	7.27754
H	7.61440	-0.41256	6.51400
H	7.13386	-0.15319	8.19206
H	8.42573	0.83550	7.49175

56

ts_2-2^{NOO}_singlet_UKS

Au	8.23181	3.31764	6.40108
P	7.59388	1.16823	5.61437
N	6.40074	3.87136	5.71652
N	9.98445	2.86090	7.05924
O	9.80692	3.23888	8.97060
O	9.73299	2.21983	9.60201
C	5.93078	1.62978	4.99952
C	5.04049	0.74530	4.39975
H	5.30602	-0.29985	4.26843
C	3.78878	1.18608	3.95182
H	3.10763	0.47857	3.48292
C	3.40743	2.52397	4.10043
H	2.43025	2.85206	3.75005
C	4.26262	3.45092	4.68962
C	5.55012	3.00207	5.14553
C	3.92514	4.84104	4.86876
H	2.95273	5.19292	4.53226
C	4.81691	5.69509	5.45253
H	4.55965	6.74251	5.58702
C	6.08133	5.23427	5.89258
C	7.10548	5.98873	6.49836
C	8.32692	5.28605	6.87491
C	9.36892	5.97661	7.46109
H	10.28399	5.45559	7.73702
C	9.25267	7.35264	7.70277
H	10.07472	7.89400	8.16775
C	8.06577	8.05104	7.34295
H	7.99657	9.11953	7.53849
C	7.01725	7.39576	6.75447
H	6.11881	7.94433	6.48156
C	8.62261	0.64521	4.12970
C	8.40795	1.74605	3.08387
H	7.37323	1.78590	2.72570
H	9.05268	1.54026	2.22006
H	8.68456	2.73727	3.46618
C	10.09696	0.61870	4.54628
H	10.41841	1.55643	5.01499
H	10.70772	0.46630	3.64717
H	10.32409	-0.19736	5.23764
C	8.20923	-0.71143	3.56177
H	8.37941	-1.52832	4.27068
H	8.82441	-0.92030	2.67658
H	7.16422	-0.73255	3.23467
C	7.35921	-0.07376	7.00264
C	6.67619	0.71320	8.12862
H	7.31677	1.50976	8.52427
H	6.46184	0.02643	8.95750
H	5.72176	1.14980	7.80858
C	6.48157	-1.26625	6.61815
H	5.44628	-0.97053	6.41968
H	6.45865	-1.96012	7.46853
H	6.86463	-1.82321	5.75762
C	8.73983	-0.55026	7.46137
H	9.20340	-1.22854	6.73734
H	8.62038	-1.10903	8.39846
H	9.42745	0.28012	7.66191

56

ts_2-2^{NOO}_singlet_BS

Au	8.71268	1.58971	6.54319
P	10.20652	-0.18700	5.98821

N	7.25423	0.20572	6.13396
N	10.00042	3.00464	6.87535
O	10.07513	3.24903	8.71489
O	9.38776	2.53494	9.40266
C	8.92773	-1.43218	5.55701
C	9.19998	-2.71216	5.10089
H	10.22897	-3.03790	4.97155
C	8.16778	-3.62283	4.79678
H	8.42226	-4.61870	4.43995
C	6.84932	-3.25783	4.95473
H	6.04797	-3.95905	4.72661
C	6.51956	-1.96330	5.41427
C	7.56618	-1.04810	5.70772
C	5.18518	-1.51580	5.59916
H	4.36459	-2.19927	5.38415
C	4.92438	-0.24379	6.03992
H	3.90219	0.09486	6.18019
C	5.99989	0.63704	6.30700
C	5.87726	2.02039	6.76027
C	7.08248	2.74005	6.94489
C	7.04712	4.06190	7.36125
H	7.97077	4.62322	7.49472
C	5.81528	4.67732	7.60805
H	5.78889	5.71512	7.93694
C	4.62376	3.97319	7.43533
H	3.67014	4.45928	7.63090
C	4.65047	2.64895	7.00929
H	3.71217	2.11372	6.87522
C	11.22710	0.16477	4.45010
C	10.20192	0.37824	3.32946
H	9.65110	-0.53706	3.08680
H	10.73134	0.70066	2.42398
H	9.47803	1.16513	3.57941
C	12.00642	1.46568	4.67566
H	11.35680	2.30257	4.95654
H	12.50865	1.73297	3.73709
H	12.78073	1.36856	5.44214
C	12.19140	-0.96236	4.08303
H	12.97252	-1.10308	4.83745
H	12.69573	-0.69678	3.14461
H	11.68675	-1.91903	3.91192
C	11.10521	-0.84400	7.49841
C	10.05182	-0.86772	8.61309
H	9.67648	0.13336	8.85615
H	10.51298	-1.27304	9.52311
H	9.20012	-1.51317	8.36397
C	11.66572	-2.25442	7.31118
H	10.87236	-2.99677	7.17455
H	12.20958	-2.53245	8.22346
H	12.37109	-2.32991	6.47852
C	12.22499	0.13793	7.85430
H	13.06265	0.07734	7.15158
H	12.61481	-0.11842	8.84759
H	11.87793	1.17753	7.89460

58

2^{NCCH2}_triplet

Au	2.19432	9.79000	5.48501
P	2.33240	7.52023	4.73413
N	3.60685	10.08731	3.84958

N	0.48405	9.66472	7.03632
C	4.02836	11.33661	3.71589
C	4.97895	11.62552	2.70047
H	5.33852	12.64108	2.56295
C	5.44031	10.61151	1.90036
H	6.17044	10.82151	1.11955
C	5.44395	8.18405	1.30651
H	6.17407	8.35776	0.51714
C	4.97288	6.91501	1.56098
H	5.32820	6.07108	0.97322
C	4.03038	6.69427	2.58713
H	3.69231	5.67634	2.76349
C	3.54053	7.73161	3.36367
C	4.03395	9.04557	3.10662
C	4.98935	9.27616	2.07798
C	3.45889	12.30654	4.66059
C	2.55103	11.82221	5.63790
C	2.00024	12.72916	6.53801
H	1.29198	12.38908	7.29274
C	2.33618	14.08547	6.49055
H	1.89437	14.77919	7.20492
C	3.23238	14.55455	5.53128
H	3.49285	15.61043	5.49276
C	3.79104	13.66741	4.61747
H	4.48483	14.04789	3.86986
C	0.73723	6.92715	3.93097
C	-0.26911	6.61453	5.04110
H	-1.25502	6.44491	4.58889
H	-0.36760	7.44578	5.74962
H	-0.00805	5.70637	5.59604
C	0.89609	5.72496	3.00091
H	1.52183	5.95854	2.13339
H	-0.09495	5.45095	2.61517
H	1.30218	4.84178	3.50314
C	0.24610	8.12867	3.11236
H	0.04036	9.00142	3.74428
H	-0.68786	7.85892	2.60224
H	0.96992	8.42266	2.34207
C	3.11167	6.34280	5.98166
C	3.05159	4.86683	5.59240
H	2.02386	4.49311	5.53719
H	3.56926	4.27704	6.36070
H	3.55152	4.65764	4.64076
C	2.39001	6.55921	7.31786
H	2.47409	7.59975	7.65224
H	2.85574	5.92170	8.08074
H	1.32803	6.29824	7.27326
C	4.56881	6.79492	6.13025
H	5.16198	6.58735	5.23349
H	5.02651	6.25433	6.96886
H	4.64434	7.86838	6.34990
C	0.12511	9.80716	8.14420
H	0.41902	10.27483	10.21550
C	-0.29334	9.97712	9.44835
H	-1.33749	9.81903	9.71068

58

2^{NCCH2}_singlet

Au	2.44650	9.78003	5.60477
P	2.39328	7.50797	4.88060
N	3.62970	10.06995	3.97830

N	1.37188	9.60569	7.26327
C	4.04723	11.32849	3.79147
C	4.86196	11.62684	2.67273
H	5.20399	12.64524	2.51421
C	5.21228	10.62758	1.80333
H	5.84289	10.84601	0.94238
C	5.09236	8.22857	1.14437
H	5.72557	8.42295	0.27991
C	4.62117	6.95992	1.39870
H	4.87956	6.13555	0.73747
C	3.79737	6.71966	2.51624
H	3.44028	5.70750	2.68593
C	3.43866	7.73586	3.38825
C	3.93599	9.04411	3.13557
C	4.76033	9.29758	2.00562
C	3.60175	12.28604	4.79705
C	2.77448	11.77818	5.82684
C	2.32522	12.62030	6.83221
H	1.70681	12.23229	7.63940
C	2.68185	13.97289	6.81386
H	2.32749	14.63302	7.60436
C	3.49103	14.48164	5.79729
H	3.76554	15.53458	5.79390
C	3.95446	13.64229	4.79002
H	4.59148	14.05020	4.00727
C	0.69783	6.94799	4.29872
C	-0.11795	6.52202	5.52228
H	-1.16508	6.39448	5.21915
H	-0.09201	7.26938	6.32284
H	0.22192	5.56424	5.92991
C	0.74381	5.81521	3.27345
H	1.20154	6.13266	2.33083
H	-0.28724	5.51487	3.04472
H	1.26454	4.92522	3.64183
C	0.06764	8.19040	3.65643
H	-0.10152	8.99245	4.38565
H	-0.90926	7.91760	3.23646
H	0.67790	8.58377	2.83337
C	3.32382	6.35775	6.04050
C	3.34506	4.90562	5.56607
H	2.34429	4.46203	5.53256
H	3.93465	4.31393	6.27880
H	3.81924	4.78618	4.58602
C	2.68943	6.45202	7.43404
H	2.59293	7.48624	7.78361
H	3.33366	5.91441	8.14169
H	1.70139	5.98577	7.47812
C	4.75087	6.91710	6.10178
H	5.27021	6.84633	5.13965
H	5.32437	6.33559	6.83474
H	4.76951	7.96353	6.43330
C	0.17208	9.80603	7.28437
H	-1.52447	10.97915	7.64729
C	-1.13046	9.99667	7.39835
H	-1.82439	9.16880	7.27342

58

8’_triplet

Au	2.19089	9.77619	5.46988
P	2.25210	7.49222	4.74319
N	3.53961	10.06532	3.95314

C	3.99523	11.31803	3.82515
C	4.95811	11.60907	2.82889
H	5.32710	12.62458	2.72094
C	5.41430	10.60824	2.01287
H	6.15526	10.82156	1.24346
C	5.38647	8.20566	1.36342
H	6.12641	8.39696	0.58766
C	4.89979	6.93487	1.57357
H	5.25041	6.10429	0.96460
C	3.94664	6.69972	2.58386
H	3.59250	5.68311	2.72993
C	3.46505	7.72363	3.38460
C	3.97054	9.03418	3.17501
C	4.93719	9.28028	2.16155
C	3.42738	12.28611	4.75377
C	2.48669	11.78811	5.68373
C	1.86677	12.65201	6.57257
H	1.11646	12.27775	7.26574
C	2.19933	14.01048	6.56394
H	1.71495	14.68680	7.26694
C	3.14082	14.50647	5.66104
H	3.39251	15.56505	5.66126
C	3.75160	13.64990	4.75218
H	4.47391	14.05177	4.04397
C	0.65538	6.94686	3.90664
C	-0.38651	6.63364	4.98068
H	-1.34015	6.40264	4.48898
H	-0.56257	7.48880	5.64066
H	-0.11697	5.76161	5.58688
C	0.82271	5.75242	2.96715
H	1.46543	5.98607	2.11262
H	-0.16476	5.49684	2.56104
H	1.20553	4.85840	3.46819
C	0.20628	8.16623	3.09060
H	-0.00089	9.03635	3.72577
H	-0.72236	7.91840	2.56042
H	0.94908	8.45218	2.33559
C	3.04887	6.29875	5.96627
C	3.02081	4.83930	5.51440
H	2.00127	4.44657	5.44162
H	3.54553	4.23407	6.26540
H	3.53244	4.67317	4.56092
C	2.33022	6.43391	7.31138
H	2.37088	7.45621	7.70620
H	2.83141	5.78309	8.03946
H	1.28442	6.11548	7.26308
C	4.49559	6.78067	6.12915
H	5.09295	6.62686	5.22452
H	4.96533	6.21183	6.94167
H	4.54782	7.84233	6.40495
N	0.88281	9.80419	6.98595
C	0.08332	8.96430	7.73466
C	1.15179	9.72261	8.34386
H	-0.10197	7.89558	7.64372
H	1.23168	10.53515	9.06833

58

8’_singlet

Au	2.13953	9.76359	5.50997
P	2.25243	7.48920	4.74436
N	3.60110	10.04582	4.01002

C	4.07593	11.28751	3.92434
C	5.09375	11.57707	2.98022
H	5.49720	12.58286	2.91018
C	5.55206	10.58375	2.15464
H	6.32810	10.79560	1.42009
C	5.44313	8.20535	1.41050
H	6.20904	8.38899	0.65831
C	4.88766	6.95380	1.55744
H	5.20843	6.13297	0.91933
C	3.90487	6.72140	2.54132
H	3.50301	5.71654	2.63681
C	3.45900	7.73175	3.37810
C	4.02295	9.02894	3.22066
C	5.02408	9.26877	2.23970
C	3.44430	12.26381	4.81394
C	2.42084	11.78810	5.66872
C	1.69492	12.69348	6.42884
H	0.85045	12.34640	7.02303
C	2.01205	14.05483	6.39968
H	1.43750	14.75750	7.00191
C	3.05248	14.51947	5.59512
H	3.29836	15.57932	5.57605
C	3.75918	13.62951	4.79351
H	4.54420	14.01110	4.14309
C	0.63505	6.96416	3.93771
C	-0.37647	6.67215	5.04801
H	-1.35973	6.49296	4.59413
H	-0.48363	7.52543	5.72740
H	-0.11967	5.77626	5.62479
C	0.74636	5.77327	2.98635
H	1.37230	6.00025	2.11741
H	-0.25679	5.54407	2.60311
H	1.12207	4.86607	3.46811
C	0.18125	8.19605	3.14232
H	0.00076	9.06272	3.79002
H	-0.76206	7.96328	2.63133
H	0.91110	8.47928	2.37382
C	3.07109	6.23964	5.90011
C	3.00694	4.79302	5.41343
H	1.98048	4.41349	5.37476
H	3.55831	4.16128	6.12262
H	3.47233	4.64899	4.43337
C	2.39948	6.34552	7.27033
H	2.48099	7.35617	7.68636
H	2.90318	5.66242	7.96664
H	1.34343	6.05748	7.24254
C	4.52994	6.69256	6.03610
H	5.10238	6.53644	5.11622
H	5.00888	6.10829	6.83234
H	4.60754	7.75220	6.31431
N	0.61605	9.86688	6.86564
C	0.47691	8.87490	7.93848
C	0.98623	10.01078	8.26441
H	0.04627	7.89848	8.10530
H	1.38582	10.76499	8.93026

58

2^{CH2CN}_triplet

Au	2.29809	9.71768	5.53469
P	2.37588	7.48944	4.66075
N	3.64590	10.08675	3.86194

C	4.08186	11.33564	3.78078
C	4.99514	11.66845	2.74490
H	5.36574	12.68488	2.64985
C	5.40589	10.69559	1.86973
H	6.10675	10.93946	1.07215
C	5.34140	8.30778	1.13691
H	6.04077	8.51640	0.32840
C	4.85794	7.03365	1.33679
H	5.17221	6.22048	0.68571
C	3.95629	6.76738	2.38850
H	3.60776	5.74620	2.51914
C	3.51945	7.76413	3.24571
C	4.02409	9.08339	3.04332
C	4.93921	9.35930	1.98946
C	3.56506	12.25921	4.79920
C	2.68689	11.73437	5.78293
C	2.18373	12.59908	6.74993
H	1.49778	12.22920	7.51123
C	2.53872	13.95135	6.76270
H	2.13387	14.61142	7.52897
C	3.40663	14.45946	5.79740
H	3.68242	15.51211	5.80617
C	3.91724	13.61556	4.81705
H	4.58910	14.02648	4.06556
C	0.73828	6.94390	3.91134
C	-0.20697	6.56557	5.05442
H	-1.21834	6.43127	4.64908
H	-0.25780	7.34819	5.82102
H	0.07804	5.62146	5.53177
C	0.84366	5.79837	2.90494
H	1.41172	6.08772	2.01483
H	-0.16851	5.53833	2.56737
H	1.28547	4.89124	3.32822
C	0.20589	8.19083	3.19299
H	0.03169	9.02274	3.88638
H	-0.75275	7.94850	2.71590
H	0.88807	8.53447	2.40514
C	3.21185	6.24761	5.80403
C	3.13892	4.79675	5.33200
H	2.11067	4.42202	5.29594
H	3.68872	4.16549	6.04286
H	3.60111	4.64673	4.35062
C	2.55234	6.38129	7.18254
H	2.63721	7.40339	7.56962
H	3.06349	5.71186	7.88676
H	1.49351	6.10481	7.17439
C	4.67324	6.69817	5.91177
H	5.22461	6.54627	4.97789
H	5.17040	6.11118	6.69486
H	4.75622	7.75715	6.19091
C	0.06906	9.82734	9.62166
H	-0.94637	9.64859	9.96958
H	0.83539	10.14396	10.32685
C	0.38448	9.65683	8.28891
N	0.65908	9.52097	7.15593

58

2^{CH2CN}_singlet

Au	2.24787	9.78346	5.53675
P	2.30454	7.50033	4.82324
N	3.54066	10.07394	3.96176

C	3.94943	11.33490	3.79327
C	4.83883	11.63875	2.73364
H	5.17343	12.66104	2.58597
C	5.27313	10.63668	1.90600
H	5.96145	10.85855	1.09135
C	5.26658	8.22296	1.28756
H	5.95898	8.41702	0.46972
C	4.81478	6.94653	1.53829
H	5.14767	6.11494	0.92078
C	3.91632	6.70589	2.59680
H	3.58166	5.68609	2.76395
C	3.46032	7.72975	3.41256
C	3.93832	9.04550	3.16614
C	4.84028	9.29878	2.09681
C	3.42296	12.29455	4.75934
C	2.54219	11.79405	5.75209
C	2.04015	12.66361	6.71008
H	1.38287	12.31247	7.50261
C	2.39126	14.01746	6.68827
H	1.99244	14.68627	7.44945
C	3.24933	14.51223	5.70808
H	3.51996	15.56602	5.69755
C	3.76701	13.65253	4.74598
H	4.44351	14.04718	3.99039
C	0.67601	6.91421	4.07656
C	-0.26173	6.45132	5.19373
H	-1.25077	6.25335	4.76095
H	-0.39796	7.20803	5.97403
H	0.07780	5.52255	5.66368
C	0.84330	5.79512	3.04705
H	1.39285	6.13103	2.16222
H	-0.15503	5.48934	2.70763
H	1.33141	4.90400	3.45417
C	0.09141	8.14906	3.37977
H	-0.13774	8.95343	4.08921
H	-0.84603	7.87012	2.88151
H	0.76521	8.54591	2.60970
C	3.14200	6.33002	6.03621
C	3.14142	4.87415	5.57037
H	2.13086	4.46034	5.49019
H	3.67687	4.27177	6.31582
H	3.66013	4.73038	4.61713
C	2.43580	6.44222	7.39206
H	2.52547	7.44337	7.82539
H	2.92206	5.75132	8.09260
H	1.37806	6.16551	7.35107
C	4.57934	6.84352	6.18318
H	5.16484	6.71635	5.26638
H	5.07567	6.27478	6.97975
H	4.61160	7.90123	6.47609
C	0.94342	9.67742	7.12381
H	0.23173	10.50585	7.03932
H	0.38195	8.73935	7.11740
C	1.70005	9.77599	8.35076
N	2.34227	9.83536	9.31423

58

$2^{CHCHN'}$_triplet

Au	-0.21254	1.08931	0.87118
P	-0.29504	-1.16764	0.08491
N	0.94621	1.39227	-0.80814

N	-3.22095	1.37588	3.83870
C	1.37191	2.64684	-0.97935
C	2.17393	2.95862	-2.10438
H	2.52448	3.97536	-2.25359
C	2.50130	1.97048	-2.99612
H	3.11694	2.19912	-3.86523
C	2.34565	-0.41828	-3.69226
H	2.95854	-0.21745	-4.56978
C	1.86257	-1.68442	-3.44796
H	2.08836	-2.49967	-4.13215
C	1.07094	-1.93653	-2.30937
H	0.70671	-2.94753	-2.15026
C	0.75514	-0.93344	-1.40619
C	1.24625	0.37689	-1.66095
C	2.04661	0.63968	-2.80554
C	0.95448	3.59062	0.05519
C	0.15983	3.08047	1.11423
C	-0.24019	3.93204	2.13543
H	-0.83736	3.55958	2.96479
C	0.12764	5.28160	2.11029
H	-0.19272	5.93935	2.91696
C	0.90037	5.78718	1.06649
H	1.18323	6.83778	1.05320
C	1.31565	4.94390	0.04139
H	1.92281	5.34900	-0.76587
C	-1.99593	-1.68820	-0.52632
C	-2.99665	-1.55529	0.62571
H	-4.00270	-1.75610	0.23571
H	-3.00210	-0.54912	1.05575
H	-2.81402	-2.26785	1.43495
C	-2.03255	-3.10968	-1.08614
H	-1.35650	-3.24800	-1.93589
H	-3.04783	-3.31382	-1.45084
H	-1.80785	-3.86348	-0.32436
C	-2.35611	-0.67687	-1.62200
H	-2.31465	0.35801	-1.25726
H	-3.38638	-0.86472	-1.94982
H	-1.70604	-0.76203	-2.49969
C	0.61860	-2.36925	1.20708
C	1.10218	-3.63406	0.49599
H	0.29039	-4.20777	0.03816
H	1.57744	-4.28580	1.24089
H	1.85821	-3.41384	-0.26422
C	-0.29106	-2.74214	2.38024
H	-0.74087	-1.86744	2.86290
H	0.30900	-3.26321	3.13722
H	-1.09398	-3.42348	2.07942
C	1.83793	-1.58691	1.71166
H	2.48102	-1.25220	0.88784
H	2.44159	-2.24304	2.35188
H	1.55510	-0.71236	2.31001
C	-2.53925	1.52814	2.74936
C	-1.28815	0.91988	2.53706
H	-2.98155	2.16471	1.96449
H	-0.87608	0.32553	3.35580

58

$2^{CHCHN'}$_singlet

Au	-0.21027	1.08574	0.87162
P	-0.29149	-1.17035	0.08487
N	0.94687	1.39095	-0.80747

N	-3.23350	1.40498	3.81286
C	1.37030	2.64634	-0.97894
C	2.17125	2.95928	-2.10435
H	2.52020	3.97656	-2.25372
C	2.49968	1.97162	-2.99628
H	3.11467	2.20122	-3.86560
C	2.34591	-0.41702	-3.69341
H	2.95826	-0.21525	-4.57109
C	1.86346	-1.68359	-3.44996
H	2.08926	-2.49814	-4.13496
C	1.07249	-1.93696	-2.31118
H	0.70842	-2.94817	-2.15280
C	0.75715	-0.93477	-1.40687
C	1.24736	0.37607	-1.66089
C	2.04667	0.64021	-2.80585
C	0.95182	3.58926	0.05601
C	0.15901	3.07718	1.11543
C	-0.24067	3.92713	2.13825
H	-0.83510	3.55367	2.96935
C	0.12441	5.27739	2.11342
H	-0.19608	5.93401	2.92094
C	0.89470	5.78504	1.06873
H	1.17541	6.83622	1.05571
C	1.31057	4.94319	0.04272
H	1.91633	5.34988	-0.76480
C	-1.99439	-1.68719	-0.52316
C	-2.99370	-1.54797	0.62946
H	-4.00036	-1.74999	0.24171
H	-2.99860	-0.53942	1.05434
H	-2.81057	-2.25634	1.44225
C	-2.03481	-3.11044	-1.07847
H	-1.35836	-3.25310	-1.92723
H	-3.05034	-3.31294	-1.44335
H	-1.81253	-3.86256	-0.31433
C	-2.35308	-0.67871	-1.62197
H	-2.30712	0.35749	-1.26150
H	-3.38467	-0.86403	-1.94704
H	-1.70504	-0.76973	-2.50056
C	0.62156	-2.37363	1.20567
C	1.10440	-3.63747	0.49232
H	0.29219	-4.21006	0.03379
H	1.57953	-4.29062	1.23607
H	1.86037	-3.41634	-0.26769
C	-0.28828	-2.74783	2.37820
H	-0.73611	-1.87344	2.86320
H	0.31127	-3.27183	3.13355
H	-1.09269	-3.42691	2.07623
C	1.84112	-1.59238	1.71129
H	2.48423	-1.25664	0.88790
H	2.44471	-2.24959	2.35047
H	1.55841	-0.71864	2.31090
C	-2.54607	1.55123	2.74824
C	-1.28757	0.91348	2.53389
H	-2.96600	2.20369	1.96107
H	-0.89260	0.31087	3.35421

58

8_triplet

Au	2.33717	9.78462	5.56416
P	2.31512	7.50706	4.81055
N	3.58677	10.07683	3.94894

C	4.03163	11.32831	3.80173
C	4.91417	11.62554	2.73510
H	5.27704	12.64024	2.60201
C	5.30678	10.62605	1.88331
H	5.98938	10.84280	1.06252
C	5.23173	8.22387	1.22803
H	5.91574	8.41401	0.40224
C	4.75671	6.95453	1.47024
H	5.06190	6.12393	0.83730
C	3.87074	6.71904	2.54062
H	3.51958	5.70393	2.70146
C	3.44570	7.74030	3.37607
C	3.94764	9.04847	3.13646
C	4.84138	9.29725	2.05841
C	3.54480	12.28453	4.79217
C	2.67284	11.78595	5.79191
C	2.16597	12.65130	6.75114
H	1.47980	12.29157	7.51584
C	2.52485	14.00336	6.73353
H	2.12272	14.67466	7.49095
C	3.38945	14.49596	5.75704
H	3.66519	15.54849	5.75074
C	3.89744	13.64037	4.78555
H	4.56677	14.03767	4.02481
C	0.66482	6.93671	4.09826
C	-0.22753	6.40126	5.21945
H	-1.23351	6.23947	4.81081
H	-0.31567	7.09981	6.06083
H	0.12408	5.43657	5.60091
C	0.81547	5.87765	3.00452
H	1.33237	6.26294	2.11990
H	-0.19039	5.57584	2.68469
H	1.32444	4.97274	3.35263
C	0.04606	8.20105	3.48998
H	-0.17897	8.95372	4.25488
H	-0.90002	7.93708	3.00005
H	0.69337	8.65511	2.72839
C	3.17561	6.31527	5.98379
C	3.12807	4.86112	5.51401
H	2.10707	4.47051	5.45934
H	3.67007	4.24348	6.24219
H	3.61718	4.70972	4.54614
C	2.51395	6.44392	7.36040
H	2.62490	7.45275	7.77030
H	3.01045	5.74997	8.05060
H	1.44756	6.20398	7.35344
C	4.62950	6.79159	6.08064
H	5.18217	6.64309	5.14677
H	5.13726	6.21625	6.86530
H	4.69978	7.85185	6.35858
N	0.11425	8.77830	7.37639
C	-0.18080	10.16978	7.08244
H	-0.66277	10.52879	6.17092
C	1.16436	9.69559	7.26199
H	1.84942	9.79972	8.11643

58

8_singlet

Au	2.33513	9.78766	5.55570
P	2.32142	7.50899	4.80178
N	3.58245	10.06911	3.90917

C	4.01384	11.32028	3.74565
C	4.88514	11.61838	2.66919
H	5.23697	12.63488	2.52087
C	5.28511	10.61308	1.82698
H	5.96062	10.82796	0.99973
C	5.23785	8.19887	1.20699
H	5.91752	8.38225	0.37601
C	4.77691	6.92904	1.47206
H	5.08957	6.09132	0.85210
C	3.89440	6.70030	2.54764
H	3.55344	5.68406	2.72166
C	3.46044	7.72931	3.36844
C	3.95071	9.03919	3.10670
C	4.83770	9.28079	2.02162
C	3.53408	12.28065	4.74162
C	2.68950	11.78508	5.76793
C	2.22771	12.65887	6.74327
H	1.58319	12.29956	7.54341
C	2.58692	14.00992	6.71301
H	2.21821	14.68329	7.48554
C	3.41334	14.49824	5.70266
H	3.69077	15.55021	5.68182
C	3.88729	13.63583	4.71964
H	4.53485	14.02845	3.93799
C	0.68393	6.91606	4.07498
C	-0.21089	6.36739	5.18799
H	-1.20984	6.18968	4.76832
H	-0.32016	7.06539	6.02533
H	0.15113	5.40701	5.57077
C	0.85221	5.86069	2.98084
H	1.37273	6.25135	2.10079
H	-0.14835	5.54967	2.65296
H	1.36724	4.96002	3.33161
C	0.05317	8.17421	3.46579
H	-0.18369	8.92197	4.23179
H	-0.88646	7.90000	2.96886
H	0.70058	8.63746	2.70987
C	3.17829	6.32725	5.98881
C	3.17902	4.87448	5.51312
H	2.16906	4.46393	5.41465
H	3.70336	4.26353	6.25971
H	3.71064	4.73908	4.56572
C	2.48111	6.43217	7.34979
H	2.51980	7.45047	7.74907
H	3.00218	5.77583	8.05866
H	1.43286	6.12335	7.32031
C	4.61715	6.83967	6.12319
H	5.19036	6.72315	5.19708
H	5.12577	6.26315	6.90651
H	4.65226	7.89593	6.42163
N	-0.02477	8.85003	7.31504
C	-0.21592	10.08729	7.21842
H	-1.04465	10.79158	7.15614
C	1.18250	9.73911	7.21857
H	1.77584	9.85979	8.13233

58

2^{CHCHN}_triplet

Au	-0.79780	-0.82003	1.73162
P	0.06850	-1.93920	3.65651
N	-0.43760	-2.62423	0.80217

N	-3.45894	0.68308	2.79227
C	-0.77725	-2.67020	-0.48908
C	-0.55424	-3.86114	-1.22266
H	-0.83193	-3.91043	-2.27114
C	0.01746	-4.94061	-0.60143
H	0.19555	-5.86110	-1.15621
C	0.99112	-5.95369	1.45691
H	1.18217	-6.88460	0.92507
C	1.32918	-5.82076	2.78489
H	1.79252	-6.64776	3.31890
C	1.07643	-4.61308	3.46630
H	1.35485	-4.54829	4.51419
C	0.48817	-3.52938	2.83315
C	0.13956	-3.66409	1.46090
C	0.38995	-4.87838	0.76641
C	-1.34938	-1.43786	-1.02491
C	-1.45959	-0.33737	-0.13714
C	-1.98693	0.86010	-0.60061
H	-2.08400	1.71403	0.06542
C	-2.40559	0.97594	-1.92993
H	-2.81891	1.91988	-2.28220
C	-2.30064	-0.10473	-2.80405
H	-2.63003	-0.00825	-3.83657
C	-1.77416	-1.31035	-2.35389
H	-1.69870	-2.14793	-3.04477
C	-1.22687	-2.35339	4.95873
C	-1.87921	-1.06149	5.45933
H	-2.66108	-1.33381	6.17995
H	-2.36458	-0.50590	4.65235
H	-1.17944	-0.39885	5.97602
C	-0.65776	-3.13794	6.14007
H	-0.20632	-4.09021	5.84403
H	-1.48204	-3.37787	6.82460
H	0.07413	-2.55769	6.71216
C	-2.28579	-3.18534	4.22331
H	-2.70820	-2.64448	3.36636
H	-3.11354	-3.38654	4.91505
H	-1.89792	-4.15020	3.87841
C	1.68142	-1.18247	4.25636
C	2.56979	-2.14436	5.04657
H	2.07649	-2.56246	5.92958
H	3.44784	-1.58802	5.39987
H	2.94156	-2.96386	4.42327
C	1.35113	0.04924	5.10293
H	0.63138	0.71389	4.61038
H	2.27285	0.62149	5.26898
H	0.95587	-0.22266	6.08734
C	2.42131	-0.76057	2.98054
H	2.60033	-1.60947	2.30837
H	3.40079	-0.34887	3.25661
H	1.88273	0.01590	2.42416
C	-2.40904	1.44068	2.83719
C	-1.12670	1.00372	2.46806
H	-2.54112	2.47621	3.19344
H	-0.29228	1.70556	2.51519

58

2^{CHCHN}_singlet

Au	-0.79642	-0.82057	1.73319
P	0.06979	-1.94108	3.65739
N	-0.43579	-2.62351	0.80252

N	-3.47219	0.67938	2.79470
C	-0.77459	-2.66829	-0.48906
C	-0.55218	-3.85888	-1.22332
H	-0.82917	-3.90710	-2.27203
C	0.01772	-4.93947	-0.60235
H	0.19509	-5.85981	-1.15761
C	0.98785	-5.95541	1.45624
H	1.17803	-6.88622	0.92392
C	1.32459	-5.82406	2.78472
H	1.78598	-6.65222	3.31862
C	1.07303	-4.61648	3.46674
H	1.35032	-4.55285	4.51503
C	0.48719	-3.53144	2.83367
C	0.13964	-3.66459	1.46101
C	0.38902	-4.87869	0.76587
C	-1.34553	-1.43515	-1.02432
C	-1.45635	-0.33583	-0.13538
C	-1.98307	0.86242	-0.59763
H	-2.08091	1.71569	0.06922
C	-2.40034	0.98022	-1.92714
H	-2.81337	1.92461	-2.27851
C	-2.29454	-0.09933	-2.80260
H	-2.62285	-0.00134	-3.83532
C	-1.76884	-1.30571	-2.35359
H	-1.69283	-2.14235	-3.04555
C	-1.22508	-2.35177	4.96095
C	-1.87889	-1.05861	5.45639
H	-2.65757	-1.32906	6.18118
H	-2.36948	-0.50911	4.64817
H	-1.17915	-0.39147	5.96741
C	-0.65422	-3.13097	6.14505
H	-0.19993	-4.08295	5.85240
H	-1.47811	-3.37085	6.83006
H	0.07588	-2.54684	6.71548
C	-2.28330	-3.18769	4.22909
H	-2.70614	-2.65070	3.36990
H	-3.11097	-3.38612	4.92172
H	-1.89494	-4.15387	3.88849
C	1.68391	-1.18504	4.25445
C	2.57127	-2.14721	5.04549
H	2.07814	-2.56250	5.92991
H	3.45092	-1.59201	5.39658
H	2.94053	-2.96876	4.42337
C	1.35544	0.04845	5.09906
H	0.63731	0.71343	4.60433
H	2.27810	0.61926	5.26484
H	0.95882	-0.22108	6.08357
C	2.42341	-0.76594	2.97748
H	2.60105	-1.61603	2.30645
H	3.40353	-0.35494	3.25229
H	1.88522	0.01041	2.42051
C	-2.43636	1.42724	2.83572
C	-1.12984	0.99790	2.47236
H	-2.57383	2.46790	3.18214
H	-0.30949	1.71677	2.50603

58

2$^{NCHCH'}$_triplet

Au	2.09302	9.78154	5.41973
P	2.23039	7.50694	4.72235
N	3.55333	10.07981	4.01814

N	0.54930	9.55013	6.60128
C	4.00537	11.33419	3.92481
C	5.00765	11.63985	2.97223
H	5.38151	12.65606	2.89066
C	5.48966	10.65049	2.15535
H	6.25744	10.87586	1.41619
C	5.45713	8.26215	1.44246
H	6.22211	8.46244	0.69369
C	4.94357	6.99413	1.60046
H	5.29883	6.17681	0.97633
C	3.95682	6.74316	2.57537
H	3.58154	5.72851	2.68202
C	3.46912	7.75143	3.39102
C	3.99905	9.06211	3.23144
C	4.99918	9.32243	2.25585
C	3.37898	12.28784	4.83684
C	2.36445	11.78332	5.68867
C	1.70393	12.64824	6.54941
H	0.90260	12.28592	7.19049
C	2.05461	14.00207	6.58798
H	1.53076	14.67418	7.26623
C	3.06245	14.49812	5.76164
H	3.32951	15.55234	5.79721
C	3.72251	13.64515	4.88358
H	4.50214	14.04581	4.23820
C	0.63044	6.93150	3.92855
C	-0.36244	6.60862	5.04891
H	-1.35004	6.44103	4.59978
H	-0.45609	7.44076	5.75854
H	-0.09582	5.69423	5.59070
C	0.79766	5.74116	2.98467
H	1.40770	5.99181	2.11031
H	-0.19549	5.45968	2.61055
H	1.21935	4.85733	3.47291
C	0.12971	8.14311	3.13061
H	-0.11381	8.98950	3.78348
H	-0.78955	7.86178	2.60078
H	0.85570	8.47022	2.37509
C	3.01398	6.35644	5.98854
C	2.97456	4.88571	5.57590
H	1.95234	4.49806	5.51849
H	3.50282	4.29437	6.33550
H	3.47666	4.69837	4.62046
C	2.28228	6.55072	7.32120
H	2.37446	7.57914	7.68463
H	2.73766	5.88953	8.06973
H	1.21764	6.30614	7.26665
C	4.46479	6.82553	6.14669
H	5.06855	6.62477	5.25545
H	4.92009	6.28752	6.98800
H	4.52828	7.89846	6.37237
C	0.64562	9.75610	7.89152
H	1.58221	10.09580	8.37362
C	-0.42916	9.56379	8.74761
H	-0.58474	9.66324	9.81764

58

2^{NCHCH}_triplet

Au	2.09505	9.78501	5.44209
P	2.22957	7.50863	4.74608
N	3.54930	10.08111	4.02982

N	0.55500	9.60045	6.65034
C	3.99008	11.33828	3.92213
C	4.99662	11.64057	2.97259
H	5.36336	12.65860	2.88225
C	5.49177	10.64580	2.17071
H	6.26275	10.86825	1.43402
C	5.47531	8.25112	1.47968
H	6.24282	8.44862	0.73277
C	4.96421	6.98294	1.64388
H	5.32336	6.16243	1.02621
C	3.97355	6.73627	2.61576
H	3.59707	5.72238	2.72325
C	3.48178	7.74783	3.42509
C	4.00676	9.05895	3.25661
C	5.00915	9.31573	2.28240
C	3.34596	12.29976	4.81222
C	2.33565	11.79744	5.66915
C	1.64837	12.67129	6.49976
H	0.85187	12.30680	7.14433
C	1.97046	14.03220	6.50416
H	1.42595	14.71131	7.15878
C	2.97762	14.52673	5.67560
H	3.22310	15.58675	5.68575
C	3.66291	13.66478	4.82685
H	4.43885	14.06415	4.17631
C	0.63406	6.96994	3.91560
C	-0.39080	6.66722	5.01188
H	-1.37382	6.53127	4.54250
H	-0.47562	7.49529	5.72723
H	-0.16084	5.74185	5.55153
C	0.79451	5.77819	2.97244
H	1.43092	6.01618	2.11351
H	-0.19588	5.52112	2.57423
H	1.18426	4.88365	3.46734
C	0.17807	8.19257	3.10758
H	-0.05954	9.04615	3.75335
H	-0.73591	7.93229	2.55814
H	0.92763	8.50219	2.36813
C	2.98379	6.32416	5.99973
C	2.98357	4.86710	5.53888
H	1.97058	4.46684	5.42833
H	3.48876	4.26051	6.30211
H	3.52857	4.71526	4.60129
C	2.20899	6.45477	7.31451
H	2.25759	7.47212	7.71476
H	2.66606	5.78454	8.05410
H	1.15624	6.17275	7.22267
C	4.41995	6.81295	6.22193
H	5.05136	6.67081	5.33849
H	4.86340	6.23934	7.04587
H	4.45241	7.87272	6.50770
C	0.73640	9.71679	7.94198
H	-0.17083	9.61393	8.56375
C	1.92975	9.94901	8.60806
H	2.24348	10.08267	9.63906

58

$2^{NCHCH'}$_singlet

Au	2.09153	9.77958	5.41950
P	2.22992	7.50618	4.72086
N	3.55205	10.07953	4.01811

N	0.55003	9.54977	6.59875
C	4.00209	11.33459	3.92427
C	5.00518	11.64098	2.97275
H	5.37769	12.65767	2.89094
C	5.48988	10.65161	2.15745
H	6.25844	10.87750	1.41925
C	5.46173	8.26247	1.44647
H	6.22745	8.46312	0.69855
C	4.94967	6.99385	1.60485
H	5.30687	6.17649	0.98189
C	3.96174	6.74235	2.57846
H	3.58747	5.72732	2.68526
C	3.47162	7.75075	3.39247
C	4.00021	9.06194	3.23273
C	5.00120	9.32287	2.25828
C	3.37284	12.28790	4.83472
C	2.35960	11.78206	5.68719
C	1.69600	12.64596	6.54678
H	0.89656	12.28160	7.18921
C	2.04255	14.00089	6.58284
H	1.51665	14.67254	7.25997
C	3.04892	14.49867	5.75569
H	3.31248	15.55383	5.78941
C	3.71203	13.64642	4.87926
H	4.49033	14.04851	4.23318
C	0.63070	6.93437	3.92336
C	-0.36687	6.61715	5.04120
H	-1.35359	6.45210	4.58923
H	-0.46019	7.45078	5.74927
H	-0.10514	5.70287	5.58555
C	0.79813	5.74119	2.98304
H	1.41290	5.98771	2.11082
H	-0.19424	5.46196	2.60522
H	1.21484	4.85731	3.47541
C	0.13643	8.14539	3.12058
H	-0.10713	8.99426	3.77025
H	-0.78175	7.86532	2.58825
H	0.86613	8.46837	2.36690
C	3.00798	6.35551	5.98993
C	2.96680	4.88464	5.57754
H	1.94401	4.49863	5.51933
H	3.49338	4.29275	6.33786
H	3.46950	4.69618	4.62264
C	2.27257	6.55173	7.32021
H	2.36854	7.57894	7.68606
H	2.72192	5.88713	8.06939
H	1.20680	6.31307	7.26207
C	4.45918	6.82192	6.15183
H	5.06520	6.61917	5.26258
H	4.91086	6.28369	6.99496
H	4.52408	7.89489	6.37688
C	0.66634	9.75164	7.90816
H	1.61339	10.08843	8.37218
C	-0.37065	9.56283	8.77404
H	-0.53052	9.64862	9.84473

58

2^{NCHCH}_singlet

Au	2.09039	9.78252	5.43794
P	2.22590	7.50644	4.74251
N	3.54844	10.07827	4.03004

N	0.55266	9.59777	6.64419
C	3.99122	11.33498	3.92463
C	4.99909	11.63735	2.97667
H	5.36745	12.65498	2.88837
C	5.49321	10.64324	2.17331
H	6.26504	10.86580	1.43754
C	5.47302	8.25013	1.47743
H	6.24115	8.44790	0.73124
C	4.95960	6.98257	1.63878
H	5.31731	6.16268	1.01946
C	3.96839	6.73550	2.61003
H	3.59028	5.72199	2.71499
C	3.47804	7.74611	3.42141
C	4.00497	9.05676	3.25538
C	5.00831	9.31384	2.28221
C	3.34753	12.29603	4.81552
C	2.33374	11.79411	5.66846
C	1.64739	12.66730	6.50081
H	0.84876	12.30320	7.14293
C	1.97368	14.02727	6.51012
H	1.42959	14.70606	7.16542
C	2.98408	14.52132	5.68529
H	3.23276	15.58054	5.69935
C	3.66865	13.65994	4.83523
H	4.44762	14.05893	4.18808
C	0.63021	6.96651	3.91339
C	-0.39494	6.66681	5.01017
H	-1.37834	6.53258	4.54110
H	-0.47821	7.49554	5.72506
H	-0.16626	5.74146	5.55028
C	0.79044	5.77171	2.97395
H	1.42546	6.00726	2.11337
H	-0.20021	5.51230	2.57795
H	1.18170	4.87943	3.47179
C	0.17489	8.18720	3.10206
H	-0.06279	9.04235	3.74573
H	-0.73893	7.92565	2.55297
H	0.92484	8.49483	2.36222
C	2.98346	6.32693	5.99857
C	3.00007	4.87130	5.53360
H	1.99156	4.46147	5.41707
H	3.50719	4.26782	6.29798
H	3.55167	4.72754	4.59863
C	2.20136	6.44672	7.30990
H	2.23631	7.46357	7.71334
H	2.66317	5.78020	8.04994
H	1.15264	6.15160	7.21304
C	4.41327	6.83047	6.22885
H	5.04962	6.70214	5.34675
H	4.86030	6.25587	7.05017
H	4.43157	7.88810	6.52354
C	0.75218	9.71761	7.95007
H	-0.15150	9.61317	8.57780
C	1.92874	9.93903	8.60778
H	2.24767	10.16769	9.62089

58

ts_2$^{CHCHN'}$-8_singlet

Au	-0.26099	1.04086	0.87797
P	-0.31010	-1.20734	0.05742
N	0.90362	1.37365	-0.80251

N	-2.54061	2.18902	3.75008
C	1.32800	2.63062	-0.95214
C	2.13425	2.96116	-2.06990
H	2.48017	3.98151	-2.20417
C	2.47219	1.98683	-2.97202
H	3.09088	2.23004	-3.83499
C	2.34694	-0.39713	-3.69039
H	2.96409	-0.18149	-4.56140
C	1.88000	-1.67204	-3.45920
H	2.12297	-2.47900	-4.14730
C	1.08429	-1.94311	-2.32825
H	0.73449	-2.96059	-2.17727
C	0.74696	-0.94989	-1.42189
C	1.22122	0.36946	-1.66211
C	2.02811	0.64985	-2.79792
C	0.90972	3.55658	0.09619
C	0.11324	3.03459	1.14931
C	-0.28407	3.87236	2.18056
H	-0.90028	3.50274	2.99930
C	0.09312	5.22020	2.17515
H	-0.22679	5.86610	2.99148
C	0.86763	5.73864	1.13945
H	1.15475	6.78822	1.14095
C	1.27643	4.90869	0.10143
H	1.88330	5.32247	-0.70154
C	-2.00012	-1.74061	-0.57229
C	-3.01663	-1.59199	0.56318
H	-4.01553	-1.81834	0.16895
H	-3.03729	-0.57165	0.95726
H	-2.83093	-2.27449	1.39724
C	-2.02591	-3.17101	-1.10945
H	-1.32842	-3.32349	-1.93926
H	-3.03171	-3.38059	-1.49671
H	-1.82009	-3.91238	-0.33043
C	-2.34914	-0.74776	-1.68816
H	-2.30223	0.29382	-1.34330
H	-3.37872	-0.93537	-2.01816
H	-1.69467	-0.85342	-2.55997
C	0.60106	-2.40339	1.18565
C	1.08907	-3.67093	0.48263
H	0.27992	-4.24998	0.02705
H	1.56590	-4.31679	1.23167
H	1.84528	-3.45238	-0.27812
C	-0.31534	-2.76561	2.35706
H	-0.76434	-1.88260	2.82761
H	0.27926	-3.28180	3.12174
H	-1.11805	-3.44798	2.05710
C	1.81751	-1.61801	1.69277
H	2.46361	-1.28391	0.87111
H	2.41907	-2.27205	2.33712
H	1.53054	-0.74301	2.28835
C	-2.61659	1.40034	2.77797
C	-1.32095	0.76238	2.54542
H	-3.49888	1.28693	2.12786
H	-0.73922	0.53305	3.44183

58

ts_8-8'_singlet

Au	-0.42312	1.01856	0.90095
P	-0.32817	-1.19227	0.01914
N	0.98784	1.37501	-0.78572

C	1.41854	2.61897	-0.85871
C	2.38308	2.94646	-1.85047
H	2.76188	3.96042	-1.94279
C	2.83885	1.96070	-2.69202
H	3.57882	2.19712	-3.45593
C	2.80892	-0.44596	-3.39220
H	3.54535	-0.25922	-4.17277
C	2.30971	-1.71330	-3.18635
H	2.64930	-2.54072	-3.80621
C	1.35856	-1.96000	-2.17161
H	1.00232	-2.97853	-2.04296
C	0.88626	-0.94675	-1.35448
C	1.40700	0.36646	-1.56617
C	2.37073	0.62359	-2.58001
C	0.83027	3.54962	0.12658
C	-0.09069	3.04196	1.09103
C	-0.65957	3.94882	1.97702
H	-1.38826	3.61323	2.71852
C	-0.33932	5.31022	1.95634
H	-0.80190	5.99049	2.67029
C	0.57117	5.79249	1.02125
H	0.82691	6.84990	0.99463
C	1.14671	4.91299	0.10844
H	1.84301	5.30618	-0.63000
C	-1.93267	-1.72119	-0.81371
C	-2.92800	-2.14417	0.26930
H	-3.91775	-2.26799	-0.18956
H	-3.02012	-1.39268	1.06156
H	-2.66224	-3.10608	0.72189
C	-1.77433	-2.83158	-1.85223
H	-1.16264	-2.51186	-2.70182
H	-2.76839	-3.08001	-2.24724
H	-1.35318	-3.75272	-1.43801
C	-2.43766	-0.45441	-1.51632
H	-2.64052	0.35498	-0.80423
H	-3.37399	-0.68309	-2.04202
H	-1.72197	-0.08585	-2.26212
C	0.43447	-2.46934	1.17877
C	0.34335	-3.91495	0.69273
H	-0.69177	-4.25981	0.60143
H	0.83800	-4.56568	1.42619
H	0.85114	-4.07343	-0.26399
C	-0.27775	-2.33566	2.52987
H	-0.14986	-1.32677	2.93832
H	0.16687	-3.04298	3.24200
H	-1.34918	-2.55028	2.47300
C	1.90133	-2.06027	1.35426
H	2.48953	-2.22497	0.44562
H	2.34742	-2.66196	2.15684
H	2.00105	-1.00349	1.63706
N	-2.44051	0.58240	2.57106
C	-1.53232	1.10113	3.37175
C	-2.76209	1.69588	3.31187
H	-0.56155	0.87684	3.80512
H	-3.40886	2.56356	3.24681

58

ts_2^{CHCHN}-2^{CH2CN}_singlet

Au	-0.33432	1.07320	0.83147
P	-0.31905	-1.19451	0.06734
N	0.91255	1.37925	-0.77033

N	-3.26339	2.60395	3.23430
C	1.34047	2.63608	-0.92768
C	2.16905	2.95795	-2.03102
H	2.51213	3.97894	-2.16672
C	2.53232	1.97788	-2.91527
H	3.17340	2.21328	-3.76383
C	2.43606	-0.41107	-3.60921
H	3.08264	-0.20057	-4.45987
C	1.97286	-1.68723	-3.37774
H	2.25157	-2.50221	-4.04242
C	1.13318	-1.94687	-2.27730
H	0.78897	-2.96545	-2.12227
C	0.74814	-0.94171	-1.40329
C	1.23953	0.37198	-1.62768
C	2.08221	0.64347	-2.73971
C	0.90555	3.57122	0.10066
C	0.06151	3.06266	1.12275
C	-0.30851	3.90647	2.16122
H	-0.93419	3.55965	2.97825
C	0.11215	5.23990	2.17828
H	-0.19599	5.88461	2.99997
C	0.91702	5.74592	1.15963
H	1.23834	6.78529	1.17725
C	1.31851	4.91046	0.12509
H	1.96246	5.30645	-0.65757
C	-1.98445	-1.79987	-0.56933
C	-2.84136	-2.23581	0.62151
H	-3.85646	-2.45072	0.26329
H	-2.92185	-1.45097	1.38161
H	-2.46487	-3.15031	1.09224
C	-1.88214	-2.93897	-1.58490
H	-1.40297	-2.61790	-2.51509
H	-2.90038	-3.25745	-1.84428
H	-1.35942	-3.81882	-1.19725
C	-2.61703	-0.58024	-1.25010
H	-2.79548	0.23561	-0.54022
H	-3.58478	-0.87184	-1.67833
H	-1.99667	-0.19920	-2.07130
C	0.62278	-2.36256	1.20906
C	0.58785	-3.81866	0.74713
H	-0.42076	-4.24413	0.77660
H	1.20848	-4.41540	1.42857
H	1.00016	-3.95643	-0.25780
C	0.04269	-2.24858	2.62299
H	0.14519	-1.23380	3.02291
H	0.60597	-2.91897	3.28481
H	-1.00887	-2.54739	2.68187
C	2.06660	-1.84703	1.23083
H	2.57375	-1.97950	0.26938
H	2.62949	-2.40941	1.98669
H	2.12050	-0.78598	1.50816
C	-2.43146	1.79862	2.88060
C	-1.62756	0.81770	2.33507
H	-3.28530	1.49384	2.13830
H	-1.75681	-0.16485	2.78266

58

ts_2^{CHCHN}-2$^{CHCHN'}$_triplet

Au	-0.25181	1.06372	0.87593
P	-0.31306	-1.19180	0.06342
N	0.91303	1.38308	-0.79764

N	-2.87442	2.54971	3.33364
C	1.35269	2.63616	-0.94712
C	2.16209	2.95942	-2.06440
H	2.51857	3.97644	-2.19593
C	2.48724	1.98417	-2.96958
H	3.10663	2.22225	-3.83345
C	2.33227	-0.39442	-3.69475
H	2.94849	-0.18153	-4.56709
C	1.85213	-1.66509	-3.46735
H	2.08284	-2.47180	-4.15993
C	1.05960	-1.93150	-2.33358
H	0.70024	-2.94575	-2.18438
C	0.73704	-0.93827	-1.42155
C	1.22122	0.37689	-1.65986
C	2.02795	0.65227	-2.79723
C	0.94376	3.56649	0.09865
C	0.13135	3.05221	1.14216
C	-0.27242	3.89539	2.16707
H	-0.91541	3.53637	2.96854
C	0.11656	5.23925	2.16473
H	-0.21288	5.89018	2.97318
C	0.91451	5.74812	1.14168
H	1.21368	6.79428	1.14787
C	1.32693	4.91427	0.10865
H	1.94828	5.32120	-0.68673
C	-2.00139	-1.73811	-0.56160
C	-3.01645	-1.57372	0.57301
H	-4.01400	-1.82272	0.18907
H	-3.04728	-0.54145	0.93378
H	-2.81940	-2.23174	1.42447
C	-2.02652	-3.17612	-1.07815
H	-1.33383	-3.33884	-1.90990
H	-3.03404	-3.39330	-1.45669
H	-1.81422	-3.90674	-0.29065
C	-2.35542	-0.76103	-1.68957
H	-2.30820	0.28513	-1.35896
H	-3.38630	-0.95365	-2.01287
H	-1.70431	-0.87822	-2.56236
C	0.60882	-2.39540	1.17837
C	1.11733	-3.64425	0.45650
H	0.31861	-4.22695	-0.01264
H	1.60011	-4.29582	1.19675
H	1.87344	-3.40185	-0.29680
C	-0.30427	-2.79494	2.33994
H	-0.75926	-1.92913	2.83368
H	0.29451	-3.32596	3.09110
H	-1.10256	-3.47512	2.02385
C	1.81329	-1.60257	1.70245
H	2.45713	-1.24838	0.88745
H	2.42204	-2.25828	2.33833
H	1.51411	-0.73945	2.30915
C	-2.61578	1.43351	2.83231
C	-1.29062	0.84471	2.56839
H	-3.50009	0.80403	2.58316
H	-0.84259	0.24466	3.36348

58

ts_2^{CHCHN}-8_triplet

Au	-0.95854	-0.79656	1.79604
P	0.00679	-1.95894	3.68198
N	-0.41753	-2.61388	0.78329

N	-2.89475	0.51819	2.81739
C	-0.71817	-2.63342	-0.51086
C	-0.45513	-3.80955	-1.25999
H	-0.69681	-3.84589	-2.31795
C	0.09846	-4.89677	-0.63610
H	0.30226	-5.80663	-1.19948
C	0.97287	-5.95095	1.44677
H	1.18579	-6.87353	0.90855
C	1.24354	-5.84272	2.79220
H	1.67531	-6.68126	3.33466
C	0.96549	-4.64444	3.48052
H	1.19893	-4.59895	4.54011
C	0.41299	-3.54479	2.84270
C	0.12843	-3.65617	1.45102
C	0.41031	-4.86065	0.74817
C	-1.30907	-1.40415	-1.04532
C	-1.54545	-0.33961	-0.14013
C	-2.09016	0.83989	-0.62788
H	-2.27475	1.68075	0.04040
C	-2.40739	0.97984	-1.98230
H	-2.83416	1.91413	-2.34487
C	-2.17885	-0.07032	-2.86958
H	-2.42407	0.03806	-3.92420
C	-1.63099	-1.25954	-2.40198
H	-1.45108	-2.07000	-3.10579
C	-1.19646	-2.36762	5.07254
C	-1.80529	-1.05725	5.58530
H	-2.58804	-1.30364	6.31453
H	-2.27896	-0.48500	4.78062
H	-1.07719	-0.42149	6.09740
C	-0.58252	-3.13684	6.24123
H	-0.17556	-4.10925	5.94757
H	-1.37170	-3.33491	6.97880
H	0.19790	-2.56520	6.75474
C	-2.30276	-3.19725	4.40803
H	-2.76075	-2.66455	3.56438
H	-3.09500	-3.38326	5.14454
H	-1.94096	-4.16897	4.05405
C	1.66750	-1.23661	4.20876
C	2.58997	-2.22936	4.91680
H	2.14848	-2.65758	5.82230
H	3.50203	-1.69904	5.22121
H	2.90011	-3.04399	4.25469
C	1.41993	-0.02634	5.11128
H	0.69237	0.67535	4.68662
H	2.36305	0.51930	5.24411
H	1.07036	-0.31781	6.10730
C	2.33600	-0.78715	2.90305
H	2.47260	-1.62183	2.20399
H	3.33098	-0.38188	3.12918
H	1.76616	-0.00236	2.39031
C	-2.61821	1.78494	2.54482
C	-1.37850	1.24760	2.55119
H	-3.20614	2.69937	2.47380
H	-0.45815	1.56028	3.04887

58

ts_2^{CHCHN}-8_singlet

Au	-0.87880	-0.83040	1.75761
P	0.04262	-1.93469	3.67310
N	-0.40758	-2.58690	0.79784

N	-3.51477	0.24846	2.84547
C	-0.71862	-2.62133	-0.50172
C	-0.48483	-3.80584	-1.24164
H	-0.73684	-3.84173	-2.29710
C	0.05428	-4.89874	-0.61555
H	0.23597	-5.81650	-1.17360
C	0.92978	-5.95295	1.46387
H	1.12079	-6.88123	0.92748
C	1.21631	-5.84433	2.80598
H	1.63938	-6.68783	3.34751
C	0.96474	-4.63895	3.49105
H	1.20825	-4.59091	4.54829
C	0.42443	-3.53476	2.85031
C	0.12694	-3.64521	1.46459
C	0.37967	-4.85636	0.76478
C	-1.27846	-1.38506	-1.03879
C	-1.45523	-0.31255	-0.12947
C	-1.93684	0.90173	-0.60020
H	-2.05609	1.74698	0.07316
C	-2.26462	1.05452	-1.95057
H	-2.64250	2.01175	-2.30689
C	-2.10995	-0.00584	-2.84258
H	-2.36876	0.11877	-3.89204
C	-1.61501	-1.22312	-2.38956
H	-1.48724	-2.04082	-3.09631
C	-1.18163	-2.30102	5.05533
C	-1.77163	-0.98336	5.56964
H	-2.52157	-1.22461	6.33395
H	-2.28766	-0.43144	4.77881
H	-1.02823	-0.33435	6.04113
C	-0.57028	-3.07588	6.22252
H	-0.16984	-4.05087	5.92868
H	-1.36175	-3.26911	6.95856
H	0.21265	-2.50869	6.73715
C	-2.30101	-3.11504	4.39360
H	-2.76797	-2.56667	3.56536
H	-3.08520	-3.29872	5.13918
H	-1.95332	-4.08839	4.02971
C	1.70873	-1.21400	4.17936
C	2.61156	-2.20915	4.90978
H	2.16217	-2.61038	5.82361
H	3.53092	-1.68645	5.20479
H	2.91112	-3.04202	4.26563
C	1.47787	0.01992	5.05404
H	0.78509	0.73481	4.59652
H	2.43551	0.53782	5.19338
H	1.10278	-0.24159	6.04892
C	2.38431	-0.80346	2.86465
H	2.50551	-1.65358	2.18137
H	3.38749	-0.41595	3.08502
H	1.83193	-0.01275	2.34246
C	-2.87512	1.27603	2.51589
C	-1.46000	0.89920	2.56236
H	-3.28942	2.28482	2.36637
H	-0.87453	1.33853	3.37652

58

ts_2^{NCHCH}-8_singlet

Au	-1.05113	-0.92220	1.84736
P	-0.02511	-1.99045	3.71607
N	-0.45153	-2.59187	0.82569

N	-1.83684	0.66575	2.75638
C	-0.75317	-2.60067	-0.47669
C	-0.34959	-3.69925	-1.27362
H	-0.58183	-3.70954	-2.33431
C	0.32527	-4.74271	-0.69548
H	0.63674	-5.59514	-1.29797
C	1.30483	-5.78578	1.34451
H	1.61985	-6.65271	0.76561
C	1.56186	-5.71133	2.69504
H	2.08304	-6.52193	3.20008
C	1.15636	-4.58236	3.43540
H	1.38271	-4.55619	4.49793
C	0.48994	-3.52284	2.84068
C	0.21502	-3.60223	1.44732
C	0.62543	-4.73371	0.69135
C	-1.51486	-1.44569	-0.94483
C	-1.83529	-0.46187	0.02212
C	-2.61602	0.62764	-0.33643
H	-2.89805	1.36860	0.40958
C	-3.06147	0.76223	-1.65560
H	-3.67576	1.61800	-1.93210
C	-2.73000	-0.19252	-2.61683
H	-3.07862	-0.08000	-3.64145
C	-1.96418	-1.29893	-2.26376
H	-1.72913	-2.04627	-3.01950
C	-1.33578	-2.48557	4.96760
C	-1.71096	-1.24002	5.77513
H	-2.60508	-1.46395	6.37121
H	-1.95171	-0.38755	5.12677
H	-0.91975	-0.94834	6.47426
C	-0.91012	-3.62715	5.89005
H	-0.74787	-4.55977	5.33930
H	-1.72364	-3.81505	6.60310
H	-0.01485	-3.39960	6.47592
C	-2.53959	-2.93989	4.13158
H	-2.95745	-2.12222	3.53234
H	-3.33031	-3.28470	4.81046
H	-2.29154	-3.77755	3.46740
C	1.54443	-1.19943	4.39066
C	2.11754	-1.92349	5.60800
H	1.45593	-1.85869	6.47793
H	3.06187	-1.43651	5.88457
H	2.34846	-2.97571	5.41387
C	1.24154	0.25891	4.74688
H	0.91896	0.82140	3.86591
H	2.16669	0.72250	5.11352
H	0.48878	0.36452	5.53324
C	2.54581	-1.22058	3.22997
H	2.85320	-2.23602	2.95790
H	3.44496	-0.66940	3.53406
H	2.14284	-0.71906	2.34036
C	-1.15512	1.74708	2.54589
C	-0.02082	1.63493	1.71268
H	-1.49063	2.69850	2.98612
H	0.06131	2.14618	0.74613

58

ts_2^{NCHCH}-2^{CHCHN}_triplet

Au	-0.97255	-0.85725	1.78475
P	0.00684	-1.93941	3.67765
N	-0.45342	-2.59237	0.80014

N	-2.11596	0.59182	2.85374
C	-0.79113	-2.63428	-0.49265
C	-0.43299	-3.76539	-1.26636
H	-0.69719	-3.80343	-2.31896
C	0.24448	-4.80069	-0.67774
H	0.52504	-5.67582	-1.26250
C	1.27872	-5.79179	1.36081
H	1.56713	-6.67854	0.79828
C	1.57854	-5.68037	2.70000
H	2.10812	-6.48069	3.21260
C	1.20034	-4.52959	3.42009
H	1.44984	-4.48100	4.47625
C	0.52514	-3.48065	2.81557
C	0.21524	-3.59448	1.43270
C	0.58930	-4.75251	0.69846
C	-1.52952	-1.47378	-0.97526
C	-1.77637	-0.43827	-0.04092
C	-2.51641	0.67198	-0.42129
H	-2.72760	1.46625	0.29069
C	-3.00497	0.76904	-1.72711
H	-3.58824	1.64134	-2.01853
C	-2.75229	-0.23957	-2.65761
H	-3.13339	-0.15427	-3.67318
C	-2.01983	-1.35990	-2.28412
H	-1.84070	-2.14719	-3.01411
C	-1.31530	-2.44966	4.91613
C	-1.67365	-1.23575	5.77615
H	-2.56544	-1.47897	6.36825
H	-1.91612	-0.35812	5.16485
H	-0.87718	-0.97886	6.48277
C	-0.90138	-3.63086	5.79332
H	-0.76000	-4.54554	5.20819
H	-1.71201	-3.82889	6.50688
H	0.00291	-3.43917	6.37863
C	-2.52717	-2.85711	4.06772
H	-2.93769	-2.01152	3.50431
H	-3.31739	-3.22269	4.73635
H	-2.28902	-3.67091	3.37090
C	1.56638	-1.15760	4.38919
C	2.14552	-1.90265	5.59175
H	1.47133	-1.88828	6.45420
H	3.06984	-1.39518	5.89779
H	2.41437	-2.93915	5.36691
C	1.25086	0.28600	4.79229
H	0.85498	0.86421	3.95202
H	2.18278	0.76631	5.11759
H	0.54142	0.35378	5.62163
C	2.57533	-1.14254	3.23496
H	2.88937	-2.14997	2.94114
H	3.47014	-0.59327	3.55479
H	2.17334	-0.63132	2.35067
C	-1.39439	1.67579	2.64045
C	-0.28280	1.33819	1.89263
H	-1.69523	2.65344	3.03512
H	0.55557	1.88092	1.46039

58

ts_2^{NCHCH}-2^{NCCH2}_singlet

Au	-0.49503	1.07108	0.63373
P	-0.36843	-1.20325	-0.05927
N	0.94511	1.37224	-0.77936

N	-2.00727	0.84512	1.87917
C	1.39348	2.62802	-0.87834
C	2.39308	2.93233	-1.83365
H	2.76314	3.94954	-1.92016
C	2.87882	1.94028	-2.64516
H	3.64538	2.16461	-3.38591
C	2.85985	-0.45262	-3.34157
H	3.62398	-0.25301	-4.09136
C	2.35364	-1.72228	-3.17458
H	2.71355	-2.54203	-3.79282
C	1.36829	-1.97199	-2.19782
H	0.99814	-2.98780	-2.08518
C	0.87506	-0.96165	-1.38817
C	1.39672	0.35079	-1.55823
C	2.39527	0.61061	-2.53574
C	0.76786	3.58310	0.03260
C	-0.23614	3.07438	0.89347
C	-0.89996	3.93750	1.75352
H	-1.69657	3.56751	2.39604
C	-0.56080	5.29434	1.78357
H	-1.08679	5.96649	2.46021
C	0.43807	5.79468	0.94854
H	0.69562	6.85149	0.97692
C	1.10014	4.94351	0.07015
H	1.87110	5.34818	-0.58313
C	-1.96610	-1.75580	-0.87454
C	-2.97987	-2.05800	0.23251
H	-3.96581	-2.20304	-0.22798
H	-3.06117	-1.22645	0.94414
H	-2.73909	-2.97844	0.77595
C	-1.80530	-2.95254	-1.81137
H	-1.17605	-2.71663	-2.67618
H	-2.79683	-3.21797	-2.20124
H	-1.40724	-3.84132	-1.31271
C	-2.43531	-0.54048	-1.68584
H	-2.67040	0.31580	-1.04262
H	-3.35331	-0.80783	-2.22506
H	-1.69412	-0.23186	-2.43414
C	0.39594	-2.36874	1.20395
C	0.36952	-3.83323	0.76737
H	-0.65009	-4.22448	0.69042
H	0.88841	-4.43232	1.52726
H	0.88704	-4.00658	-0.18233
C	-0.35554	-2.20406	2.52940
H	-0.26559	-1.19117	2.93580
H	0.08725	-2.88925	3.26392
H	-1.41868	-2.44909	2.45212
C	1.84193	-1.89549	1.39182
H	2.45967	-2.07404	0.50529
H	2.28706	-2.44946	2.22811
H	1.89519	-0.82825	1.64469
C	-1.82064	1.01370	3.12223
H	-0.98618	1.93697	4.86483
C	-0.95839	1.06377	4.20071
H	-2.73167	0.91511	3.81603

58

ts_2-2$^{NCHCH'}$_triplet

Au	2.02571	9.77285	5.48184
P	2.13334	7.51311	4.74273
N	3.48597	10.07658	4.05599

N	0.65700	9.60798	6.84875
C	3.91403	11.33703	3.94508
C	4.93345	11.64101	3.01036
H	5.29293	12.66132	2.91515
C	5.45494	10.64114	2.23006
H	6.23601	10.86437	1.50422
C	5.47494	8.23689	1.56510
H	6.25135	8.43219	0.82683
C	4.96698	6.96784	1.73158
H	5.33658	6.14533	1.12276
C	3.96391	6.72121	2.69164
H	3.58569	5.70731	2.79254
C	3.46029	7.73415	3.49191
C	3.97397	9.04950	3.31162
C	4.98835	9.30594	2.35020
C	3.24585	12.29828	4.82243
C	2.24493	11.78857	5.68657
C	1.53139	12.65677	6.50135
H	0.73370	12.27877	7.14105
C	1.82119	14.02562	6.48710
H	1.25887	14.70244	7.12881
C	2.81978	14.52849	5.65347
H	3.04093	15.59390	5.64761
C	3.52819	13.67030	4.81823
H	4.29378	14.07990	4.16167
C	0.57210	7.02842	3.81332
C	-0.63065	7.23916	4.74065
H	-1.54802	7.10453	4.15341
H	-0.65511	8.24592	5.17399
H	-0.66227	6.51893	5.56282
C	0.59485	5.59157	3.29494
H	1.43524	5.39711	2.61978
H	-0.32344	5.41353	2.72001
H	0.61273	4.85620	4.10607
C	0.47512	8.01248	2.64081
H	0.46837	9.05709	2.97904
H	-0.47130	7.83727	2.11360
H	1.28801	7.88606	1.91724
C	2.76599	6.31616	6.04010
C	3.32525	5.01327	5.46745
H	2.60216	4.46397	4.85674
H	3.60270	4.36068	6.30563
H	4.23422	5.18023	4.88042
C	1.61718	6.02487	7.00930
H	1.12354	6.94082	7.35653
H	2.02399	5.50908	7.88887
H	0.86389	5.36448	6.56689
C	3.88780	7.07204	6.76447
H	4.68988	7.37406	6.07897
H	4.33336	6.40754	7.51621
H	3.51498	7.95921	7.28881
C	1.69916	9.76258	8.63424
H	2.06065	10.75071	8.40088
C	1.59615	8.80222	9.40278
H	1.38647	7.90585	9.95399

58

ts_2-2^{NCHCH}_triplet

Au	2.05970	9.75892	5.46736
P	2.17241	7.49967	4.72146
N	3.49734	10.07645	4.02687

N	0.67592	9.56799	6.81438
C	3.91529	11.34063	3.91409
C	4.90537	11.65800	2.95229
H	5.25434	12.68168	2.85421
C	5.41050	10.66771	2.14989
H	6.16912	10.90136	1.40379
C	5.42920	8.26875	1.46774
H	6.18495	8.47507	0.71124
C	4.93560	6.99456	1.63860
H	5.29648	6.17874	1.01573
C	3.95817	6.73474	2.62114
H	3.59027	5.71731	2.72524
C	3.46609	7.73896	3.43950
C	3.96848	9.05847	3.25863
C	4.95580	9.32853	2.27297
C	3.27489	12.28832	4.82410
C	2.30147	11.76570	5.71122
C	1.63985	12.61334	6.58788
H	0.89148	12.21539	7.27091
C	1.94106	13.97908	6.59517
H	1.41901	14.64174	7.28400
C	2.90364	14.49847	5.72868
H	3.13273	15.56218	5.74165
C	3.57033	13.65811	4.84352
H	4.31520	14.07773	4.16967
C	0.59702	6.98707	3.83056
C	-0.58427	7.16346	4.79177
H	-1.51407	7.01375	4.22812
H	-0.61825	8.16516	5.23578
H	-0.57622	6.43335	5.60596
C	0.63246	5.55522	3.29923
H	1.46105	5.37827	2.60519
H	-0.29492	5.36713	2.74234
H	0.67832	4.81460	4.10444
C	0.45066	7.97982	2.67045
H	0.43071	9.02074	3.01923
H	-0.50400	7.78994	2.16347
H	1.24873	7.87749	1.92676
C	2.86506	6.32278	6.00764
C	3.39244	5.00774	5.43266
H	2.63702	4.44660	4.87456
H	3.71529	4.37275	6.26815
H	4.26822	5.16009	4.79335
C	1.76715	6.05694	7.04107
H	1.30601	6.98398	7.40268
H	2.21275	5.54898	7.90594
H	0.98274	5.40165	6.64797
C	4.02269	7.08784	6.66206
H	4.79205	7.37257	5.93326
H	4.49916	6.43811	7.40745
H	3.67271	7.98621	7.18291
C	1.45567	9.62215	8.73324
H	0.52543	9.29653	9.16432
C	2.62800	10.00299	8.75628
H	3.63108	10.36691	8.64653

58

ts_2-2$^{NCHCH'}$_singlet

Au	2.01997	9.79464	5.46616
P	2.12310	7.52996	4.74397
N	3.48413	10.08379	4.03981

N	0.66240	9.64348	6.82560
C	3.91540	11.34230	3.91820
C	4.93529	11.63625	2.98083
H	5.29600	12.65522	2.87613
C	5.45714	10.62808	2.21150
H	6.23965	10.84325	1.48478
C	5.47881	8.21622	1.57405
H	6.25818	8.40281	0.83665
C	4.97006	6.94917	1.75316
H	5.34220	6.11962	1.15556
C	3.96283	6.71378	2.71165
H	3.58348	5.70132	2.82265
C	3.45682	7.73610	3.49833
C	3.97229	9.04905	3.30604
C	4.98969	9.29439	2.34503
C	3.25403	12.31095	4.79243
C	2.25848	11.80895	5.66695
C	1.56118	12.68059	6.49200
H	0.77311	12.30424	7.14453
C	1.85740	14.04796	6.47153
H	1.30724	14.72861	7.11974
C	2.84648	14.54484	5.62289
H	3.07138	15.60944	5.61114
C	3.54215	13.68175	4.78192
H	4.30263	14.08649	4.11644
C	0.56446	7.04321	3.81147
C	-0.64256	7.26954	4.72946
H	-1.55708	7.14155	4.13631
H	-0.65962	8.27840	5.15876
H	-0.68691	6.55336	5.55454
C	0.58465	5.60072	3.30885
H	1.42598	5.39742	2.63739
H	-0.33296	5.41865	2.73409
H	0.59935	4.87400	4.12779
C	0.47884	8.01496	2.62783
H	0.47332	9.06310	2.95491
H	-0.46458	7.83717	2.09616
H	1.29615	7.87784	1.91126
C	2.74189	6.34527	6.05890
C	3.30686	5.03811	5.50218
H	2.58971	4.48359	4.88910
H	3.57695	4.39270	6.34823
H	4.22094	5.20171	4.92217
C	1.58270	6.06397	7.01867
H	1.08724	6.98421	7.35191
H	1.98012	5.55639	7.90725
H	0.83333	5.40012	6.57480
C	3.85557	7.10758	6.78887
H	4.66130	7.41246	6.10890
H	4.29860	6.44703	7.54555
H	3.47274	7.99279	7.30897
C	1.78092	9.78928	8.67862
H	2.19804	10.73616	8.38181
C	1.57625	8.83914	9.43159
H	1.29528	7.97124	9.99677

58

ts_2-2^{NCHCH}_singlet

Au	2.05978	9.74961	5.46714
P	2.16933	7.49556	4.71212
N	3.49625	10.07201	4.02519
N	0.70124	9.53811	6.81431
C	3.91516	11.33611	3.91477
C	4.90418	11.65536	2.95249
H	5.25316	12.67920	2.85611
C	5.40859	10.66662	2.14773
H	6.16651	10.90162	1.40135
C	5.42795	8.26825	1.46283
H	6.18323	8.47574	0.70617
C	4.93556	6.99339	1.63279
H	5.29710	6.17845	1.00914
C	3.95841	6.73191	2.61510
H	3.59110	5.71416	2.71863
C	3.46575	7.73547	3.43396
C	3.96746	9.05552	3.25480
C	4.95430	9.32705	2.26921
C	3.27628	12.28158	4.82811
C	2.30448	11.75672	5.71545
C	1.64053	12.60247	6.59245
H	0.89124	12.20163	7.27273
C	1.94020	13.96843	6.60156
H	1.41685	14.62970	7.29077
C	2.90236	14.49008	5.73580
H	3.12988	15.55412	5.74993
C	3.56974	13.65187	4.84915
H	4.31314	14.07352	4.17493
C	0.59462	6.98400	3.82054
C	-0.58823	7.17165	4.77770
H	-1.51744	7.02345	4.21270
H	-0.61827	8.17699	5.21466
H	-0.58608	6.44739	5.59702
C	0.62815	5.54804	3.29996
H	1.45683	5.36552	2.60737
H	-0.29927	5.35671	2.74425
H	0.67356	4.81312	4.11046
C	0.45403	7.96901	2.65325
H	0.43671	9.01229	2.99500
H	-0.50025	7.77863	2.14576
H	1.25318	7.85907	1.91186
C	2.85405	6.32215	6.00497
C	3.39888	5.01387	5.43078
H	2.65519	4.45011	4.85949
H	3.71526	4.37685	6.26716
H	4.28173	5.17654	4.80373
C	1.74454	6.04497	7.02290
H	1.26649	6.96781	7.37412
H	2.18414	5.54464	7.89526
H	0.97404	5.37889	6.62037
C	3.99654	7.09422	6.67797
H	4.76930	7.39642	5.95971
H	4.47434	6.44148	7.41991
H	3.63146	7.98228	7.20642
C	1.52866	9.62928	8.80354
H	0.60944	9.23639	9.19729
C	2.66530	10.09388	8.78745
H	3.63871	10.52848	8.66897

10 References

(1) Fulmer, G. R.; Miller, A. J. M.; Sherden, N. H.; Gottlieb, H. E.; Nudelman, A.; Stoltz, B. M.; Bercaw, J. E.; Goldberg, K. I. NMR Chemical Shifts of Trace Impurities: Common Laboratory Solvents, Organics, and Gases in Deuterated Solvents Relevant to the Organometallic Chemist. *Organometallics* **2010**, *29*, 2176-2179.

(2) Martín, J.; Schörgenhumer, J.; Biedrzycki, M.; Nevado, C. *Inorg. Chem.* **2024**, *63*, 8390-8396.

(3) Slagt, M. Q.; Dijkstra, H. P.; McDonald, A.; Klein Gebbink, R. J. M.; Lutz, M.; Ellis, D. D.; Mills, A. M.; Spek, A. L.; van Koten, G. Self-Assembly of p-Nitro NCN-Pincer Palladium Complexes into Dimers through Electron Donor−Acceptor Interactions. *Organometallics* **2003**, *22*, 27-29.

(4) Pompidor, G.; Dworkowski, F. S. N.; Thominet, V.; Schulze-Briese, C.; Fuchs, M. R. A new on-axis micro-spectrophotometer for combining Raman, fluorescence and UV/Vis absorption spectroscopy with macromolecular crystallography at the Swiss Light Source. *J. Synchrotron Rad.*, **2013**, *20*, 765–776.

(5) Winter, G.; Beilsten-Edmands, J.; Devenish, N.; Gerstel, M.; Gildea, R. J.; McDonagh, D.; Pascal, E.; Waterman, D. G.; Williams, B. H.; Evans, G. DIALS as a toolkit. *Protein Sci.* **2022**, *31*, 232-250.

(6) Sheldrick, G. M. SHELXT - Integrated space-group and crystal-structure determination. *Acta Cryst. A*, **2015**, *71*, 3-8.

(7) Chadwick, F. M.; McKay, A. I.; Martinez-Martinez, A. J.; Rees, N. H.; Krämer, T.; Macgregor, S. A.; Weller, A. S. Solid-state molecular organometallic chemistry. Single-crystal to single-crystal reactivity and catalysis with light hydrocarbon substrates. *Chem. Sci.* **2017**, *8*, 6014-6029.

(8) Jung, H. M.; Kweon, J.; Suh, J. M.; Lim, M. H.; Kim, D.; Chang, S. K. Mechanistic snapshots of rhodium-catalyzed acylnitrene transfer reactions. *Science* **2023**, *381*, 525-532.

(9) Themed issue: Polymorphism and crystal forms. *New J. Chem.* **2008**, *32*, 1657-1658.

(10) a) Winter, G.; Waterman, D. G.; Parkhurst, J. M.; Brewster, A. S.; Gildea, R. J.; Gerstel, M.; Fuentes-Montero, L.; Vollmar, M.; Michels-Clark, T.; Young, I. D.; Sauter, N. K.; Evans, G. DIALS: implementation and evaluation of a new integration package. *Acta Cryst. D* **2018**, *74*, 85-97; b) Beilsten-Edmands, J; Winter, G.; Gildea, R.; Parkhurst, J.; Watermanb, D.; Evans G. Scaling diffraction data in the DIALS software package: algorithms and new approaches for multi-crystal scaling. *Acta Cryst. D* **2020**, *76*, 385-399.

(11) Sheldrick, G. Crystal structure refinement with SHELXL. *Acta Cryst. C* **2015**, *71*, 3-8.

(12) Dolomanov, O. V.; Bourhis, L. J.; Gildea, R. J.; Howard, J. A. K.; Puschmann, H. OLEX2: a complete structure solution, refinement and analysis program. *J. Appl. Cryst.* **2009**, *42*, 339-341.

(13) Macrae, C. F.; Sovago, I.; Cottrell, S. J.; Galek, P. T. A.; McCabe, P.; Pidcock, E.; Platings, M.; Shields, G. P.; Stevens, J. S.; Towler, M.; Wood, P. A. Mercury 4.0: from visualization to analysis, design and prediction. *J. Appl. Cryst.* **2020**, *53*, 226-235.

(14) Thorn, A.; Dittrich, B.; Sheldrick, G. M. Enhanced rigid-bond restraints. *Acta Cryst. A* **2012**, *68*, 448-451.

(15) Neese, F. The ORCA program system. *WIREs Comput. Mol. Sci.* **2012**, *2*, 73-78.

(16) Neese, F.; Wennmohs, F.; Becker, U.; Riplinger, C. The ORCA quantum chemistry program package. *J. Chem. Phys.* **2020**, *152*, 224108.

(17) Neese, F. Software update: The ORCA program system—Version 5.0. *WIREs Comput. Mol. Sci.* **2022**, *12*, e1606.

(18) Adamo, C.; Barone, V. Toward reliable density functional methods without adjustable parameters: The PBE0 model. *J. Chem. Phys.* **1999**, *110*, 6158-6170.

(19) Weigend, F.; Ahlrichs, R. Balanced basis sets of split valence, triple zeta valence and quadruple zeta valence quality for H to Rn: Design and assessment of accuracy. *Phys. Chem. Chem. Phys.* **2005**, *7*, 3297-3305.

(20) Pantazis, D. A.; Chen, X.-Y.; Landis, C. R.; Neese, F. All-Electron Scalar Relativistic Basis Sets for Third-Row Transition Metal Atoms. *J. Chem. Theory Comput.* **2008**, *4*, 908-919.

(21) Caldeweyher, E.; Ehlert, S.; Hansen, A.; Neugebauer, H.; Spicher, S.; Bannwarth, C.; Grimme, S. A generally applicable atomic-charge dependent London dispersion correction. *J. Chem. Phys.* **2019**, *150*, 154122.

(22) Lenthe, E. v.; Baerends, E. J.; Snijders, J. G. Relativistic regular two-component Hamiltonians. *J. Chem. Phys.* **1993**, *99*, 4597-4610.

(23) van Lenthe, E.; Ehlers, A.; Baerends, E.-J. Geometry optimizations in the zero order regular approximation for relativistic effects. *J. Chem. Phys.* **1999**, *110*, 8943-8953.

(24) Grimme, S.; Hansen, A.; Ehlert, S.; Mewes, J.-M. r^2SCAN-3c: A “Swiss army knife” composite electronic-structure method. *J. Chem. Phys.* **2021**, *154*, 064103.

(25) Ásgeirsson, V.; Birgisson, B. O.; Bjornsson, R.; Becker, U.; Neese, F.; Riplinger, C.; Jónsson, H. Nudged Elastic Band Method for Molecular Reactions Using Energy-Weighted Springs Combined with Eigenvector Following. *J. Chem. Theory Comput.* **2021**, *17*, 4929-4945.

(26) Cossi, M.; Rega, N.; Scalmani, G.; Barone, V. Energies, structures, and electronic properties of molecules in solution with the C-PCM solvation model. *J. Comput. Chem.* **2003**, *24*, 669-681.

(27) Mardirossian, N.; Head-Gordon, M. ωB97X-V: A 10-parameter, range-separated hybrid, generalized gradient approximation density functional with nonlocal correlation, designed by a survival-of-the-fittest strategy. *Phys. Chem. Chem. Phys.* **2014**, *16*, 9904-9924.

(28) Maurer, L. R.; Bursch, M.; Grimme, S.; Hansen, A. Assessing Density Functional Theory for Chemically Relevant Open-Shell Transition Metal Reactions. *J. Chem. Theory Comput.* **2021**, *17*, 6134-6151.

(29) Neese, F.; Wennmohs, F.; Hansen, A.; Becker, U. Efficient, approximate and parallel Hartree–Fock and hybrid DFT calculations. A ‘chain-of-spheres’ algorithm for the Hartree–Fock exchange. *Chem. Phys.* **2009**, *356*, 98-109.

(30) Izsák, R.; Neese, F. An overlap fitted chain of spheres exchange method. *J. Chem. Phys.* **2011**, *135*, 144105.

(31) Weigend, F. Accurate Coulomb-fitting basis sets for H to Rn. *Phys. Chem. Chem. Phys.* **2006**, *8*, 1057-1065.

(32) Stoychev, G. L.; Auer, A. A.; Neese, F. Automatic Generation of Auxiliary Basis Sets. *J. Chem. Theory Comput.* **2017**, *13*, 554-562.

(33) Glendening, E. D.; Badenhoop, J, K.; Reed, A. E.; Carpenter, J. E.; Bohmann, J. A.; Morales, C. M.; Karafiloglou, P.; Landis, C. R.; Weinhold, F. ***NBO 7.0.*** Theoretical Chemistry Institute, University of Wisconsin, Madison (2018).

(34) Sun, J.; Abbenseth, J.; Verplancke, H.; Diefenbach, M.; de Bruin, B.; Hunger, D.; Würtele, C.; van Slageren, J.; Holthausen, M. C.; Schneider, S. A platinum(II) metallonitrene with a triplet ground state. *Nat. Chem.* **2020**, *12*, 1054-1059.

(35) Knizia, G. Intrinsic Atomic Orbitals: An Unbiased Bridge between Quantum Theory and Chemical Concepts. *J. Chem. Theory Comput.* **2013**, *9*, 4834-4843.

(36) Schneider, W. B.; Bistoni, G.; Sparta, M.; Saitow, M.; Riplinger, C.; Auer, A. A.; Neese, F. Decomposition of Intermolecular Interaction Energies within the Local Pair Natural Orbital Coupled Cluster Framework. *J. Chem. Theory Comput.* **2016**, *12*, 4778-4792.

(37) Drosou, M.; Mitsopoulou, C. A.; Pantazis, D. A. Reconciling Local Coupled Cluster with Multireference Approaches for Transition Metal Spin-State Energetics. *J. Chem. Theory Comput.* **2022**, *18*, 3538-3548.

(38) Guo, Y.; Riplinger, C.; Liakos, D. G.; Becker, U.; Saitow, M.; Neese, F. Linear scaling perturbative triples correction approximations for open-shell domain-based local pair natural orbital coupled cluster singles and doubles theory [DLPNO-CCSD(T/T)]. *J. Chem. Phys.* **2020**, *152*, 024116.

(39) Knizia, G. IBOView – A program for chemical analysis. http://www.iboview.org/.

(40) Chemcraft - graphical software for visualization of quantum chemistry computations. https://www.chemcraftprog.com.

(41) Schmidt-Räntsch, T.; Verplancke, H.; Lienert, J. N.; Demeshko, S.; Otte, M.; Van Trieste III, G. P.; Reid, K. A.; Reibenspies, J. H.; Powers, D. C.; Holthausen, M. C.; Schneider, S. Nitrogen Atom Transfer Catalysis by Metallonitrene C−H Insertion: Photocatalytic Amidation of Aldehydes. *Angew. Chem. Int. Ed.* **2022**, *61*, e202115626.

(42) Geerlings, P.; De Proft, F.; Langenaeker, W. Conceptual Density Functional Theory. *Chem. Rev.* **2003**, *103*, 1793-1874.

(43) Gritsenko, O. V.; Baerends, E. J. The spin-unrestricted molecular Kohn–Sham solution and the analogue of Koopmans's theorem for open-shell molecules. *J. Chem. Phys.* **2004**, *120*, 8364-8372.
