# Supplementary material for: A photogenerated triplet aura-nitrene for gold-mediated nitrene transfer
Source: Nat Chem. 2026 Jul 21;18(8):1413–21. doi: 10.1038/s41557-026-02152-3 (PMC13423833; doi:10.1038/s41557-026-02152-3)
Supplement: Supplementary file 2 — Coordinates for structures calculated by density functional theory. [file 41557_2026_2152_MOESM2_ESM.txt]

**XYZ Coordinates**

**O2**

O 10.19390 2.65582 8.14721

O 10.45367 2.32154 9.25670

**NO**

N 10.19890 2.64939 8.16856

O 10.44867 2.32797 9.23534

**N2** -109.5462448

N 10.20373 2.64317 8.18922

N 10.44383 2.33420 9.21468

**acetylene**

H 3.31144 4.33146 -8.39605

C 3.31144 4.33146 -7.32448

C 3.31144 4.33146 -6.11532

H 3.31144 4.33146 -5.04375

**1_triplet**

Au 8.42797 3.41805 6.34779

P 7.64466 1.26611 5.58385

N 6.36959 3.96925 5.66432

N 10.48169 3.06781 6.83372

N 10.85778 2.72335 7.92178

N 11.21657 2.38684 8.96585

C 5.97956 1.70487 4.96111

C 5.11252 0.80329 4.36414

H 5.40726 -0.23400 4.23123

C 3.83204 1.19110 3.91951

H 3.18285 0.45130 3.45571

C 3.40318 2.49003 4.07824

H 2.41106 2.79183 3.74524

C 4.25033 3.44828 4.67698

C 5.54802 3.05523 5.10919

C 3.88309 4.80438 4.88032

H 2.89509 5.13749 4.56465

C 4.75292 5.68623 5.46783

H 4.46292 6.72074 5.62575

C 6.03917 5.23567 5.86650

C 7.06564 6.06718 6.50442

C 8.29072 5.45663 6.84996

C 9.28761 6.21117 7.45863

H 10.24479 5.76488 7.72168

C 9.07489 7.56618 7.73317

H 9.86207 8.14843 8.21049

C 7.86669 8.17235 7.39470

H 7.70475 9.22720 7.60715

C 6.86599 7.42708 6.78036

H 5.92923 7.91529 6.51805

C 8.64858 0.63021 4.12020

C 8.42496 1.65823 3.00493

H 7.38904 1.66887 2.64925

H 9.06975 1.40371 2.15415

H 8.69176 2.67434 3.32440

C 10.13170 0.61832 4.50262

H 10.48833 1.60572 4.81489

H 10.71414 0.32135 3.62097

H 10.35894 -0.09936 5.29681

C 8.22841 -0.76371 3.65261

H 8.41213 -1.53152 4.41149

H 8.82994 -1.03030 2.77344

H 7.17946 -0.81060 3.34411

C 7.36782 0.07819 7.01844

C 6.71374 0.92672 8.11604

H 7.36733 1.73806 8.46056

H 6.49659 0.28607 8.98053

H 5.76483 1.36624 7.78484

C 6.44557 -1.09495 6.68070

H 5.42547 -0.76563 6.45942

H 6.38674 -1.74888 7.56078

H 6.80972 -1.70578 5.84888

C 8.72389 -0.44054 7.49911

H 9.18274 -1.13256 6.78514

H 8.58007 -0.99034 8.43795

H 9.43250 0.36996 7.70389

**1_singlet**

Au 8.12845 3.29112 6.55065

P 7.54069 1.14003 5.68046

N 6.37916 3.88309 5.74261

N 9.77945 2.55558 7.37121

N 10.64487 3.20624 7.91743

N 11.51319 3.71061 8.45032

C 5.90900 1.62484 5.00699

C 5.03517 0.76282 4.36210

H 5.29178 -0.28728 4.25218

C 3.80805 1.21101 3.83622

H 3.14761 0.50350 3.33933

C 3.45114 2.53583 3.94959

H 2.50541 2.89339 3.54519

C 4.31079 3.45438 4.59180

C 5.54593 2.99320 5.12340

C 4.00065 4.83144 4.73210

H 3.05908 5.20525 4.33189

C 4.86948 5.68184 5.36177

H 4.63185 6.73533 5.47194

C 6.08793 5.18342 5.88089

C 7.08901 5.97153 6.58233

C 8.23156 5.27579 7.05336

C 9.21525 5.98034 7.73043

H 10.10999 5.49585 8.11293

C 9.07765 7.35743 7.94148

H 9.86072 7.89424 8.47481

C 7.95595 8.04182 7.47818

H 7.85799 9.11233 7.64600

C 6.96054 7.34995 6.79882

H 6.08810 7.89083 6.43742

C 8.60644 0.63494 4.21488

C 8.43916 1.75987 3.18598

H 7.41606 1.82735 2.79974

H 9.10333 1.55968 2.33568

H 8.72410 2.73767 3.59593

C 10.07132 0.57886 4.66285

H 10.39053 1.50259 5.15641

H 10.69713 0.43213 3.77326

H 10.27168 -0.25157 5.34498

C 8.19068 -0.70336 3.60567

H 8.32005 -1.53673 4.30412

H 8.83493 -0.90652 2.73998

H 7.15838 -0.70063 3.23956

C 7.25554 -0.13905 7.02516

C 6.60284 0.63541 8.17718

H 7.27729 1.39230 8.59459

H 6.35965 -0.06907 8.98303

H 5.66631 1.12052 7.87335

C 6.33388 -1.28268 6.59909

H 5.30950 -0.94152 6.41696

H 6.28869 -2.00879 7.42131

H 6.69485 -1.81871 5.71562

C 8.61528 -0.68348 7.47247

H 9.05293 -1.35815 6.72892

H 8.46912 -1.26623 8.39109

H 9.32910 0.11905 7.69276

**2^NO2^_triplet**

Au 2.26542 9.85066 5.45395

P 2.25927 7.55592 4.76575

O 0.19851 8.84061 7.21117

O -0.33577 9.98808 6.54682

N 3.60290 10.07107 3.94441

N 0.98802 9.96553 7.00713

C 4.08189 11.39046 3.75680

C 5.00922 11.63220 2.71623

H 5.37678 12.64380 2.56443

C 5.44139 10.61723 1.91214

H 6.15675 10.80057 1.11402

C 5.36698 8.19109 1.35099

H 6.09324 8.34936 0.55551

C 4.86885 6.90591 1.59723

H 5.21154 6.06808 0.99312

C 3.93004 6.68701 2.61139

H 3.56052 5.67883 2.77038

C 3.47065 7.73887 3.39850

C 3.99585 9.04965 3.16732

C 4.95287 9.27587 2.11519

C 3.55762 12.32718 4.66663

C 2.61817 11.83535 5.65996

C 2.08997 12.68642 6.60611

H 1.40571 12.30126 7.36279

C 2.44229 14.04446 6.59880

H 2.02308 14.71930 7.34304

C 3.34883 14.54667 5.62489

H 3.61077 15.60309 5.63613

C 3.89836 13.71825 4.68142

H 4.59572 14.11773 3.94877

C 0.63971 6.98537 3.97868

C -0.30659 6.44836 5.05419

H -1.28363 6.26422 4.58908

H -0.45606 7.15836 5.87111

H 0.03655 5.49536 5.47020

C 0.84505 5.92272 2.89851

H 1.41264 6.30299 2.04355

H -0.14335 5.62542 2.52453

H 1.32985 5.01629 3.27698

C 0.05008 8.25168 3.34525

H -0.21540 8.99867 4.10301

H -0.86824 7.98667 2.80528

H 0.73465 8.70780 2.61807

C 3.04481 6.39298 6.02108

C 3.02254 4.92920 5.58020

H 2.00669 4.54081 5.45687

H 3.50652 4.32433 6.35827

H 3.58157 4.75805 4.65465

C 2.30625 6.55379 7.35412

H 2.37714 7.57689 7.73975

H 2.77813 5.89351 8.09310

H 1.24926 6.28014 7.29630

C 4.49117 6.87235 6.19022

H 5.09339 6.70643 5.29066

H 4.95481 6.31125 7.01159

H 4.54550 7.93717 6.45334

**2^NO2^_isomer_singlet**

Au 2.22282 9.84432 5.43828

P 2.28159 7.55100 4.73567

O -0.03395 10.55929 7.09950

O 0.96937 10.12721 8.02684

N 3.60600 10.10186 3.94230

N 0.78787 9.48005 6.81050

C 4.07273 11.34747 3.80676

C 5.00863 11.62888 2.78184

H 5.39007 12.63853 2.66393

C 5.41989 10.62475 1.94577

H 6.13907 10.83089 1.15398

C 5.31587 8.23096 1.26838

H 6.03516 8.41502 0.47168

C 4.80212 6.96976 1.47358

H 5.11193 6.14066 0.84094

C 3.87288 6.74343 2.50786

H 3.49358 5.73491 2.65030

C 3.44385 7.76777 3.33750

C 3.98006 9.06913 3.13582

C 4.91984 9.30506 2.09537

C 3.54589 12.31522 4.76100

C 2.58631 11.84199 5.69044

C 2.06241 12.71683 6.62882

H 1.32723 12.37392 7.35111

C 2.47638 14.05322 6.65119

H 2.05664 14.73037 7.39366

C 3.41681 14.52287 5.73563

H 3.73349 15.56357 5.75980

C 3.95275 13.65572 4.79048

H 4.69025 14.03000 4.08312

C 0.64166 6.95060 4.04433

C -0.26614 6.57099 5.21876

H -1.27951 6.39791 4.83404

H -0.32463 7.36482 5.97258

H 0.05666 5.64397 5.70473

C 0.77233 5.78043 3.06934

H 1.26594 4.90741 3.50799

H 1.29754 6.06518 2.15164

H -0.23751 5.46536 2.77499

C 0.05844 8.16099 3.30402

H -0.15515 8.99559 3.98278

H -0.89082 7.86960 2.83631

H 0.72105 8.51829 2.50520

C 3.13482 6.43592 5.98795

C 3.09876 4.95894 5.59819

H 2.08092 4.55598 5.58149

H 3.66087 4.38471 6.34650

H 3.57077 4.76572 4.62863

C 2.46321 6.64447 7.35088

H 2.54422 7.68264 7.69159

H 2.97566 6.01633 8.09080

H 1.40581 6.36685 7.35806

C 4.58467 6.92726 6.07036

H 5.14755 6.72998 5.15185

H 5.08818 6.40071 6.89112

H 4.64509 8.00163 6.28972

**2^NO2^_singlet**

Au 2.29891 9.83461 5.48538

P 2.29268 7.54222 4.77825

O 0.17295 8.88226 7.18456

O -0.17505 10.20868 6.78282

N 3.58244 10.09682 3.90893

N 1.14170 9.87601 7.12669

C 4.00163 11.35421 3.72198

C 4.89823 11.63395 2.66310

H 5.23773 12.65258 2.50077

C 5.33681 10.61359 1.86012

H 6.03444 10.81626 1.04837

C 5.33481 8.18862 1.29332

H 6.03933 8.36538 0.48193

C 4.87823 6.91827 1.56442

H 5.21975 6.07335 0.97016

C 3.95984 6.70203 2.61083

H 3.61554 5.68734 2.78948

C 3.49105 7.74221 3.39847

C 3.98053 9.05066 3.13569

C 4.89792 9.28135 2.07374

C 3.48239 12.33198 4.67250

C 2.61653 11.83856 5.67543

C 2.10392 12.69337 6.63735

H 1.45637 12.31356 7.42744

C 2.43272 14.05307 6.59838

H 2.02598 14.72626 7.35157

C 3.27945 14.55024 5.60744

H 3.53178 15.60848 5.58665

C 3.80869 13.69410 4.64688

H 4.47455 14.09508 3.88488

C 0.66980 6.98665 3.99102

C -0.28024 6.44332 5.06031

H -1.25484 6.25882 4.59022

H -0.43609 7.15223 5.87727

H 0.06234 5.48948 5.47459

C 0.86986 5.93448 2.89926

H 1.35065 5.02235 3.26926

H 1.44032 6.32030 2.04854

H -0.11902 5.64487 2.52064

C 0.08441 8.26243 3.37264

H -0.16894 9.00695 4.13775

H -0.84095 8.01047 2.83854

H 0.76579 8.72037 2.64370

C 3.06971 6.36977 6.02500

C 3.04473 4.90685 5.58106

H 2.02825 4.52374 5.44669

H 3.51840 4.29702 6.36151

H 3.61105 4.73521 4.65983

C 2.32234 6.53247 7.35394

H 2.39273 7.55493 7.74206

H 2.78584 5.87115 8.09715

H 1.26508 6.26043 7.28870

C 4.51622 6.84434 6.20480

H 5.12170 6.68807 5.30564

H 4.97613 6.27353 7.02155

H 4.57130 7.90597 6.48039

**2^O^+NO_triplet**

Au -0.27888 1.13935 -0.92883

P 0.92537 0.22128 0.89189

O 1.57759 2.32579 -4.09148

O 0.52397 2.86131 -0.93689

N -1.18613 -0.63980 -0.97584

N 1.96345 1.60319 -3.29893

C -2.17571 -0.79261 -1.98463

C -2.86228 -2.02299 -2.07364

H -3.62101 -2.14550 -2.84240

C -2.58696 -3.04736 -1.21213

H -3.11594 -3.99477 -1.28144

C -1.24538 -3.88066 0.71721

H -1.76090 -4.83816 0.66645

C -0.25552 -3.67259 1.68917

H -0.00803 -4.47063 2.38654

C 0.41560 -2.44917 1.77346

H 1.17658 -2.31685 2.53774

C 0.11052 -1.41501 0.89223

C -0.89207 -1.62363 -0.10721

C -1.58221 -2.88333 -0.19006

C -2.35091 0.35222 -2.78707

C -1.52611 1.50445 -2.48300

C -1.63996 2.66277 -3.22187

H -1.01663 3.52532 -2.99068

C -2.56009 2.72601 -4.28011

H -2.64775 3.63707 -4.86944

C -3.37655 1.60771 -4.59133

H -4.08451 1.67721 -5.41515

C -3.28241 0.44513 -3.86864

H -3.91532 -0.40297 -4.11988

C 0.48189 1.08034 2.49972

C 1.17736 2.44413 2.51122

H 0.76637 3.03964 3.33649

H 1.00874 3.00459 1.58238

H 2.25526 2.35460 2.68204

C 0.84623 0.26781 3.74252

H 1.91306 0.03499 3.80782

H 0.27263 -0.66263 3.80725

H 0.59064 0.86370 4.62843

C -1.03972 1.26982 2.44692

H -1.34597 1.92657 1.62409

H -1.36663 1.74120 3.38265

H -1.57268 0.31518 2.35602

C 2.74379 -0.02888 0.49077

C 3.53975 -0.62155 1.65248

H 3.60040 0.06096 2.50665

H 4.56781 -0.80083 1.31115

H 3.14515 -1.58495 1.99287

C 3.33674 1.32307 0.07818

H 2.77747 1.79399 -0.73779

H 4.36165 1.15474 -0.27664

H 3.39078 2.02947 0.91102

C 2.76154 -0.98153 -0.71116

H 2.39005 -1.98070 -0.45767

H 3.79773 -1.08778 -1.05657

H 2.17886 -0.58969 -1.55434

**2^O^+NO_singlet_UKS**

Au -0.19466 1.07271 -1.02016

P 0.94198 0.20329 0.86552

O 1.46670 3.11050 -2.77933

O 0.75563 2.73431 -1.05698

N -1.17141 -0.70200 -1.02168

N 1.40829 2.35624 -3.58675

C -2.06266 -0.87003 -2.00621

C -2.76216 -2.09616 -2.10609

H -3.48068 -2.24227 -2.90724

C -2.53049 -3.08508 -1.18542

H -3.06795 -4.03043 -1.24938

C -1.32281 -3.87324 0.84681

H -1.85676 -4.82165 0.81024

C -0.39938 -3.62520 1.83746

H -0.19509 -4.37600 2.59796

C 0.29419 -2.39803 1.87207

H 1.02288 -2.23894 2.66321

C 0.06800 -1.40938 0.92792

C -0.89918 -1.65640 -0.08726

C -1.59266 -2.89572 -0.13734

C -2.24515 0.28941 -2.87548

C -1.45092 1.42501 -2.58374

C -1.66883 2.60969 -3.27377

H -1.11807 3.50826 -2.99932

C -2.62582 2.65886 -4.29282

H -2.79105 3.58976 -4.83356

C -3.37521 1.52728 -4.61614

H -4.11508 1.57171 -5.41280

C -3.19754 0.34713 -3.90130

H -3.81555 -0.51697 -4.13889

C 0.53816 1.16596 2.42252

C 1.06730 2.59484 2.24067

H 0.59758 3.23692 2.99676

H 0.83600 3.00337 1.24725

H 2.14908 2.65503 2.39104

C 1.09146 0.54328 3.70255

H 2.18541 0.51204 3.71406

H 0.70323 -0.46473 3.88425

H 0.77960 1.16362 4.55312

C -0.99504 1.19435 2.47518

H -1.42599 1.68868 1.59523

H -1.30363 1.77193 3.35581

H -1.43088 0.19229 2.56603

C 2.74253 -0.15888 0.48569

C 3.42213 -1.07448 1.50274

H 3.42499 -0.65763 2.51430

H 4.47082 -1.20597 1.20469

H 2.97052 -2.07203 1.52967

C 3.47583 1.18294 0.37957

H 2.90466 1.91764 -0.20331

H 4.44165 1.01688 -0.11508

H 3.68712 1.61089 1.36448

C 2.72561 -0.84793 -0.88523

H 2.15421 -1.78437 -0.87525

H 3.75673 -1.09476 -1.16941

H 2.31309 -0.19755 -1.66637

**2^O^+NO_singlet_BS**

Au -0.28790 1.14831 -0.93119

P 0.92019 0.22470 0.89032

O 1.58719 2.34580 -4.08484

O 0.51277 2.86370 -0.93303

N -1.20901 -0.64502 -0.99289

N 1.93380 1.60723 -3.28930

C -2.14068 -0.78619 -1.94660

C -2.84245 -2.01045 -2.05457

H -3.59918 -2.12750 -2.82466

C -2.56166 -3.03279 -1.18695

H -3.09626 -3.97886 -1.26240

C -1.24197 -3.89595 0.74161

H -1.76358 -4.85014 0.68415

C -0.27153 -3.68317 1.69541

H -0.01493 -4.46902 2.40271

C 0.39603 -2.44416 1.76402

H 1.15719 -2.30828 2.52842

C 0.10086 -1.41326 0.88613

C -0.89575 -1.63153 -0.10377

C -1.57463 -2.87727 -0.17889

C -2.33544 0.38183 -2.79453

C -1.53594 1.50858 -2.49597

C -1.65075 2.66879 -3.24569

H -1.03669 3.53822 -3.01613

C -2.56506 2.71350 -4.30308

H -2.65819 3.62316 -4.89453

C -3.35737 1.60505 -4.60753

H -4.06403 1.65177 -5.43364

C -3.24734 0.43951 -3.85734

H -3.87142 -0.41698 -4.10617

C 0.48364 1.07656 2.50248

C 1.17880 2.44074 2.51753

H 0.77021 3.03372 3.34569

H 1.00812 3.00517 1.59148

H 2.25720 2.35072 2.68453

C 0.85087 0.26248 3.74373

H 1.91730 0.02659 3.80463

H 0.27477 -0.66633 3.81065

H 0.60039 0.85789 4.63139

C -1.03809 1.26716 2.45501

H -1.34736 1.92285 1.63221

H -1.36178 1.74003 3.39110

H -1.57201 0.31279 2.36711

C 2.73583 -0.02868 0.48394

C 3.53160 -0.64004 1.63634

H 3.59539 0.03045 2.49973

H 4.55872 -0.81838 1.29183

H 3.13389 -1.60667 1.96409

C 3.33545 1.32506 0.08655

H 2.77021 1.81509 -0.71404

H 4.35475 1.15485 -0.28294

H 3.40502 2.01608 0.93110

C 2.74823 -0.96649 -0.72970

H 2.36879 -1.96593 -0.48939

H 3.78418 -1.07653 -1.07457

H 2.17034 -0.55979 -1.56904

**2^O^_free_oxo**

Au 2.30014 9.76262 5.44044

P 2.31102 7.50094 4.72532

N 3.57768 10.06525 3.90923

O 1.12797 9.55693 6.90744

C 3.99146 11.32890 3.73824

C 4.90782 11.62346 2.70061

H 5.24240 12.64657 2.55682

C 5.36780 10.61576 1.89439

H 6.07768 10.83187 1.09698

C 5.38318 8.19717 1.29627

H 6.09734 8.38926 0.49692

C 4.92746 6.92025 1.53834

H 5.27841 6.08801 0.93186

C 4.00033 6.68213 2.57240

H 3.65551 5.66398 2.73498

C 3.52702 7.71050 3.37186

C 4.00469 9.02808 3.13248

C 4.93313 9.27825 2.08654

C 3.43428 12.29074 4.67959

C 2.55317 11.76857 5.65405

C 1.96869 12.60569 6.59177

H 1.28939 12.20382 7.34214

C 2.25965 13.97367 6.56482

H 1.80133 14.63466 7.29907

C 3.13062 14.49836 5.60806

H 3.34953 15.56416 5.59791

C 3.71951 13.66289 4.66528

H 4.39500 14.08599 3.92388

C 0.67922 7.03261 3.92620

C -0.35240 6.81277 5.03522

H -1.34840 6.74839 4.57871

H -0.37020 7.64021 5.75620

H -0.18234 5.87547 5.57510

C 0.78408 5.80233 3.02452

H 1.43257 5.98040 2.15998

H -0.21606 5.57531 2.63280

H 1.13396 4.91082 3.55362

C 0.28205 8.24921 3.07960

H 0.09777 9.13786 3.69508

H -0.65038 8.01916 2.54817

H 1.03809 8.49344 2.32293

C 3.02573 6.34320 6.01941

C 3.07219 4.88278 5.57122

H 2.07318 4.46856 5.40093

H 3.53434 4.28907 6.37071

H 3.67932 4.73255 4.67220

C 2.18878 6.47607 7.29740

H 2.07797 7.51775 7.62298

H 2.69860 5.92753 8.09976

H 1.18990 6.04355 7.19309

C 4.44202 6.86713 6.28647

H 5.09490 6.77805 5.41099

H 4.88702 6.27537 7.09637

H 4.43624 7.91423 6.61589

**2^ONO^_triplet**

Au -0.41293 1.01630 -0.92020

P 0.81056 0.15280 0.91495

O 1.58617 4.27713 -1.66505

O 0.35756 2.83366 -0.76984

N -1.28011 -0.76047 -0.95783

N 1.06795 3.24104 -1.85243

C -2.23553 -0.95638 -1.98497

C -2.89498 -2.20404 -2.06340

H -3.62122 -2.36335 -2.85630

C -2.63643 -3.19478 -1.15936

H -3.14490 -4.15397 -1.21951

C -1.38244 -3.93783 0.86100

H -1.89027 -4.89997 0.82164

C -0.44374 -3.68565 1.86498

H -0.22224 -4.45265 2.60456

C 0.22043 -2.45440 1.92523

H 0.95479 -2.29078 2.70927

C -0.04901 -1.45796 0.99314

C -1.00705 -1.70969 -0.03630

C -1.68284 -2.97679 -0.10202

C -2.42335 0.16581 -2.81263

C -1.62814 1.34839 -2.50678

C -1.77134 2.49376 -3.26070

H -1.18097 3.38018 -3.03925

C -2.67649 2.51578 -4.33219

H -2.78767 3.41812 -4.93079

C -3.45384 1.36423 -4.64747

H -4.14914 1.40512 -5.48400

C -3.33829 0.21471 -3.91223

H -3.94067 -0.65516 -4.16342

C 0.48963 1.10564 2.49752

C 1.33731 2.38163 2.46185

H 1.02811 3.02554 3.29535

H 1.19324 2.94773 1.53347

H 2.40456 2.17331 2.59452

C 0.77233 0.29488 3.76254

H 1.80092 -0.07429 3.81776

H 0.08271 -0.54887 3.87098

H 0.61716 0.94940 4.63024

C -1.00009 1.46924 2.44850

H -1.22830 2.15462 1.62349

H -1.26919 1.98068 3.38171

H -1.64211 0.58283 2.36534

C 2.60296 -0.20545 0.46739

C 3.42484 -0.66101 1.67212

H 3.53931 0.12770 2.42286

H 4.43273 -0.92578 1.32611

H 3.00882 -1.55243 2.15470

C 3.20964 1.05836 -0.15327

H 2.71211 1.33008 -1.09021

H 4.26240 0.85558 -0.38830

H 3.18255 1.92659 0.51095

C 2.56485 -1.30899 -0.59603

H 2.23397 -2.27085 -0.19033

H 3.57827 -1.44428 -0.99460

H 1.91738 -1.04500 -1.44277

**2^ONO^_singlet**

Au -0.41248 1.01843 -0.93342

P 0.81841 0.14985 0.90541

O 1.56700 4.28730 -1.68172

O 0.37596 2.82113 -0.77449

N -1.30413 -0.76802 -0.97360

N 1.02766 3.26550 -1.88258

C -2.22022 -0.94044 -1.93964

C -2.91251 -2.17141 -2.02103

H -3.65132 -2.31810 -2.80321

C -2.64936 -3.16040 -1.11041

H -3.17897 -4.11072 -1.16535

C -1.38906 -3.94863 0.88601

H -1.91186 -4.90315 0.84878

C -0.44672 -3.70173 1.85956

H -0.21286 -4.46057 2.60328

C 0.22380 -2.46360 1.89731

H 0.96923 -2.30228 2.67220

C -0.04306 -1.46453 0.97412

C -1.01195 -1.71775 -0.03432

C -1.69118 -2.96517 -0.08188

C -2.41479 0.20342 -2.81686

C -1.63406 1.34812 -2.52900

C -1.76657 2.48985 -3.30180

H -1.16974 3.37540 -3.09558

C -2.67336 2.49820 -4.36834

H -2.77646 3.39563 -4.97654

C -3.44359 1.37187 -4.66036

H -4.14383 1.39114 -5.49297

C -3.31824 0.22254 -3.88789

H -3.92421 -0.64997 -4.12542

C 0.48446 1.08793 2.49355

C 1.32472 2.36949 2.47031

H 1.00554 3.00875 3.30357

H 1.18547 2.93799 1.54260

H 2.39204 2.16669 2.61070

C 0.76457 0.27246 3.75617

H 1.79459 -0.09226 3.81470

H 0.07838 -0.57513 3.85747

H 0.60200 0.92128 4.62679

C -1.00676 1.44443 2.43904

H -1.23537 2.12977 1.61385

H -1.28287 1.95364 3.37141

H -1.64443 0.55524 2.35208

C 2.61412 -0.19920 0.47063

C 3.43271 -0.66115 1.67535

H 3.53677 0.12097 2.43445

H 4.44472 -0.91533 1.33358

H 3.02044 -1.55985 2.14770

C 3.22014 1.07264 -0.13560

H 2.72340 1.35441 -1.06984

H 4.27359 0.87455 -0.37121

H 3.18977 1.93332 0.53800

C 2.58884 -1.29207 -0.60411

H 2.25219 -2.25827 -0.21358

H 3.60693 -1.42416 -0.99168

H 1.95254 -1.01808 -1.45617

**2^NOO^_triplet**

Au 8.26400 3.42507 6.35612

P 7.65211 1.22986 5.62681

N 6.39358 3.93979 5.71355

N 10.13126 3.13173 6.92455

O 10.18633 2.36919 8.06673

O 9.80364 3.00761 9.12748

C 5.98522 1.67246 4.99581

C 5.11950 0.78225 4.37886

H 5.41002 -0.25618 4.24590

C 3.85092 1.18348 3.91558

H 3.20162 0.45322 3.43725

C 3.43523 2.48688 4.07215

H 2.45370 2.80316 3.72225

C 4.28298 3.43450 4.68692

C 5.56551 3.02295 5.14148

C 3.92340 4.79464 4.87344

H 2.94365 5.13249 4.53780

C 4.79138 5.67607 5.46343

H 4.51432 6.71651 5.60444

C 6.06406 5.22562 5.88863

C 7.09017 6.04893 6.51761

C 8.30188 5.40121 6.85418

C 9.32849 6.10931 7.45791

H 10.26072 5.61259 7.72071

C 9.15592 7.46991 7.73471

H 9.96051 8.02657 8.21292

C 7.96468 8.11750 7.40613

H 7.84108 9.17587 7.62636

C 6.93149 7.41274 6.79706

H 6.00792 7.93098 6.54538

C 8.67738 0.64342 4.16630

C 8.47715 1.70333 3.07671

H 7.44458 1.73941 2.71223

H 9.12403 1.46020 2.22427

H 8.76061 2.70661 3.42155

C 10.15123 0.62680 4.58823

H 10.48262 1.59215 4.98871

H 10.76225 0.41110 3.70242

H 10.37080 -0.14533 5.33066

C 8.25998 -0.73369 3.65165

H 8.38480 -1.51842 4.40520

H 8.90202 -0.99867 2.80133

H 7.22797 -0.75162 3.28611

C 7.39200 0.01239 7.04018

C 6.86585 0.86165 8.20497

H 7.61123 1.58018 8.56574

H 6.62217 0.19821 9.04484

H 5.94813 1.40384 7.94271

C 6.37174 -1.07675 6.70584

H 5.36627 -0.66966 6.55898

H 6.31929 -1.76656 7.55813

H 6.64907 -1.67113 5.82845

C 8.73004 -0.62616 7.42176

H 9.08424 -1.33022 6.66132

H 8.58548 -1.19734 8.34782

H 9.51300 0.11348 7.61650

**2^NOO^_singlet_bs**

Au 8.27834 3.37941 6.34385

P 7.63643 1.19462 5.61162

N 6.39487 3.90114 5.76923

N 10.19652 3.09453 6.76634

O 10.39245 2.71880 7.91892

O 9.39424 2.51377 8.73026

C 5.99070 1.66785 4.95219

C 5.13223 0.80329 4.29154

H 5.43286 -0.22381 4.10312

C 3.85827 1.21753 3.85447

H 3.21331 0.50853 3.33965

C 3.43075 2.50573 4.08581

H 2.44360 2.83053 3.76048

C 4.27435 3.42821 4.74359

C 5.56424 3.00587 5.16526

C 3.90482 4.77354 5.00096

H 2.91753 5.11841 4.69593

C 4.77564 5.63420 5.61653

H 4.49385 6.66559 5.80608

C 6.05996 5.17759 5.99667

C 7.09953 5.99032 6.61582

C 8.33381 5.34884 6.87175

C 9.38057 6.05734 7.44096

H 10.34031 5.58045 7.63152

C 9.20471 7.40596 7.76877

H 10.02733 7.96019 8.21812

C 7.98867 8.04412 7.52541

H 7.86133 9.09289 7.78565

C 6.93674 7.34193 6.94700

H 5.99395 7.85279 6.75998

C 8.69206 0.58997 4.17751

C 8.51065 1.63411 3.06961

H 7.49012 1.64901 2.67158

H 9.18928 1.39209 2.24185

H 8.76562 2.64601 3.41147

C 10.15700 0.57939 4.62782

H 10.49898 1.56325 4.96777

H 10.77979 0.29348 3.77049

H 10.35042 -0.14435 5.42545

C 8.29887 -0.79718 3.67030

H 8.46586 -1.57726 4.42009

H 8.93047 -1.04161 2.80619

H 7.26042 -0.84852 3.32780

C 7.28539 -0.01085 7.01462

C 6.54919 0.81447 8.07741

H 7.17205 1.62745 8.46667

H 6.30043 0.15643 8.91996

H 5.60580 1.22715 7.69801

C 6.39920 -1.18191 6.58443

H 5.39711 -0.85651 6.28740

H 6.27443 -1.84347 7.45133

H 6.83422 -1.78355 5.78104

C 8.60958 -0.52955 7.57761

H 9.14776 -1.16284 6.86446

H 8.38959 -1.14875 8.45677

H 9.25901 0.28301 7.91928

**2^NOO^_singlet**

Au 8.27832 3.37922 6.34378

P 7.63651 1.19449 5.61153

N 6.39473 3.90093 5.76951

N 10.19658 3.09420 6.76603

O 10.39260 2.71890 7.91870

O 9.39447 2.51422 8.73022

C 5.99038 1.66747 4.95290

C 5.13163 0.80268 4.29294

H 5.43209 -0.22453 4.10488

C 3.85752 1.21682 3.85619

H 3.21234 0.50764 3.34189

C 3.43016 2.50515 4.08715

H 2.44295 2.82991 3.76199

C 4.27400 3.42782 4.74439

C 5.56398 3.00555 5.16585

C 3.90456 4.77324 5.00148

H 2.91725 5.11807 4.69651

C 4.77551 5.63401 5.61672

H 4.49380 6.66546 5.80606

C 6.05985 5.17741 5.99683

C 7.09949 5.99019 6.61585

C 8.33379 5.34871 6.87173

C 9.38055 6.05722 7.44095

H 10.34026 5.58028 7.63150

C 9.20469 7.40583 7.76876

H 10.02732 7.96007 8.21809

C 7.98865 8.04400 7.52541

H 7.86130 9.09276 7.78568

C 6.93670 7.34180 6.94703

H 5.99392 7.85269 6.76005

C 8.69156 0.59055 4.17670

C 8.51026 1.63592 3.06993

H 7.48925 1.65268 2.67319

H 9.18746 1.39359 2.24109

H 8.76714 2.64715 3.41234

C 10.15661 0.57889 4.62664

H 10.49866 1.56185 4.96912

H 10.77919 0.29550 3.76832

H 10.35030 -0.14706 5.42217

C 8.29760 -0.79584 3.66811

H 8.46353 -1.57672 4.41730

H 8.92958 -1.03998 2.80419

H 7.25933 -0.84599 3.32486

C 7.28634 -0.01113 7.01462

C 6.55038 0.81387 8.07781

H 7.17314 1.62699 8.46691

H 6.30209 0.15568 8.92038

H 5.60673 1.22632 7.69880

C 6.40014 -1.18236 6.58474

H 5.39751 -0.85713 6.28938

H 6.27699 -1.84471 7.45126

H 6.83422 -1.78314 5.78019

C 8.61090 -0.52950 7.57711

H 9.14909 -1.16246 6.86371

H 8.39134 -1.14896 8.45619

H 9.25997 0.28336 7.91875

**2_UKS_BS**

Au 2.22460 9.77923 5.43055

P 2.28139 7.51099 4.71266

N 3.58960 10.06564 3.91240

N 0.95800 9.60743 6.84028

C 4.01867 11.32279 3.76323

C 4.95680 11.61608 2.74363

H 5.31046 12.63438 2.61184

C 5.41022 10.60863 1.93151

H 6.13317 10.82311 1.14534

C 5.38705 8.19491 1.30070

H 6.11163 8.37988 0.50901

C 4.90250 6.92550 1.52610

H 5.24130 6.09254 0.91366

C 3.96274 6.69329 2.55113

H 3.60155 5.67892 2.70033

C 3.50019 7.72148 3.35689

C 4.00667 9.03249 3.13259

C 4.95113 9.27560 2.09943

C 3.44902 12.28571 4.70342

C 2.51977 11.78468 5.64781

C 1.93623 12.64091 6.57005

H 1.22128 12.25853 7.29761

C 2.26991 13.99956 6.56229

H 1.81042 14.67063 7.28655

C 3.18545 14.49990 5.63624

H 3.44012 15.55782 5.63771

C 3.77523 13.64809 4.70768

H 4.48764 14.05311 3.99119

C 0.65746 6.99709 3.92560

C -0.36064 6.75825 5.04335

H -1.35866 6.66916 4.59550

H -0.39317 7.58668 5.76270

H -0.16518 5.82753 5.58638

C 0.77657 5.76485 3.02857

H 1.40802 5.95258 2.15382

H -0.22403 5.51427 2.65272

H 1.15285 4.88390 3.55730

C 0.22926 8.20125 3.07627

H 0.03315 9.08997 3.68833

H -0.70265 7.95173 2.55280

H 0.97587 8.45635 2.31386

C 3.03391 6.37249 6.00486

C 3.09469 4.91030 5.56453

H 2.09894 4.47790 5.42258

H 3.58828 4.32913 6.35444

H 3.68036 4.76601 4.65052

C 2.21879 6.49739 7.29689

H 2.12328 7.53586 7.63533

H 2.73571 5.93670 8.08620

H 1.21301 6.07820 7.20526

C 4.44729 6.91704 6.24538

H 5.09079 6.81585 5.36448

H 4.90830 6.34836 7.06288

H 4.43537 7.97158 6.55085

**2_UKS**

Au 2.25613 9.73992 5.48763

P 2.30355 7.49868 4.71640

N 3.57963 10.05441 3.91042

N 1.12591 9.40249 6.86618

C 4.00423 11.30977 3.74197

C 4.92869 11.60618 2.71005

H 5.27268 12.62699 2.57229

C 5.38311 10.60062 1.89844

H 6.09661 10.81472 1.10386

C 5.36721 8.18283 1.28712

H 6.08295 8.36786 0.48742

C 4.89243 6.91113 1.52180

H 5.22906 6.07762 0.90900

C 3.96509 6.67856 2.55680

H 3.60649 5.66431 2.71182

C 3.51182 7.71160 3.36237

C 4.00279 9.02489 3.12834

C 4.93355 9.26626 2.08307

C 3.44544 12.27057 4.68352

C 2.55134 11.76223 5.65243

C 1.97172 12.61567 6.58387

H 1.28238 12.23377 7.33386

C 2.27967 13.97814 6.55443

H 1.82309 14.64495 7.28444

C 3.16402 14.48879 5.60304

H 3.39804 15.55129 5.58990

C 3.74613 13.63999 4.66990

H 4.43300 14.04903 3.93138

C 0.65594 7.04505 3.93800

C -0.37880 6.85414 5.04750

H -1.37385 6.80253 4.58746

H -0.38698 7.69336 5.75674

H -0.22683 5.92097 5.59861

C 0.75488 5.79527 3.06035

H 1.40567 5.95059 2.19348

H -0.24712 5.57173 2.67212

H 1.09319 4.91049 3.60802

C 0.27248 8.24894 3.06775

H 0.08918 9.14733 3.66821

H -0.65792 8.01118 2.53632

H 1.03344 8.47658 2.31106

C 3.03763 6.36136 6.02156

C 3.19533 4.92179 5.52777

H 2.23522 4.45052 5.29529

H 3.65497 4.33360 6.33239

H 3.85617 4.84088 4.65855

C 2.15384 6.39826 7.27055

H 1.93691 7.42524 7.60076

H 2.69307 5.90074 8.08673

H 1.20825 5.86683 7.13351

C 4.41013 6.96357 6.34600

H 5.08230 6.96726 5.48018

H 4.87928 6.35437 7.12882

H 4.32719 7.98643 6.73377

**2_triplet**

Au 2.21651 9.78627 5.42065

P 2.27839 7.51484 4.71062

N 3.59177 10.07014 3.91487

N 0.91564 9.62965 6.83325

C 4.02145 11.32763 3.77011

C 4.96408 11.62112 2.75479

H 5.31934 12.63918 2.62542

C 5.41936 10.61399 1.94299

H 6.14569 10.82900 1.16006

C 5.39623 8.20117 1.30773

H 6.12413 8.38648 0.51917

C 4.90988 6.93190 1.53011

H 5.25091 6.09925 0.91847

C 3.96577 6.69928 2.55117

H 3.60468 5.68463 2.69921

C 3.49935 7.72661 3.35563

C 4.00906 9.03722 3.13517

C 4.95815 9.28112 2.10629

C 3.44686 12.29002 4.70865

C 2.50872 11.78887 5.64503

C 1.92041 12.64487 6.56394

H 1.19826 12.26290 7.28474

C 2.25784 14.00296 6.56198

H 1.79472 14.67388 7.28408

C 3.18169 14.50274 5.64409

H 3.43914 15.55999 5.64979

C 3.77652 13.65136 4.71799

H 4.49552 14.05635 4.00804

C 0.65900 6.98768 3.92336

C -0.35540 6.74033 5.04307

H -1.35249 6.63523 4.59657

H -0.39872 7.57133 5.75856

H -0.14760 5.81521 5.59141

C 0.78335 5.75806 3.02370

H 1.41284 5.95122 2.14870

H -0.21629 5.50315 2.64808

H 1.16469 4.87775 3.54976

C 0.22059 8.19001 3.07654

H 0.02292 9.07785 3.68956

H -0.71229 7.93678 2.55661

H 0.96316 8.44921 2.31155

C 3.03439 6.37384 5.99897

C 3.04961 4.90480 5.57859

H 2.04069 4.49234 5.47562

H 3.55666 4.32364 6.36001

H 3.60006 4.73368 4.64727

C 2.24954 6.53678 7.30573

H 2.21569 7.57782 7.64635

H 2.75002 5.94820 8.08531

H 1.22104 6.17249 7.23206

C 4.46556 6.88497 6.20440

H 5.09245 6.73873 5.31815

H 4.92222 6.32911 7.03310

H 4.48858 7.94866 6.47620

**3_triplet**

Au 2.23406 9.81434 5.40189

P 2.25971 7.53021 4.72849

O -0.30021 9.53073 6.51149

O 1.20779 9.81888 8.00885

N 3.57167 10.06334 3.92356

N 0.84295 9.69966 6.86574

C 4.03916 11.38538 3.74762

C 4.97697 11.63732 2.71806

H 5.33778 12.65221 2.57288

C 5.42800 10.62821 1.91653

H 6.15091 10.82110 1.12750

C 5.38369 8.20252 1.34587

H 6.11548 8.37209 0.55785

C 4.89758 6.91340 1.58188

H 5.25372 6.08121 0.97783

C 3.95238 6.68110 2.58887

H 3.59228 5.66853 2.74488

C 3.47749 7.72649 3.37384

C 3.98713 9.04278 3.15132

C 4.95154 9.28252 2.11093

C 3.49804 12.31343 4.65585

C 2.55459 11.80717 5.64619

C 2.02928 12.66022 6.59539

H 1.34836 12.29127 7.35965

C 2.38099 14.01889 6.59174

H 1.96016 14.68913 7.33913

C 3.28413 14.52989 5.61707

H 3.54123 15.58746 5.63143

C 3.83330 13.70658 4.67144

H 4.52809 14.10905 3.93815

C 0.64194 6.96509 3.94833

C -0.32540 6.51734 5.04683

H -1.30196 6.32616 4.58339

H -0.47823 7.28436 5.81306

H -0.00785 5.58405 5.52357

C 0.83934 5.84093 2.93052

H 1.41926 6.16840 2.06181

H -0.15033 5.54044 2.56264

H 1.30702 4.94815 3.35873

C 0.09080 8.20694 3.23566

H -0.18713 8.99335 3.94670

H -0.81672 7.92499 2.68621

H 0.80030 8.61366 2.50303

C 3.03042 6.39827 6.01794

C 3.02779 4.92997 5.59297

H 2.01554 4.53133 5.47104

H 3.51429 4.33978 6.38057

H 3.59216 4.75406 4.67121

C 2.26207 6.57020 7.33326

H 2.31000 7.59607 7.71302

H 2.72524 5.92282 8.08897

H 1.21070 6.27848 7.25773

C 4.46746 6.89723 6.20670

H 5.08614 6.73494 5.31757

H 4.92462 6.34599 7.03824

H 4.50270 7.96319 6.46798

**3_singlet**

Au 2.23028 9.82471 5.40180

P 2.25944 7.53277 4.72985

O -0.30996 9.58030 6.49029

O 1.19919 9.79672 8.00022

N 3.59156 10.08423 3.91862

N 0.83359 9.71438 6.85555

C 4.03381 11.33874 3.76067

C 4.97264 11.61340 2.73785

H 5.33369 12.62800 2.59905

C 5.41726 10.59621 1.93434

H 6.14263 10.79772 1.14700

C 5.38007 8.18208 1.32276

H 6.10901 8.35939 0.53341

C 4.88940 6.91666 1.55542

H 5.22765 6.07679 0.95226

C 3.94279 6.69921 2.57601

H 3.57588 5.68837 2.73110

C 3.47727 7.73534 3.37096

C 3.99532 9.03873 3.14365

C 4.94615 9.26954 2.11250

C 3.48528 12.31397 4.69468

C 2.55223 11.82814 5.64218

C 2.00941 12.69117 6.58269

H 1.30919 12.33312 7.33449

C 2.37767 14.04148 6.57691

H 1.94709 14.71652 7.31505

C 3.28958 14.52770 5.64042

H 3.56969 15.57908 5.64583

C 3.84774 13.66723 4.70127

H 4.56591 14.05669 3.98208

C 0.64430 6.96348 3.95080

C -0.32245 6.52010 5.05203

H -1.30150 6.33318 4.59229

H -0.46982 7.28731 5.81942

H -0.00712 5.58559 5.52790

C 0.83550 5.83629 2.93490

H 1.40859 6.16192 2.06089

H -0.15600 5.53362 2.57379

H 1.30697 4.94506 3.36222

C 0.09229 8.20410 3.23635

H -0.17915 8.99478 3.94548

H -0.81972 7.92345 2.69372

H 0.79825 8.60572 2.49752

C 3.03709 6.40187 6.01468

C 3.05493 4.93608 5.58162

H 2.04797 4.52744 5.44896

H 3.54143 4.34578 6.36911

H 3.62852 4.77242 4.66317

C 2.26233 6.55588 7.32868

H 2.28380 7.58267 7.70861

H 2.73943 5.92046 8.08582

H 1.21867 6.23845 7.25122

C 4.46663 6.91801 6.21451

H 5.09092 6.77745 5.32550

H 4.92932 6.36151 7.03944

H 4.48581 7.98029 6.49203

**ts_1-2_triplet**

Au 8.22151 3.33615 6.40519

P 7.58884 1.17034 5.62648

N 6.38588 3.90062 5.71321

N 10.03027 2.93031 7.03531

N 9.91326 2.56473 8.57672

N 10.38053 3.25753 9.36211

C 5.93125 1.64249 4.99413

C 5.04824 0.76870 4.37893

H 5.31824 -0.27566 4.24767

C 3.78886 1.19659 3.91296

H 3.12357 0.47906 3.43730

C 3.40291 2.51000 4.06159

H 2.42954 2.84647 3.70779

C 4.27049 3.44172 4.67382

C 5.54079 3.00266 5.13548

C 3.94244 4.81082 4.85325

H 2.97289 5.17075 4.51105

C 4.82735 5.67349 5.44648

H 4.57330 6.72036 5.58317

C 6.08455 5.19352 5.88509

C 7.12319 5.99120 6.52880

C 8.30847 5.31058 6.89772

C 9.34584 5.99979 7.50686

H 10.26312 5.48665 7.79122

C 9.20979 7.36881 7.76190

H 10.02420 7.90793 8.24349

C 8.04397 8.04605 7.40451

H 7.94843 9.11089 7.60668

C 7.00118 7.36268 6.78733

H 6.09863 7.90449 6.51046

C 8.62157 0.62105 4.15561

C 8.43497 1.71799 3.10040

H 7.40410 1.77553 2.73363

H 9.08234 1.49532 2.24278

H 8.72765 2.70662 3.47792

C 10.09298 0.57706 4.58380

H 10.42680 1.51622 5.04007

H 10.70707 0.40306 3.69094

H 10.30316 -0.23407 5.28634

C 8.19586 -0.73389 3.59210

H 8.33482 -1.54701 4.31225

H 8.82550 -0.96366 2.72243

H 7.15800 -0.73974 3.24245

C 7.31939 -0.07124 7.01046

C 6.68642 0.73682 8.15043

H 7.36752 1.50288 8.53966

H 6.45293 0.05572 8.97892

H 5.74684 1.21520 7.84595

C 6.38352 -1.21870 6.62621

H 5.36037 -0.87395 6.44496

H 6.34039 -1.91968 7.46988

H 6.72917 -1.78439 5.75503

C 8.67781 -0.62082 7.45274

H 9.10800 -1.30467 6.71354

H 8.53429 -1.19352 8.37799

H 9.40222 0.17179 7.67122

**ts_1-2_singlet_BS**

Au 8.22794 3.41696 6.39024

P 7.60646 1.21230 5.66820

N 6.35077 3.92245 5.73621

N 10.09105 3.22098 6.92253

N 10.21080 2.13352 8.31563

N 10.73089 2.14073 9.29816

C 5.95048 1.66052 5.00574

C 5.09779 0.77639 4.36252

H 5.39074 -0.26058 4.22634

C 3.83895 1.18111 3.87558

H 3.20021 0.45418 3.37827

C 3.42017 2.48330 4.03179

H 2.44662 2.80272 3.66294

C 4.25688 3.42658 4.66798

C 5.52888 3.01087 5.14835

C 3.89872 4.78803 4.84552

H 2.92631 5.12900 4.49219

C 4.76320 5.66793 5.44285

H 4.49143 6.71156 5.57002

C 6.02738 5.21258 5.88771

C 7.05635 6.04210 6.50622

C 8.27061 5.39914 6.84255

C 9.30110 6.11462 7.43145

H 10.23317 5.61326 7.69019

C 9.13434 7.47975 7.68736

H 9.94443 8.04406 8.14705

C 7.93993 8.12263 7.36023

H 7.81819 9.18418 7.56595

C 6.90129 7.40948 6.77016

H 5.97534 7.92468 6.52090

C 8.65488 0.64413 4.21572

C 8.50935 1.74566 3.15866

H 7.48404 1.83126 2.78155

H 9.15890 1.50524 2.30742

H 8.82520 2.72569 3.53952

C 10.11599 0.57856 4.67727

H 10.43813 1.50200 5.17420

H 10.75104 0.43310 3.79401

H 10.30432 -0.25981 5.35353

C 8.21944 -0.70202 3.63924

H 8.28871 -1.51347 4.37146

H 8.89038 -0.95990 2.80922

H 7.20434 -0.67711 3.22936

C 7.30056 -0.04801 7.03704

C 6.77583 0.77403 8.22092

H 7.52050 1.48829 8.59017

H 6.52905 0.09494 9.04728

H 5.86113 1.32523 7.96700

C 6.26224 -1.10773 6.66512

H 5.26605 -0.67838 6.52004

H 6.18919 -1.81893 7.49826

H 6.53683 -1.68401 5.77487

C 8.61712 -0.73368 7.41020

H 8.95013 -1.43314 6.63620

H 8.45839 -1.31572 8.32722

H 9.42305 -0.02159 7.60808

**ts_1-2_singlet**

Au 8.27976 3.28826 6.30189

P 7.44172 1.11142 5.79759

N 6.45087 3.92058 5.55318

N 9.82705 2.46460 6.88503

N 11.50496 3.52786 7.58484

N 12.52541 3.30541 7.92866

C 5.83235 1.63428 5.10295

C 4.88297 0.74959 4.61389

H 5.07614 -0.31961 4.62152

C 3.65285 1.20250 4.09834

H 2.93196 0.47923 3.72315

C 3.36716 2.54925 4.07031

H 2.41868 2.90723 3.67254

C 4.30297 3.48797 4.55786

C 5.54186 3.02541 5.07794

C 4.07012 4.88832 4.55560

H 3.12890 5.26839 4.16066

C 5.01577 5.75099 5.04178

H 4.84150 6.82277 5.03993

C 6.23392 5.23841 5.55146

C 7.32288 6.03048 6.10120

C 8.46004 5.31935 6.55333

C 9.52463 6.01773 7.10472

H 10.40415 5.49437 7.46445

C 9.47332 7.41134 7.20246

H 10.31604 7.94745 7.63647

C 8.35791 8.11660 6.75187

H 8.32644 9.20130 6.83085

C 7.28386 7.42817 6.20282

H 6.41481 7.98406 5.85616

C 8.42532 0.29472 4.41943

C 8.62806 1.39548 3.37031

H 7.67690 1.79179 2.99385

H 9.16796 0.96652 2.51644

H 9.22988 2.22512 3.75882

C 9.78000 -0.14612 4.97702

H 10.27320 0.65724 5.54201

H 10.43218 -0.40835 4.13415

H 9.69656 -1.03223 5.61334

C 7.69557 -0.88900 3.78175

H 7.46401 -1.68484 4.49631

H 8.35631 -1.32266 3.02014

H 6.77670 -0.58527 3.27035

C 7.09114 0.13970 7.36923

C 6.26012 1.08846 8.24270

H 6.81714 1.99508 8.50853

H 6.01213 0.57041 9.17784

H 5.31641 1.37948 7.76620

C 6.30649 -1.14655 7.10764

H 5.32162 -0.95911 6.66792

H 6.13455 -1.64230 8.07184

H 6.85505 -1.85428 6.47821

C 8.41436 -0.17666 8.07049

H 8.97115 -0.97233 7.56762

H 8.19010 -0.52602 9.08644

H 9.06133 0.70762 8.15236

**ts_2^NO2^-3_singlet**

Au -0.50040 1.14022 0.82946

P -0.43949 -1.15366 0.13134

O -2.75896 1.82738 2.50107

O -1.57343 1.22851 3.50133

N 0.86843 1.40181 -0.67987

N -1.92624 0.82856 2.24234

C 1.32425 2.65103 -0.82078

C 2.25196 2.93468 -1.85270

H 2.62444 3.94689 -1.97789

C 2.66842 1.92899 -2.68474

H 3.38208 2.13721 -3.48099

C 2.58800 -0.47209 -3.34341

H 3.30273 -0.28745 -4.14409

C 2.08910 -1.73713 -3.12512

H 2.40652 -2.56825 -3.75132

C 1.16519 -1.96504 -2.08619

H 0.79725 -2.97650 -1.93409

C 0.72711 -0.93847 -1.26426

C 1.24926 0.36683 -1.47905

C 2.18310 0.60486 -2.52432

C 0.80057 3.61657 0.13842

C -0.14215 3.13549 1.08038

C -0.65546 3.99819 2.03554

H -1.37471 3.64243 2.76979

C -0.24893 5.33699 2.05830

H -0.65775 6.00782 2.81251

C 0.67176 5.81726 1.12765

H 0.98169 6.86001 1.15220

C 1.19869 4.95967 0.16834

H 1.92245 5.34333 -0.54828

C -2.07669 -1.74489 -0.57350

C -3.00324 -2.09706 0.59444

H -4.01447 -2.26250 0.20091

H -3.06089 -1.29159 1.33598

H -2.69859 -3.02039 1.09895

C -1.94852 -2.92896 -1.53178

H -1.47595 -3.80387 -1.07439

H -1.40325 -2.66383 -2.44364

H -2.95796 -3.23197 -1.83983

C -2.63594 -0.53704 -1.33651

H -2.84213 0.31140 -0.67256

H -3.58570 -0.82102 -1.80774

H -1.96150 -0.20151 -2.13476

C 0.39752 -2.27080 1.39173

C 0.35243 -3.74947 1.00892

H -0.66863 -4.14409 0.98927

H 0.90625 -4.32435 1.76286

H 0.82727 -3.95133 0.04253

C -0.28308 -2.05214 2.74886

H -0.19155 -1.01510 3.09145

H 0.21474 -2.68566 3.49414

H -1.34373 -2.31777 2.74727

C 1.85002 -1.78900 1.48211

H 2.41721 -1.99334 0.56780

H 2.34484 -2.31561 2.30809

H 1.91607 -0.71421 1.69785

**ts_2^O^+NO-2^ONO^_triplet**

Au -0.44788 1.08565 0.94915

P -0.57107 -1.16152 0.19259

O -0.03386 0.38782 4.53934

O -1.52725 0.77734 2.53697

N 0.69008 1.39649 -0.67545

N -0.60561 1.19694 3.92510

C 1.13781 2.64995 -0.84374

C 1.96470 2.94391 -1.95396

H 2.32974 3.95685 -2.09509

C 2.29630 1.94986 -2.83626

H 2.93209 2.16753 -3.69355

C 2.12676 -0.43527 -3.53191

H 2.75917 -0.23737 -4.39612

C 1.62853 -1.69852 -3.30175

H 1.86108 -2.51343 -3.98397

C 0.81389 -1.94429 -2.17928

H 0.43306 -2.95071 -2.02448

C 0.49237 -0.93811 -1.28167

C 0.99583 0.36926 -1.52239

C 1.82051 0.62551 -2.65077

C 0.70321 3.60023 0.16893

C -0.11511 3.08002 1.19837

C -0.60695 3.91513 2.18839

H -1.24620 3.52083 2.97542

C -0.27212 5.27382 2.16789

H -0.65650 5.93219 2.94566

C 0.54743 5.79304 1.16420

H 0.80273 6.85076 1.16227

C 1.03541 4.96189 0.16205

H 1.66723 5.38034 -0.61939

C -2.29359 -1.61864 -0.39718

C -3.27539 -1.35029 0.75027

H -4.29562 -1.50691 0.37729

H -3.21198 -0.32132 1.12376

H -3.12940 -2.02469 1.59847

C -2.40445 -3.07029 -0.86143

H -2.24032 -3.78022 -0.04412

H -1.71678 -3.30692 -1.68078

H -3.42158 -3.24099 -1.23792

C -2.59829 -0.66232 -1.55638

H -2.49800 0.39012 -1.25988

H -3.63893 -0.81204 -1.87050

H -1.95764 -0.84136 -2.42678

C 0.29829 -2.34333 1.35986

C 0.71459 -3.65635 0.69582

H -0.12735 -4.20692 0.26482

H 1.16241 -4.30079 1.46361

H 1.47620 -3.50438 -0.07642

C -0.62507 -2.60705 2.55276

H -1.01204 -1.68026 2.99324

H -0.04861 -3.12489 3.32984

H -1.46788 -3.25481 2.28827

C 1.55158 -1.59396 1.83093

H 2.19509 -1.29017 0.99537

H 2.14119 -2.26454 2.46918

H 1.30447 -0.71209 2.43360

**ts_2^O^+NO-2^ONO^_singlet**

Au -0.38478 1.05781 -0.87897

P 0.82215 0.15787 0.95719

O 1.86796 3.59844 -3.54839

O 0.42157 2.76986 -0.92187

N -1.31766 -0.73069 -0.90596

N 1.80180 2.54371 -3.12324

C -2.25369 -0.88163 -1.85415

C -2.96644 -2.10147 -1.93693

H -3.72700 -2.22610 -2.70203

C -2.69071 -3.11028 -1.05209

H -3.23333 -4.05311 -1.10844

C -1.36923 -3.95066 0.88499

H -1.89900 -4.90131 0.84647

C -0.39240 -3.72953 1.83020

H -0.13856 -4.50513 2.54970

C 0.28526 -2.49484 1.87444

H 1.05074 -2.35253 2.63322

C -0.00549 -1.47636 0.98054

C -1.00854 -1.70384 -0.00099

C -1.69779 -2.94522 -0.05138

C -2.43989 0.27094 -2.72481

C -1.62582 1.39402 -2.45371

C -1.73083 2.53981 -3.22690

H -1.10308 3.40488 -3.02048

C -2.65130 2.57379 -4.27922

H -2.73762 3.47237 -4.88842

C -3.45827 1.46861 -4.55663

H -4.16941 1.50690 -5.37931

C -3.35698 0.31720 -3.78393

H -3.99203 -0.53697 -4.01202

C 0.40075 1.03126 2.56231

C 1.09200 2.39744 2.55413

H 0.68296 3.00233 3.37341

H 0.91901 2.94716 1.61974

H 2.17075 2.31326 2.72103

C 0.78042 0.23588 3.81203

H 1.84795 0.00320 3.86687

H 0.20694 -0.69293 3.89771

H 0.53665 0.84354 4.69324

C -1.12196 1.21723 2.52456

H -1.44002 1.85977 1.69469

H -1.43941 1.70303 3.45614

H -1.65380 0.26024 2.45512

C 2.63228 -0.11330 0.53558

C 3.42608 -0.75994 1.66994

H 3.50017 -0.11009 2.54798

H 4.44996 -0.94123 1.31740

H 3.01894 -1.72944 1.97639

C 3.25103 1.23683 0.15731

H 2.68511 1.75171 -0.62745

H 4.26316 1.05547 -0.22650

H 3.34339 1.90929 1.01451

C 2.62199 -1.02751 -0.69605

H 2.21104 -2.01975 -0.47793

H 3.65548 -1.16264 -1.03934

H 2.05952 -0.58734 -1.52972

**ts_2^ONO^-3_singlet**

Au -0.38293 1.01451 -0.99911

P 0.85528 0.18057 0.86008

O 1.69632 3.57556 -1.96417

O 0.03189 3.20509 -0.68559

N -1.29090 -0.75537 -0.99595

N 0.92822 2.79784 -1.51240

C -2.21737 -0.93940 -1.95290

C -2.90232 -2.17489 -2.01971

H -3.64613 -2.32775 -2.79611

C -2.62797 -3.15634 -1.10555

H -3.15296 -4.10964 -1.14887

C -1.35457 -3.92058 0.88870

H -1.87571 -4.87632 0.86155

C -0.41118 -3.66166 1.85756

H -0.17489 -4.41134 2.60972

C 0.25713 -2.42250 1.87976

H 0.99944 -2.25158 2.65465

C -0.00949 -1.43400 0.94445

C -0.98467 -1.69781 -0.05271

C -1.66311 -2.94674 -0.08641

C -2.43447 0.19726 -2.82722

C -1.65305 1.34114 -2.55027

C -1.81517 2.50174 -3.28903

H -1.23100 3.39337 -3.07079

C -2.75240 2.51967 -4.32786

H -2.88362 3.42874 -4.91294

C -3.51873 1.38974 -4.62192

H -4.24082 1.41915 -5.43530

C -3.36710 0.22867 -3.87375

H -3.97696 -0.64261 -4.10592

C 0.47642 1.12364 2.43911

C 1.31568 2.40500 2.45040

H 0.97330 3.03594 3.28077

H 1.19735 2.98738 1.52927

H 2.37887 2.20212 2.61757

C 0.72152 0.30815 3.70918

H 1.74992 -0.05507 3.79833

H 0.03341 -0.53952 3.79310

H 0.53359 0.95831 4.57361

C -1.01307 1.47893 2.34423

H -1.21867 2.16556 1.51483

H -1.31395 1.98420 3.27108

H -1.64636 0.58859 2.23867

C 2.66169 -0.19907 0.48724

C 3.41156 -0.76044 1.69491

H 3.49984 -0.03110 2.50691

H 4.43226 -1.01480 1.38028

H 2.96394 -1.67903 2.08871

C 3.35347 1.07546 -0.00783

H 2.89913 1.46916 -0.92129

H 4.39658 0.82828 -0.24320

H 3.37053 1.87400 0.73895

C 2.64228 -1.22435 -0.65287

H 2.22538 -2.18984 -0.34612

H 3.67382 -1.39700 -0.98507

H 2.08073 -0.85899 -1.52276

**ts_2^NOO^-2^NO2^_singlet**

Au -0.38115 1.11833 0.94898

P -0.41714 -1.16952 0.19951

O -2.99590 1.33264 1.62102

O -2.39096 1.66192 2.86501

N 0.88529 1.38575 -0.64408

N -1.46170 0.83070 2.59047

C 1.34395 2.63210 -0.80349

C 2.22717 2.91609 -1.87256

H 2.60048 3.92661 -2.00813

C 2.60318 1.91186 -2.72511

H 3.28336 2.11876 -3.55042

C 2.47903 -0.48348 -3.39007

H 3.15840 -0.29834 -4.22082

C 1.97866 -1.74493 -3.15903

H 2.25776 -2.57399 -3.80587

C 1.10006 -1.97263 -2.08118

H 0.72432 -2.98029 -1.92699

C 0.71203 -0.95028 -1.22901

C 1.23048 0.35257 -1.46193

C 2.11773 0.59082 -2.54719

C 0.86533 3.60066 0.17571

C -0.02121 3.11889 1.16716

C -0.53204 3.98950 2.11578

H -1.22726 3.64234 2.87750

C -0.16405 5.33961 2.09180

H -0.56941 6.01857 2.84049

C 0.71599 5.81914 1.12277

H 0.99996 6.86947 1.11330

C 1.22849 4.95370 0.16255

H 1.90756 5.34102 -0.59461

C -2.08521 -1.68135 -0.49795

C -3.05514 -1.91760 0.66282

H -4.06420 -2.03659 0.24734

H -3.09013 -1.07040 1.35700

H -2.82489 -2.83381 1.21557

C -2.02078 -2.91710 -1.39639

H -1.62649 -3.80063 -0.88474

H -1.43796 -2.73933 -2.30585

H -3.04291 -3.15922 -1.71567

C -2.54653 -0.47469 -1.32712

H -2.69783 0.41777 -0.70914

H -3.51011 -0.71713 -1.79338

H -1.84312 -0.23528 -2.13449

C 0.44984 -2.32227 1.40921

C 0.63824 -3.74343 0.88098

H -0.31385 -4.25207 0.69947

H 1.17266 -4.32610 1.64295

H 1.24456 -3.78180 -0.02974

C -0.34296 -2.35358 2.72056

H -0.56252 -1.35328 3.10843

H 0.25920 -2.87906 3.47294

H -1.28724 -2.89669 2.62488

C 1.81605 -1.67108 1.65922

H 2.42885 -1.61988 0.75176

H 2.36302 -2.27363 2.39556

H 1.72104 -0.65936 2.07420

**ts_2^NOO^-2^O^+NO_singlet**

Au -0.29224 1.01444 -1.06326

P 0.90304 0.16288 0.80605

O 1.07374 3.30070 -1.58945

O 0.04527 3.18462 -0.64482

N -1.30040 -0.73906 -0.99059

N 1.34095 2.28991 -2.15777

C -2.25807 -0.89357 -1.91498

C -2.98617 -2.10644 -1.95948

H -3.76253 -2.23685 -2.70769

C -2.70476 -3.10018 -1.05981

H -3.25830 -4.03785 -1.08701

C -1.35991 -3.91468 0.87133

H -1.90209 -4.85905 0.86294

C -0.36398 -3.68456 1.79410

H -0.10733 -4.44652 2.52703

C 0.32986 -2.45849 1.79814

H 1.10768 -2.31033 2.54258

C 0.04025 -1.45603 0.88570

C -0.98309 -1.69369 -0.07036

C -1.69190 -2.92598 -0.08087

C -2.45911 0.25179 -2.78823

C -1.61910 1.36604 -2.55857

C -1.77856 2.52685 -3.29840

H -1.16179 3.40218 -3.10556

C -2.76009 2.57703 -4.29362

H -2.88519 3.48799 -4.87705

C -3.57909 1.47478 -4.54364

H -4.33738 1.52534 -5.32232

C -3.43640 0.31496 -3.79108

H -4.09210 -0.53214 -3.98436

C 0.45272 1.09717 2.37527

C 1.27490 2.38784 2.43496

H 0.90189 2.99790 3.26786

H 1.16629 2.98212 1.52012

H 2.33641 2.19781 2.62730

C 0.65164 0.26798 3.64464

H 1.68117 -0.07646 3.78051

H -0.02131 -0.59518 3.68281

H 0.40794 0.90187 4.50721

C -1.03531 1.44037 2.22744

H -1.21641 2.13362 1.39781

H -1.37513 1.93423 3.14696

H -1.65647 0.54614 2.08928

C 2.73206 -0.18365 0.52625

C 3.45738 -0.64406 1.79187

H 3.48149 0.13276 2.56281

H 4.49896 -0.87155 1.53013

H 3.03328 -1.55663 2.22307

C 3.39461 1.09122 -0.00759

H 3.00635 1.37097 -0.99305

H 4.46817 0.89901 -0.13019

H 3.28901 1.94728 0.66490

C 2.79896 -1.26715 -0.55537

H 2.41365 -2.23177 -0.20816

H 3.84758 -1.41003 -0.84592

H 2.24890 -0.97591 -1.45988

**ts_2^NOO^-2^O^_free_triplet**

Au -0.36516 1.13718 0.90987

P -0.43460 -1.15428 0.20505

O -2.14393 1.13748 3.09059

O -2.49615 1.37464 1.69894

N 0.87329 1.39143 -0.67207

N -0.90313 0.86066 2.97444

C 1.33327 2.63957 -0.84616

C 2.21574 2.90673 -1.91901

H 2.58865 3.91605 -2.06561

C 2.58963 1.89118 -2.75909

H 3.26928 2.08689 -3.58751

C 2.45784 -0.51000 -3.39978

H 3.13555 -0.33374 -4.23379

C 1.95272 -1.76752 -3.15810

H 2.22574 -2.60228 -3.80018

C 1.07828 -1.98386 -2.07469

H 0.69905 -2.98856 -1.91048

C 0.69891 -0.95439 -1.22679

C 1.21749 0.34440 -1.47614

C 2.10232 0.57291 -2.56538

C 0.85230 3.61485 0.11933

C -0.02714 3.12821 1.11254

C -0.56146 3.98175 2.06247

H -1.25093 3.61318 2.81889

C -0.20908 5.33552 2.03629

H -0.62619 6.01110 2.78166

C 0.66742 5.82736 1.06757

H 0.93430 6.88214 1.06018

C 1.19744 4.97342 0.10722

H 1.87459 5.37034 -0.64697

C -2.10801 -1.65106 -0.49317

C -3.11558 -1.76516 0.65478

H -4.11939 -1.85220 0.21912

H -3.11368 -0.87815 1.29756

H -2.95079 -2.65739 1.26483

C -2.07476 -2.94985 -1.29839

H -1.74579 -3.80872 -0.70461

H -1.45125 -2.87224 -2.19489

H -3.09534 -3.16788 -1.63946

C -2.50669 -0.48402 -1.40651

H -2.63244 0.44879 -0.84422

H -3.47404 -0.71641 -1.86997

H -1.78527 -0.32064 -2.21660

C 0.42340 -2.31121 1.41484

C 0.82750 -3.65506 0.80822

H -0.02334 -4.21802 0.41148

H 1.27426 -4.26735 1.60256

H 1.58538 -3.54942 0.02521

C -0.48546 -2.53599 2.62673

H -0.88075 -1.59949 3.03544

H 0.10804 -3.01314 3.41710

H -1.32041 -3.20531 2.40002

C 1.68279 -1.55062 1.85170

H 2.34068 -1.31168 1.00668

H 2.25418 -2.18513 2.54137

H 1.44073 -0.62501 2.38688

**ts_2-2^NOO^_triplet**

Au 8.24230 3.34411 6.39271

P 7.59861 1.18110 5.61552

N 6.38762 3.90406 5.71041

N 10.01218 2.94328 7.03167

O 9.79725 3.11028 8.95645

O 9.80991 2.05106 9.52145

C 5.93297 1.64513 4.99965

C 5.04709 0.76639 4.39629

H 5.31979 -0.27732 4.26385

C 3.78134 1.18962 3.94254

H 3.11320 0.46879 3.47592

C 3.39287 2.50249 4.09051

H 2.41502 2.83450 3.74486

C 4.26373 3.43900 4.69101

C 5.53911 3.00474 5.14231

C 3.93607 4.80860 4.86901

H 2.96289 5.16647 4.53498

C 4.82623 5.67478 5.44993

H 4.57195 6.72199 5.58390

C 6.08797 5.19686 5.87874

C 7.13461 5.99811 6.50854

C 8.32613 5.32231 6.86666

C 9.36864 6.01619 7.46221

H 10.28855 5.50129 7.73482

C 9.23412 7.38614 7.71139

H 10.05283 7.93025 8.18000

C 8.06236 8.05885 7.36470

H 7.96669 9.12448 7.56273

C 7.01361 7.37042 6.76295

H 6.10685 7.90958 6.49471

C 8.62439 0.64194 4.13610

C 8.41422 1.73533 3.08153

H 7.38014 1.77642 2.72173

H 9.05942 1.52188 2.21994

H 8.69339 2.72870 3.45677

C 10.09930 0.61792 4.55299

H 10.42323 1.56307 5.00495

H 10.71006 0.45002 3.65671

H 10.32503 -0.18738 5.25734

C 8.21259 -0.71890 3.57672

H 8.37845 -1.53048 4.29276

H 8.83087 -0.93607 2.69574

H 7.16888 -0.74205 3.24550

C 7.35307 -0.05735 7.00407

C 6.68055 0.73681 8.13101

H 7.32292 1.53722 8.51583

H 6.47259 0.05662 8.96690

H 5.72376 1.17154 7.81554

C 6.46064 -1.24043 6.62362

H 5.42771 -0.93346 6.42980

H 6.43333 -1.93417 7.47398

H 6.83346 -1.80187 5.76142

C 8.72872 -0.55198 7.45821

H 9.18006 -1.23636 6.73216

H 8.60685 -1.10874 8.39609

H 9.42911 0.26785 7.65659

**ts_2-2^NOO^_singlet_UKS2**

Au 8.59959 4.08385 6.43716

P 7.10455 2.46947 5.53204

N 7.14737 5.42934 5.87864

N 10.00076 2.89697 6.98583

O 9.08338 3.56053 9.68311

O 9.97602 2.97444 9.15019

C 5.78519 3.63572 5.01671

C 4.60839 3.26073 4.38820

H 4.41846 2.21344 4.16650

C 3.63353 4.21071 4.02147

H 2.72202 3.87758 3.52965

C 3.83078 5.54785 4.28618

H 3.07856 6.28500 4.00926

C 5.01684 5.98109 4.92002

C 5.99729 5.01815 5.27967

C 5.29181 7.34089 5.22170

H 4.55469 8.09908 4.95989

C 6.46431 7.70277 5.83391

H 6.66942 8.74375 6.06553

C 7.41465 6.70721 6.16665

C 8.70418 6.93807 6.81434

C 9.50499 5.79757 7.06695

C 10.74734 5.93985 7.66678

H 11.36861 5.06569 7.85640

C 11.20128 7.21311 8.02681

H 12.17671 7.32335 8.49839

C 10.41603 8.34053 7.78598

H 10.77673 9.32724 8.06948

C 9.17046 8.20732 7.18031

H 8.57069 9.09670 6.99536

C 7.78850 1.64505 3.98762

C 7.99813 2.78233 2.98030

H 7.05348 3.22607 2.64724

H 8.50926 2.37909 2.09687

H 8.63393 3.58053 3.38588

C 9.14559 1.02168 4.33399

H 9.84084 1.74805 4.77086

H 9.59762 0.64053 3.40933

H 9.05934 0.18006 5.02691

C 6.85806 0.58218 3.40468

H 6.73793 -0.27502 4.07540

H 7.30083 0.20295 2.47421

H 5.86848 0.97602 3.14938

C 6.38563 1.36712 6.86780

C 6.07175 2.30838 8.03789

H 6.97046 2.78582 8.44623

H 5.61695 1.72310 8.84741

H 5.35568 3.09030 7.75588

C 5.10261 0.64856 6.44737

H 4.27914 1.34888 6.27267

H 4.79310 -0.01083 7.26868

H 5.23089 0.02125 5.56055

C 7.46121 0.35761 7.27754

H 7.61440 -0.41256 6.51400

H 7.13386 -0.15319 8.19206

H 8.42573 0.83550 7.49175

**ts_2-2^NOO^_singlet_UKS**

Au 8.23181 3.31764 6.40108

P 7.59388 1.16823 5.61437

N 6.40074 3.87136 5.71652

N 9.98445 2.86090 7.05924

O 9.80692 3.23888 8.97060

O 9.73299 2.21983 9.60201

C 5.93078 1.62978 4.99952

C 5.04049 0.74530 4.39975

H 5.30602 -0.29985 4.26843

C 3.78878 1.18608 3.95182

H 3.10763 0.47857 3.48292

C 3.40743 2.52397 4.10043

H 2.43025 2.85206 3.75005

C 4.26262 3.45092 4.68962

C 5.55012 3.00207 5.14553

C 3.92514 4.84104 4.86876

H 2.95273 5.19292 4.53226

C 4.81691 5.69509 5.45253

H 4.55965 6.74251 5.58702

C 6.08133 5.23427 5.89258

C 7.10548 5.98873 6.49836

C 8.32692 5.28605 6.87491

C 9.36892 5.97661 7.46109

H 10.28399 5.45559 7.73702

C 9.25267 7.35264 7.70277

H 10.07472 7.89400 8.16775

C 8.06577 8.05104 7.34295

H 7.99657 9.11953 7.53849

C 7.01725 7.39576 6.75447

H 6.11881 7.94433 6.48156

C 8.62261 0.64521 4.12970

C 8.40795 1.74605 3.08387

H 7.37323 1.78590 2.72570

H 9.05268 1.54026 2.22006

H 8.68456 2.73727 3.46618

C 10.09696 0.61870 4.54628

H 10.41841 1.55643 5.01499

H 10.70772 0.46630 3.64717

H 10.32409 -0.19736 5.23764

C 8.20923 -0.71143 3.56177

H 8.37941 -1.52832 4.27068

H 8.82441 -0.92030 2.67658

H 7.16422 -0.73255 3.23467

C 7.35921 -0.07376 7.00264

C 6.67619 0.71320 8.12862

H 7.31677 1.50976 8.52427

H 6.46184 0.02643 8.95750

H 5.72176 1.14980 7.80858

C 6.48157 -1.26625 6.61815

H 5.44628 -0.97053 6.41968

H 6.45865 -1.96012 7.46853

H 6.86463 -1.82321 5.75762

C 8.73983 -0.55026 7.46137

H 9.20340 -1.22854 6.73734

H 8.62038 -1.10903 8.39846

H 9.42745 0.28012 7.66191

**ts_2-2^NOO^_singlet_BS**

Au 8.71268 1.58971 6.54319

P 10.20652 -0.18700 5.98821

N 7.25423 0.20572 6.13396

N 10.00042 3.00464 6.87535

O 10.07513 3.24903 8.71489

O 9.38776 2.53494 9.40266

C 8.92773 -1.43218 5.55701

C 9.19998 -2.71216 5.10089

H 10.22897 -3.03790 4.97155

C 8.16778 -3.62283 4.79678

H 8.42226 -4.61870 4.43995

C 6.84932 -3.25783 4.95473

H 6.04797 -3.95905 4.72661

C 6.51956 -1.96330 5.41427

C 7.56618 -1.04810 5.70772

C 5.18518 -1.51580 5.59916

H 4.36459 -2.19927 5.38415

C 4.92438 -0.24379 6.03992

H 3.90219 0.09486 6.18019

C 5.99989 0.63704 6.30700

C 5.87726 2.02039 6.76027

C 7.08248 2.74005 6.94489

C 7.04712 4.06190 7.36125

H 7.97077 4.62322 7.49472

C 5.81528 4.67732 7.60805

H 5.78889 5.71512 7.93694

C 4.62376 3.97319 7.43533

H 3.67014 4.45928 7.63090

C 4.65047 2.64895 7.00929

H 3.71217 2.11372 6.87522

C 11.22710 0.16477 4.45010

C 10.20192 0.37824 3.32946

H 9.65110 -0.53706 3.08680

H 10.73134 0.70066 2.42398

H 9.47803 1.16513 3.57941

C 12.00642 1.46568 4.67566

H 11.35680 2.30257 4.95654

H 12.50865 1.73297 3.73709

H 12.78073 1.36856 5.44214

C 12.19140 -0.96236 4.08303

H 12.97252 -1.10308 4.83745

H 12.69573 -0.69678 3.14461

H 11.68675 -1.91903 3.91192

C 11.10521 -0.84400 7.49841

C 10.05182 -0.86772 8.61309

H 9.67648 0.13336 8.85615

H 10.51298 -1.27304 9.52311

H 9.20012 -1.51317 8.36397

C 11.66572 -2.25442 7.31118

H 10.87236 -2.99677 7.17455

H 12.20958 -2.53245 8.22346

H 12.37109 -2.32991 6.47852

C 12.22499 0.13793 7.85430

H 13.06265 0.07734 7.15158

H 12.61481 -0.11842 8.84759

H 11.87793 1.17753 7.89460

**2^NCCH2^_triplet**

Au 2.19432 9.79000 5.48501

P 2.33240 7.52023 4.73413

N 3.60685 10.08731 3.84958

N 0.48405 9.66472 7.03632

C 4.02836 11.33661 3.71589

C 4.97895 11.62552 2.70047

H 5.33852 12.64108 2.56295

C 5.44031 10.61151 1.90036

H 6.17044 10.82151 1.11955

C 5.44395 8.18405 1.30651

H 6.17407 8.35776 0.51714

C 4.97288 6.91501 1.56098

H 5.32820 6.07108 0.97322

C 4.03038 6.69427 2.58713

H 3.69231 5.67634 2.76349

C 3.54053 7.73161 3.36367

C 4.03395 9.04557 3.10662

C 4.98935 9.27616 2.07798

C 3.45889 12.30654 4.66059

C 2.55103 11.82221 5.63790

C 2.00024 12.72916 6.53801

H 1.29198 12.38908 7.29274

C 2.33618 14.08547 6.49055

H 1.89437 14.77919 7.20492

C 3.23238 14.55455 5.53128

H 3.49285 15.61043 5.49276

C 3.79104 13.66741 4.61747

H 4.48483 14.04789 3.86986

C 0.73723 6.92715 3.93097

C -0.26911 6.61453 5.04110

H -1.25502 6.44491 4.58889

H -0.36760 7.44578 5.74962

H -0.00805 5.70637 5.59604

C 0.89609 5.72496 3.00091

H 1.52183 5.95854 2.13339

H -0.09495 5.45095 2.61517

H 1.30218 4.84178 3.50314

C 0.24610 8.12867 3.11236

H 0.04036 9.00142 3.74428

H -0.68786 7.85892 2.60224

H 0.96992 8.42266 2.34207

C 3.11167 6.34280 5.98166

C 3.05159 4.86683 5.59240

H 2.02386 4.49311 5.53719

H 3.56926 4.27704 6.36070

H 3.55152 4.65764 4.64076

C 2.39001 6.55921 7.31786

H 2.47409 7.59975 7.65224

H 2.85574 5.92170 8.08074

H 1.32803 6.29824 7.27326

C 4.56881 6.79492 6.13025

H 5.16198 6.58735 5.23349

H 5.02651 6.25433 6.96886

H 4.64434 7.86838 6.34990

C 0.12511 9.80716 8.14420

H 0.41902 10.27483 10.21550

C -0.29334 9.97712 9.44835

H -1.33749 9.81903 9.71068

**2^NCCH2^_singlet**

Au 2.44650 9.78003 5.60477

P 2.39328 7.50797 4.88060

N 3.62970 10.06995 3.97830

N 1.37188 9.60569 7.26327

C 4.04723 11.32849 3.79147

C 4.86196 11.62684 2.67273

H 5.20399 12.64524 2.51421

C 5.21228 10.62758 1.80333

H 5.84289 10.84601 0.94238

C 5.09236 8.22857 1.14437

H 5.72557 8.42295 0.27991

C 4.62117 6.95992 1.39870

H 4.87956 6.13555 0.73747

C 3.79737 6.71966 2.51624

H 3.44028 5.70750 2.68593

C 3.43866 7.73586 3.38825

C 3.93599 9.04411 3.13557

C 4.76033 9.29758 2.00562

C 3.60175 12.28604 4.79705

C 2.77448 11.77818 5.82684

C 2.32522 12.62030 6.83221

H 1.70681 12.23229 7.63940

C 2.68185 13.97289 6.81386

H 2.32749 14.63302 7.60436

C 3.49103 14.48164 5.79729

H 3.76554 15.53458 5.79390

C 3.95446 13.64229 4.79002

H 4.59148 14.05020 4.00727

C 0.69783 6.94799 4.29872

C -0.11795 6.52202 5.52228

H -1.16508 6.39448 5.21915

H -0.09201 7.26938 6.32284

H 0.22192 5.56424 5.92991

C 0.74381 5.81521 3.27345

H 1.20154 6.13266 2.33083

H -0.28724 5.51487 3.04472

H 1.26454 4.92522 3.64183

C 0.06764 8.19040 3.65643

H -0.10152 8.99245 4.38565

H -0.90926 7.91760 3.23646

H 0.67790 8.58377 2.83337

C 3.32382 6.35775 6.04050

C 3.34506 4.90562 5.56607

H 2.34429 4.46203 5.53256

H 3.93465 4.31393 6.27880

H 3.81924 4.78618 4.58602

C 2.68943 6.45202 7.43404

H 2.59293 7.48624 7.78361

H 3.33366 5.91441 8.14169

H 1.70139 5.98577 7.47812

C 4.75087 6.91710 6.10178

H 5.27021 6.84633 5.13965

H 5.32437 6.33559 6.83474

H 4.76951 7.96353 6.43330

C 0.17208 9.80603 7.28437

H -1.52447 10.97915 7.64729

C -1.13046 9.99667 7.39835

H -1.82439 9.16880 7.27342

**8’_triplet**

Au 2.19089 9.77619 5.46988

P 2.25210 7.49222 4.74319

N 3.53961 10.06532 3.95314

C 3.99523 11.31803 3.82515

C 4.95811 11.60907 2.82889

H 5.32710 12.62458 2.72094

C 5.41430 10.60824 2.01287

H 6.15526 10.82156 1.24346

C 5.38647 8.20566 1.36342

H 6.12641 8.39696 0.58766

C 4.89979 6.93487 1.57357

H 5.25041 6.10429 0.96460

C 3.94664 6.69972 2.58386

H 3.59250 5.68311 2.72993

C 3.46505 7.72363 3.38460

C 3.97054 9.03418 3.17501

C 4.93719 9.28028 2.16155

C 3.42738 12.28611 4.75377

C 2.48669 11.78811 5.68373

C 1.86677 12.65201 6.57257

H 1.11646 12.27775 7.26574

C 2.19933 14.01048 6.56394

H 1.71495 14.68680 7.26694

C 3.14082 14.50647 5.66104

H 3.39251 15.56505 5.66126

C 3.75160 13.64990 4.75218

H 4.47391 14.05177 4.04397

C 0.65538 6.94686 3.90664

C -0.38651 6.63364 4.98068

H -1.34015 6.40264 4.48898

H -0.56257 7.48880 5.64066

H -0.11697 5.76161 5.58688

C 0.82271 5.75242 2.96715

H 1.46543 5.98607 2.11262

H -0.16476 5.49684 2.56104

H 1.20553 4.85840 3.46819

C 0.20628 8.16623 3.09060

H -0.00089 9.03635 3.72577

H -0.72236 7.91840 2.56042

H 0.94908 8.45218 2.33559

C 3.04887 6.29875 5.96627

C 3.02081 4.83930 5.51440

H 2.00127 4.44657 5.44162

H 3.54553 4.23407 6.26540

H 3.53244 4.67317 4.56092

C 2.33022 6.43391 7.31138

H 2.37088 7.45621 7.70620

H 2.83141 5.78309 8.03946

H 1.28442 6.11548 7.26308

C 4.49559 6.78067 6.12915

H 5.09295 6.62686 5.22452

H 4.96533 6.21183 6.94167

H 4.54782 7.84233 6.40495

N 0.88281 9.80419 6.98595

C 0.08332 8.96430 7.73466

C 1.15179 9.72261 8.34386

H -0.10197 7.89558 7.64372

H 1.23168 10.53515 9.06833

**8’_singlet**

Au 2.13953 9.76359 5.50997

P 2.25243 7.48920 4.74436

N 3.60110 10.04582 4.01002

C 4.07593 11.28751 3.92434

C 5.09375 11.57707 2.98022

H 5.49720 12.58286 2.91018

C 5.55206 10.58375 2.15464

H 6.32810 10.79560 1.42009

C 5.44313 8.20535 1.41050

H 6.20904 8.38899 0.65831

C 4.88766 6.95380 1.55744

H 5.20843 6.13297 0.91933

C 3.90487 6.72140 2.54132

H 3.50301 5.71654 2.63681

C 3.45900 7.73175 3.37810

C 4.02295 9.02894 3.22066

C 5.02408 9.26877 2.23970

C 3.44430 12.26381 4.81394

C 2.42084 11.78810 5.66872

C 1.69492 12.69348 6.42884

H 0.85045 12.34640 7.02303

C 2.01205 14.05483 6.39968

H 1.43750 14.75750 7.00191

C 3.05248 14.51947 5.59512

H 3.29836 15.57932 5.57605

C 3.75918 13.62951 4.79351

H 4.54420 14.01110 4.14309

C 0.63505 6.96416 3.93771

C -0.37647 6.67215 5.04801

H -1.35973 6.49296 4.59413

H -0.48363 7.52543 5.72740

H -0.11967 5.77626 5.62479

C 0.74636 5.77327 2.98635

H 1.37230 6.00025 2.11741

H -0.25679 5.54407 2.60311

H 1.12207 4.86607 3.46811

C 0.18125 8.19605 3.14232

H 0.00076 9.06272 3.79002

H -0.76206 7.96328 2.63133

H 0.91110 8.47928 2.37382

C 3.07109 6.23964 5.90011

C 3.00694 4.79302 5.41343

H 1.98048 4.41349 5.37476

H 3.55831 4.16128 6.12262

H 3.47233 4.64899 4.43337

C 2.39948 6.34552 7.27033

H 2.48099 7.35617 7.68636

H 2.90318 5.66242 7.96664

H 1.34343 6.05748 7.24254

C 4.52994 6.69256 6.03610

H 5.10238 6.53644 5.11622

H 5.00888 6.10829 6.83234

H 4.60754 7.75220 6.31431

N 0.61605 9.86688 6.86564

C 0.47691 8.87490 7.93848

C 0.98623 10.01078 8.26441

H 0.04627 7.89848 8.10530

H 1.38582 10.76499 8.93026

**2^CH2CN^_triplet**

Au 2.29809 9.71768 5.53469

P 2.37588 7.48944 4.66075

N 3.64590 10.08675 3.86194

C 4.08186 11.33564 3.78078

C 4.99514 11.66845 2.74490

H 5.36574 12.68488 2.64985

C 5.40589 10.69559 1.86973

H 6.10675 10.93946 1.07215

C 5.34140 8.30778 1.13691

H 6.04077 8.51640 0.32840

C 4.85794 7.03365 1.33679

H 5.17221 6.22048 0.68571

C 3.95629 6.76738 2.38850

H 3.60776 5.74620 2.51914

C 3.51945 7.76413 3.24571

C 4.02409 9.08339 3.04332

C 4.93921 9.35930 1.98946

C 3.56506 12.25921 4.79920

C 2.68689 11.73437 5.78293

C 2.18373 12.59908 6.74993

H 1.49778 12.22920 7.51123

C 2.53872 13.95135 6.76270

H 2.13387 14.61142 7.52897

C 3.40663 14.45946 5.79740

H 3.68242 15.51211 5.80617

C 3.91724 13.61556 4.81705

H 4.58910 14.02648 4.06556

C 0.73828 6.94390 3.91134

C -0.20697 6.56557 5.05442

H -1.21834 6.43127 4.64908

H -0.25780 7.34819 5.82102

H 0.07804 5.62146 5.53177

C 0.84366 5.79837 2.90494

H 1.41172 6.08772 2.01483

H -0.16851 5.53833 2.56737

H 1.28547 4.89124 3.32822

C 0.20589 8.19083 3.19299

H 0.03169 9.02274 3.88638

H -0.75275 7.94850 2.71590

H 0.88807 8.53447 2.40514

C 3.21185 6.24761 5.80403

C 3.13892 4.79675 5.33200

H 2.11067 4.42202 5.29594

H 3.68872 4.16549 6.04286

H 3.60111 4.64673 4.35062

C 2.55234 6.38129 7.18254

H 2.63721 7.40339 7.56962

H 3.06349 5.71186 7.88676

H 1.49351 6.10481 7.17439

C 4.67324 6.69817 5.91177

H 5.22461 6.54627 4.97789

H 5.17040 6.11118 6.69486

H 4.75622 7.75715 6.19091

C 0.06906 9.82734 9.62166

H -0.94637 9.64859 9.96958

H 0.83539 10.14396 10.32685

C 0.38448 9.65683 8.28891

N 0.65908 9.52097 7.15593

**2^CH2CN^_singlet**

Au 2.24787 9.78346 5.53675

P 2.30454 7.50033 4.82324

N 3.54066 10.07394 3.96176

C 3.94943 11.33490 3.79327

C 4.83883 11.63875 2.73364

H 5.17343 12.66104 2.58597

C 5.27313 10.63668 1.90600

H 5.96145 10.85855 1.09135

C 5.26658 8.22296 1.28756

H 5.95898 8.41702 0.46972

C 4.81478 6.94653 1.53829

H 5.14767 6.11494 0.92078

C 3.91632 6.70589 2.59680

H 3.58166 5.68609 2.76395

C 3.46032 7.72975 3.41256

C 3.93832 9.04550 3.16614

C 4.84028 9.29878 2.09681

C 3.42296 12.29455 4.75934

C 2.54219 11.79405 5.75209

C 2.04015 12.66361 6.71008

H 1.38287 12.31247 7.50261

C 2.39126 14.01746 6.68827

H 1.99244 14.68627 7.44945

C 3.24933 14.51223 5.70808

H 3.51996 15.56602 5.69755

C 3.76701 13.65253 4.74598

H 4.44351 14.04718 3.99039

C 0.67601 6.91421 4.07656

C -0.26173 6.45132 5.19373

H -1.25077 6.25335 4.76095

H -0.39796 7.20803 5.97403

H 0.07780 5.52255 5.66368

C 0.84330 5.79512 3.04705

H 1.39285 6.13103 2.16222

H -0.15503 5.48934 2.70763

H 1.33141 4.90400 3.45417

C 0.09141 8.14906 3.37977

H -0.13774 8.95343 4.08921

H -0.84603 7.87012 2.88151

H 0.76521 8.54591 2.60970

C 3.14200 6.33002 6.03621

C 3.14142 4.87415 5.57037

H 2.13086 4.46034 5.49019

H 3.67687 4.27177 6.31582

H 3.66013 4.73038 4.61713

C 2.43580 6.44222 7.39206

H 2.52547 7.44337 7.82539

H 2.92206 5.75132 8.09260

H 1.37806 6.16551 7.35107

C 4.57934 6.84352 6.18318

H 5.16484 6.71635 5.26638

H 5.07567 6.27478 6.97975

H 4.61160 7.90123 6.47609

C 0.94342 9.67742 7.12381

H 0.23173 10.50585 7.03932

H 0.38195 8.73935 7.11740

C 1.70005 9.77599 8.35076

N 2.34227 9.83536 9.31423

**2^CHCHN’^_triplet**

Au -0.21254 1.08931 0.87118

P -0.29504 -1.16764 0.08491

N 0.94621 1.39227 -0.80814

N -3.22095 1.37588 3.83870

C 1.37191 2.64684 -0.97935

C 2.17393 2.95862 -2.10438

H 2.52448 3.97536 -2.25359

C 2.50130 1.97048 -2.99612

H 3.11694 2.19912 -3.86523

C 2.34565 -0.41828 -3.69226

H 2.95854 -0.21745 -4.56978

C 1.86257 -1.68442 -3.44796

H 2.08836 -2.49967 -4.13215

C 1.07094 -1.93653 -2.30937

H 0.70671 -2.94753 -2.15026

C 0.75514 -0.93344 -1.40619

C 1.24625 0.37689 -1.66095

C 2.04661 0.63968 -2.80554

C 0.95448 3.59062 0.05519

C 0.15983 3.08047 1.11423

C -0.24019 3.93204 2.13543

H -0.83736 3.55958 2.96479

C 0.12764 5.28160 2.11029

H -0.19272 5.93935 2.91696

C 0.90037 5.78718 1.06649

H 1.18323 6.83778 1.05320

C 1.31565 4.94390 0.04139

H 1.92281 5.34900 -0.76587

C -1.99593 -1.68820 -0.52632

C -2.99665 -1.55529 0.62571

H -4.00270 -1.75610 0.23571

H -3.00210 -0.54912 1.05575

H -2.81402 -2.26785 1.43495

C -2.03255 -3.10968 -1.08614

H -1.35650 -3.24800 -1.93589

H -3.04783 -3.31382 -1.45084

H -1.80785 -3.86348 -0.32436

C -2.35611 -0.67687 -1.62200

H -2.31465 0.35801 -1.25726

H -3.38638 -0.86472 -1.94982

H -1.70604 -0.76203 -2.49969

C 0.61860 -2.36925 1.20708

C 1.10218 -3.63406 0.49599

H 0.29039 -4.20777 0.03816

H 1.57744 -4.28580 1.24089

H 1.85821 -3.41384 -0.26422

C -0.29106 -2.74214 2.38024

H -0.74087 -1.86744 2.86290

H 0.30900 -3.26321 3.13722

H -1.09398 -3.42348 2.07942

C 1.83793 -1.58691 1.71166

H 2.48102 -1.25220 0.88784

H 2.44159 -2.24304 2.35188

H 1.55510 -0.71236 2.31001

C -2.53925 1.52814 2.74936

C -1.28815 0.91988 2.53706

H -2.98155 2.16471 1.96449

H -0.87608 0.32553 3.35580

**2^CHCHN’^_singlet**

Au -0.21027 1.08574 0.87162

P -0.29149 -1.17035 0.08487

N 0.94687 1.39095 -0.80747

N -3.23350 1.40498 3.81286

C 1.37030 2.64634 -0.97894

C 2.17125 2.95928 -2.10435

H 2.52020 3.97656 -2.25372

C 2.49968 1.97162 -2.99628

H 3.11467 2.20122 -3.86560

C 2.34591 -0.41702 -3.69341

H 2.95826 -0.21525 -4.57109

C 1.86346 -1.68359 -3.44996

H 2.08926 -2.49814 -4.13496

C 1.07249 -1.93696 -2.31118

H 0.70842 -2.94817 -2.15280

C 0.75715 -0.93477 -1.40687

C 1.24736 0.37607 -1.66089

C 2.04667 0.64021 -2.80585

C 0.95182 3.58926 0.05601

C 0.15901 3.07718 1.11543

C -0.24067 3.92713 2.13825

H -0.83510 3.55367 2.96935

C 0.12441 5.27739 2.11342

H -0.19608 5.93401 2.92094

C 0.89470 5.78504 1.06873

H 1.17541 6.83622 1.05571

C 1.31057 4.94319 0.04272

H 1.91633 5.34988 -0.76480

C -1.99439 -1.68719 -0.52316

C -2.99370 -1.54797 0.62946

H -4.00036 -1.74999 0.24171

H -2.99860 -0.53942 1.05434

H -2.81057 -2.25634 1.44225

C -2.03481 -3.11044 -1.07847

H -1.35836 -3.25310 -1.92723

H -3.05034 -3.31294 -1.44335

H -1.81253 -3.86256 -0.31433

C -2.35308 -0.67871 -1.62197

H -2.30712 0.35749 -1.26150

H -3.38467 -0.86403 -1.94704

H -1.70504 -0.76973 -2.50056

C 0.62156 -2.37363 1.20567

C 1.10440 -3.63747 0.49232

H 0.29219 -4.21006 0.03379

H 1.57953 -4.29062 1.23607

H 1.86037 -3.41634 -0.26769

C -0.28828 -2.74783 2.37820

H -0.73611 -1.87344 2.86320

H 0.31127 -3.27183 3.13355

H -1.09269 -3.42691 2.07623

C 1.84112 -1.59238 1.71129

H 2.48423 -1.25664 0.88790

H 2.44471 -2.24959 2.35047

H 1.55841 -0.71864 2.31090

C -2.54607 1.55123 2.74824

C -1.28757 0.91348 2.53389

H -2.96600 2.20369 1.96107

H -0.89260 0.31087 3.35421

**8_triplet**

Au 2.33717 9.78462 5.56416

P 2.31512 7.50706 4.81055

N 3.58677 10.07683 3.94894

C 4.03163 11.32831 3.80173

C 4.91417 11.62554 2.73510

H 5.27704 12.64024 2.60201

C 5.30678 10.62605 1.88331

H 5.98938 10.84280 1.06252

C 5.23173 8.22387 1.22803

H 5.91574 8.41401 0.40224

C 4.75671 6.95453 1.47024

H 5.06190 6.12393 0.83730

C 3.87074 6.71904 2.54062

H 3.51958 5.70393 2.70146

C 3.44570 7.74030 3.37607

C 3.94764 9.04847 3.13646

C 4.84138 9.29725 2.05841

C 3.54480 12.28453 4.79217

C 2.67284 11.78595 5.79191

C 2.16597 12.65130 6.75114

H 1.47980 12.29157 7.51584

C 2.52485 14.00336 6.73353

H 2.12272 14.67466 7.49095

C 3.38945 14.49596 5.75704

H 3.66519 15.54849 5.75074

C 3.89744 13.64037 4.78555

H 4.56677 14.03767 4.02481

C 0.66482 6.93671 4.09826

C -0.22753 6.40126 5.21945

H -1.23351 6.23947 4.81081

H -0.31567 7.09981 6.06083

H 0.12408 5.43657 5.60091

C 0.81547 5.87765 3.00452

H 1.33237 6.26294 2.11990

H -0.19039 5.57584 2.68469

H 1.32444 4.97274 3.35263

C 0.04606 8.20105 3.48998

H -0.17897 8.95372 4.25488

H -0.90002 7.93708 3.00005

H 0.69337 8.65511 2.72839

C 3.17561 6.31527 5.98379

C 3.12807 4.86112 5.51401

H 2.10707 4.47051 5.45934

H 3.67007 4.24348 6.24219

H 3.61718 4.70972 4.54614

C 2.51395 6.44392 7.36040

H 2.62490 7.45275 7.77030

H 3.01045 5.74997 8.05060

H 1.44756 6.20398 7.35344

C 4.62950 6.79159 6.08064

H 5.18217 6.64309 5.14677

H 5.13726 6.21625 6.86530

H 4.69978 7.85185 6.35858

N 0.11425 8.77830 7.37639

C -0.18080 10.16978 7.08244

H -0.66277 10.52879 6.17092

C 1.16436 9.69559 7.26199

H 1.84942 9.79972 8.11643

**8_singlet**

Au 2.33513 9.78766 5.55570

P 2.32142 7.50899 4.80178

N 3.58245 10.06911 3.90917

C 4.01384 11.32028 3.74565

C 4.88514 11.61838 2.66919

H 5.23697 12.63488 2.52087

C 5.28511 10.61308 1.82698

H 5.96062 10.82796 0.99973

C 5.23785 8.19887 1.20699

H 5.91752 8.38225 0.37601

C 4.77691 6.92904 1.47206

H 5.08957 6.09132 0.85210

C 3.89440 6.70030 2.54764

H 3.55344 5.68406 2.72166

C 3.46044 7.72931 3.36844

C 3.95071 9.03919 3.10670

C 4.83770 9.28079 2.02162

C 3.53408 12.28065 4.74162

C 2.68950 11.78508 5.76793

C 2.22771 12.65887 6.74327

H 1.58319 12.29956 7.54341

C 2.58692 14.00992 6.71301

H 2.21821 14.68329 7.48554

C 3.41334 14.49824 5.70266

H 3.69077 15.55021 5.68182

C 3.88729 13.63583 4.71964

H 4.53485 14.02845 3.93799

C 0.68393 6.91606 4.07498

C -0.21089 6.36739 5.18799

H -1.20984 6.18968 4.76832

H -0.32016 7.06539 6.02533

H 0.15113 5.40701 5.57077

C 0.85221 5.86069 2.98084

H 1.37273 6.25135 2.10079

H -0.14835 5.54967 2.65296

H 1.36724 4.96002 3.33161

C 0.05317 8.17421 3.46579

H -0.18369 8.92197 4.23179

H -0.88646 7.90000 2.96886

H 0.70058 8.63746 2.70987

C 3.17829 6.32725 5.98881

C 3.17902 4.87448 5.51312

H 2.16906 4.46393 5.41465

H 3.70336 4.26353 6.25971

H 3.71064 4.73908 4.56572

C 2.48111 6.43217 7.34979

H 2.51980 7.45047 7.74907

H 3.00218 5.77583 8.05866

H 1.43286 6.12335 7.32031

C 4.61715 6.83967 6.12319

H 5.19036 6.72315 5.19708

H 5.12577 6.26315 6.90651

H 4.65226 7.89593 6.42163

N -0.02477 8.85003 7.31504

C -0.21592 10.08729 7.21842

H -1.04465 10.79158 7.15614

C 1.18250 9.73911 7.21857

H 1.77584 9.85979 8.13233

**2^CHCHN^_triplet**

Au -0.79780 -0.82003 1.73162

P 0.06850 -1.93920 3.65651

N -0.43760 -2.62423 0.80217

N -3.45894 0.68308 2.79227

C -0.77725 -2.67020 -0.48908

C -0.55424 -3.86114 -1.22266

H -0.83193 -3.91043 -2.27114

C 0.01746 -4.94061 -0.60143

H 0.19555 -5.86110 -1.15621

C 0.99112 -5.95369 1.45691

H 1.18217 -6.88460 0.92507

C 1.32918 -5.82076 2.78489

H 1.79252 -6.64776 3.31890

C 1.07643 -4.61308 3.46630

H 1.35485 -4.54829 4.51419

C 0.48817 -3.52938 2.83315

C 0.13956 -3.66409 1.46090

C 0.38995 -4.87838 0.76641

C -1.34938 -1.43786 -1.02491

C -1.45959 -0.33737 -0.13714

C -1.98693 0.86010 -0.60061

H -2.08400 1.71403 0.06542

C -2.40559 0.97594 -1.92993

H -2.81891 1.91988 -2.28220

C -2.30064 -0.10473 -2.80405

H -2.63003 -0.00825 -3.83657

C -1.77416 -1.31035 -2.35389

H -1.69870 -2.14793 -3.04477

C -1.22687 -2.35339 4.95873

C -1.87921 -1.06149 5.45933

H -2.66108 -1.33381 6.17995

H -2.36458 -0.50590 4.65235

H -1.17944 -0.39885 5.97602

C -0.65776 -3.13794 6.14007

H -0.20632 -4.09021 5.84403

H -1.48204 -3.37787 6.82460

H 0.07413 -2.55769 6.71216

C -2.28579 -3.18534 4.22331

H -2.70820 -2.64448 3.36636

H -3.11354 -3.38654 4.91505

H -1.89792 -4.15020 3.87841

C 1.68142 -1.18247 4.25636

C 2.56979 -2.14436 5.04657

H 2.07649 -2.56246 5.92958

H 3.44784 -1.58802 5.39987

H 2.94156 -2.96386 4.42327

C 1.35113 0.04924 5.10293

H 0.63138 0.71389 4.61038

H 2.27285 0.62149 5.26898

H 0.95587 -0.22266 6.08734

C 2.42131 -0.76057 2.98054

H 2.60033 -1.60947 2.30837

H 3.40079 -0.34887 3.25661

H 1.88273 0.01590 2.42416

C -2.40904 1.44068 2.83719

C -1.12670 1.00372 2.46806

H -2.54112 2.47621 3.19344

H -0.29228 1.70556 2.51519

**2^CHCHN^_singlet**

Au -0.79642 -0.82057 1.73319

P 0.06979 -1.94108 3.65739

N -0.43579 -2.62351 0.80252

N -3.47219 0.67938 2.79470

C -0.77459 -2.66829 -0.48906

C -0.55218 -3.85888 -1.22332

H -0.82917 -3.90710 -2.27203

C 0.01772 -4.93947 -0.60235

H 0.19509 -5.85981 -1.15761

C 0.98785 -5.95541 1.45624

H 1.17803 -6.88622 0.92392

C 1.32459 -5.82406 2.78472

H 1.78598 -6.65222 3.31862

C 1.07303 -4.61648 3.46674

H 1.35032 -4.55285 4.51503

C 0.48719 -3.53144 2.83367

C 0.13964 -3.66459 1.46101

C 0.38902 -4.87869 0.76587

C -1.34553 -1.43515 -1.02432

C -1.45635 -0.33583 -0.13538

C -1.98307 0.86242 -0.59763

H -2.08091 1.71569 0.06922

C -2.40034 0.98022 -1.92714

H -2.81337 1.92461 -2.27851

C -2.29454 -0.09933 -2.80260

H -2.62285 -0.00134 -3.83532

C -1.76884 -1.30571 -2.35359

H -1.69283 -2.14235 -3.04555

C -1.22508 -2.35177 4.96095

C -1.87889 -1.05861 5.45639

H -2.65757 -1.32906 6.18118

H -2.36948 -0.50911 4.64817

H -1.17915 -0.39147 5.96741

C -0.65422 -3.13097 6.14505

H -0.19993 -4.08295 5.85240

H -1.47811 -3.37085 6.83006

H 0.07588 -2.54684 6.71548

C -2.28330 -3.18769 4.22909

H -2.70614 -2.65070 3.36990

H -3.11097 -3.38612 4.92172

H -1.89494 -4.15387 3.88849

C 1.68391 -1.18504 4.25445

C 2.57127 -2.14721 5.04549

H 2.07814 -2.56250 5.92991

H 3.45092 -1.59201 5.39658

H 2.94053 -2.96876 4.42337

C 1.35544 0.04845 5.09906

H 0.63731 0.71343 4.60433

H 2.27810 0.61926 5.26484

H 0.95882 -0.22108 6.08357

C 2.42341 -0.76594 2.97748

H 2.60105 -1.61603 2.30645

H 3.40353 -0.35494 3.25229

H 1.88522 0.01041 2.42051

C -2.43636 1.42724 2.83572

C -1.12984 0.99790 2.47236

H -2.57383 2.46790 3.18214

H -0.30949 1.71677 2.50603

**2^NCHCH’^_triplet**

Au 2.09302 9.78154 5.41973

P 2.23039 7.50694 4.72235

N 3.55333 10.07981 4.01814

N 0.54930 9.55013 6.60128

C 4.00537 11.33419 3.92481

C 5.00765 11.63985 2.97223

H 5.38151 12.65606 2.89066

C 5.48966 10.65049 2.15535

H 6.25744 10.87586 1.41619

C 5.45713 8.26215 1.44246

H 6.22211 8.46244 0.69369

C 4.94357 6.99413 1.60046

H 5.29883 6.17681 0.97633

C 3.95682 6.74316 2.57537

H 3.58154 5.72851 2.68202

C 3.46912 7.75143 3.39102

C 3.99905 9.06211 3.23144

C 4.99918 9.32243 2.25585

C 3.37898 12.28784 4.83684

C 2.36445 11.78332 5.68867

C 1.70393 12.64824 6.54941

H 0.90260 12.28592 7.19049

C 2.05461 14.00207 6.58798

H 1.53076 14.67418 7.26623

C 3.06245 14.49812 5.76164

H 3.32951 15.55234 5.79721

C 3.72251 13.64515 4.88358

H 4.50214 14.04581 4.23820

C 0.63044 6.93150 3.92855

C -0.36244 6.60862 5.04891

H -1.35004 6.44103 4.59978

H -0.45609 7.44076 5.75854

H -0.09582 5.69423 5.59070

C 0.79766 5.74116 2.98467

H 1.40770 5.99181 2.11031

H -0.19549 5.45968 2.61055

H 1.21935 4.85733 3.47291

C 0.12971 8.14311 3.13061

H -0.11381 8.98950 3.78348

H -0.78955 7.86178 2.60078

H 0.85570 8.47022 2.37509

C 3.01398 6.35644 5.98854

C 2.97456 4.88571 5.57590

H 1.95234 4.49806 5.51849

H 3.50282 4.29437 6.33550

H 3.47666 4.69837 4.62046

C 2.28228 6.55072 7.32120

H 2.37446 7.57914 7.68463

H 2.73766 5.88953 8.06973

H 1.21764 6.30614 7.26665

C 4.46479 6.82553 6.14669

H 5.06855 6.62477 5.25545

H 4.92009 6.28752 6.98800

H 4.52828 7.89846 6.37237

C 0.64562 9.75610 7.89152

H 1.58221 10.09580 8.37362

C -0.42916 9.56379 8.74761

H -0.58474 9.66324 9.81764

**2^NCHCH^_triplet**

Au 2.09505 9.78501 5.44209

P 2.22957 7.50863 4.74608

N 3.54930 10.08111 4.02982

N 0.55500 9.60045 6.65034

C 3.99008 11.33828 3.92213

C 4.99662 11.64057 2.97259

H 5.36336 12.65860 2.88225

C 5.49177 10.64580 2.17071

H 6.26275 10.86825 1.43402

C 5.47531 8.25112 1.47968

H 6.24282 8.44862 0.73277

C 4.96421 6.98294 1.64388

H 5.32336 6.16243 1.02621

C 3.97355 6.73627 2.61576

H 3.59707 5.72238 2.72325

C 3.48178 7.74783 3.42509

C 4.00676 9.05895 3.25661

C 5.00915 9.31573 2.28240

C 3.34596 12.29976 4.81222

C 2.33565 11.79744 5.66915

C 1.64837 12.67129 6.49976

H 0.85187 12.30680 7.14433

C 1.97046 14.03220 6.50416

H 1.42595 14.71131 7.15878

C 2.97762 14.52673 5.67560

H 3.22310 15.58675 5.68575

C 3.66291 13.66478 4.82685

H 4.43885 14.06415 4.17631

C 0.63406 6.96994 3.91560

C -0.39080 6.66722 5.01188

H -1.37382 6.53127 4.54250

H -0.47562 7.49529 5.72723

H -0.16084 5.74185 5.55153

C 0.79451 5.77819 2.97244

H 1.43092 6.01618 2.11351

H -0.19588 5.52112 2.57423

H 1.18426 4.88365 3.46734

C 0.17807 8.19257 3.10758

H -0.05954 9.04615 3.75335

H -0.73591 7.93229 2.55814

H 0.92763 8.50219 2.36813

C 2.98379 6.32416 5.99973

C 2.98357 4.86710 5.53888

H 1.97058 4.46684 5.42833

H 3.48876 4.26051 6.30211

H 3.52857 4.71526 4.60129

C 2.20899 6.45477 7.31451

H 2.25759 7.47212 7.71476

H 2.66606 5.78454 8.05410

H 1.15624 6.17275 7.22267

C 4.41995 6.81295 6.22193

H 5.05136 6.67081 5.33849

H 4.86340 6.23934 7.04587

H 4.45241 7.87272 6.50770

C 0.73640 9.71679 7.94198

H -0.17083 9.61393 8.56375

C 1.92975 9.94901 8.60806

H 2.24348 10.08267 9.63906

**2^NCHCH’^_singlet**

Au 2.09153 9.77958 5.41950

P 2.22992 7.50618 4.72086

N 3.55205 10.07953 4.01811

N 0.55003 9.54977 6.59875

C 4.00209 11.33459 3.92427

C 5.00518 11.64098 2.97275

H 5.37769 12.65767 2.89094

C 5.48988 10.65161 2.15745

H 6.25844 10.87750 1.41925

C 5.46173 8.26247 1.44647

H 6.22745 8.46312 0.69855

C 4.94967 6.99385 1.60485

H 5.30687 6.17649 0.98189

C 3.96174 6.74235 2.57846

H 3.58747 5.72732 2.68526

C 3.47162 7.75075 3.39247

C 4.00021 9.06194 3.23273

C 5.00120 9.32287 2.25828

C 3.37284 12.28790 4.83472

C 2.35960 11.78206 5.68719

C 1.69600 12.64596 6.54678

H 0.89656 12.28160 7.18921

C 2.04255 14.00089 6.58284

H 1.51665 14.67254 7.25997

C 3.04892 14.49867 5.75569

H 3.31248 15.55383 5.78941

C 3.71203 13.64642 4.87926

H 4.49033 14.04851 4.23318

C 0.63070 6.93437 3.92336

C -0.36687 6.61715 5.04120

H -1.35359 6.45210 4.58923

H -0.46019 7.45078 5.74927

H -0.10514 5.70287 5.58555

C 0.79813 5.74119 2.98304

H 1.41290 5.98771 2.11082

H -0.19424 5.46196 2.60522

H 1.21484 4.85731 3.47541

C 0.13643 8.14539 3.12058

H -0.10713 8.99426 3.77025

H -0.78175 7.86532 2.58825

H 0.86613 8.46837 2.36690

C 3.00798 6.35551 5.98993

C 2.96680 4.88464 5.57754

H 1.94401 4.49863 5.51933

H 3.49338 4.29275 6.33786

H 3.46950 4.69618 4.62264

C 2.27257 6.55173 7.32021

H 2.36854 7.57894 7.68606

H 2.72192 5.88713 8.06939

H 1.20680 6.31307 7.26207

C 4.45918 6.82192 6.15183

H 5.06520 6.61917 5.26258

H 4.91086 6.28369 6.99496

H 4.52408 7.89489 6.37688

C 0.66634 9.75164 7.90816

H 1.61339 10.08843 8.37218

C -0.37065 9.56283 8.77404

H -0.53052 9.64862 9.84473

**2^NCHCH^_singlet**

Au 2.09039 9.78252 5.43794

P 2.22590 7.50644 4.74251

N 3.54844 10.07827 4.03004

N 0.55266 9.59777 6.64419

C 3.99122 11.33498 3.92463

C 4.99909 11.63735 2.97667

H 5.36745 12.65498 2.88837

C 5.49321 10.64324 2.17331

H 6.26504 10.86580 1.43754

C 5.47302 8.25013 1.47743

H 6.24115 8.44790 0.73124

C 4.95960 6.98257 1.63878

H 5.31731 6.16268 1.01946

C 3.96839 6.73550 2.61003

H 3.59028 5.72199 2.71499

C 3.47804 7.74611 3.42141

C 4.00497 9.05676 3.25538

C 5.00831 9.31384 2.28221

C 3.34753 12.29603 4.81552

C 2.33374 11.79411 5.66846

C 1.64739 12.66730 6.50081

H 0.84876 12.30320 7.14293

C 1.97368 14.02727 6.51012

H 1.42959 14.70606 7.16542

C 2.98408 14.52132 5.68529

H 3.23276 15.58054 5.69935

C 3.66865 13.65994 4.83523

H 4.44762 14.05893 4.18808

C 0.63021 6.96651 3.91339

C -0.39494 6.66681 5.01017

H -1.37834 6.53258 4.54110

H -0.47821 7.49554 5.72506

H -0.16626 5.74146 5.55028

C 0.79044 5.77171 2.97395

H 1.42546 6.00726 2.11337

H -0.20021 5.51230 2.57795

H 1.18170 4.87943 3.47179

C 0.17489 8.18720 3.10206

H -0.06279 9.04235 3.74573

H -0.73893 7.92565 2.55297

H 0.92484 8.49483 2.36222

C 2.98346 6.32693 5.99857

C 3.00007 4.87130 5.53360

H 1.99156 4.46147 5.41707

H 3.50719 4.26782 6.29798

H 3.55167 4.72754 4.59863

C 2.20136 6.44672 7.30990

H 2.23631 7.46357 7.71334

H 2.66317 5.78020 8.04994

H 1.15264 6.15160 7.21304

C 4.41327 6.83047 6.22885

H 5.04962 6.70214 5.34675

H 4.86030 6.25587 7.05017

H 4.43157 7.88810 6.52354

C 0.75218 9.71761 7.95007

H -0.15150 9.61317 8.57780

C 1.92874 9.93903 8.60778

H 2.24767 10.16769 9.62089

**ts_2^CHCHN’^-8_singlet**

Au -0.26099 1.04086 0.87797

P -0.31010 -1.20734 0.05742

N 0.90362 1.37365 -0.80251

N -2.54061 2.18902 3.75008

C 1.32800 2.63062 -0.95214

C 2.13425 2.96116 -2.06990

H 2.48017 3.98151 -2.20417

C 2.47219 1.98683 -2.97202

H 3.09088 2.23004 -3.83499

C 2.34694 -0.39713 -3.69039

H 2.96409 -0.18149 -4.56140

C 1.88000 -1.67204 -3.45920

H 2.12297 -2.47900 -4.14730

C 1.08429 -1.94311 -2.32825

H 0.73449 -2.96059 -2.17727

C 0.74696 -0.94989 -1.42189

C 1.22122 0.36946 -1.66211

C 2.02811 0.64985 -2.79792

C 0.90972 3.55658 0.09619

C 0.11324 3.03459 1.14931

C -0.28407 3.87236 2.18056

H -0.90028 3.50274 2.99930

C 0.09312 5.22020 2.17515

H -0.22679 5.86610 2.99148

C 0.86763 5.73864 1.13945

H 1.15475 6.78822 1.14095

C 1.27643 4.90869 0.10143

H 1.88330 5.32247 -0.70154

C -2.00012 -1.74061 -0.57229

C -3.01663 -1.59199 0.56318

H -4.01553 -1.81834 0.16895

H -3.03729 -0.57165 0.95726

H -2.83093 -2.27449 1.39724

C -2.02591 -3.17101 -1.10945

H -1.32842 -3.32349 -1.93926

H -3.03171 -3.38059 -1.49671

H -1.82009 -3.91238 -0.33043

C -2.34914 -0.74776 -1.68816

H -2.30223 0.29382 -1.34330

H -3.37872 -0.93537 -2.01816

H -1.69467 -0.85342 -2.55997

C 0.60106 -2.40339 1.18565

C 1.08907 -3.67093 0.48263

H 0.27992 -4.24998 0.02705

H 1.56590 -4.31679 1.23167

H 1.84528 -3.45238 -0.27812

C -0.31534 -2.76561 2.35706

H -0.76434 -1.88260 2.82761

H 0.27926 -3.28180 3.12174

H -1.11805 -3.44798 2.05710

C 1.81751 -1.61801 1.69277

H 2.46361 -1.28391 0.87111

H 2.41907 -2.27205 2.33712

H 1.53054 -0.74301 2.28835

C -2.61659 1.40034 2.77797

C -1.32095 0.76238 2.54542

H -3.49888 1.28693 2.12786

H -0.73922 0.53305 3.44183

**ts_8-8’_singlet**

Au -0.42312 1.01856 0.90095

P -0.32817 -1.19227 0.01914

N 0.98784 1.37501 -0.78572

C 1.41854 2.61897 -0.85871

C 2.38308 2.94646 -1.85047

H 2.76188 3.96042 -1.94279

C 2.83885 1.96070 -2.69202

H 3.57882 2.19712 -3.45593

C 2.80892 -0.44596 -3.39220

H 3.54535 -0.25922 -4.17277

C 2.30971 -1.71330 -3.18635

H 2.64930 -2.54072 -3.80621

C 1.35856 -1.96000 -2.17161

H 1.00232 -2.97853 -2.04296

C 0.88626 -0.94675 -1.35448

C 1.40700 0.36646 -1.56617

C 2.37073 0.62359 -2.58001

C 0.83027 3.54962 0.12658

C -0.09069 3.04196 1.09103

C -0.65957 3.94882 1.97702

H -1.38826 3.61323 2.71852

C -0.33932 5.31022 1.95634

H -0.80190 5.99049 2.67029

C 0.57117 5.79249 1.02125

H 0.82691 6.84990 0.99463

C 1.14671 4.91299 0.10844

H 1.84301 5.30618 -0.63000

C -1.93267 -1.72119 -0.81371

C -2.92800 -2.14417 0.26930

H -3.91775 -2.26799 -0.18956

H -3.02012 -1.39268 1.06156

H -2.66224 -3.10608 0.72189

C -1.77433 -2.83158 -1.85223

H -1.16264 -2.51186 -2.70182

H -2.76839 -3.08001 -2.24724

H -1.35318 -3.75272 -1.43801

C -2.43766 -0.45441 -1.51632

H -2.64052 0.35498 -0.80423

H -3.37399 -0.68309 -2.04202

H -1.72197 -0.08585 -2.26212

C 0.43447 -2.46934 1.17877

C 0.34335 -3.91495 0.69273

H -0.69177 -4.25981 0.60143

H 0.83800 -4.56568 1.42619

H 0.85114 -4.07343 -0.26399

C -0.27775 -2.33566 2.52987

H -0.14986 -1.32677 2.93832

H 0.16687 -3.04298 3.24200

H -1.34918 -2.55028 2.47300

C 1.90133 -2.06027 1.35426

H 2.48953 -2.22497 0.44562

H 2.34742 -2.66196 2.15684

H 2.00105 -1.00349 1.63706

N -2.44051 0.58240 2.57106

C -1.53232 1.10113 3.37175

C -2.76209 1.69588 3.31187

H -0.56155 0.87684 3.80512

H -3.40886 2.56356 3.24681

**ts_2^CHCHN^-2^CH2CN^_singlet**

Au -0.33432 1.07320 0.83147

P -0.31905 -1.19451 0.06734

N 0.91255 1.37925 -0.77033

N -3.26339 2.60395 3.23430

C 1.34047 2.63608 -0.92768

C 2.16905 2.95795 -2.03102

H 2.51213 3.97894 -2.16672

C 2.53232 1.97788 -2.91527

H 3.17340 2.21328 -3.76383

C 2.43606 -0.41107 -3.60921

H 3.08264 -0.20057 -4.45987

C 1.97286 -1.68723 -3.37774

H 2.25157 -2.50221 -4.04242

C 1.13318 -1.94687 -2.27730

H 0.78897 -2.96545 -2.12227

C 0.74814 -0.94171 -1.40329

C 1.23953 0.37198 -1.62768

C 2.08221 0.64347 -2.73971

C 0.90555 3.57122 0.10066

C 0.06151 3.06266 1.12275

C -0.30851 3.90647 2.16122

H -0.93419 3.55965 2.97825

C 0.11215 5.23990 2.17828

H -0.19599 5.88461 2.99997

C 0.91702 5.74592 1.15963

H 1.23834 6.78529 1.17725

C 1.31851 4.91046 0.12509

H 1.96246 5.30645 -0.65757

C -1.98445 -1.79987 -0.56933

C -2.84136 -2.23581 0.62151

H -3.85646 -2.45072 0.26329

H -2.92185 -1.45097 1.38161

H -2.46487 -3.15031 1.09224

C -1.88214 -2.93897 -1.58490

H -1.40297 -2.61790 -2.51509

H -2.90038 -3.25745 -1.84428

H -1.35942 -3.81882 -1.19725

C -2.61703 -0.58024 -1.25010

H -2.79548 0.23561 -0.54022

H -3.58478 -0.87184 -1.67833

H -1.99667 -0.19920 -2.07130

C 0.62278 -2.36256 1.20906

C 0.58785 -3.81866 0.74713

H -0.42076 -4.24413 0.77660

H 1.20848 -4.41540 1.42857

H 1.00016 -3.95643 -0.25780

C 0.04269 -2.24858 2.62299

H 0.14519 -1.23380 3.02291

H 0.60597 -2.91897 3.28481

H -1.00887 -2.54739 2.68187

C 2.06660 -1.84703 1.23083

H 2.57375 -1.97950 0.26938

H 2.62949 -2.40941 1.98669

H 2.12050 -0.78598 1.50816

C -2.43146 1.79862 2.88060

C -1.62756 0.81770 2.33507

H -3.28530 1.49384 2.13830

H -1.75681 -0.16485 2.78266

**ts_2^CHCHN^-2^CHCHN’^_triplet**

Au -0.25181 1.06372 0.87593

P -0.31306 -1.19180 0.06342

N 0.91303 1.38308 -0.79764

N -2.87442 2.54971 3.33364

C 1.35269 2.63616 -0.94712

C 2.16209 2.95942 -2.06440

H 2.51857 3.97644 -2.19593

C 2.48724 1.98417 -2.96958

H 3.10663 2.22225 -3.83345

C 2.33227 -0.39442 -3.69475

H 2.94849 -0.18153 -4.56709

C 1.85213 -1.66509 -3.46735

H 2.08284 -2.47180 -4.15993

C 1.05960 -1.93150 -2.33358

H 0.70024 -2.94575 -2.18438

C 0.73704 -0.93827 -1.42155

C 1.22122 0.37689 -1.65986

C 2.02795 0.65227 -2.79723

C 0.94376 3.56649 0.09865

C 0.13135 3.05221 1.14216

C -0.27242 3.89539 2.16707

H -0.91541 3.53637 2.96854

C 0.11656 5.23925 2.16473

H -0.21288 5.89018 2.97318

C 0.91451 5.74812 1.14168

H 1.21368 6.79428 1.14787

C 1.32693 4.91427 0.10865

H 1.94828 5.32120 -0.68673

C -2.00139 -1.73811 -0.56160

C -3.01645 -1.57372 0.57301

H -4.01400 -1.82272 0.18907

H -3.04728 -0.54145 0.93378

H -2.81940 -2.23174 1.42447

C -2.02652 -3.17612 -1.07815

H -1.33383 -3.33884 -1.90990

H -3.03404 -3.39330 -1.45669

H -1.81422 -3.90674 -0.29065

C -2.35542 -0.76103 -1.68957

H -2.30820 0.28513 -1.35896

H -3.38630 -0.95365 -2.01287

H -1.70431 -0.87822 -2.56236

C 0.60882 -2.39540 1.17837

C 1.11733 -3.64425 0.45650

H 0.31861 -4.22695 -0.01264

H 1.60011 -4.29582 1.19675

H 1.87344 -3.40185 -0.29680

C -0.30427 -2.79494 2.33994

H -0.75926 -1.92913 2.83368

H 0.29451 -3.32596 3.09110

H -1.10256 -3.47512 2.02385

C 1.81329 -1.60257 1.70245

H 2.45713 -1.24838 0.88745

H 2.42204 -2.25828 2.33833

H 1.51411 -0.73945 2.30915

C -2.61578 1.43351 2.83231

C -1.29062 0.84471 2.56839

H -3.50009 0.80403 2.58316

H -0.84259 0.24466 3.36348

**ts_2^CHCHN^-8_triplet**

Au -0.95854 -0.79656 1.79604

P 0.00679 -1.95894 3.68198

N -0.41753 -2.61388 0.78329

N -2.89475 0.51819 2.81739

C -0.71817 -2.63342 -0.51086

C -0.45513 -3.80955 -1.25999

H -0.69681 -3.84589 -2.31795

C 0.09846 -4.89677 -0.63610

H 0.30226 -5.80663 -1.19948

C 0.97287 -5.95095 1.44677

H 1.18579 -6.87353 0.90855

C 1.24354 -5.84272 2.79220

H 1.67531 -6.68126 3.33466

C 0.96549 -4.64444 3.48052

H 1.19893 -4.59895 4.54011

C 0.41299 -3.54479 2.84270

C 0.12843 -3.65617 1.45102

C 0.41031 -4.86065 0.74817

C -1.30907 -1.40415 -1.04532

C -1.54545 -0.33961 -0.14013

C -2.09016 0.83989 -0.62788

H -2.27475 1.68075 0.04040

C -2.40739 0.97984 -1.98230

H -2.83416 1.91413 -2.34487

C -2.17885 -0.07032 -2.86958

H -2.42407 0.03806 -3.92420

C -1.63099 -1.25954 -2.40198

H -1.45108 -2.07000 -3.10579

C -1.19646 -2.36762 5.07254

C -1.80529 -1.05725 5.58530

H -2.58804 -1.30364 6.31453

H -2.27896 -0.48500 4.78062

H -1.07719 -0.42149 6.09740

C -0.58252 -3.13684 6.24123

H -0.17556 -4.10925 5.94757

H -1.37170 -3.33491 6.97880

H 0.19790 -2.56520 6.75474

C -2.30276 -3.19725 4.40803

H -2.76075 -2.66455 3.56438

H -3.09500 -3.38326 5.14454

H -1.94096 -4.16897 4.05405

C 1.66750 -1.23661 4.20876

C 2.58997 -2.22936 4.91680

H 2.14848 -2.65758 5.82230

H 3.50203 -1.69904 5.22121

H 2.90011 -3.04399 4.25469

C 1.41993 -0.02634 5.11128

H 0.69237 0.67535 4.68662

H 2.36305 0.51930 5.24411

H 1.07036 -0.31781 6.10730

C 2.33600 -0.78715 2.90305

H 2.47260 -1.62183 2.20399

H 3.33098 -0.38188 3.12918

H 1.76616 -0.00236 2.39031

C -2.61821 1.78494 2.54482

C -1.37850 1.24760 2.55119

H -3.20614 2.69937 2.47380

H -0.45815 1.56028 3.04887

**ts_2^CHCHN^-8_singlet**

Au -0.87880 -0.83040 1.75761

P 0.04262 -1.93469 3.67310

N -0.40758 -2.58690 0.79784

N -3.51477 0.24846 2.84547

C -0.71862 -2.62133 -0.50172

C -0.48483 -3.80584 -1.24164

H -0.73684 -3.84173 -2.29710

C 0.05428 -4.89874 -0.61555

H 0.23597 -5.81650 -1.17360

C 0.92978 -5.95295 1.46387

H 1.12079 -6.88123 0.92748

C 1.21631 -5.84433 2.80598

H 1.63938 -6.68783 3.34751

C 0.96474 -4.63895 3.49105

H 1.20825 -4.59091 4.54829

C 0.42443 -3.53476 2.85031

C 0.12694 -3.64521 1.46459

C 0.37967 -4.85636 0.76478

C -1.27846 -1.38506 -1.03879

C -1.45523 -0.31255 -0.12947

C -1.93684 0.90173 -0.60020

H -2.05609 1.74698 0.07316

C -2.26462 1.05452 -1.95057

H -2.64250 2.01175 -2.30689

C -2.10995 -0.00584 -2.84258

H -2.36876 0.11877 -3.89204

C -1.61501 -1.22312 -2.38956

H -1.48724 -2.04082 -3.09631

C -1.18163 -2.30102 5.05533

C -1.77163 -0.98336 5.56964

H -2.52157 -1.22461 6.33395

H -2.28766 -0.43144 4.77881

H -1.02823 -0.33435 6.04113

C -0.57028 -3.07588 6.22252

H -0.16984 -4.05087 5.92868

H -1.36175 -3.26911 6.95856

H 0.21265 -2.50869 6.73715

C -2.30101 -3.11504 4.39360

H -2.76797 -2.56667 3.56536

H -3.08520 -3.29872 5.13918

H -1.95332 -4.08839 4.02971

C 1.70873 -1.21400 4.17936

C 2.61156 -2.20915 4.90978

H 2.16217 -2.61038 5.82361

H 3.53092 -1.68645 5.20479

H 2.91112 -3.04202 4.26563

C 1.47787 0.01992 5.05404

H 0.78509 0.73481 4.59652

H 2.43551 0.53782 5.19338

H 1.10278 -0.24159 6.04892

C 2.38431 -0.80346 2.86465

H 2.50551 -1.65358 2.18137

H 3.38749 -0.41595 3.08502

H 1.83193 -0.01275 2.34246

C -2.87512 1.27603 2.51589

C -1.46000 0.89920 2.56236

H -3.28942 2.28482 2.36637

H -0.87453 1.33853 3.37652

**ts_2^NCHCH^-8_singlet**

Au -1.05113 -0.92220 1.84736

P -0.02511 -1.99045 3.71607

N -0.45153 -2.59187 0.82569

N -1.83684 0.66575 2.75638

C -0.75317 -2.60067 -0.47669

C -0.34959 -3.69925 -1.27362

H -0.58183 -3.70954 -2.33431

C 0.32527 -4.74271 -0.69548

H 0.63674 -5.59514 -1.29797

C 1.30483 -5.78578 1.34451

H 1.61985 -6.65271 0.76561

C 1.56186 -5.71133 2.69504

H 2.08304 -6.52193 3.20008

C 1.15636 -4.58236 3.43540

H 1.38271 -4.55619 4.49793

C 0.48994 -3.52284 2.84068

C 0.21502 -3.60223 1.44732

C 0.62543 -4.73371 0.69135

C -1.51486 -1.44569 -0.94483

C -1.83529 -0.46187 0.02212

C -2.61602 0.62764 -0.33643

H -2.89805 1.36860 0.40958

C -3.06147 0.76223 -1.65560

H -3.67576 1.61800 -1.93210

C -2.73000 -0.19252 -2.61683

H -3.07862 -0.08000 -3.64145

C -1.96418 -1.29893 -2.26376

H -1.72913 -2.04627 -3.01950

C -1.33578 -2.48557 4.96760

C -1.71096 -1.24002 5.77513

H -2.60508 -1.46395 6.37121

H -1.95171 -0.38755 5.12677

H -0.91975 -0.94834 6.47426

C -0.91012 -3.62715 5.89005

H -0.74787 -4.55977 5.33930

H -1.72364 -3.81505 6.60310

H -0.01485 -3.39960 6.47592

C -2.53959 -2.93989 4.13158

H -2.95745 -2.12222 3.53234

H -3.33031 -3.28470 4.81046

H -2.29154 -3.77755 3.46740

C 1.54443 -1.19943 4.39066

C 2.11754 -1.92349 5.60800

H 1.45593 -1.85869 6.47793

H 3.06187 -1.43651 5.88457

H 2.34846 -2.97571 5.41387

C 1.24154 0.25891 4.74688

H 0.91896 0.82140 3.86591

H 2.16669 0.72250 5.11352

H 0.48878 0.36452 5.53324

C 2.54581 -1.22058 3.22997

H 2.85320 -2.23602 2.95790

H 3.44496 -0.66940 3.53406

H 2.14284 -0.71906 2.34036

C -1.15512 1.74708 2.54589

C -0.02082 1.63493 1.71268

H -1.49063 2.69850 2.98612

H 0.06131 2.14618 0.74613

**ts_2^NCHCH^-2^CHCHN^_triplet**

Au -0.97255 -0.85725 1.78475

P 0.00684 -1.93941 3.67765

N -0.45342 -2.59237 0.80014

N -2.11596 0.59182 2.85374

C -0.79113 -2.63428 -0.49265

C -0.43299 -3.76539 -1.26636

H -0.69719 -3.80343 -2.31896

C 0.24448 -4.80069 -0.67774

H 0.52504 -5.67582 -1.26250

C 1.27872 -5.79179 1.36081

H 1.56713 -6.67854 0.79828

C 1.57854 -5.68037 2.70000

H 2.10812 -6.48069 3.21260

C 1.20034 -4.52959 3.42009

H 1.44984 -4.48100 4.47625

C 0.52514 -3.48065 2.81557

C 0.21524 -3.59448 1.43270

C 0.58930 -4.75251 0.69846

C -1.52952 -1.47378 -0.97526

C -1.77637 -0.43827 -0.04092

C -2.51641 0.67198 -0.42129

H -2.72760 1.46625 0.29069

C -3.00497 0.76904 -1.72711

H -3.58824 1.64134 -2.01853

C -2.75229 -0.23957 -2.65761

H -3.13339 -0.15427 -3.67318

C -2.01983 -1.35990 -2.28412

H -1.84070 -2.14719 -3.01411

C -1.31530 -2.44966 4.91613

C -1.67365 -1.23575 5.77615

H -2.56544 -1.47897 6.36825

H -1.91612 -0.35812 5.16485

H -0.87718 -0.97886 6.48277

C -0.90138 -3.63086 5.79332

H -0.76000 -4.54554 5.20819

H -1.71201 -3.82889 6.50688

H 0.00291 -3.43917 6.37863

C -2.52717 -2.85711 4.06772

H -2.93769 -2.01152 3.50431

H -3.31739 -3.22269 4.73635

H -2.28902 -3.67091 3.37090

C 1.56638 -1.15760 4.38919

C 2.14552 -1.90265 5.59175

H 1.47133 -1.88828 6.45420

H 3.06984 -1.39518 5.89779

H 2.41437 -2.93915 5.36691

C 1.25086 0.28600 4.79229

H 0.85498 0.86421 3.95202

H 2.18278 0.76631 5.11759

H 0.54142 0.35378 5.62163

C 2.57533 -1.14254 3.23496

H 2.88937 -2.14997 2.94114

H 3.47014 -0.59327 3.55479

H 2.17334 -0.63132 2.35067

C -1.39439 1.67579 2.64045

C -0.28280 1.33819 1.89263

H -1.69523 2.65344 3.03512

H 0.55557 1.88092 1.46039

**ts_2^NCHCH^-2^NCCH2^_singlet**

Au -0.49503 1.07108 0.63373

P -0.36843 -1.20325 -0.05927

N 0.94511 1.37224 -0.77936

N -2.00727 0.84512 1.87917

C 1.39348 2.62802 -0.87834

C 2.39308 2.93233 -1.83365

H 2.76314 3.94954 -1.92016

C 2.87882 1.94028 -2.64516

H 3.64538 2.16461 -3.38591

C 2.85985 -0.45262 -3.34157

H 3.62398 -0.25301 -4.09136

C 2.35364 -1.72228 -3.17458

H 2.71355 -2.54203 -3.79282

C 1.36829 -1.97199 -2.19782

H 0.99814 -2.98780 -2.08518

C 0.87506 -0.96165 -1.38817

C 1.39672 0.35079 -1.55823

C 2.39527 0.61061 -2.53574

C 0.76786 3.58310 0.03260

C -0.23614 3.07438 0.89347

C -0.89996 3.93750 1.75352

H -1.69657 3.56751 2.39604

C -0.56080 5.29434 1.78357

H -1.08679 5.96649 2.46021

C 0.43807 5.79468 0.94854

H 0.69562 6.85149 0.97692

C 1.10014 4.94351 0.07015

H 1.87110 5.34818 -0.58313

C -1.96610 -1.75580 -0.87454

C -2.97987 -2.05800 0.23251

H -3.96581 -2.20304 -0.22798

H -3.06117 -1.22645 0.94414

H -2.73909 -2.97844 0.77595

C -1.80530 -2.95254 -1.81137

H -1.17605 -2.71663 -2.67618

H -2.79683 -3.21797 -2.20124

H -1.40724 -3.84132 -1.31271

C -2.43531 -0.54048 -1.68584

H -2.67040 0.31580 -1.04262

H -3.35331 -0.80783 -2.22506

H -1.69412 -0.23186 -2.43414

C 0.39594 -2.36874 1.20395

C 0.36952 -3.83323 0.76737

H -0.65009 -4.22448 0.69042

H 0.88841 -4.43232 1.52726

H 0.88704 -4.00658 -0.18233

C -0.35554 -2.20406 2.52940

H -0.26559 -1.19117 2.93580

H 0.08725 -2.88925 3.26392

H -1.41868 -2.44909 2.45212

C 1.84193 -1.89549 1.39182

H 2.45967 -2.07404 0.50529

H 2.28706 -2.44946 2.22811

H 1.89519 -0.82825 1.64469

C -1.82064 1.01370 3.12223

H -0.98618 1.93697 4.86483

C -0.95839 1.06377 4.20071

H -2.73167 0.91511 3.81603

**ts_2-2^NCHCH’^_triplet**

Au 2.02571 9.77285 5.48184

P 2.13334 7.51311 4.74273

N 3.48597 10.07658 4.05599

N 0.65700 9.60798 6.84875

C 3.91403 11.33703 3.94508

C 4.93345 11.64101 3.01036

H 5.29293 12.66132 2.91515

C 5.45494 10.64114 2.23006

H 6.23601 10.86437 1.50422

C 5.47494 8.23689 1.56510

H 6.25135 8.43219 0.82683

C 4.96698 6.96784 1.73158

H 5.33658 6.14533 1.12276

C 3.96391 6.72121 2.69164

H 3.58569 5.70731 2.79254

C 3.46029 7.73415 3.49191

C 3.97397 9.04950 3.31162

C 4.98835 9.30594 2.35020

C 3.24585 12.29828 4.82243

C 2.24493 11.78857 5.68657

C 1.53139 12.65677 6.50135

H 0.73370 12.27877 7.14105

C 1.82119 14.02562 6.48710

H 1.25887 14.70244 7.12881

C 2.81978 14.52849 5.65347

H 3.04093 15.59390 5.64761

C 3.52819 13.67030 4.81823

H 4.29378 14.07990 4.16167

C 0.57210 7.02842 3.81332

C -0.63065 7.23916 4.74065

H -1.54802 7.10453 4.15341

H -0.65511 8.24592 5.17399

H -0.66227 6.51893 5.56282

C 0.59485 5.59157 3.29494

H 1.43524 5.39711 2.61978

H -0.32344 5.41353 2.72001

H 0.61273 4.85620 4.10607

C 0.47512 8.01248 2.64081

H 0.46837 9.05709 2.97904

H -0.47130 7.83727 2.11360

H 1.28801 7.88606 1.91724

C 2.76599 6.31616 6.04010

C 3.32525 5.01327 5.46745

H 2.60216 4.46397 4.85674

H 3.60270 4.36068 6.30563

H 4.23422 5.18023 4.88042

C 1.61718 6.02487 7.00930

H 1.12354 6.94082 7.35653

H 2.02399 5.50908 7.88887

H 0.86389 5.36448 6.56689

C 3.88780 7.07204 6.76447

H 4.68988 7.37406 6.07897

H 4.33336 6.40754 7.51621

H 3.51498 7.95921 7.28881

C 1.69916 9.76258 8.63424

H 2.06065 10.75071 8.40088

C 1.59615 8.80222 9.40278

H 1.38647 7.90585 9.95399

**ts_2-2^NCHCH^_triplet**

Au 2.05970 9.75892 5.46736

P 2.17241 7.49967 4.72146

N 3.49734 10.07645 4.02687

N 0.67592 9.56799 6.81438

C 3.91529 11.34063 3.91409

C 4.90537 11.65800 2.95229

H 5.25434 12.68168 2.85421

C 5.41050 10.66771 2.14989

H 6.16912 10.90136 1.40379

C 5.42920 8.26875 1.46774

H 6.18495 8.47507 0.71124

C 4.93560 6.99456 1.63860

H 5.29648 6.17874 1.01573

C 3.95817 6.73474 2.62114

H 3.59027 5.71731 2.72524

C 3.46609 7.73896 3.43950

C 3.96848 9.05847 3.25863

C 4.95580 9.32853 2.27297

C 3.27489 12.28832 4.82410

C 2.30147 11.76570 5.71122

C 1.63985 12.61334 6.58788

H 0.89148 12.21539 7.27091

C 1.94106 13.97908 6.59517

H 1.41901 14.64174 7.28400

C 2.90364 14.49847 5.72868

H 3.13273 15.56218 5.74165

C 3.57033 13.65811 4.84352

H 4.31520 14.07773 4.16967

C 0.59702 6.98707 3.83056

C -0.58427 7.16346 4.79177

H -1.51407 7.01375 4.22812

H -0.61825 8.16516 5.23578

H -0.57622 6.43335 5.60596

C 0.63246 5.55522 3.29923

H 1.46105 5.37827 2.60519

H -0.29492 5.36713 2.74234

H 0.67832 4.81460 4.10444

C 0.45066 7.97982 2.67045

H 0.43071 9.02074 3.01923

H -0.50400 7.78994 2.16347

H 1.24873 7.87749 1.92676

C 2.86506 6.32278 6.00764

C 3.39244 5.00774 5.43266

H 2.63702 4.44660 4.87456

H 3.71529 4.37275 6.26815

H 4.26822 5.16009 4.79335

C 1.76715 6.05694 7.04107

H 1.30601 6.98398 7.40268

H 2.21275 5.54898 7.90594

H 0.98274 5.40165 6.64797

C 4.02269 7.08784 6.66206

H 4.79205 7.37257 5.93326

H 4.49916 6.43811 7.40745

H 3.67271 7.98621 7.18291

C 1.45567 9.62215 8.73324

H 0.52543 9.29653 9.16432

C 2.62800 10.00299 8.75628

H 3.63108 10.36691 8.64653

**ts_2-2^NCHCH’^_singlet**

Au 2.01997 9.79464 5.46616

P 2.12310 7.52996 4.74397

N 3.48413 10.08379 4.03981

N 0.66240 9.64348 6.82560

C 3.91540 11.34230 3.91820

C 4.93529 11.63625 2.98083

H 5.29600 12.65522 2.87613

C 5.45714 10.62808 2.21150

H 6.23965 10.84325 1.48478

C 5.47881 8.21622 1.57405

H 6.25818 8.40281 0.83665

C 4.97006 6.94917 1.75316

H 5.34220 6.11962 1.15556

C 3.96283 6.71378 2.71165

H 3.58348 5.70132 2.82265

C 3.45682 7.73610 3.49833

C 3.97229 9.04905 3.30604

C 4.98969 9.29439 2.34503

C 3.25403 12.31095 4.79243

C 2.25848 11.80895 5.66695

C 1.56118 12.68059 6.49200

H 0.77311 12.30424 7.14453

C 1.85740 14.04796 6.47153

H 1.30724 14.72861 7.11974

C 2.84648 14.54484 5.62289

H 3.07138 15.60944 5.61114

C 3.54215 13.68175 4.78192

H 4.30263 14.08649 4.11644

C 0.56446 7.04321 3.81147

C -0.64256 7.26954 4.72946

H -1.55708 7.14155 4.13631

H -0.65962 8.27840 5.15876

H -0.68691 6.55336 5.55454

C 0.58465 5.60072 3.30885

H 1.42598 5.39742 2.63739

H -0.33296 5.41865 2.73409

H 0.59935 4.87400 4.12779

C 0.47884 8.01496 2.62783

H 0.47332 9.06310 2.95491

H -0.46458 7.83717 2.09616

H 1.29615 7.87784 1.91126

C 2.74189 6.34527 6.05890

C 3.30686 5.03811 5.50218

H 2.58971 4.48359 4.88910

H 3.57695 4.39270 6.34823

H 4.22094 5.20171 4.92217

C 1.58270 6.06397 7.01867

H 1.08724 6.98421 7.35191

H 1.98012 5.55639 7.90725

H 0.83333 5.40012 6.57480

C 3.85557 7.10758 6.78887

H 4.66130 7.41246 6.10890

H 4.29860 6.44703 7.54555

H 3.47274 7.99279 7.30897

C 1.78092 9.78928 8.67862

H 2.19804 10.73616 8.38181

C 1.57625 8.83914 9.43159

H 1.29528 7.97124 9.99677

**ts_2-2^NCHCH^_singlet**

Au 2.05978 9.74961 5.46714

P 2.16933 7.49556 4.71212

N 3.49625 10.07201 4.02519

N 0.70124 9.53811 6.81431

C 3.91516 11.33611 3.91477

C 4.90418 11.65536 2.95249

H 5.25316 12.67920 2.85611

C 5.40859 10.66662 2.14773

H 6.16651 10.90162 1.40135

C 5.42795 8.26825 1.46283

H 6.18323 8.47574 0.70617

C 4.93556 6.99339 1.63279

H 5.29710 6.17845 1.00914

C 3.95841 6.73191 2.61510

H 3.59110 5.71416 2.71863

C 3.46575 7.73547 3.43396

C 3.96746 9.05552 3.25480

C 4.95430 9.32705 2.26921

C 3.27628 12.28158 4.82811

C 2.30448 11.75672 5.71545

C 1.64053 12.60247 6.59245

H 0.89124 12.20163 7.27273

C 1.94020 13.96843 6.60156

H 1.41685 14.62970 7.29077

C 2.90236 14.49008 5.73580

H 3.12988 15.55412 5.74993

C 3.56974 13.65187 4.84915

H 4.31314 14.07352 4.17493

C 0.59462 6.98400 3.82054

C -0.58823 7.17165 4.77770

H -1.51744 7.02345 4.21270

H -0.61827 8.17699 5.21466

H -0.58608 6.44739 5.59702

C 0.62815 5.54804 3.29996

H 1.45683 5.36552 2.60737

H -0.29927 5.35671 2.74425

H 0.67356 4.81312 4.11046

C 0.45403 7.96901 2.65325

H 0.43671 9.01229 2.99500

H -0.50025 7.77863 2.14576

H 1.25318 7.85907 1.91186

C 2.85405 6.32215 6.00497

C 3.39888 5.01387 5.43078

H 2.65519 4.45011 4.85949

H 3.71526 4.37685 6.26716

H 4.28173 5.17654 4.80373

C 1.74454 6.04497 7.02290

H 1.26649 6.96781 7.37412

H 2.18414 5.54464 7.89526

H 0.97404 5.37889 6.62037

C 3.99654 7.09422 6.67797

H 4.76930 7.39642 5.95971

H 4.47434 6.44148 7.41991

H 3.63146 7.98228 7.20642

C 1.52866 9.62928 8.80354

H 0.60944 9.23639 9.19729

C 2.66530 10.09388 8.78745

H 3.63871 10.52848 8.66897
